# A Supramolecular Approach to Structure-Based Design with A Focus on Synthons Hierarchy in Ornithine-Derived Ligands: Review, Synthesis, Experimental and in Silico Studies

**DOI:** 10.3390/molecules25051135

**Published:** 2020-03-03

**Authors:** Joanna Bojarska, Milan Remko, Martin Breza, Izabela D. Madura, Krzysztof Kaczmarek, Janusz Zabrocki, Wojciech M. Wolf

**Affiliations:** 1 Institute of General and Ecological Chemistry, Faculty of Chemistry, Lodz University of Technology, Żeromskiego 116, 90-924 Lodz, Poland; wmwolf@p.lodz.pl; 2 Remedika, Sustekova, 1 85104 Bratislava, Slovakia; milan.remko@gmail.com; 3 Department of Physical Chemistry, Slovak Technical University, Radlinskeho 9, SK-81237 Bratislava, Slovakia; martin.breza@stuba.sk; 4 Warsaw University of Technology, Faculty of Chemistry, Noakowskiego 3, 00-664 Warszawa, Poland; izabela@ch.pw.edu.pl; 5 Institute of Organic Chemistry, Lodz University of Technology, Faculty of Chemistry, Żeromskiego 116, 90-924 Lodz, Poland; krzysztof.kaczmarek@p.lodz.pl (K.K.); janusz.zabrocki@p.lodz.pl (J.Z.)

**Keywords:** ornithine, supramolecular synthon, π^…^π, C-H^…^π, C=O (lone pair)^…^π and N-O^…^π interactions, DFT, electrostatic molecular potential, H-bond energies, molecular (Hirshfeld) surface, SwissADME, drug-likeness, ProTox II

## Abstract

The success of innovative drugs depends on an interdisciplinary and holistic approach to their design and development. The supramolecular architecture of living systems is controlled by non-covalent interactions to a very large extent. The latter are prone to extensive cooperation and like a virtuoso play a symphony of life. Thus, the design of effective ligands should be based on thorough knowledge on the interactions at either a molecular or high topological level. In this work, we emphasize the importance of supramolecular structure and ligand-based design keeping the potential of supramolecular H-bonding synthons in focus. In this respect, the relevance of supramolecular chemistry for advanced therapies is appreciated and undisputable. It has developed tools, such as Hirshfeld surface analysis, using a huge data on supramolecular interactions in over one million structures which are deposited in the Cambridge Structure Database (CSD). In particular, molecular interaction surfaces are useful for identification of macromolecular active sites followed by *in silico* docking experiments. Ornithine-derived compounds are a new, promising class of multi-targeting ligands for innovative therapeutics and cosmeceuticals. In this work, we present the synthesis together with the molecular and supramolecular structure of a novel ornithine derivative, namely *N*-α,*N*-δ)-dibenzoyl-(α)-hydroxymethylornithine, **1**. It was investigated by modern experimental and *in silico* methods in detail. The incorporation of an aromatic system into the ornithine core induces stacking interactions, which are vital in biological processes. In particular, rare C=O^…^π intercontacts have been identified in **1**. Supramolecular interactions were analyzed in all structures of ornithine derivatives deposited in the CSD. The influence of substituent was assessed by the Hirshfeld surface analysis. It revealed that the crystal packing is stabilized mainly by H^…^O, O^…^H, C^…^H, Cl (Br, F)^…^H and O^…^O interactions. Additionally, π^…^π, C-H^…^π and N-O^…^π interactions were also observed. All relevant H-bond energies were calculated using the Lippincott and Schroeder H-bond model. A library of synthons is provided. In addition, the large synthons (*Long-Range Synthon Aufbau Module*) were considered. The DFT optimization either *in vacuo* or *in solutio* yields very similar molecular species. The major difference with the relevant crystal structure was related to the conformation of terminal benzoyl C15-C20 ring. Furthermore, *in silico* prediction of the extensive physicochemical ADME profile (absorption, distribution, metabolism and excretion) related to the drug-likeness and medicinal chemistry friendliness revealed that a novel ornithine derivative **1** has the potential to be a new drug candidate. It has shown good *in silico* absorption and very low toxicity.

## 1. Introduction

Recently, short peptides have attracted an increasing interest due to their numerous advantages and applications, *inter alia* in drug delivery, cancer therapy or immunology. This work follows our interest in the supramolecular chemistry of modified amino acids and oligopeptides [1,2,3,4,5,6,7]. The former are building blocks for proteins and the topology of their non-covalent interactions is important to understand the protein activity and folding. In this paper, we emphasize the biological relevance and the medical value of the non-proteinogenic amino acid ornithine. Its derivatives show potential for cancer and other complex diseases treatments [8]. Notably, despite recent advances, cancer is expected to rank as the leading cause of death in the 21^st^ century [9]. Therefore, the design and synthesis of innovative first-in-class therapeutics become a priority objective and a major challenge for contemporary science. As recently pointed out by Spackman et al. [10,11,12,13] supramolecular chemistry approaches, including crystal engineering, are crucial for drug discovery as well as development of innovative therapies. Mostly results from the architecture of biological species are affected by numerous non-covalent interactions [14]. The discovery of how the latter arrange into networks responsible for the life functions is still a challenge. One of the possible solutions to this issue may follow from the widely accepted supramolecular synthon approach, as introduced by Desiraju [15,16]. Synthons are the design backbone. They may be regarded also as the missing pieces to the puzzle of the drug-target map. Screening of appropriate synthons from model libraries of interactions can help to design effective ligand molecules that fit the best to the protein pockets [10].

This work is organized in two main sections. The first, theoretical part is a short overview treating the newest scientific worldwide findings regarded to the above aspects. The second, experimental section is devoted to the synthesis of the novel ornithine-based compound, and holistic view at its molecular and supramolecular structure using experimental and *in silico* methods, coupled with comparative analysis with all currently known ornithine-based crystal structures, *via* a survey of interactions interplay, keeping in mind building a library of ornithine-based synthons. Furthermore, *in silico* ADME parameters, physicochemical descriptors, lipophilicity, solubility, pharmacokinetics properties, drug-like nature and medicinal chemistry friendliness were studied as *per* SwissADME online interface [17,18,19,20,21], while toxicity aspects *via* webserver ProTox II [22,23], in order to predict the behavior of ornithine-based compounds to be potential drugs. These preliminary results providing systematic molecular, supramolecular and pharmacological information could be a suitable platform facilitating the design and development of new therapeutics.

### 1.1. L-Ornithine

L-Ornithine [NH_2_ (CH_2_) (NH_3_CH_2_) COOH] is a non-essential amino acid with a highly basic side chain. It is synthesized from the *L*-glutamate in plants or through the urea cycle from arginine in mammals. It is a metabolic intermediate in the Krebs-Henseleit cycle of the liver [24]. It helps to convert highly toxic ammonia into less toxic urea, which is further eliminated *via* kidneys. *L*-Ornithine is a precursor for the synthesis of amino acids, like *L*-arginine and the *L*-citrulline [25,26]. Ornithine is a cofactor of several proteins [27,28]. It is used as a modifier of bioactive peptides, amino acids and their derivatives [29,30] and applied as a dietary supplement in either pharmaceutical or food products. It is responsible for elevation of the human growth hormone secretion and consequently the metabolism of excess body fat. Therefore, it may be used for repairing muscles and related tissues, stimulation of pancreas, regeneration of liver [31] proper functioning of the immune system, wound healing [32] and regulation of hypnotic effects [33,34]. It plays an important role in improving the quality of sleep [35] and recovery from fatigue [36,37]. It is a promising cosmeceutical linking the biological activity with a profound cosmetic action [38]. It provides significant cosmetic benefits, *inter alia* in the improvement of skin conditions by increasing plasma growth hormone and/or insulin-like growth factor-1 levels [39]. On the other hand, poly(ornithine) is promising in the treatment of acute liver injury and as a new concept in nanomedicine [40]. Natural ornithine can be found in food with the freshwater clams being a good example.

### 1.2. Role of Ornithine in Polyamine Biosynthesis

L-Ornithine is a precursor of putrescine (1,4 diaminobutane), the first polyamine, that is highly engaged in cellular functions, like: cell growth, multiplication/division, gene transcription and translation, the transformation of cells *via* the regulation of cell proliferation, cell differentiation and apoptosis, and consequently also in tumor development. The increased level of polyamine is associated with tumor promotion and progression [41,42]. The polyamine biosynthetic pathway is initiated by enzyme ornithine decarboxylase (pyridoxal-5`-phosphate-dependent enzyme). Therefore, inhibition of ornithine decarboxylase may be the essential approach for the management of cancer and other various diseases: malaria or African sleeping sickness, neglected tropical diseases [43,44,45,46,47]. This situation prompts an intensive search for new ornithine-based pharmaceuticals.

### 1.3. Ornithine-Based Inhibitors of Ornithine Decarboxylase Enzyme

The inhibitory effect of ornithine-based compounds has been recognized *inter alia* by Bey in 1978 [48]. In particular, the fluorinated ornithine analog of *α-DL*-difluoromethylornithine (eflornithine or ornidyl, DFMO) is the most famous and successful inhibitor of ornithine decarboxylase. It has been applied in therapies of hyperactivity in children or in a face hirsutism in women for almost thirty years [49,50,51,52,53]. It is also used in the treatment of African sleeping sickness and has been successfully tested against malaria. The latter poses a global health threat with over 3 billion people at danger [54]. Recent studies revealed that eflornithine is a promising agent with proven activity against several forms of cancer with endometrial [55] leukemia [56], breast [56], gastric [57], colon [56] and lung tumors [56] as well as melanoma, neuroblastoma [58] or glioma [56] being the most prone. Unfortunately, this drug has as yet not been approved for cancer prevention or treatment by the Federal Food and Drug Administration [59]. On the other hand, its chemotherapeutic value is limited by the low efficacy when applied at high doses during lengthy therapies. Adverse effects of eflornithine include seizures, gastrointestinal disorders or hearing loss. In this context, complexes with biologically active metals, like Cu (II) complex with eflornithine hydrochloride hydrate, were investigated as positive alternative with reduced side effects [60]. Moreover, *N-u*-chloroacetyl-L-ornithine was initially described as a dual-acting inhibitor against ornithine decarboxylase and histone deacetylase [61] with anticipated antitumor activity [36,37,62]. To conclude, in our opinion, ornithine and its derivatives deserve attention as an interesting alternative to the existing chemotherapeutics and cosmeceuticals.

### 1.4. Multi-Target-Directed Ligands: New Anticancer Drug Strategy

Multi-targeting inhibitors are the next generation of anticancer agents [63,64]. Cancer and related complex, multigenic diseases tend to be treated by the „one-compound-various-targets” approaches. They improve the overall therapeutic efficiency [65] and trigger search for bioactive compounds with multi-target properties [66]. It is worth noting that, the ornithine is active within several pathways and its derivatives (e.g., *N-u*-chloroacetyl-L-ornithine) can be very prospective multi-targeting ligands.

### 1.5. Supramolecular Chemistry as A “New Old” Avenue for Structure-Based Design

The success of innovative drugs for cancer and other complex diseases vitally depends on an interdisciplinary and holistic approach to their design and development. In this respect, the structure-based techniques have been dynamically evolving, and are still far from reaching their final potential and maturity [67]. To a large extent they are based on analysis of interactions among ligands and target molecules within the protein binding sites [68]. Thorough knowledge on geometries and affinities of relevant molecular species [69] is crucial. Structure-based design benefits from supramolecular chemistry, which investigates interactions of molecule with its environment [69]. In particular, forces generated *via* the differences in electrostatic potentials are usually responsible for association of the molecules, which was recently pointed out by Vepuri et al. [70] in his poetry driven sentences “*Molecules are social. They mutually interact and speak to one other in the language of electrostatics*”. This situation has prompted the development of supramolecular biochemistry in connection with novel advanced therapies [11,12,13]. The rational design of biologically important substances makes use of the supramolecular H-bond synthon approach, i.e., the structural units formed *via* synthetic operations, which involve the non-covalent interactions, e.g., hydrogen or halogen bonds [15]. This methodology was applied also to proteins treated as drug targets [71,72]. They considered mainly O (N, C)-H^...^O and N (C)–H^...^N hydrogen bonds, which were responsible for the stabilization of the secondary structure elements. Nowadays, the increasing resolution of macromolecular crystal structures allows identification of subtle molecular features and facilitates detailed synthons analysis of either protein chains or diverse ligands and cofactors [73]. The *supramolecular structure and ligand-based design* indicates the importance of thorough topological 3-D analysis of all components responsible for activity of complex bio-molecular entities.

#### 1.5.1. Supramolecular Synergy of Small Molecules and Macromolecules

Nowadays, the structure-based drug design mainly relies on data of large biological molecular species, as deposited in the Research Collaboratory for Structural Bioinformatics Protein Data Bank (RCSB PDB), [74]. In particular, optimization of the protein – ligand binding benefits from the information on the initial shape of either ligand or target macromolecule. The Cambridge Structure Database (CSD), [75,76,77] is the world`s largest collection of structure data for small molecules. It contains over one million entities. This abundance of structural information helps to investigate particular interactions in diverse crystal environments [78]. Those results can be analyzed *in silico* and extended over objects, which are difficult to be examined experimentally [14]. The latter approach may be systematized in the form of synthon libraries, which can be further transferred and applied to the macromolecular environment. This combination yields supramolecular data for the smart structure-based design. Unfortunately, it has not been fully appreciated as yet. In particular, Groom and Cole [79] pointed out that the skills of the professional drug designers should be focused on “*understanding and exploiting what small-molecule crystal structures tell them; it is just a matter of listening*”.

#### 1.5.2. Molecular Hirshfeld Surfaces and Electrostatic Potentials as Useful Tools in Drug Discovery

Hirshfeld surface (HS) analysis provides information on structural (*shape-index*, *d_norm_*, *curvedness*) and electrostatic features (molecular electrostatic potential) of a molecule onto its HS. It is useful in *inter alia* identification of all inter-contacts, in study of molecular packing modes (see Section 3.3).

Spackam [10] drew attention to the fact that computational methods for calculating molecular surface driven shape properties, including HS analysis, can be effective tools in the drug discovery process. In specific terms, they characterize and visualize molecular shapes of investigated ligands and their inter-contacts surfaces as related to neighboring molecules. The electrostatic potential (EP), when mapped on the HS couples the shape information with the surface properties and leads to an enhanced description of the molecular environment. Therefore, molecules mined from the CSD may be used for the screening of binding affinities. The latter is based on molecular shape complementarities with respect to the topology of binding sites and resulting interactions. In consequence, the EP can be used to identify a 3D “pharmacophore” model surface [10]. To conclude, the design of idealized ligands may be significantly improved by the synthon methodology.

## 2. Results and Discussion

This part of work is devoted to the synthesis of novel ornithine-based compound, and a holistic view at its molecular and supramolecular structure using experimental and *in silico* methods, coupled with comparative analysis with all currently known ornithine-based crystal structures (Table S1), *via* a survey of interactions interplay, keeping in mind building a library of ornithine-based synthons. Additionally, a preliminary prediction of drug-likeness and pharmcokinetic profile are also presented.

### 2.1. Synthesis

**Scheme 1:**
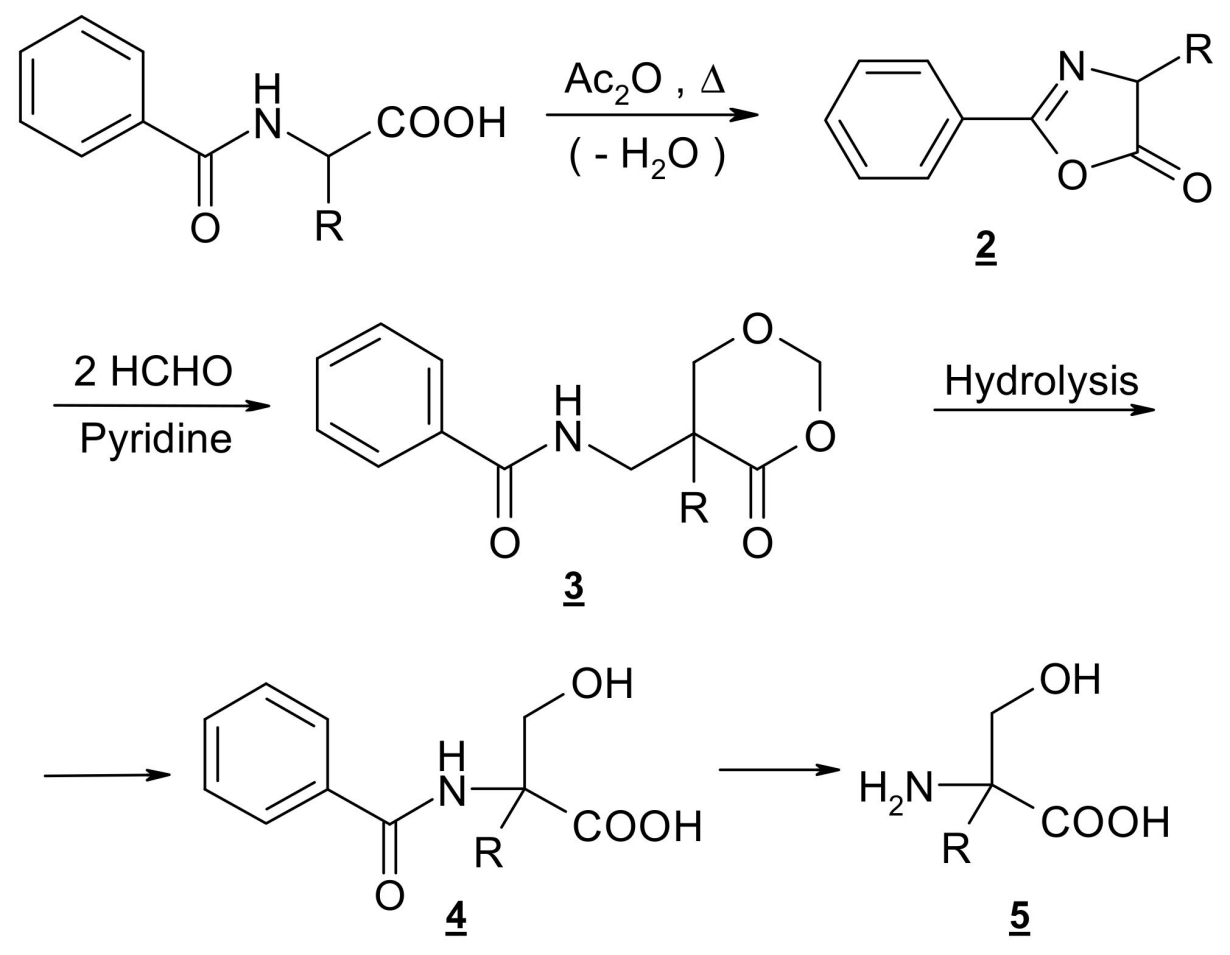
Outline of the **s**ynthesis of compound **1**.

Easy access to racemic α-hydroxymethyl amino acids is provided by the general method as developed in our laboratory [80,81,82] *via* selective -hydroxymethylation of proteinogenic amino acids. The four-step synthesis involves the formation of oxazolone **2** as a result of well-known dehydration, subsequent ring enlargement to the 4-oxo-1,3-dioxane **3** by insertion of two molecules of formaldehyde and hydrolytic degradation of **3** to the protected derivative **4** followed by hydrolysis to the amino acid (Scheme 1). Resolution into enantiomers can be attempted through fractional crystallization of diastereomeric salts of **4**. (*R,S*)–Hydroxymethylornithine, like its *N*,*N*-dibenzoyl derivative **5** (*R*=(CH_2_)_3_NHBz) has been obtained as described by [48], who followed a general procedure developed by our group, but they did not attempt to resolve the racemic mixture.

### 2.2. X-ray Crystal Structure

X-ray crystallographic analysis revealed that **1** crystallizes in the orthorhombic, chiral *P*2_1_2_1_2_1_ space group. The crystal parameters, data collection and structure refinement details are collected in Table 1.

**Figure 1:**
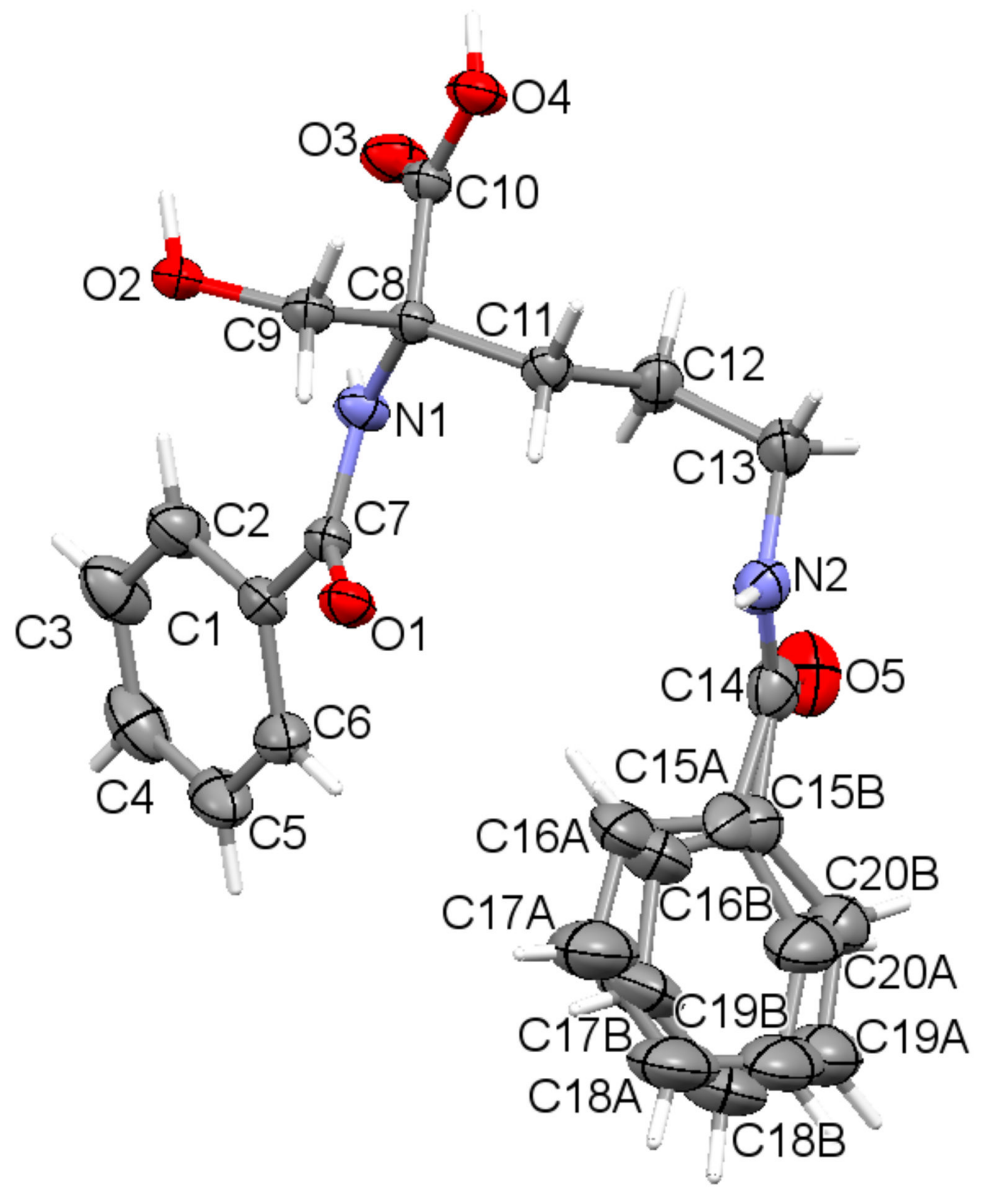
The asymmetric unit of the title compound **1**, showing disorder of the phenyl ring (C15 to C20) over two sites, with displacement ellipsoids for non-H atoms drawn at the 30% probability level, with the atom labeling. Hydrogen atoms are shown by spheres of arbitrary size.

The asymmetric unit contains a sole molecule. An ORTEP plot with the atom labeling system is depicted in Figure 1.

**Figure 2:**
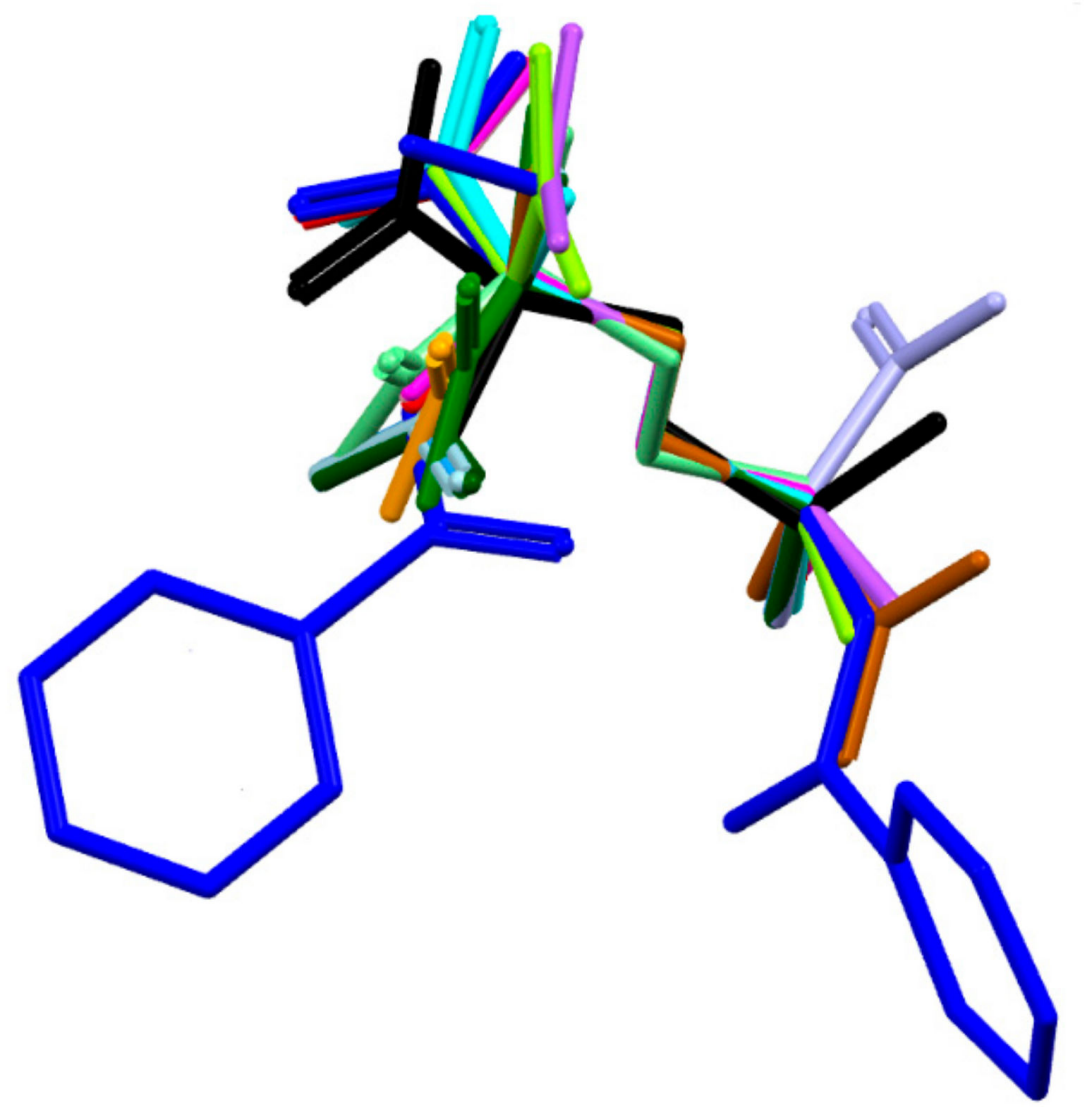
Overlay of the unique molecules in the crystal structures of **1** - dark blue, **2** - light blue and other ornithine derivatives known so far, such as: BAPKIP-light green, BAPKOV-magenta, BAPKUB-pink, BEZQOO-orange, BIHYEX- green, CAPRAM-violet, EVIJAU-red, GOTFAY-cyan, JADGED-black, ORNBDL10-lavendae, TEFMIA-light brown, VUYHII-blue, YIGMAE-light green, PUYVUA-dark green. H-atoms are omitted for clarity. CSD reference codes are described in the Section 3.3.

The molecule has one chiral center, namely *R* at C8. It adopts a folded *U*-type conformation, with the central torsion angles C1-C7-N1-C8 = 172.1 (5)° and C7-N1-C8-C11 = 63.4 (3)°. The aliphatic linker has a fully extended conformation as characterized by almost straight torsion angle C8-C11-C12-C13 = 172.2 (2)°. The plane of the linker and the plane of the carboxyl group are nearly perpendicular to each other. The carboxylate group exists in the *syn* conformation. Benzene rings and the carboxyl group are planar. Both peptide bonds adopt a planar *trans* configuration. The geometric parameters of all studied ornithine-derived structures are in good agreement what is clearly visible on a superposition plot as presented in Figure 2.

### 2.3. DFT Study

**Figure 3:**
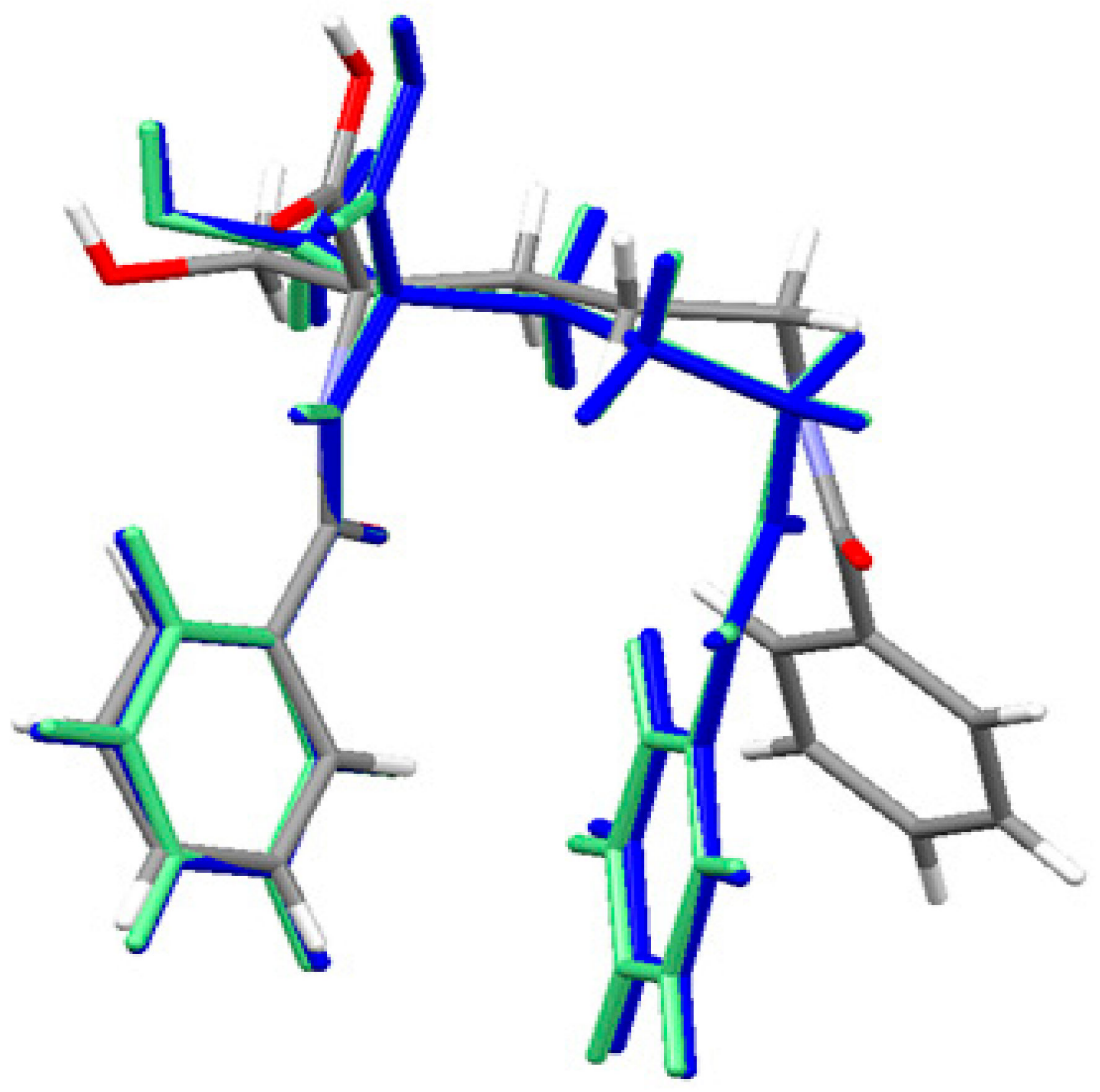
Superposition of the X-ray structure of ornithine derivative **1** and the M06 optimized ornithine derivative **1** in gas-phase (blue) and solvated state (light green). Experimental X-ray structure is colored by atoms.

The geometry of the neutral ornithine derivative molecule **1** in the singlet ground spin state has been completely optimized (starting from its X-ray structure) in both gas-phase and water solution using the Gaussian 09 program [83] at the density functional theoretical [84,85,86] level using the M06/6–311++G(d,p) [87,88] treatment where solvent effects were approximated within the Conductor-like Polarizable Continuum Model (CPCM) [89,90,91]. The molecular superposition of the X-ray structure of ornithine derivative **1** and corresponding gas phase and condensed phase (water) structures shows that in the absence of important intramolecular interactions between two aromatic moieties connected by a flexible *C4* aliphatic chain enabling almost a free rotation the position of two aromatic end groups is different in the solid-state and the gas phase. An equilibrium geometry of ornithine derivative **1** in the isolated molecule is a result of optimal intermolecular contacts between aromatic rings resulting in a more sandwich structure. This conformation is also present in the water solution. The effect of the environment (water) was accessed using the CPCM method. Water has a slight effect on the geometry of ornithine derivative **1** moiety and it is close to this one computed for the isolated state. The results are shown in Figure 3 and Table S2 as well. They show a high level of similarity between the vacuum and solvated structures. The major difference with the crystal structure is related to the conformation of the terminal benzoyl C15-C20 ring.

### 2.4. Supramolecular Commentary

In supramolecular studies, it is important to take a holistic view of all levels of supramolecular architecture. In **1**, molecules are associated tail-to-tail (head-to-head) in a zigzag fashion *via* N-H^…^O hydrogen bonds (Figure S1). Additional O-H^…^O and C-H^…^O bonds complete organization of the 3D crystal network. Detailed geometrical parameters are collected in Table 2.

**Figure 4:**
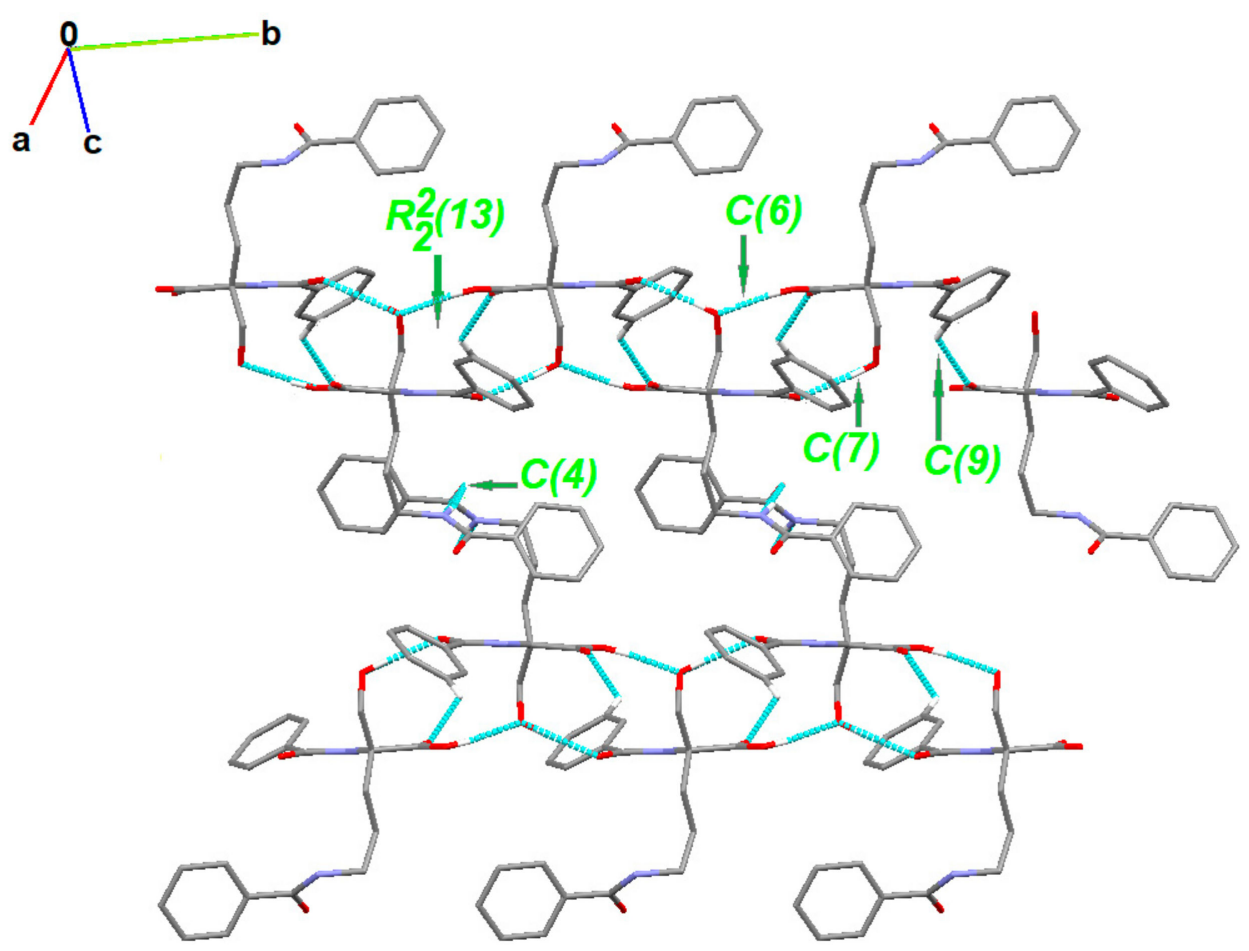
Partial crystal packing of **1** showing supramolecular synthons. H-atoms not involved in the inetractiosn were omitted for clarity.

The partial crystal packing is shown in Figure S2. At the first level of the Graph Set Theory [92,93,94], basic synthons are as follows: *C*(7), namely motif *a*, *via* O2-H21^…^O1, *C*(6); motif *b*, *via* O4-H41^…^O2, *C(4)*; motif *c*, through N2-H20^…^O5 as well as C(9) by C3-H3^…^O3 interactions. At the second level, the pairwise combination of those motifs gives longer chains: *C*^2^_2_(9) (*ab*), *C*^2^_2_(18) (*bc*) and *C^2^_2_(19)*(*ac*). Motifs *a* and *b* jointly form a ring pattern with the *R*^2^_2_(13) graph descriptor. Consequently, a chain of fused rings along the *b*-axis is generated. Additionally, C3-H3^…^O3 interaction is involved in the formation of long supramolecular chains *C^2^_2_ (13)* and *C^2^_2_(15)* (Figure 4 and Figure S3).

**Figure 5:**
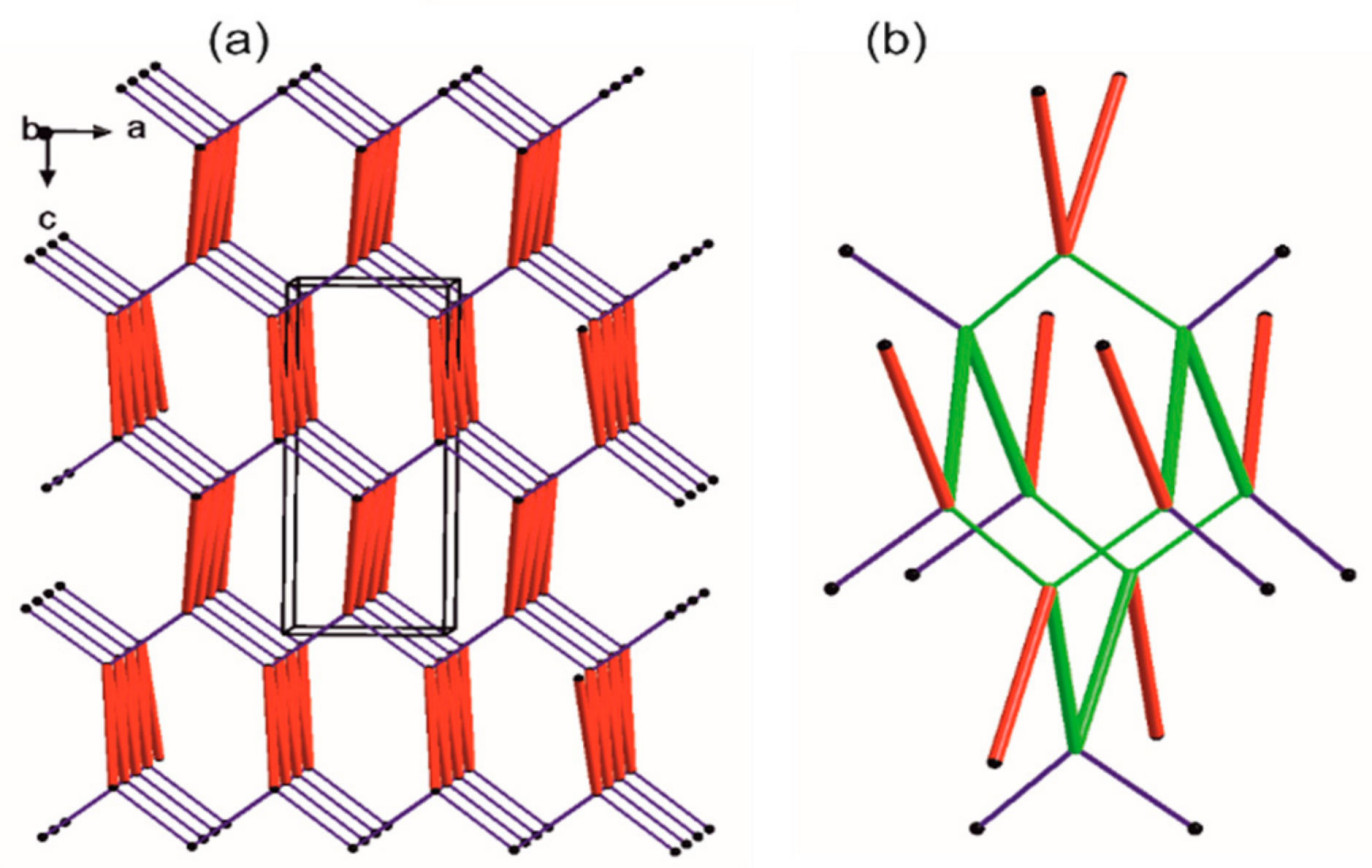
Simplified representation of the crystal packing in **1**. The black balls represent the gravity center of the molecule, red thick vertices are in accordance to a ribbon-like large synthon (motifs *a* and *b* jointly) while blue thin lines correspond to N-H^…^O hydrogen bonds of motif *c*. (**a**) Simplified drawing showing the diamondoid 3-D packing; (**b**) A distorted diamondoid fragment of the network (in green).

Following their H-bonds energies, those entities (Figure 5) are large synthons, *Long-Range Synthon Aufbau Module* (LSAM). The latter contain more than one type of intermolecular interactions combined with representative information on symmetry, long range order and overall topology of the crystal. They represent a more comprehensive approach than the basic synthon concept [95]. For motifs *a* and *b,* H-bond energies are of 5.34 and 7.16 kcal/mol, respectively. These energies are higher than that of the ribbon type, almost hexagonally packed motif c (2.75 kcal/mol). Both *a* and *b* motifs link molecules generated by the same symmetry operator [*-x, -1/2+y, 3/2-z*] and can be reduced to one connection in the simplified topological representation of the network. Moreover, each molecule can be reduced to a node that is graphically represented by a sphere of an arbitrary radius indicating the gravity center of a molecule (Figure 5). 

Each node has four connections running along the strongest hydrogen bonds, i.e., two times motif c (blue lines) and two times joint motifs *a* and *b* (red lines). In effect, a diamondoid uninodal 4-connected topology is observed with {66} or (6, 4) point group symbols according to Wells or Schläfli notations [96], respectively. Furthermore, we compile our research with information derived from the CSD (Table S1).

One of the interesting issues is related to aromatic interactions involving π-system, which are vital for the ligand-target binding [97]. Those weak π^…^π stacking interactions are observed in **1**, EXOFAY [98], GOTFAY [99] and TEFMIA [100]. The first two structures have terminal phenyl substituents and form stacks in the crystal. The latter two structures incorporate aromatic solvents and are involved in π^…^π interactions. Additionally, the C-H^…^π contacts stabilize crystal packing of EXOFAY [98]. Rare, non-covalent C=O^….^π interactions exist in **1**, while N-O^…^π are in GOTFAY [99] and TEFMIA [100]. Relevant data are summarized in Tables S3 and S4. Similar π^…^stacking interactions drive ornithine association in two macromolecular complexes: the ornithine decarboxylase complex with D-ornithine (RCSB PDB reference number: 1NJJ.pdb) [101] and ornithine decarboxylase with *α*-difluoromethylornithine (2TOD.pdb) [102]. The former is shown in Figure S4. A library of synthon patterns in crystals of ornithine derivatives is presented in Table S5. The most popular synthons are *C^2^_2_(10)* and *C^2^_2_(11),* built by O (N, C)-H^…^O and N-H^…^N interactions (Figure S5 and Table S6). Here, we would like to emphasize that the interplay of weak non-covalent interactions often leads to the increase in their strength through the cooperativity and related associated effects [103,104]. They exert an impact on either stability of the supramolecular architecture or the mechanisms of its formation and rearrangements. In this respect, the decent hierarchy of synthons is crucial for the proper conformation and folding pathways assignments. Comprehensive synthon libraries may be particularly useful for searching the binding sites in biological macromolecules and their complexes further fostering new active sites discoveries.

### 2.5. Hirshfeld Surface Analysis

**Figure 6:**
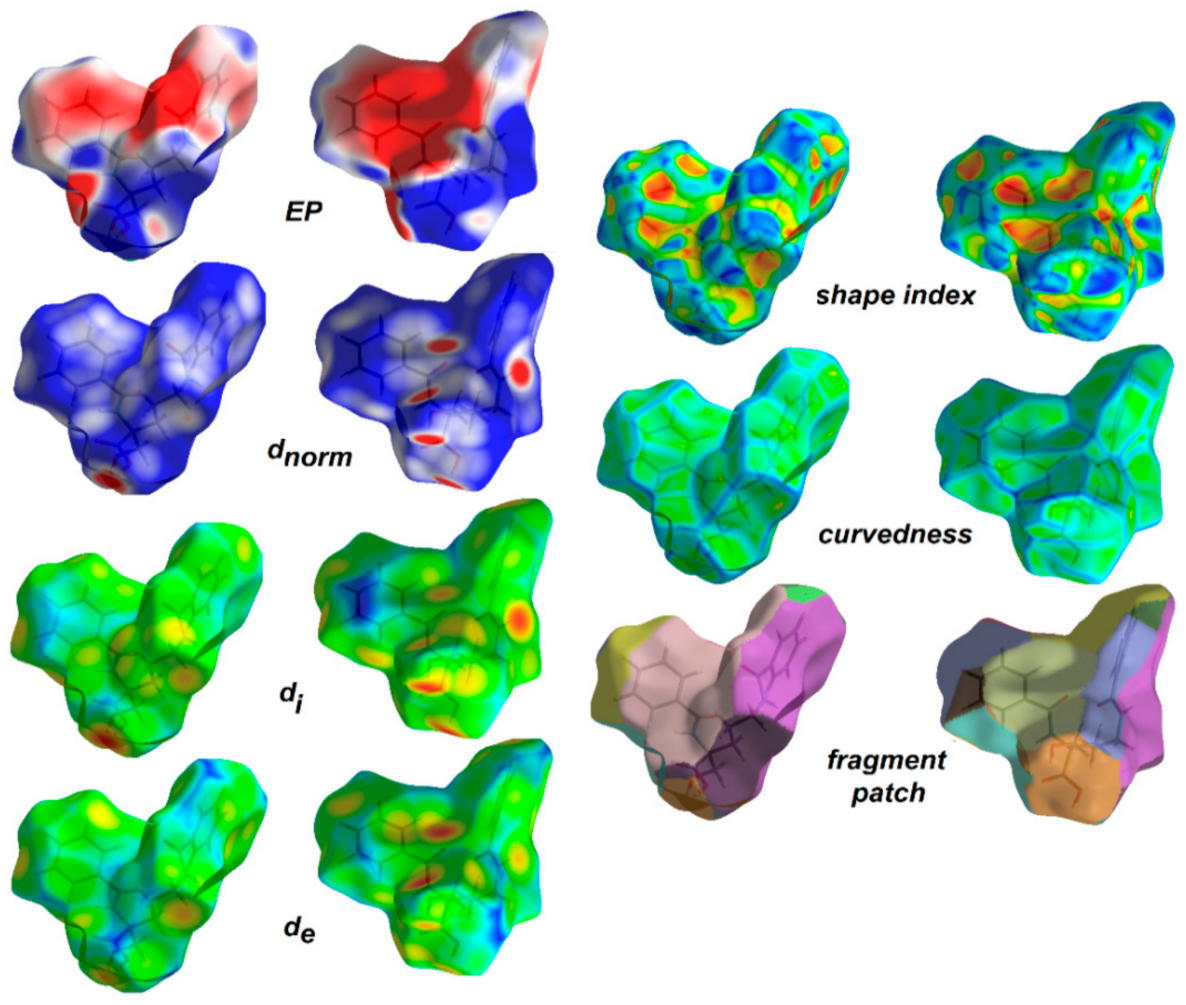
View of the 3D Hirshfeld surface of the title compound **1** mapped with *d_norm_*, showing basic synthons: *C*(**6**) [on the left], *C*(**4**) [in the middle] and *C*(**9**) [on the right], created by O-H^…^O, N-H^…^O and C-H^…^O hydrogen bonds, respectively. The surfaces are shown as transparent to allow visualization of the orientation and conformation of the functional groups (on the left side: front; on the right: back).

Hirshfeld surface analysis [105,106] was employed to understand the nature of interactions and visualize the packing pattern in **1** and the related crystal structure of ornithine derivatives. The surface maps facilitate identification of interactions, while fingerprint plots (FP) outline the distances among atoms involved in those interactions. In other words, 3D HS maps enable quantitative analysis, while 2D FP – qualitative analysis. In the *d_norm_* map (0.60 to 0.90 Å), the vivid bright-red spots in the HS maps are due to short normalized O^…^H distances as corresponding to O-H^…^O interactions. The medium red areas correlate with N-H^…^O, while light red regions are related to the weak C-H^…^O interactions. The main ornithine-based synthons are clearly visible. White spots represent subtle H^…^H contacts, playing a role of “supramolecular glue” as related to the crystal stability. HS mapped over *d_norm_*, *shape-index*, *curvedness* and *fragment patches* properties for **1** are shown in Figure 6 and are in agreement with the crystal packing topology.

**Figure 7:**
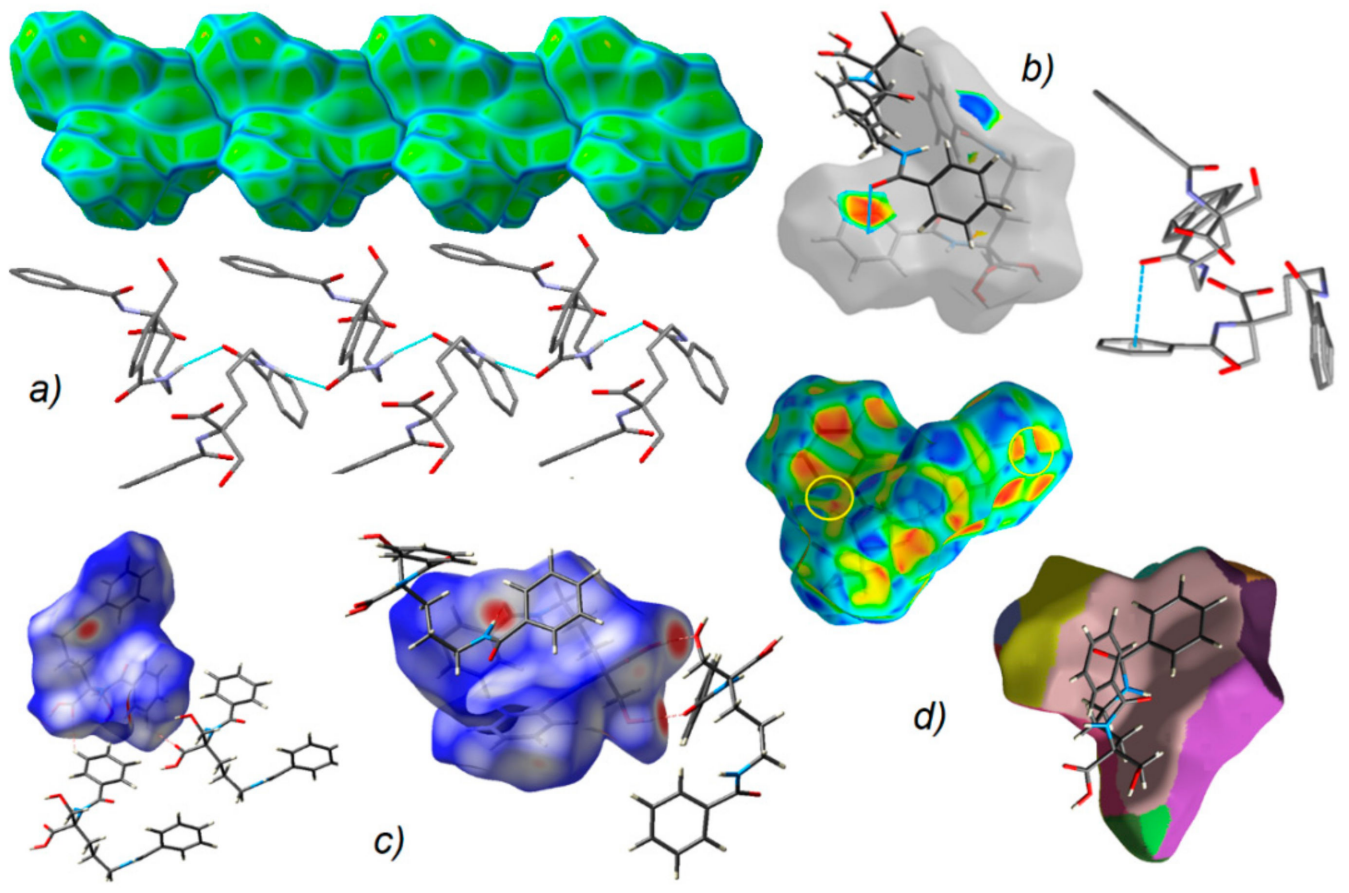
(**a**) Supramolecular chain showed in the context of HS packing; (**b**) view of Hirshfeld surface of **1** mapped over the *shape-index* property highlighting C=O^…^π by blue line and π^…^π contacts showed by yellow circles; (**c**) O-H^…^O, N-H^…^O and C-H^…^O H-bonds on the HS over *d_norm_*; (**d**) neighborhood in the relation of fragment patch property.

**Figure 8:**
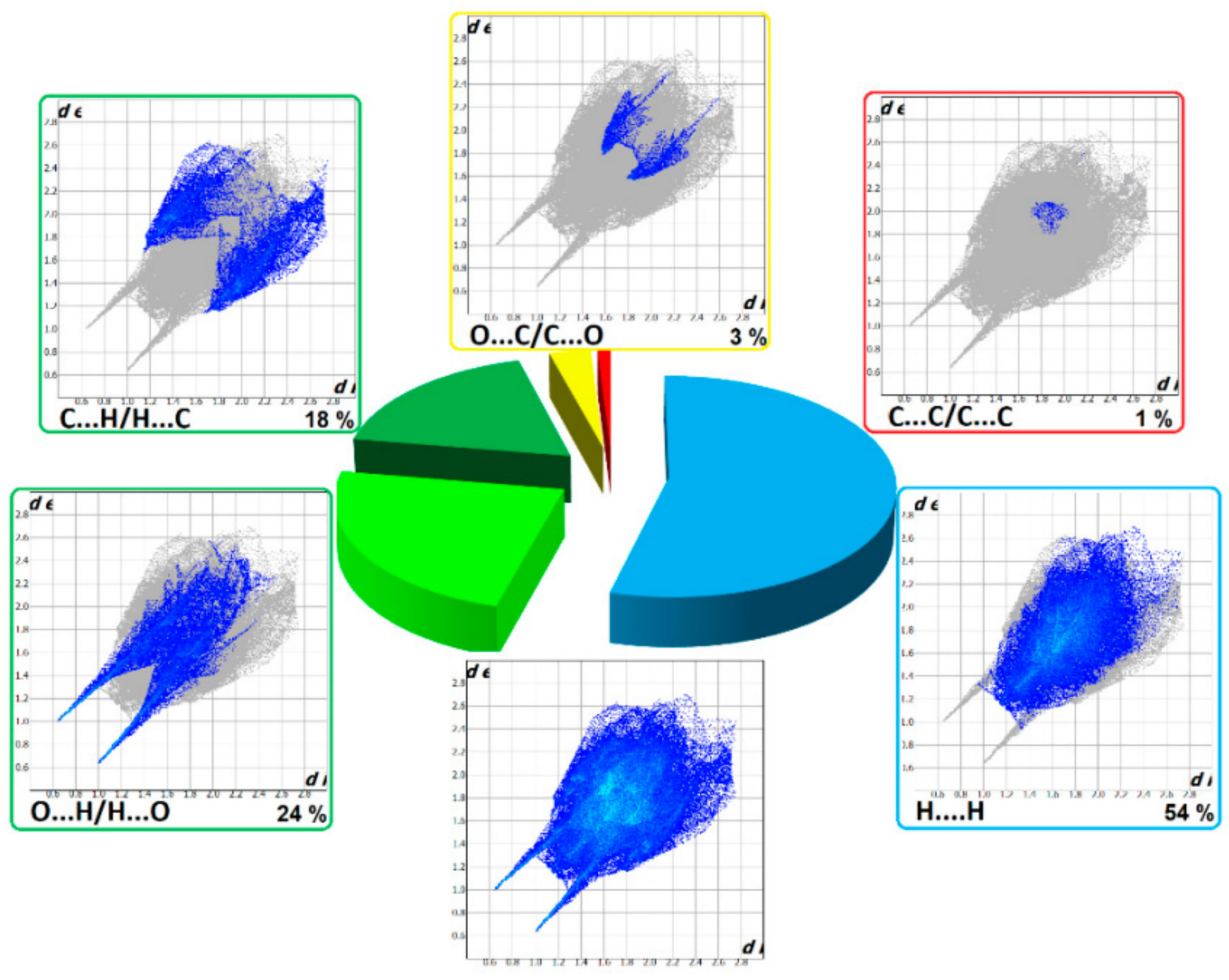
Full two-dimensional (2D) fingerprint plots of **1**, and those delineated into the major non-covalent interactions: H^...^H, O^…^H/H^…^O, C^…^H/H^…^C and O^…^C/C^…^O.

The HS cloning (Figure 7) clearly shows supramolecular chains as identified in crystals of **1**. The analysis of FP plots (*d_e_*-*d_i_* diagrams) indicates the importance of three main categories of interactions. In particular, HS coverage for **1** is 54 % for H^…^H, 23 % for O^…^H/H^…^O and 18 % for C^…^H/H^…^C contacts. The less visible C^…^O/O^…^C contacts contribute to the overall crystal packing at the ~ 2.5 % level. Additionally, the N^…^H/H^…^N, O^…^O, N^…^C/C^…^N and C^…^C contacts are observed (~ 1%). The full and decomposed FP of **1** are drawn in Figure 8. Comparative analysis of all ornithine derivatives revealed that in all structures H^…^H and O^…^H/H^…^O interactions dominate over the others. The highest contribution of H^…^H contacts is in **1** and EXOFAY [98], while the lowest in GOTFAY [99] and TEFMIA [100]. The opposite situation is observed for O^…^H/H^…^O hydrogen bonds, which are followed by halogen bonds. Namely, F^…^H/H^…^F in YIGMAE [107], BEZQOO [108] and IHEPES [60], Br^…^H/H^…^Br in ORNBDL10 [109] and BAPKUB [110], Cl^…^H/H^…^Cl in IHEPES [60], BAPKOV [110] and EVIJAU [111]. The C^…^H/H^…^C and C^…^C interactions have a significant share in aromatic ornithine derivatives, **1** and EXOFAY [98] only.

They reciprocate π^…^π contacts, at the level ~ 1.5 %. and appear as adjacent blue/red triangles in the *shape-index* map of **1**, Figure 8.

**Figure 9:**
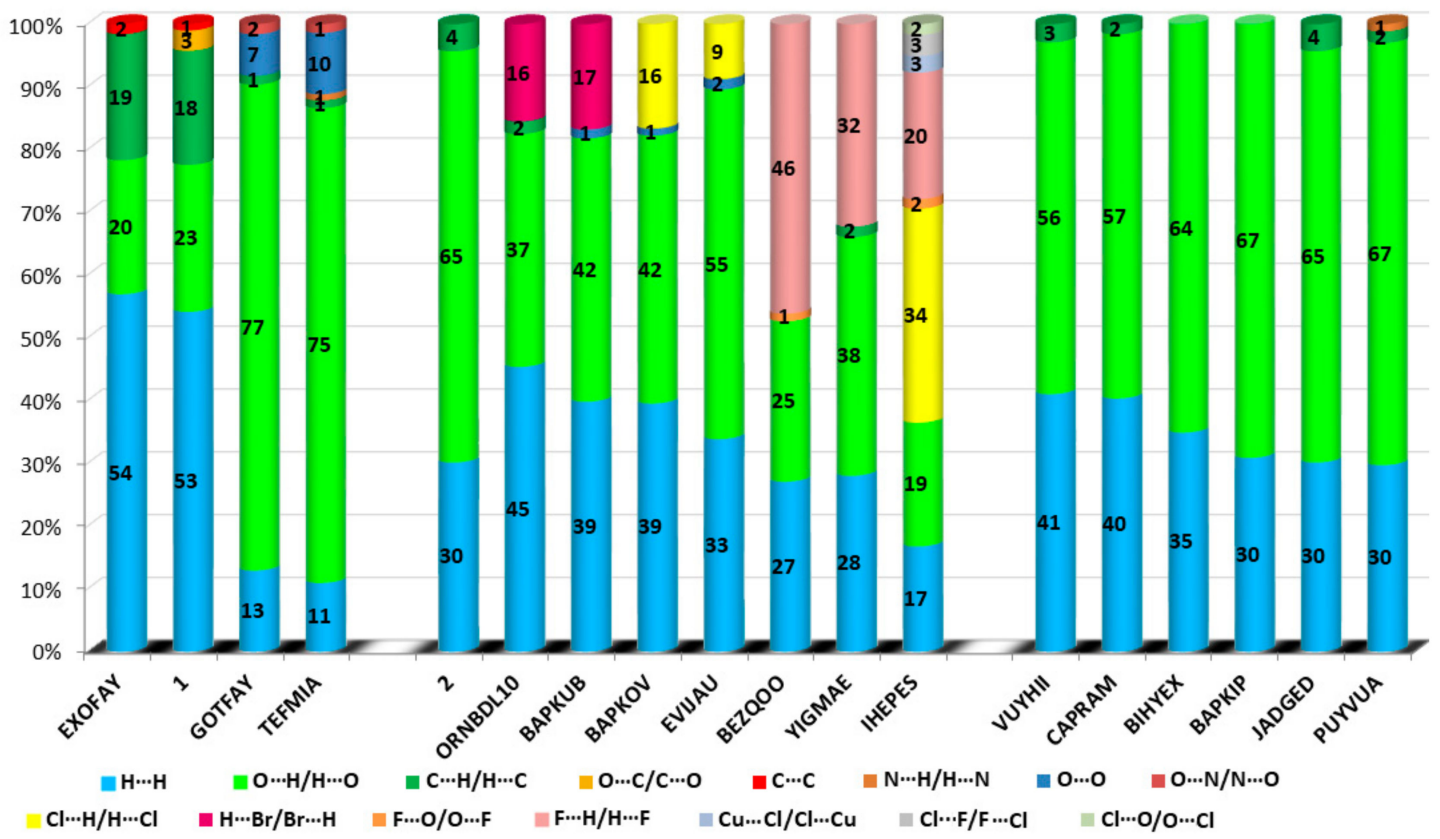
Relative contributions of the different interactions to the Hirshfeld surface for all ornithine derivatives.

The C^…^O interactions were detected in **1** only while the O^…^O interactions are important for GOTFAY [99] and TEFMIA [100]. A summary of essential contacts and their relative contributions is shown in Figure 9 and summarized in Table S7. Finger plots are visualized in Figure S6. HS parameters are shown in Table S8.

**Figure 10:**
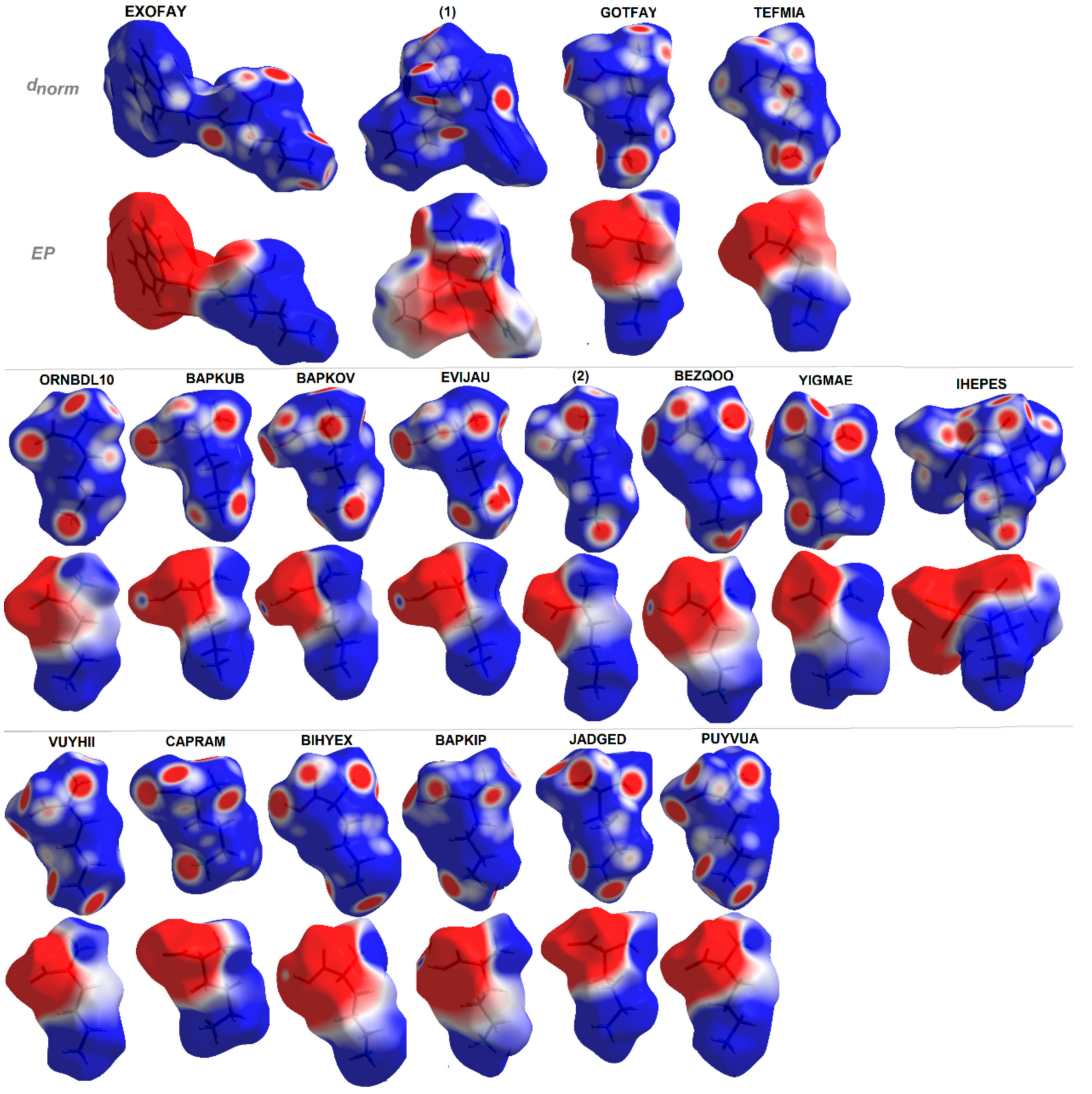
Molecular electrostatic potentials (EPs) of ornithine-based crystals plotted on Hirshfeld surfaces. For interpretation of the references to color in this figure legend, the reader is referred to the web version of this article.

Additionally, EP maps for all discussed compounds were calculated and are presented in Figure 10. They clearly show all H-bond donor (blue regions) and acceptor (red surfaces) sites and indicate the dipolar character of the investigated structures with positive and negative areas clearly resolved over distinguished fragments of molecules [112]. The EP in combination with structural data lead to the hierarchy of H-bonding synthons for planning further advanced studies.

### 2.6. In Silico ADME Predictions

From a drug discovery point of view, *in silico* prediction of molecular physicochemical parameters, bioavailability and pharmacokinetic become more important for the investigation of efficient potential drug molecules [113,114]. The theoretical studies have a fundamental role in providing reliable data in a quick and easy manner. Recently, many free web platforms have been developed for faster screening, to reduce the time and cost of researches of drug candidates (no animal testing) [115,116]. SwissADME is a new comprehensive tool run by the Swiss Institute of Bioinformatics (SIB) enabling estimation of ADME (absorption, distribution, metabolism and excretion) parameters of drug candidates. The ADME properties [117] determining either the access of the potential drug in the target or its elimination by the organism, are necessary at the initial stage of the drug discovery process. These parameters can be verified by *in silico* studies based on calculated physicochemical standards. The latter emphasize lipophilicity, water-solubility, molecule size, polarity, saturation or flexibility. Lipinski et al. [118,119], as the first, presented a drug-likeness related to the relationship between pharmacokinetics and physico-chemical features. In other words, drug-likeness is a complex balance of molecular properties and structural features determining if the studied molecule is like the known drugs. The “Rules of 5” (Ro5), known as Pfizer’s or Lipinski’s rules, were described in 1997 by Christopher Lipinski. It evaluates the drug-likeness, which depends on the following factors: molar mass (which should be ≤ 500 g mol^-1^), log P (≤ 5), number of hydrogen bond acceptors (≤ 10; accounted in the function of N or O atoms in the molecule), and number of hydrogen bond donors (≤ 5, accounted in the function of NH or OH groups in the molecule). Besides, Ghose [120,121], Veber [122], Egan [123,124] and Muegge [125] rules can be also applied to predict drug-likeness. Moreover, SwissADME includes ‘BOILED-egg evaluation’ [18,19,20,21] enabling insight into the human gastrointestinal absorption (HIA) and blood-brain-barrier (BBB) permeability.

In this work, an *in silico* study of novel ornithine derivative **1** and related compounds were performed to predict drug-likeness, physicochemical properties, lipophilicity, solubility, pharmacokinetics and medicinal chemistry using the SwissADME web tool. The results are summarized in Table 3, Table 4 and Table 5. Investigated molecules possess several favorable ADME properties. A novel compound **1** is a good drug candidate. Its pharmacokinetic parameters are comparable to DFMO. In addition, it is not a P-gp substrate (p-glycoprotein). Thus, it could be a potential anticancer agent. And moreover, all compounds are in agreement with the desirable Lipinski rule principles, indicating drug-likeness. The majority of compounds have bioavailability score of 0.55 or 0.56, which means good pharmacokinetic properties. Besides, they have good absorption (ABS), to nearly 80 %. The %ABS, a very functional physiochemical variable, defines drug transport properties. It was calculated according to the equation %ABS = 109 – (0.345 × TPSA) [126,127]. The compounds with TPSA values below 140 Ǻ^2^ characterize a significant permeability in the cellular plasma membrane. Some of the molecules show high gastrointestinal (GI) absorption. An acceptable range of molar refractivity (40–130) was found in all cases. Compound **1** and some relative compounds (EXOFAY, BEZQOO, IHEPES, ref. codes are described in the Section 3.3) are rather a skin permeable, revealing relatively good permeability values (log *Kp*; with *Kp* in cm/s; –9.7 < log *Kp* < –3.5) [128]. In **1**, the log *P* (octanol–water partition coefficient) = 2.30 indicates rather a reasonable absorbancy, while log *S* (ESOL criteria; [129]) = −2.78 defines good solubility in the body. Generally, ornithine-based compounds have very good water solubility. Nevertheless, some differences among the methods are observed (Table S9). The ESOL and Ali methods are based on complete molecular topology, while SILICOS-IT involves a fragment-based approach in Log *S* calculation.

**Figure 11:**
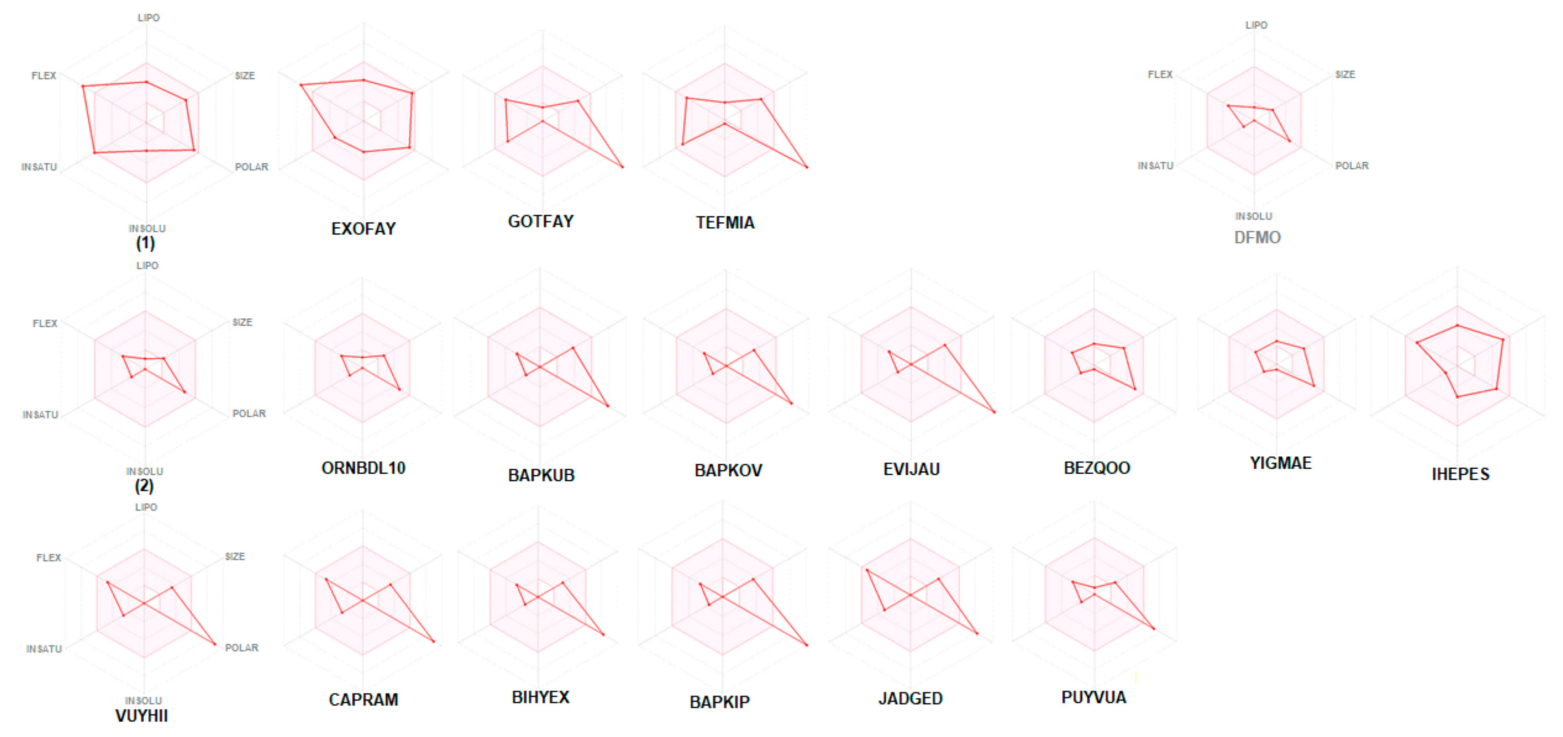
Bioavailability radar (pink area exhibits optimal range of particular property) for studied compounds [LIPO = lipophilicity as XLOGP3; SIZE = size as molecular weight; POLAR = polarity as TPSA (topological polar surface area); INSOLU = insolubility in water by log *S* scale; INSATU = insaturation *as per* fraction of carbons in the sp^3^ hybridization and FLEX = flexibility *as per* rotatable bonds].

The bioavailability radars of studied compounds can be analyzed intuitively (Figure 11). These for SwissADME unique snapshots are the drug-likeness graphs, presented in the form of a hexagon with each of the vertices representing a parameter that defines a bioavailable drug. The pink regions represent the optimum range of the following six properties: lipophilicity (XLOGP3 between −0.7 and +5.0), size (MW between 150 and 500 g/mol), polarity (TPSA between 20 and 130 Å^2^), solubility (log *S* not higher than 6), saturation (fraction of carbons in the sp^3^ hybridization not less than 0.25) and flexibility (no more than nine rotatable bonds). Drug-likeness properties are represented by the red distorted hexagon within the pink shade. It was found that compound **1** is slightly outside the pink area on one side, due to the inconformity of flexibility. In some other cases, off-shoot of one of the vertices towards polarity is observed.

**Figure 12:**
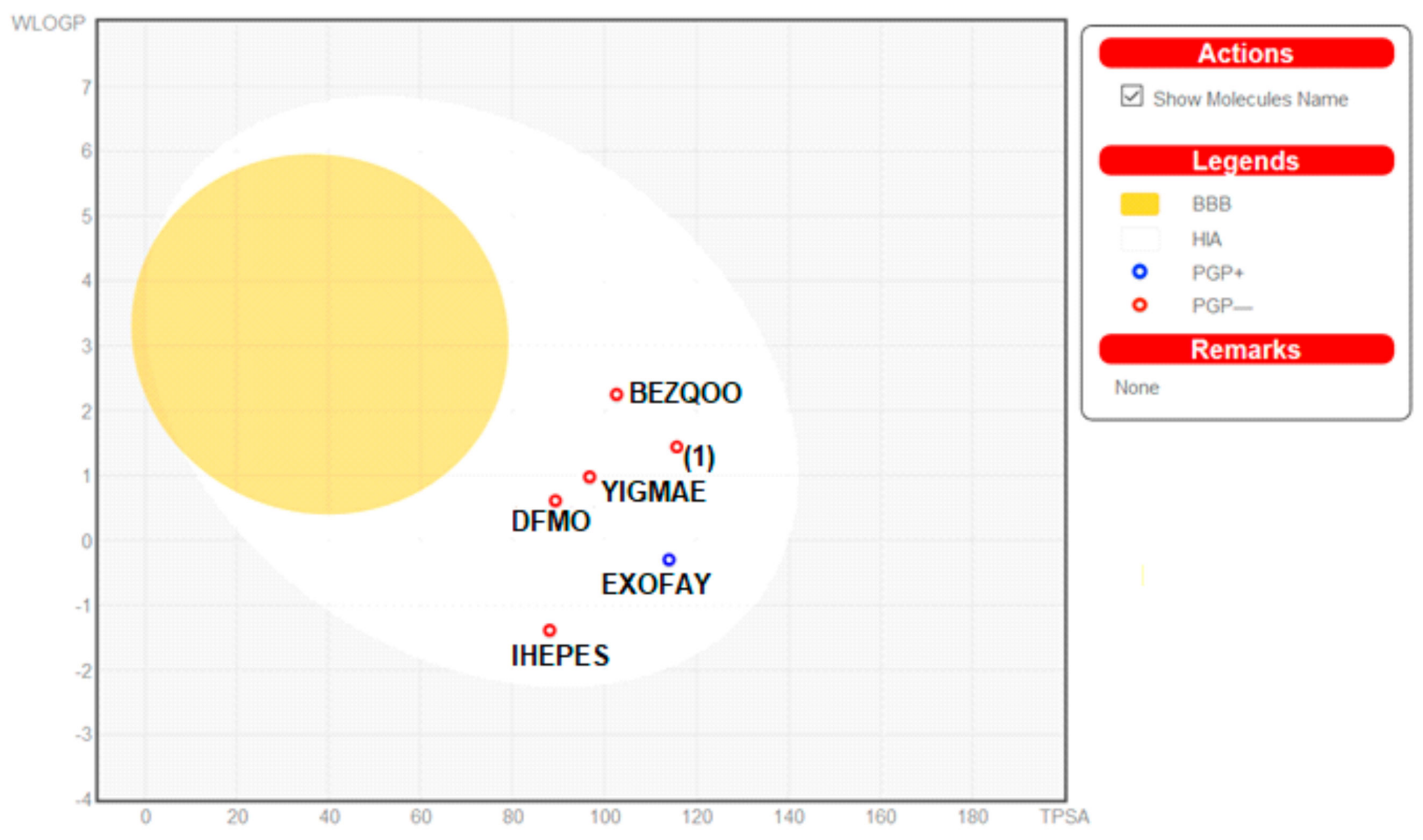
Predicted BOILED-Egg diagram of the selected compounds, from SwissADME web tool.

Moreover, the ADME properties *in vivo* were predicted with the graphical classification model, the Egan BOILED-Egg (Brain Or IntestinaL EstimateD) permeation predictive model diagram, including passive human gastrointestinal absorption (HIA), blood–brain barrier (BBB) permeation. All compounds have no BBB permeability. Nevertheless, **1** as well as BEZQOO, EXOFAY, IHEPES and YIGMAE exert high HIA (in the white region), see Figure 12.

In addition, toxicity risk was estimated using web servers pkCSM [130] and ProTox II [22,23]. Calculations revealed that **1** represents category V with LD50: 5000 mg/kg (Tables S10–S12). 

To sum up, obtained good parameters suggest that the novel ornithine-based compound **1** has potential as a drug.

The findings can be useful platform for design and synthesis either novel ornithine derivatives or other compounds based on the ornithine-derived synthons.

## 3. Materials and Methods

### 3.1. Crystallization, X-ray Structure Determinations

(*S*)-Ornithine and solvents used in this work are commercially available. They were purchased from Sigma Aldrich (Poznan, Poland) and used without further purification. We found very efficient fractional crystallization of racemic *N,N*-dibenzoylhydroxymethylornithine as its diastereomeric salts formed with (–)-cinchonidine. After releasing from its salt a single crystal of (+)-*N,N*-dibenzoyl-hydroxymethylornithine suitable for SC-XRD experiment has been grown by slow evaporation of its solution in ethyl acetate. An absolute configuration for this enantiomer has been assigned by X-ray diffraction as (*S*). Data of were collected using a Siemens P3 diffractometer with fine-focus sealed tube using graphite monochromated Mo-*Kα* radiation (λ = 0.71073 Å) as a source of radiation. The structure was solved with the SHELXS-2014 structure solution program and refined in the SHELXL-2014 [131] by the full-matrix least-squares on *F*^2^. All non-hydrogen atoms were initially located on *E* maps and refined anisotropically. Hydrogen atoms were found in difference Fourier map. The final refinement converged at *R* = 0.045, calculated for 3838 reflections [*F_0_* > 4 (*F_0_*)] [131]. The crystal data, data collection and structure refinement details for the compound **1** can be found in Table 1. Hydrogen atoms were located from a difference map and refined isotropically. For the final refinement, C- and N- and O-bound H atoms were positioned geometrically, with C-H = 0.97 (methylene CH_2_) and 0.93 (CH aromatic), with N-H = 0.86 (1) and O-H = 0.82 (3) Å, and refined using a riding model, with *U*_iso_(H) = 1.5*U*_eq_(O), 1.2*U*_eq_(C, N). One of the phenyl ring is disordered over two sites [occupancies: 0.6 and 0.4]. The absolute structure was established with the use of Cu *Kα* radiation. The *R* value of the refined model is 3.2 %. Additional calculations and figures were carried out by PLATON program [132], ORTEP-3 for Windows [133] and Mercury [134]. Additionally, to better comparative analysis, a redetermination of the crystal structure of the ornithine hydrochloride (new version of ORNHCL, see Section 3.3) was carried out using Rigaku (Breslau, Poland) diffactometer and is denoted **2** [135]. Crystal data of both compounds can be obtained from the CCDC free of charge *via* http://www.ccdc.cam.uk/conts/retrieving.html (or from the Cambridge Crystallographic Data Centre 12, Union Road, Cambridge CB2 1EZ, UK; fax: +44 1223 336033). CCDC numbers for **1** and **2** are 1981249 and 1981318, respectively.

### 3.2. Computations

#### 3.2.1. DFT Calculations

*In silico* calculations were performed with the GAUSSIAN09 [83] software using the DFT level of theory [136], were used for geometry optimization. GaussView5.0 [137] software was used to prepare and analyze the computed geometries. The geometry of neutral ornithine derivative **1** molecule in singlet ground spin state obtained from X-ray analysis of single crystals has been completely optimized using the Gaussian 09 program [83]. For calculations of stable conformers in both gas-phase and solvated state the density functional theory [84,85,86] at the M06 [87] level of theory and the polarized triple-ζ 6–311++G(d,p) basis set [88] were used. The Conductor-like Polarizable Continuum Model (CPCM) [89,90,91] was exploited for the evaluation of the effect of hydration on the structure of ornithine derivative **1** conformers studied. The gas-phase and solvated state molecular structures obtained from theoretical calculations were compared and discussed with the structure of those compounds in the crystalline state.

#### 3.2.2. H-bond Energies

The O−H···O and N−H···O bonds energies were estimated using the method proposed by Lippincott and Schroeder [138,139] with the Lippincott Schroeder H-bond (LSHB) program courtesy of the authors [140]. To correct for X-ray proton positioning errors, all O−H and N−H bonds were renormalized by setting the distances to the reference value of 0.993 and 1.015 Å, respectively. We are aware that the estimated standard deviation for LSHB calculations can exceed 0.1–0.2 kcal/mol. However, we use the more precise values in accord with the original works [138,139].

#### 3.2.3. Hirshfeld Surface Analysis

Qualitative and quantitative analysis of intermolecular interactions were performed *via* Hirshfeld surface (HS) study [105,106,141] using the CrystalExplorer 17.5 [142,143,144,145] program, based on the X-ray data. The bond lengths of the H-atoms were normalized to standard neutron diffraction values. The color scheme of the surface areas are related to the normalized contact distance *d_norm_* defined in terms of the van der Waals (vdW) radii of the atoms and *d_e_*, *d_i_*, the distances from the Hirshfeld surface to the nearest atom outside (external) and inside (internal) the surface, respectively. Red regions show the distances shorter than the sum of vdW radii, white - distances equal to the sum of vdW radii, while blue - distances longer than the sum of vdW radii. The electrostatic potential is mapped on Hirshfeld Surface using wave function STO-3 G basis set at Hartree-Fock theory level over the range of ±0.030 au.

#### 3.2.4. *In Silico* ADME Screening

Physicochemical, pharmacokinetic and drug-likeness properties of the studied compounds were analyzed by SwissADME web-based interface, provided by the Molecular Modeling Group of the Swiss Institute of Bioinformatics (http://www.sib.swiss) [17,18,19,20,21]. 2D structural models of analyzed compounds were drawn in the molecular sketcher into the ChemAxon’s Marvin JS window and transferred to the SMILES (simplified molecular-input line-entry system) format to predict suitable properties. An egg plot is a graph showing WLOGP (*y* axis; threshold value should be ≤5.88), index of lipid solubility for the drug, *versus* TPSA (*x* axis; ≤131.6), the surface area of the molecule occupied by polar groups [18,19,20,21]. An egg yolk (yellow area) characterizes the boundary of properties related to the passing of molecules *via* the blood-brain barrier (BBB), while an egg white (white region) defines the properties of molecules with a high probability of passive absorption by the gastrointestinal (GI) tract. Additionally, the blue points describe molecules predicted as actively effluxed from the central nervous system (CNS) by P-glycoprotein (P-gp; PGP+), while red dots indicate molecules predicted as non-substrate of P-gp (PGP−).

### 3.3. CSD and RCSB PDB Survey

A CSD [146] survey revealed that 24 ornithine-derived crystal structures have been determined so far. The results of search are summarized in the supplementary information (Scheme S1, Table S1). We included various filters: no organometallic and disordered structures, no errors, not polymeric, no relatively high *R1* factor. Namely, *L*-ornithinium sulfate monohydrate, CSD reference code: BIHYEX [147]; *L*-ornithine nitrate, PUYVUA [148]; tris(1-carboxybutane-1,4-diaminium)dinitrate bis(sulfate), BAPKIP [149]; bis(*L*-ornithinium) chloride nitrate sulfate, EVIJAU [111]; bis(1-carboxybutane-1,4-diaminium) dichloride sulfate, BAPKOV [110]; bis(1-carboxybutane-1,4-diaminium) dibromide sulfate, BAPKUB [110]; L-ornithinium hexafluorosilicate monohydrate, BEZQOO [108]; bis(2,5-diammoniopentanoate) hexafluorosilicate, YIGMAE [107]; *L*-ornithine *L*-aspartate hemihydrate, CAPRAM [150] and NAGLYC [151]; *L*-ornithine *D*-aspartate monohydrate, VUYHII [152]; *N*5-aminocarbonyl-*L*-ornithine dihydrate, GOTFAY [99]; TEFMIA [100]; 2,5-diammoniopentanoate 4-carboxy-2-oxobutanoate, JADGED [153]; *D,L*-ornithine hydrobromide ORNBDL01 [154]; ORNBDL10 [109] and QQQAOJ [155]; various measurements of L-ornithine hydrochloride: ORNHCL [155], ORNHCL11 [156]; ORNHCL12 [157] and ORNHCL13 [158]; catena-[(5-ammonio-2-(difluoromethyl)norvalinato)-(*m*-chloro)-dichlorocopper monohydrate IHEPES [60]; *D,L*-ornithinium hexabromo-selenium, ORNSEB [159]; *N-*(9-fluorenyl)methoxycarbonyl*-L-*ornithine hydrochloride diethyl ether clathrate EXOFAY [98].

Above mentioned CSD entries (hits) of ornithine derivatives were found including filters such as no organometallic and disordered structures, no errors, not polymeric, no relatively high *R1* factor. Nevertheless, in this set, eight records lacked 3D coordinates (NAGLYC [151]; ORNSEB [159]; QQQAOJ [155]), without hydrogen atoms or worse quality (repetitions) (ORNHCL [155], ORNBDL01 [154]; ORNHCL11 [156]; ORNHCL13 [158]). The scheme of query used in the CSD search is shown in Scheme S1 in the supporting information. In order to systematically investigate the interactions in a family of ornithine-based compounds, we have divided it into the three groups: those containing halogen atoms [**2**, BAPKOV, BAPKUB, BEZQOO, EVIJAU, IHEPES, ORNBDL10, YIGMAE], aromatic rings [**1**, EXOFAY, GOTFAY, TEFMIA] and others [BAPKIB, BIHYEX, CAPRAM, JADGED, PUYVUA, VUYHII, YIGMAE].

On the other hand, the growing number of bio-complexes with ornithine in RCSB PDB is a rapidly gaining momentum. Interestingly, the survey revealed 475 complexes with ornithine, 23 of which were deposited this year. Nevertheless, we found only two complexes of ornithine decarboxylase with ornithine-derived compounds. Namely, the complex with *D*-ornithine (reference number: 1NJJ.pdb) [100] and with α-difluoroornithine (ref. code: 2TOD; [102]).

## 4. Conclusions

In this work, we briefly outline the medical significance of ornithine and shed light on new developments in the structure-based design, which benefits from the supramolecular chemistry methodology. Ornithine derivatives have potential in the design of novel drugs and cosmeceuticals with improved therapeutic efficacy and are promising multi-targeting ligands. A novel ornithine-based compound, namely *N*-α,*N*-δ)-dibenzoyl-(α)-hydroxymethyl ornithine **1** was successfully synthesized and thoroughly characterized by X-ray diffraction and theoretical DFT approaches. We took a holistic look at its molecular and supramolecular landscape keeping all currently known ornithine derivatives in focus. A comprehensive survey of interactions yielded a library of ornithine-based synthons. This classification takes into consideration the diversity of ornithine structures and may facilitate the structure based design of related substances. The supramolecular frameworks and topologies are well developed and controlled mainly by H^…^O, O^…^H, C^…^H, Cl(Br,F)^…^H, O^…^O, π^…^π, C-H^…^π, N-O^…^π and C=O^…^π interactions. The synthons hierarchy was based on their geometry augmented by relevant EP maps. The presented preferences in building synthons can give valuable information not only in ornithine-based substances. A plethora of interactions make them an interesting object for studying cooperative effects. These studies combined with the docking experiments are in progress and the results will be presented in a separate paper. Additionally, a novel ornithine-based compound **1** was predicted to have good ADME characteristics, notably in terms of the Lipinsky rule and GI absorption, as well as very low toxicity. We hope that the offered reliable theoretical pharmacokinetic basis, molecular and supramolecular profiles of ornithine derivatives may be useful for further studies on new biologically active substances based on the ornithine-derived synthons.

## Figures and Tables

**Table 1:** Crystallographic data and structure refinement parameters for **1**.

Chemical Formula	C_20_ H_22_ N_2_ O_5_
molecular weight (g/mol)	370.40
crystal system, space group	orthorhombic, *P*2_1_2_1_2_1_
T (K)	293
a, b, c (Ǻ)	8.9040(10) 9.9480(10) 22.489(3)
α, β, γ (^o^)	90, 90, 90
*Z*	4
Volume (Ǻ^3^)	1992.0(4)
radiation type	Mo *K α*
µ (mm^−1^)	0.089
diffractometer	Siemens P3
no. of total and independent reflections	3647, 2396
*R* [*F*^2^ > 4 σ(F^2^)], w*R*^2^, S	0.0432, 0.1307, 0.974
no. of reflections and parameters	3174, 301
∆ρ_max_, ∆ρ_min_ (e^−3^)	0.196; −0.152

**Table 2:** Supramolecular interactions present in **1**.

	*D*-H (Å)	H^…^*A* (Å)	*D^…^A* (Å)	*D*-H^…^*A* (^o^)
**1**				
N1-H1^…^O3^*^	0.86	2.17	2.608(2)	111
N2-H20^…^O5^i^	0.86	1.96	2.801(4)	166
O2-H21^…^O1^ii^	0.82	1.84	2.661(2)	174
O4-H41^…^O2^ii^	0.82	1.79	2.600(2)	172
C3-H3^…^O3^iii^	0.93	2.56	3.246(4)	131
C9-H91^…^O1^*^	0.97	2.57	3.093(3)	114
C9-H92^…^O4^*^	0.97	2.57	2.896(3)	100
C11-H112^…^O1^*^	0.97	2.52	3.087(3)	117
C12-H121^…^N1^*^	0.97	2.61	2.992(3)	104

Symmetry codes: (i) ½+x, 3/2-y, 2-z; (ii) 1-x, -1/2+y, 3/2-z; (iii) –x, ½+y, 3/2-z. *Intramolecular H-bonds

**Table 3:** Pharmacological profile, medicinal chemistry principles and lead-likeness properties of **1**.

Physicochemical Properties		Pharmacokinetics		Druglikeness		Medicinal Chemistry	
Molecular weight [g/mol]	370.40	GI absorption	high	Lipinski violation	yes, 0	PAINS	0
Heavy atoms	27	BBB permeant	no	Ghose	yes	leadlikeness	no, 2
Aromatic heavy atoms	12	Pgp substrate	no	Veber	no, 1	synth. accessibility	2.61
Fraction Csp^3^	0.25	CYP1A2 inhibitor	no	Egan	yes		
Rotatable bonds	11	CYP2C19 inhibitor	no	Muegge	yes		
H-bond acceptors	5	CYP2C9 inhibitor	no	Bioavailability score	0.56		
H-bond donors	4	CYP2D6 inhibitor	no				
Molar refractivity	98.96	CYP3A4 inhibitor	no				
TPSA [Ǻ^2^]	115.73	Log K*p* [cm/s]	−7.39				
Consensus log P _0/w_	1.81						

**Table 4:** Selected physicochemical and pharmacokinetic properties of the analyzed compounds.

Comp. Name/CSD Ref. Code	iLog *P*	Molar Refractivity	Log *S*	WLOGP	TPSA [Ǻ^2^]	*In Silico*% Absorption	GI Absorption	P-gp Substrate	Log *Kp*
DFMO	0.53	38.28	S	0.61	89.34	78.17	high	no	−9.48
**1**	2.30	98.96	MS	1.44	115.73	69.08	high	no	−7.39
EXOFAY	0.00	128.51	S	−0.42	112.50	70.19	high	yes	−7.90
GOTFAY	−2.81	88.26	S	−1.19	247.11	23.75	low	no	−10.55
TEFMIA	0.64	88.89	S	0.83	253.10	21.69	low	no	−10.11
**2**	0.00	39.76	S	−6.63	95.41	76.09	low	no	−9.89
ORNBDL10	0.00	42.84	S	−6.63	95.41	76.09	low	no	−10.04
BAPKUB	0.00	55.30	S	−5.55	181.22	46.48	low	yes	−13.23
BAPKOV	0.00	52.22	S	−5.55	181.22	46.48	low	yes	−13.08
EVIJAU	0.00	64.75	S	−5.27	250.10	22.72	low	yes	−13.34
BEZQOO	0.00	51.30	S	2.30	101.81	73.88	high	no	−9.20
YIGMAE	0.00	46.31	S	1.03	95.41	76.09	high	no	−8.75
IHEPES	0.00	58.60	S	−1.66	89.19	78.23	high	no	−7.81
VUYHII	0.42	61.92	S	−8.21	212.54	35.68	low	yes	−15.30
CAPRAM	0.42	61.92	S	−8.21	212.54	35.68	low	yes	−15.30
BIHYEX	−0.97	49.42	S	−2.62	190.45	43.30	low	yes	−13.88
BAPKIP	−1.31	58.90	S	−2.27	250.10	22.72	low	yes	−13.69
JADGED	0.06	61.86	S	−5.46	189.91	43.49	low	yes	−12.80
PUYVUA	0.69	46.44	S	−3.35	164.29	52.32	low	no	−10.50

**iLogP** – liphophilicity; **Log *S*** – water solubility (SILICOS-IT; S-soluble, MS-moderately soluble); **TPSA** – topological polar surface area [Ǻ^2^]; *in silico* % absorption = 109 – (0.345 x TPSA); **GI** – gastro intestinal absorption; **P-gp** – p-glycoprotein inhibitors; **Log *Kp*** – skin permeability coefficient (*Kp* in cm/s).

**Table 5:** Drug-likeness predictions of the analyzed compounds.

Comp. Name/CSD Rref. Rcode	Mol. Weight [g/mol]	Liphophilicity (MLogP)	H-Bond Donors	H-Bond Acceptors	Lipinski Violations	Ghose Viol.	Veber Viol.	Egan Viol.	Muegge Viol.	Bioavailability Score
	Lipinski`s Rule of Five									
DFMO	182.17	−2.32	3	6	yes, 0	no, 1	yes	yes	no, 2	0.55
**1**	370.40	1.61	4	5	yes, 0	yes	no, 1	yes	yes	0.56
EXOFAY	469.01	−1.28	3	5	yes, 0	no, 1	no, 1	yes	yes	0.56
GOTFAY	363.28	−5.79	3	9	yes, 1	no, 1	no, 1	no, 1	no, 2	0.55
TEFMIA	362.27	−4.33	3	9	yes, 1	yes	no, 1	no, 1	no, 2	0.55
**2**	168.62	−7.83	2	2	yes, 0	no, 2	yes	yes	no, 2	0.55
ORNBDL10	213.07	−7.65	2	2	yes, 0	no, 1	yes	yes	no, 1	0.55
BAPKUB	310.14	−9.05	3	6	yes, 0	no, 1	no, 1	no, 1	no, 2	0.11
BAPKOV	265.69	−9.23	3	6	yes, 0	no, 1	no, 1	no, 1	no, 2	0.11
EVIJAU	327.70	−9.01	3	9	yes, 1	no, 1	no, 1	no, 1	no, 2	0.11
BEZQOO	294.27	−7.97	4	9	yes, 0	yes	yes	yes	yes	0.56
YIGMAE	275.24	−7.14	2	8	yes, 0	yes	yes	yes	yes	0.56
IHEPES	433.63	−3.66	3	6	yes, 0	no, 1	yes	yes	yes	0.55
VUYHII	283.28	−13.86	4	7	yes, 0	no, 1	no, 1	no, 1	no, 2	0.55
CAPRAM	283.28	−13.86	4	7	yes, 0	no, 1	no, 1	no, 1	no, 2	0.55
BIHYEX	248.25	−10.39	4	4	yes, 0	no, 1	no, 1	no, 1	no, 2	0.11
BAPKIP	292.24	−9.38	3	9	yes, 1	no, 1	no, 1	no, 1	no, 2	0.11
JADGED	278.26	−9.20	3	7	yes, 0	no, 1	no, 1	no, 1	no, 2	0.11
PUYVUA	195.17	−8.17	2	5	yes, 0	no, 2	no, 1	no, 1	no, 3	0.55

## Supplementary Materials

The following are available online. Additional figures and tables can be found. In particular: Scheme S1. Scheme of query used in CSD database search containing ornithine moiety. Figure S1. Molecules arrangement in the crystal of **1**. Figure S2. The intermolecular hydrogen bonds in the asymmetric unit of **1** [*symmetry codes: (i) ½ + x, 3/2 − y, 2 − z; (ii) 1 − x, −1/2 + y, 3/2 − z; (iii) −x, ½ + y, 3/2 − z*]. Figure S3. Supramolecular synthons in the assembly of **1**. For clarity, hydrogen atoms not involved in the inter-contacts were omitted. Figure S4. Ligand (difluoromethylornithine) interactions in the complex of ornithine decarboxylase with alpha-difluoromethylornithine (2TOD.pdb) (Grishin, 1999). Figure S5. Frequency of occurrence concerning synthons. Figure S6. Full FP plots of all studied crystals. Table S1. Basic information concerning ornithine derivatives retrieved from the CSD. Table S2. Cartesian coordinates (Å) of ornithine derivative **1**, optimized at the M06/6-311++G(d,p) level of theory. **Table S3.** Geometrical parameters (in Ǻ and in ^o^) for the π-stacking moieties involved in the π^…^π interactions for studied compounds. Table S4. Geometrical parameters (in Ǻ and in ^o^) for the other π-stacking moieties for studied compounds. Table S5. Gallery of ornithine-based synthons (selected, below 20-membered). Synthons without participation of ornithine moiety are grey. Table S6. Interplay of interactions (forming synthons) between various functionalities in crystals of ornithine derivatives. Table S7. Percentage contributions of interatomic contacts to the Hirshfeld surface for **1**, **2** and other ornithine derivatives. Table S8 Properties of ornithine moiety in analyzed structures, as derived from HS calculations. Table S9. Water solubility predictions of ornithine-based compounds. Table S10. Pharmacokinetic toxicity parameters obtained *via* pkCSM web server. Table S11. Toxicity parameters for **1**. Table S12. Toxicity model report for **1**.

## Abbreviations

ABS	Absorption
ADME	absorption, distribution, metabolism and excretion
BBB	blood-brain barrier
BOILED-Egg	brain or intestinal estimated
CNS	central nervous system
CPCM	Conductor-like Polarizable Continuum Model
CSD	Cambridge Structure Database
DFMO	difluoroornithine
DFT	Density Functional Theory
*d_e_*	distances from the Hirshfeld surface to the nearest atom outside (external) the surface
*d_i_*	distances from the Hirshfeld surface to the nearest atom inside (internal) the surface
*d_norm_*	normalized contact distance
EP	molecular electrostatic potential
FLEX	flexibility
HIA	human gastrointestinal absorption
HS	Hirshfeld surface analysis
FP	fingerprint plots
GI	gastro intestinal absorption
INSATU	insaturation
INSOLU	insolubility
LIPO	lipophilicilty
LSAM	Long-Range Synthon Aufbau Module
LSHB	the Lippincott and Schroeder H-bond model
P-gp	P-glycoprotein
POLAR	polarity
RCSB PDB	Research Collaboratory fof Structural Bioinformatics
RO5	rule-of-five
SC-XRD	single-crystal X-ray diffraction
SMILES	simplified molecular-input line-entry system
SIB	Swiss Institute of Bioinformatics
TPSA	topological polar surface area
vdW	van derWaals radii
