# Supplementary material for: A Supramolecular Approach to Structure-Based Design with A Focus on Synthons Hierarchy in Ornithine-Derived Ligands: Review, Synthesis, Experimental and in Silico Studies
Source: Molecules. 2020 Mar 3;25(5):1135. doi: 10.3390/molecules25051135 (PMC7179192; doi:10.3390/molecules25051135)
Supplement: Supplementary file 1 [file molecules-25-01135-s001.pdf]

SUPPLEMENTARY MATERIALS

A Supramolecular Approach to Structure-based Design with a Focus on Synthons Hierarchy in Ornithine-derived Ligands: Review, Synthesis, Experimental and *in silico* Studies

Joanna Bojarska [1*], Milan Remko [2], Martin Breza [3], Izabela D. Madura [4], Krzysztof Kaczmarek [5], Janusz Zabrocki [5], and Wojciech M. Wolf [1]

[1] Institute of General and Ecological Chemistry, Lodz University of Technology, Faculty of Chemistry, Żeromskiego 116, Lodz, 90- 924, Poland; wojciech.wolf@p.lodz.pl (W.M.W.); joanna.bojarska@p.lodz.pl (J.B.)

[2] Remedika, Sustekova 1, 85104, Bratislava, Slovakia; milan.remko@gmail.com (M.R.)

[3] Department of Physical Chemistry, Slovak Technical University, Radlinskeho 9, SK-81237 Bratislava, Slovakia; martin.breza@stuba.sk (M.B.)

[4] Warsaw University of Technology, Faculty of Chemistry, Noakowskiego 3, 00-664, Warszawa, Poland; izabela@ch.pw.edu.pl (I.D.M.)

[5] Institute of Organic Chemistry, Lodz University of Technology, Faculty of Chemistry, Żeromskiego 116, Lodz, 90-924, Poland; janusz.zabrocki@p.lodz.pl (J.Z.); krzysztof.kaczmarek@p.lodz.pl (K.K.)

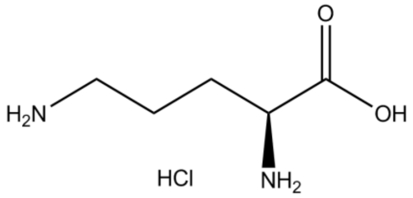

Ornithine

(2)

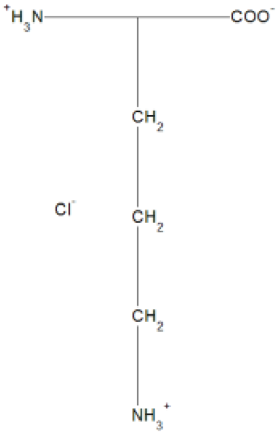

JADGED

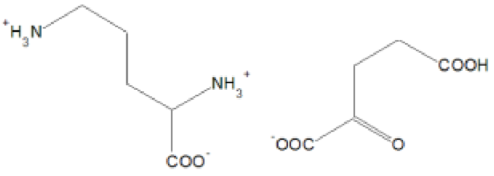

**BAPKIP** 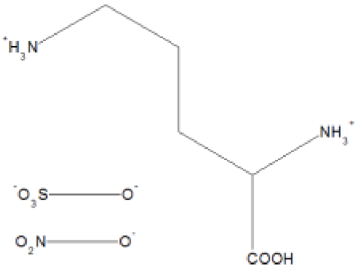	**NAGLYC** 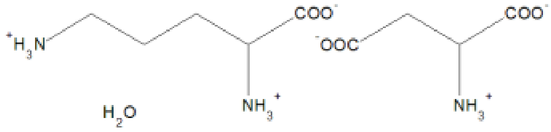
**BAPKOV** 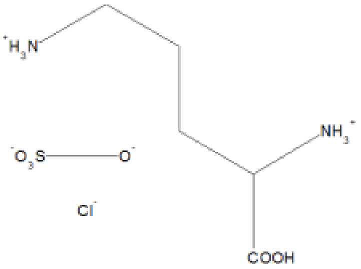	**ORNBDL10** 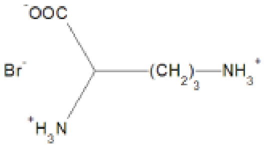
**BAPKUB** 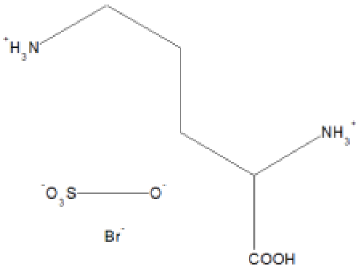	**ORNSEB** 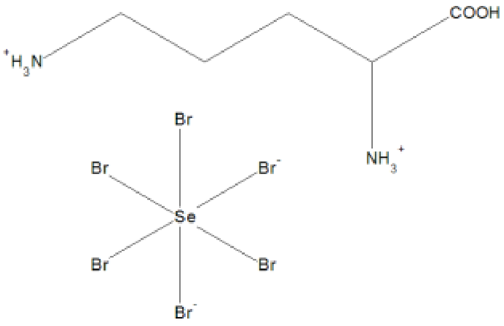
**BEZQOO** 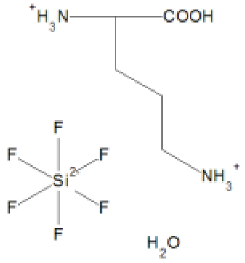	**PUYVUA** 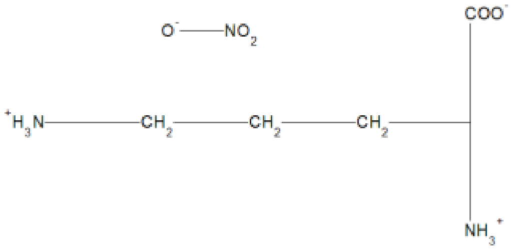
**BIHYEX** 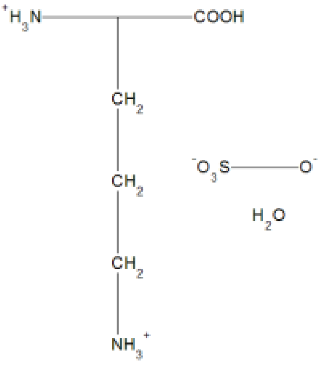	**QQQAOJ** 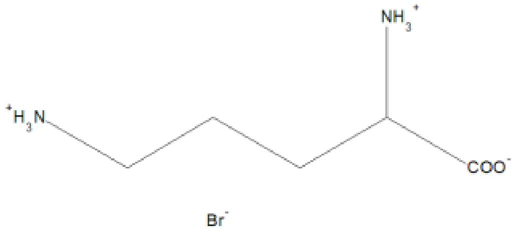
**CAPRAM** 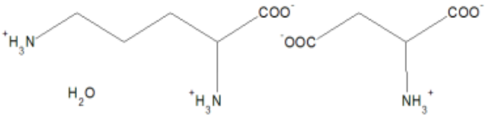	**TEFMIA** 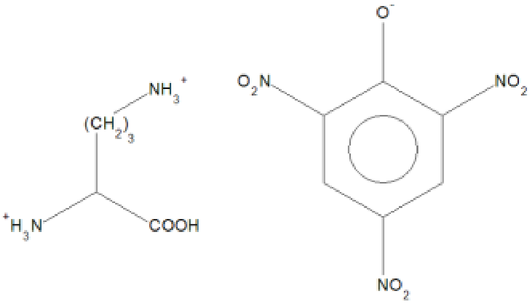

**GOTFAY** 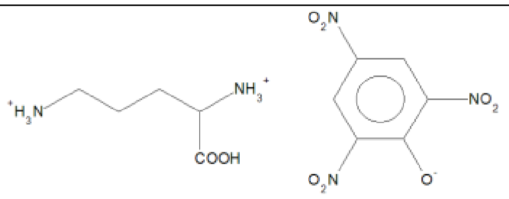	**VUYHII** 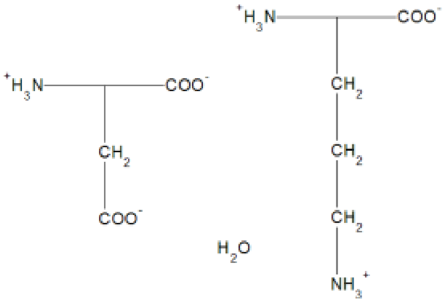
**EVIJAU** 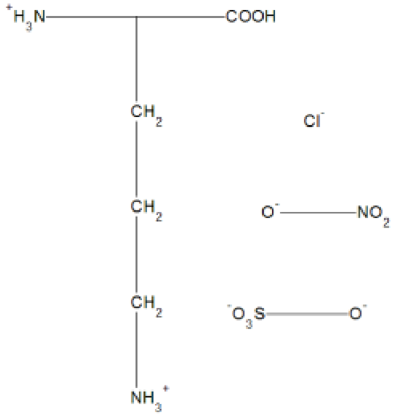	**YIGMAE** 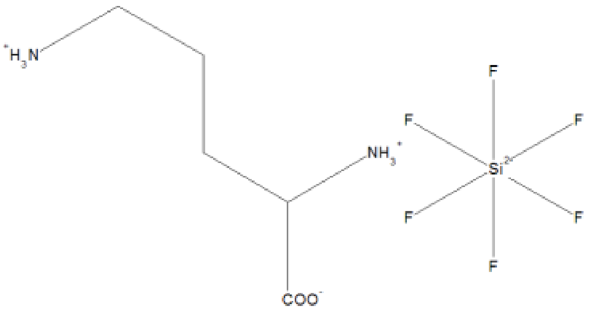
**EXOFAY** 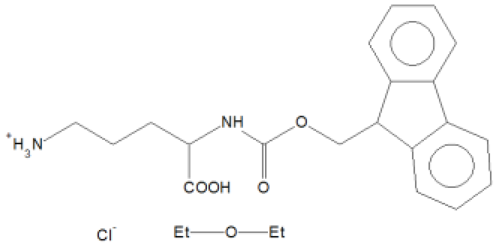	
**IHEPES** 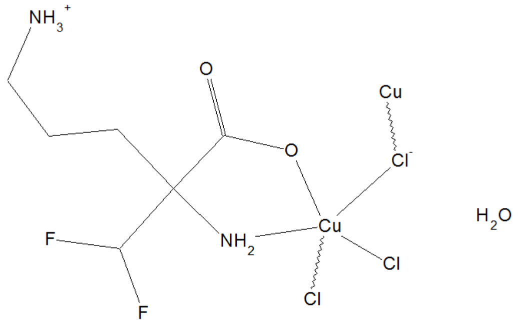	

Scheme S1. Scheme of query used in CSD database search containing ornithine moiety.

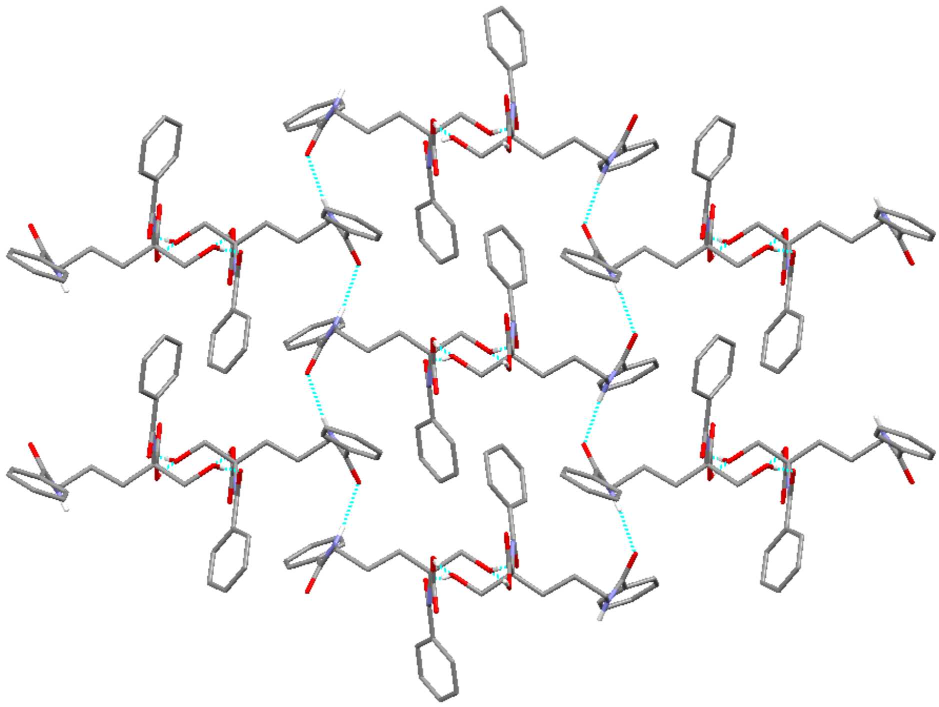

Figure S1. Molecules arrangement in the crystal of (**1**).

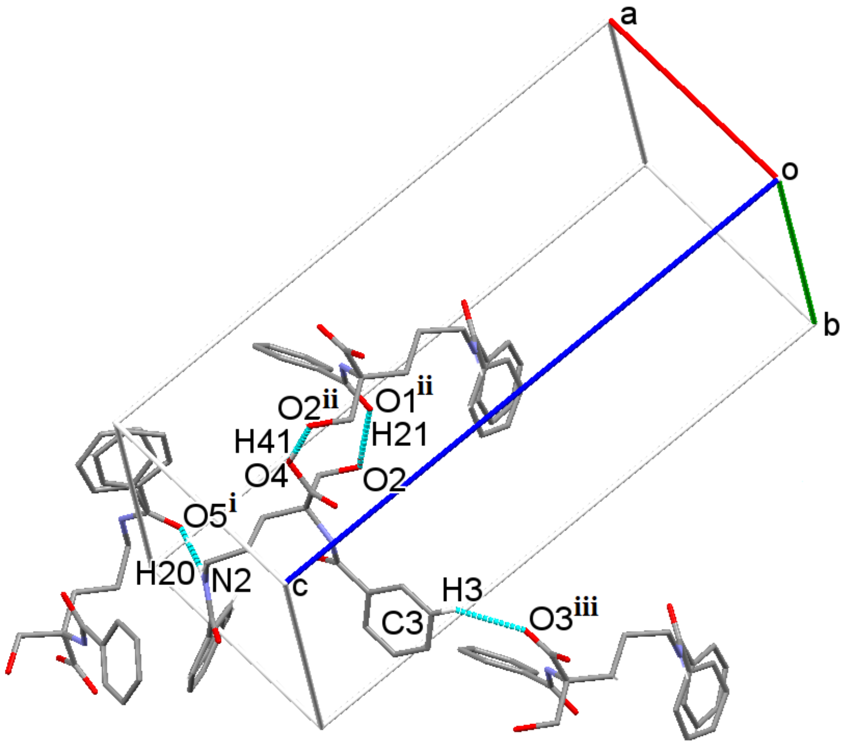

Figure S2. The intermolecular hydrogen bonds in the assymetric unit of (**1**) [symmetry codes: *(i) ½+x, 3/2-y, 2-z; (ii) 1-x, -1/2+y, 3/2-z; (iii) –x, ½+y, 3/2-z*].

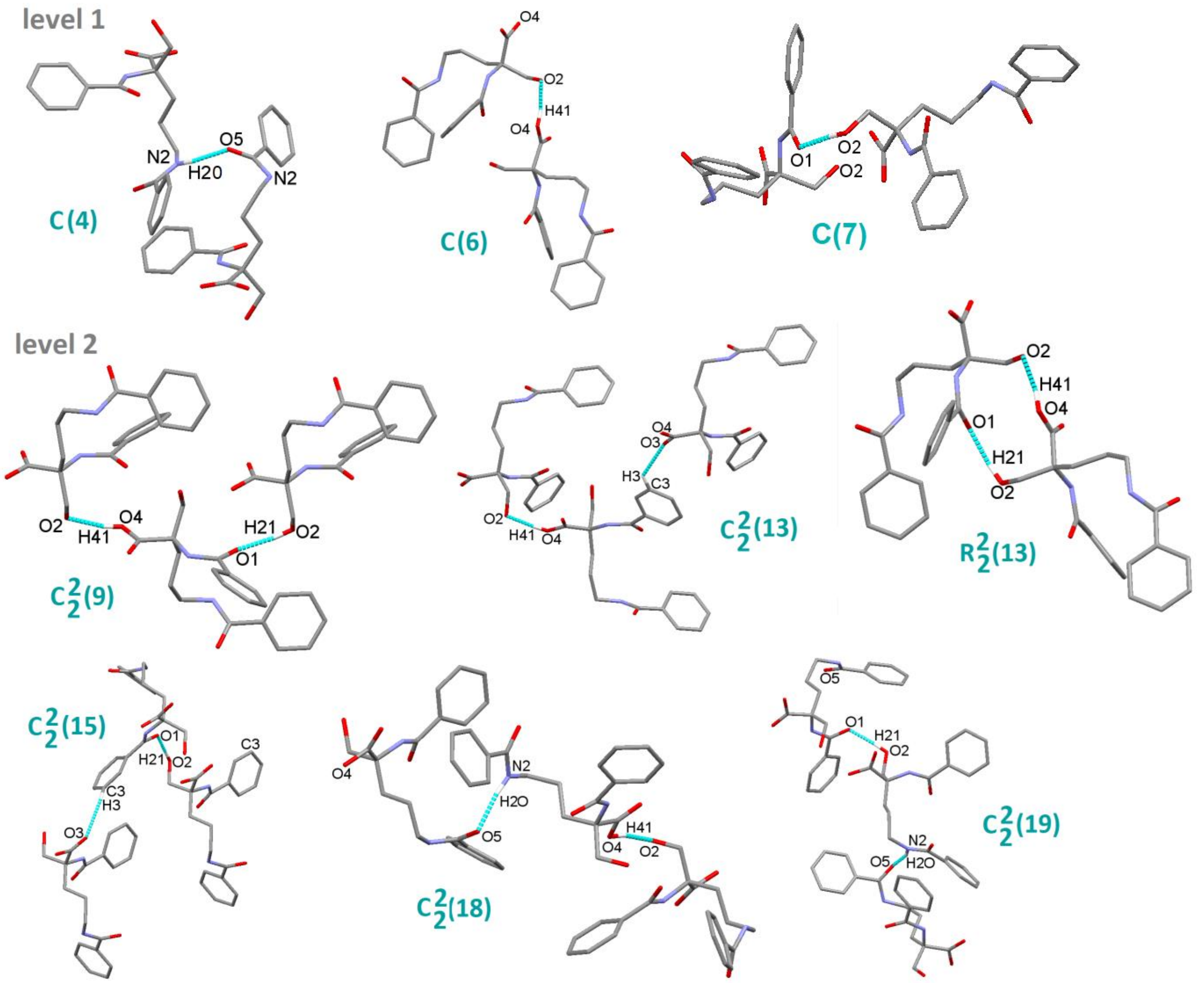

Figure S3. Supramolecular synthons in the assembly of (**1**). For clarity, hydrogen atoms not involved in the inter-contacts were omitted.

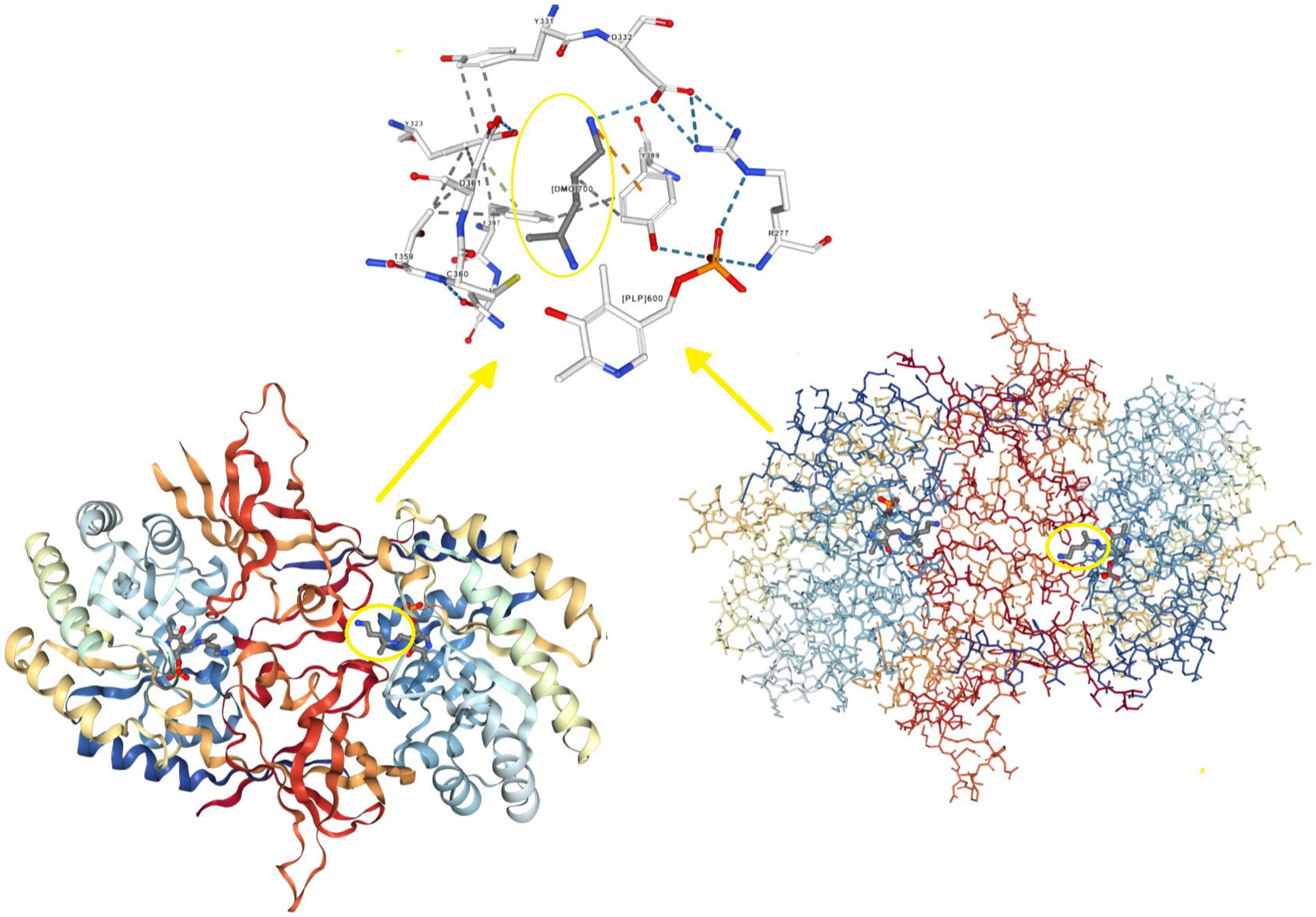

Figure S4. Ligand (difluoromethylornithine) interactions in the complex of ornithine decarboxylase with alpha-difluoromethylornithine (2TOD.pdb) (Grishin, 1999).

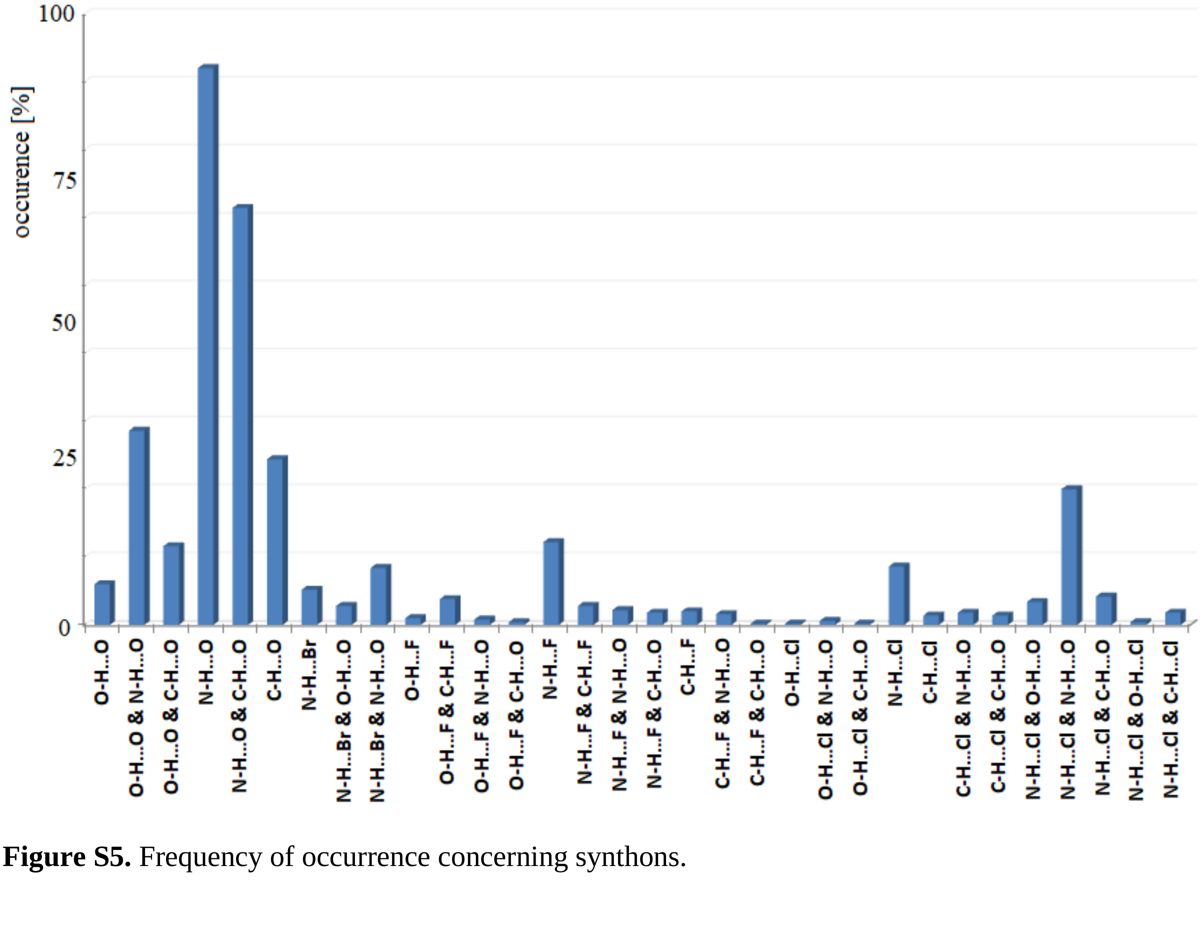

Figure S5. Frequency of occurrence concerning synthons.

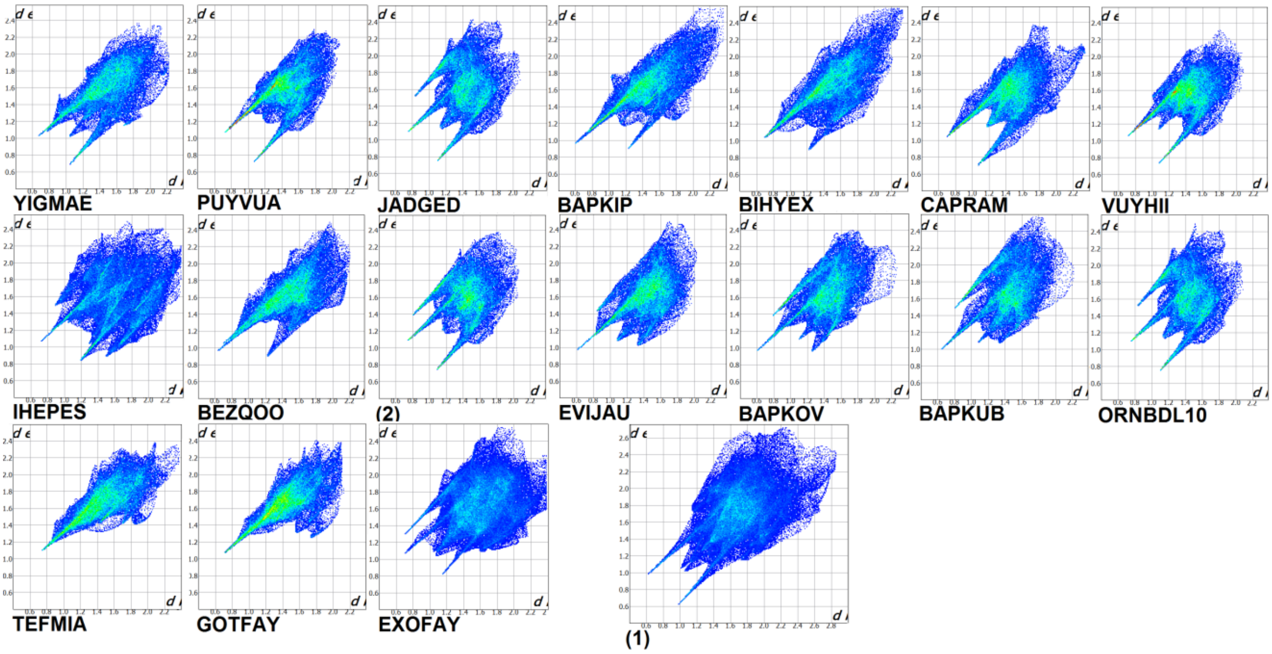

Figure S6. Full FP plots of all studied crystals.

Table S1. Basic information concerning ornithine derivatives retrieved from the CSD.

CSD ref. code	*formula*	*Crystal system*	*Space group*	*Unit cel parameters[Å; °]*	*Z*	*R1*	*T*
**BAPKIP**	*tris(1-carboxybutane-1,4-diaminium) dinitrate bis(sulfate) [tris(ornithine) dinitrate bis(sulfate)]*						
	$C_{15}H_{42}N_8O_{20}S_2$	hexagonal	*P*6$_3$	*a* = 10.0952(8)<br>*b* = 10.0952(8)<br>*c* = 17.7907(16)<br>*α* = 90, *β* = 90, *γ* =120	2	4.84	room
**BAPKOV**	*bis(1-carboxybutane-1,4-diaminium) dichloride sulfate [bis(ornithine) dichloride sulfate]*						
	$C_{10}H_{28}Cl_2N_4O_8S_1$	monoclinic	*C*2	*a* =13.9500(6)<br>*b* =9.2300(4)<br>*c* =9.3169(7)<br>*α* = *γ* = 90, *β* =127.260(1)	2	2.48	room
**BAPKUB**	*bis(1-carboxybutane-1,4-diaminium) dibromide sulfate [bis(ornithine) dibromide bis(sulfate)*						
	$C_{10}H_{28}Br_2N_4O_8S_1$	monoclinic	*C*2	*a* = 14.129(2)<br>*b* = 9.389(1)<br>*c* = 9.437(3)<br>*α* = *γ* = 90, *β* = 127.628(5)	2	7.36	room
**BEZQOO**	*L-Ornithinium hexafluorosilicate monohydrate*						
	$C_5H_{16}F_6N_2O_3Si_1$	orthorhombic	*P*2$_1$2$_1$2$_1$	*a* = 6.1310(1)<br>*b* = 9.8651(2)<br>*c* = 19.4339(3)<br>*α* = *β* = *γ* = 90	4	3.28	room
**BIHYEX**	*L-Ornithinium sulfate monohydrate*						
	$C_5H_{16}N_2O_7S_1$	orthorhombic	*P*2$_1$2$_1$2$_1$	*a* = 6.1589(2)<br>*b* = 9.682(6)<br>*c* = 18.714(6)<br>*α* = *β* = *γ* = 90	4	5.87	room
**CAPRAM**	*L-Ornithine L-aspartate hemihydrate*						
	$C_9H_{20}N_3O_{6.499}$	monoclinic	*C*2	*a* = 21.858(2)<br>*b* = 4.718(1)	4	4.1	room

				c = 18.046(2)<br>$\alpha = \gamma$ = 90, β = 137.4(1)			
**EVIJAU**	*bis(L-Ornithinium) chloride nitrate sulfate*						
	$C_{10}H_{28}Cl_1N_5\,O_{11}S_1$	monoclinic	*C*2	a = 13.872(5)<br>b = 9.578(6)<br>c = 9.178(1)<br>$\alpha = \gamma$ = 90, β = 125.350(14)	2	3.83	room
**EXOFAY**	*N-(9-Fluorenyl)methoxycarbonyl-L-ornithine hydrochloride diethyl ether clathrate*						
	$C_{20}H_{23}N_2O_4^+$, Cl^-,<br>$C_4H_{10}O$	monoclinic	$P2_1$	a = 5.094(1)<br>b = 15.499(3)<br>c = 15.575(3)<br>$\alpha = \gamma$ = 90, β = 96.74(3)	2	5.43	room
**GOTFAY**	*1-carboxybutane-1,4-diaminium bis(2,4,6-trinitrophenolate) [L-ornithinium dipicrate]*						
	$C_{17}H_{18}N_8O_{16}$	monoclinic	$P2_1$	a = 7.3909(4)<br>b = 14.0725(7)<br>c = 11.7089(6)<br>$\alpha = \gamma$ = 90, β = 104.655(3)	2	3.49	room
**IHEPES**	*catena-[(5-ammonio-2-(difluoromethyl)norvalinato)-(m-chloro)-dichloro-copper monohydrate]*<br>*[catena-[(m-eflornithine)-(m-chloro)-chloro-copper(ii) monohydrate]*						
	$(C_6H_{12}Cl_2CuF_2N_2O_2)_n$<br>$\cdot H_2O$	monoclinic	*C*c	a = 13.130(1)<br>b = 12.186(1)<br>c = 8.193(<1)<br>$\alpha = \gamma$ = 90, β = 118.36(<1)	4	1.58	100
**JADGED**	*2,5-diammoniopentanoate 4-carboxy-2-oxobutanoate*						
	$C_{10}H_{18}N_2O_7$	monoclinic	$P2_1$	a = 15.4326(3)<br>b = 5.2015(1)<br>c = 16.2067(3)<br>$\alpha = \gamma$ = 90, β = 91.986(1)	4	4.7	room
***NAGLYC**	*L-Ornithine L-aspartate monohydrate*						
	$C_9H_{21}N_3O_7$	triclinic	*P* -1	a = 4.718(1)<br>b = 11.181(2)<br>c = 12.653(2)<br>α = 102.8(1)<br>β = 100.7(1)<br>γ = 77.8(1)	2	-	room
**ORNBDL01**	*DL-Ornithine hydrobromide*						
	$C_5H_{13}N_2O_2^+\,Br^-$	monoclinic	$P2_1$/c	a = 12.180(20)<br>b = 7.880(20)<br>c = 11/610(20)<br>$\alpha = \gamma$ = 90, β = 133.65(33)	4	13	room
**ORNBDL10**	*DL-Ornithine hydrobromide*						
		monoclinic	$P2_1$/c	a = 9.388(9)<br>b = 7.901(8)<br>c = 11.663(13)<br>$\alpha = \gamma$ = 90, β = 109.83(25)	4	7.3	room
**ORNHCL**	*L-Ornithine hydrochloride*						
	$C_5H_{13}N_2O_2^+\,Cl^-$	monoclinic	$P2_1$	a = 10.005(10)<br>b = 7.992(6)<br>c = 5.000(10)<br>$\alpha = \gamma$ = 90, β = 96.98(5)	2	4.0	room
**ORNHCL11**	*L-Ornithine hydrochloride*						
	$C_5H_{13}N_2O_2^+$, Cl^-	monoclinic	$P2_1$	a = 4.990<br>b = 8.00<br>c = 10.00<br>$\alpha = \gamma$ = 90, β = 97.00	2	6.4	room
**ORNHCL12**		monoclinic	$P2_1$	a = 9.948(1)<br>b = 7.964(1)<br>c = 4.983(1)	2	0.89	100

				α = *γ* = 90, *β* = 83.13(1)			
**ORNHCL13**		monoclinic	$P2_1$	*a* = 9.948(1)<br>*b* = 7.964(1)<br>*c* = 4.983(1)<br>*α* = *γ* = 90, *β* = 83.13(1)	2	1.15	100
**ORNSEB**	*DL-Ornithinium hexabromo-selenium*<br>$C_5H_{14}B_{r6}N_2O_2Se$	triclinic	P *	a = 10.89(2)<br>b = 7.45(1)<br>c = 11.84(2)<br>*α* = 101.8(3)<br>*β* = 100.5(3)<br>*γ* = 103.3(3)	2	-	room
**PUYVUA**	*L-Ornithine nitrate*<br>$C_5H_{13}N_3O_5$	monoclinic	$P2_1$	*a* = 5.1944(6)<br>*b* = 7.803(1)<br>*c* = 11.050(1)<br>*α* = *γ* = 90, *β* = 98.75(1)	2	3.7	room
***QQQAOJ**	*L-Ornithine hydrobromide*<br>$C_5H_{13}BrN_2O_2$	monoclinic	$P2_1$	*a* = 10.21<br>*b* = 8.04<br>*c* = 4.98<br>*α* = 90<br>*β* = 97.0<br>*γ* = 90	2	-	room
**TEFMIA**	*L-Ornitine dipicrate*<br>$C_{17}H_{18}N_8O_{16}$	monoclinic	$P2_1$	*a* = 11.692(2)<br>*b* = 14.058(3)<br>*c* = 7.364(1)<br>*α* = *γ* = 90, *β* = 104.65(2)	2	6.4	room
**VUYHII**	*L-Ornithine D-aspartate monohydrate*<br>$C_9H_{21}N_3O_7$	monoclinic	$P2_1$	*a* = 5.118(1)<br>*b* = 7.881(2)<br>*c* = 16.025(2)<br>*α* = *γ* = 90, *β* = 91.78(2)	2	5.5	room
**YIGMAE**	*bis(2,5-diammoniopentanoate) hexafluorosilicate [bis(L-ornithine) hexafluorosilicate]*<br>$C_{10}H_{26}F_6N_4O_4Si_1$	monoclinic	$P2_1$	*a* = 9.846(2)<br>*b* = 20.641(4)<br>*c* = 13.789(3)<br>*α* = *γ* = 90, *β* = 110.67(3)	6	10.69	room

**worse quality (or containing errors) structures*

Table S2. Cartesian coordinates (Å) of ornithine derivative (**1**), optimized at the M06/6–311++G(d,p) level of theory.

**The M06 optimized ornithine derivative (1) in gas phase**			
8	4.728254	7.994915	18.870503
8	4.723641	5.275528	16.032248
1	4.807266	4.362551	15.748489
8	2.814411	3.547106	18.486564
8	4.897736	2.852706	18.078967
1	4.432576	2.004487	18.053636
8	3.352609	6.966873	22.929590
7	3.505424	6.102550	18.593343

1	2.587087	5.680333	18.649001
7	5.509407	6.799240	22.286432
1	6.352573	7.291564	22.037719
6	2.362523	8.201552	18.946922
6	1.145640	7.759553	18.429754
1	1.091545	6.843551	17.847608
6	-0.006732	8.507801	18.615582
1	-0.947837	8.160951	18.200360
6	0.044898	9.699263	19.326386
1	-0.860198	10.279673	19.478522
6	1.255835	10.150168	19.834656
1	1.305143	11.081084	20.391857
6	2.410703	9.411341	19.635625
1	3.365509	9.759367	20.018423
6	3.641563	7.441942	18.795109
6	4.612738	5.170460	18.476930
6	5.451855	5.458897	17.222391
1	6.352545	4.832395	17.248045
1	5.761284	6.505756	17.266837
6	3.980135	3.795612	18.352734
6	5.511347	5.188485	19.727871
1	6.362537	4.516662	19.559819
1	5.903040	6.208748	19.803728
6	4.785041	4.816184	21.010887
1	4.682851	3.725499	21.096320
1	3.767244	5.224566	21.007172
6	5.490880	5.349262	22.249637
1	6.529641	5.000652	22.287379
1	14.985577	4.985612	23.150145
6	4.386047	7.510251	22.583515
6	4.492652	8.997843	22.468934
6	5.441091	9.640104	21.675598
1	6.143289	9.068637	21.074777
6	5.448741	11.024148	21.577008
1	6.178612	11.514823	20.940940
6	4.510252	11.775823	22.271221
1	4.517235	12.858922	22.192292
6	3.557079	11.140135	23.057733
1	2.818489	11.725660	23.597040
6	3.544583	9.757976	23.149688
1	2.796141	9.236890	23.738366

The M06 optimized ornithine derivative (1) in solvated state (CPCM method)

8	0.166469	0.311465	1.669694
8	3.926055	1.184671	2.023178
1	4.860711	1.062531	1.833319
8	3.841272	0.685312	-1.488838
8	4.858234	0.890765	-0.271788
1	5.551262	0.771413	-0.939300
8	-1.352595	-1.383301	-2.146508
7	1.740567	0.799983	0.104828

1	1.945808	1.316936	-0.742285
7	-0.597009	-2.750403	-0.513749
1	-0.790847	3.179100	0.378827
6	-0.338843	2.025803	0.107317
6	0.156311	3.126275	-0.591688
1	1.226602	3.281838	-0.695086
6	-0.715707	4.060572	-1.132408
1	-0.321500	4.920661	-1.664202
6	-2.087970	3.897672	-0.989559
1	-2.768354	4.625822	-1.420222
6	-2.587293	2.805903	-0.289310
1	-3.658889	2.671505	-0.174988
6	-1.716485	1.881427	0.266484
1	-2.095601	1.026210	0.818821
6	0.538841	0.974101	0.709183
6	2.707861	-0.234613	0.442967
6	3.253377	-0.045096	1.865289
1	3.901578	-0.893001	2.112639
1	2.404307	-0.054326	2.552261
6	3.847160	-0.073768	-0.554447
6	2.107458	-1.643759	0.289136
1	2.845212	-2.381529	0.625757
1	1.257439	-1.694877	0.978395
6	1.656897	-1.954149	-1.130530
1	2.518341	-2.218919	-1.757805
1	1.200673	-1.065017	-1.583331
6	0.642624	-3.086355	-1.193611
1	1.039673	-3.990904	-0.722492
1	0.419973	-3.329277	-2.237652
6	-1.485438	-1.870528	-1.027988
6	-2.642853	-1.508446	-0.154468
6	-2.583970	-1.567633	1.237538
1	-1.672540	-1.878250	1.741014
6	-3.668475	-1.157861	2.000753
1	-3.608644	-1.192348	3.083907
6	-4.819377	-0.688563	1.380428
1	-5.665940	-0.366918	1.979242
6	-4.883231	-0.625070	-0.006939
1	-5.781002	-0.257289	-0.493987
6	-3.797632	-1.027382	-0.768811
1	-3.828176	-0.969633	-1.852301

Table S3

Gemetrical parameters (in Ǻ and in o) for the π-stacking moieties involved in the π···π interactions forstudied compounds.

CgI-CgJ[a,b]	symmetry	Cg···Cg[c]	Cg(I)-perp[d]	Cg(J)-per[pe]	α[f]	β[g]	γ[f]
**(1)**							

Cg1···Cg1	-x, ½+y, 3/2-z	5.8060(19)	0.7192(13)	-4.4381(13)	62.45(15)	40.1	82.9
Cg1···Cg2	-1/2+x, 3/2-y, 2-z	5.744(9)	1.9329(13)	5.054(10)	45	28.4	70.3
Cg2···Cg2	-1/2+x, 5/2-y, 2-z	5.843(14)	2.726(11)	-5.293(11)	52	25.1	62.2
**EXOFAY***							
Cg1···Cg2	1+x, y, z	4.048(6)	-3.480(4)	3.558(4)	2.3(5)	28.5	30.7
Cg1···Cg3	-1+x, y, z	4.838(7)	3.516(4)	-3.549(6)	1.1(6)	42.8	43.4
Cg2···Cg1	-1+x, y, z	4.049(6)	3.559(4)	-3.480(4)	2.3(5)	30.7	28.5
Cg2···Cg3	-1+x, y, z	4.652(7)	3.549(4)	-3.431(6)	2.2(6)	42.5	40.3
Cg3···Cg1	1+x, y, z	4.837(7)	-3.549(6)	3.516(4)	1.1(6)	43.4	42.8
Cg3···Cg2	1+x, y, z	4.651(7)	-3.431(6)	3.548(4)	2.2(6)	40.3	42.5
**GOTFAY**							
Cg1···Cg2	x, y, z	4.2034(10)	3.4096(8)	-3.7073(7)	9.91(8)	28.1	35.8
Cg2···Cg1	-1+x, y, z	4.2121(10)	3.6061(7)	-3.6559(8)	9.91(8)	29.8	31.1
**TEFMIA**							
Cg1···Cg2	-x, -1/2+y, 2-z	4.191(3)	3.6832(17)	3.3926(17)	9.78(19)	36	28.5

Table S4

Gemetrical parameters (in Å and in °) for the other π-stacking moieties forstudied compounds.

Y-X···Cg	symmetry	X(H)···Cg	Y-X···Cg	Y···Cg
(**1**)				
C14-O5···Cg1	½+x, 3/2-y, 2-z	3.545(3)	99.27(19)	3.941(3)
**EXOFAY**				
C8-H15···Cg2	1+x, y, z	2.82	143	3.649(9)
C22-H27···Cg3	-x, -1/2+y, 2-z	2.99	130	3.69(4)
**GOTFAY**				
N3-O6···Cg2	1+x, y, z	3.769(3)	73.57(16)	3.618(2)
**TEFMIA**				
N5-O11···Cg1	-x, ½+y, 1-z	3.763(6)	74.0(3)	3.621(5)
N5-O12···Cg1	-x, ½+y, 2-z	3.996(7)	81.4(4)	4.002(5)

[c] distance between ring centroids [Å], [d] perpendicular distance of Cg(I) on ring J [Å], [e] perpendicular distance of Cg(J) on ring I [Å], [f] dihedral angle between planes I and J [°], [g] angle between the centroid vector Cg(I)···Cg(J) and normal to plane I [°], [h] angle between the centroid vector Cg(I)···Cg(J) and normal to plane J [°].

Table S5. Galley of ornithine-based synthons (selected, below 20-membered). Synthons without participation of ornithine moiety are grey.

name	*synthon*	*interactions*
(**1**)	*C*(4)	**N2**$_{\mathbf{ornithine}}$**-H20**$_{(-NH)}$···O5$_{(-C=O)}$
	C(6)	**O4**$_{\mathbf{ornithine}}$**-H41**$_{(-COOH)}$···O2$_{(-OH)}$
	C(7)	O2-H21$_{(-OH)}$···O1$_{(-C=O)}$
	C(9)	C3-H3$_{(-Carom.)}$ ···O3$_{(-COOH)}$
level 2	$C^{2}_{2}(9)$	O2-H21$_{(-OH)}$···O1$_{(-C=O)}$ & **O4**$_{\mathbf{ornithine}}$**-H41**$_{(-COOH)}$···O2$_{(-OH)}$
	$C^{2}_{2}(12)$	O2-H21$_{(-OH)}$···O1$_{(-C=O)}$ & C3-H3$_{(-Carom.)}$ ···O3$_{(-COOH)}$
	$C^{2}_{2}(13)$	**O4**$_{\mathbf{ornithine}}$**-H41**$_{(-COOH)}$···O2$_{(-OH)}$ & C3-H3$_{(-Carom.)}$ ···O3$_{(-COOH)}$
	$C^{2}_{2}(15)$	**O4**$_{\mathbf{ornithine}}$**-H41**$_{(-COOH)}$···O2$_{(-OH)}$ & C3-H3$_{(-Carom.)}$ ···O3$_{(-COOH)}$
	$C^{2}_{2}(16)$	O2-H21$_{(-OH)}$···O1$_{(-C=O)}$ & C3-H3$_{(-Carom.)}$ ···O3$_{(-COOH)}$
	$C^{2}_{2}(18)$	**O4**$_{\mathbf{ornithine}}$**-H41**$_{(-COOH)}$···O2$_{(-OH)}$ & **N2**$_{\mathbf{ornithine}}$**-H20**$_{(-NH)}$···O5$_{(-C=O)}$
	$C^{2}_{2}(19)$	O2-H21$_{(-OH)}$···O1$_{(-C=O)}$ & **N**$_{\mathbf{2ornithine}}$**-H20**$_{(-NH)}$···O5$_{(-C=O)}$
	$R^{2}_{2}(13)$	O2-H21$_{(-OH)}$···O1$_{(-C=O)}$ & **O4**$_{\mathbf{ornithine}}$**-H41**$_{(-COOH)}$···O2$_{(-OH)}$
**(2)**	S(6)	C6-H6A···O2
	C(5)	*N9-H9B···O2<br>*N9-H9C···O2
	C(6)	C6-H6A···O10
	C(8)	N8-H8B···O10
	D(2)	*N9-H9A···Cl1<br>*N8-H8A···Cl1<br>*N8-H8C···Cl1<br>*C7-H7A···Cl1<br>*C6-H6B···Cl1<br>*C5-H5A···Cl1
Level 2	$C^{1}_{2}(4)$	*N9-H9B···O2 & N9-H9C···O2<br>*N8-H8A···Cl1 & N8-H8C···Cl1
	$C^{1}_{2}(5)$	*N8-H8A···Cl1 & C7-H7A···Cl1<br>*N8-H8C···Cl1 & C7-H7A···Cl1<br>*C6-H6B···Cl1 & $(C7\text{-}H7A\cdots Cl1)_2$<br>*C5-H5A···Cl1 & C6-H6B···Cl1
	$C^{1}_{2}(6)$	*N8-H8C···Cl1 & C6-H6B···Cl1<br>*C5-H5A···Cl1 & C7-H7A···Cl1
	$C^{1}_{2}(7)$	*N9-H9A···Cl1 & C6-H6B···Cl1<br>*N8-H8A···Cl1 & C5-H5A···Cl1<br>*N8-H8C···Cl1 & C5-H5A···Cl1
	$C^{1}_{2}(8)$	N9-H9A···Cl1 & C7-H7A···Cl1
	$C^{1}_{2}(9)$	*N9-H9A···Cl1 & N8-H8A···Cl1<br>*N8-H8C···Cl1 & N9-H9A···Cl1
	$C^{2}_{2}(9)$	*N9-H9B···O2 & C6-H6A···O10<br>*C7-H7A···Cl1 & C6-H6A···O10
	$C^{2}_{2}(10)$	N9-H9B···O2 & N9-H9C···O2
	$C^{2}_{2}(11)$	*N8-H8B···O10 & N9-H9B···O2<br>*N9-H9B···O2 & C6-H6A···O10<br>*N8-H8B···Cl1 & N9-H9C···O2
	$C^{2}_{2}(13)$	N8-H8B···O10 & N9-H9B···O2
	$C^{2}_{2}(14)$	N8-H8B···O10 & C6-H6A···O10
	$C^{3}_{4}(14)$	$(N9\text{-}H9C\cdots O2)_2$ & $(N9\text{-}H9B\cdots O2)_2$
	$C^{4}_{4}(20)$	$(C6\text{-}H6A\cdots O10)_2$ & $(N9\text{-}H9B\cdots O2)_2$
	$R^{1}_{2}(6)$	*$(N9\text{-}H9A\cdots Cl1)_2$ & C5-H5A···Cl1<br>*N8-H8A…Cl1 & C6-H6B···Cl1<br>*N8-H8B···O10 & C6-H6A···O10
	$R^{2}_{2}(11)$	N9-H9C···O2 & C6-H6A···O10
	$R^{2}_{2}(13)$	N8-H8B···O10 & N9-H9C···O2
	$R^{3}_{4}(14)$	$(N9\text{-}H9B\cdots O2)_2$ & N9-H9C···O2
	$R^{4}_{4}(20)$	$(N9\text{-}H9B\cdots O2)_2$ & $(C6\text{-}H6A\ldots O10)_2$
	$D^{3}_{3}(10)$	*$(N9\text{-}H9A\cdots Cl1)_2$ & N9-H9B···O2<br>*$(N9\text{-}H9A\cdots Cl1)_2$ & N9-H9C···O2
	$D^{3}_{3}(11)$	*C6-H6A···O10 & $(C6\text{-}H6B\cdots Cl1)^2$<br>*$(C5\text{-}H5A\cdots Cl1)_2$ & C6-H6A···O10
	$D^{3}_{3}(12)$	*N9-H9B···O2 & $(C5\text{-}H5A\cdots Cl1)_2$<br>*N9-H9C···O2 & $(C5\text{-}H5A\cdots Cl1)_2$
	$D^{3}_{3}(13)$	*N9-H9A···Cl1 & C6-H6A···O10<br>*N8-H8A···Cl1 & N8-H8B···O10<br>*N8-H8B···O10 & $(N8\text{-}H8C\cdots Cl1)_2$<br>*N8-H8B···O10 & $(C7\text{-}H7A\cdots Cl1)_2$

		*N8-H8B···O10 & C6-H6A···O10 & C6-H6B···Cl1<br>*N8-H8B···O10 & (C5-H5A···Cl1)$_2$<br>*C6-H6A···O10 & (C7-H7A···Cl1)$_2$
	$D^{3}_{3}(14)$	*N9-H9B···O2 & (C6-H6B···Cl1)2<br>*N9-H9C···O2 & (C6-H6B···Cl1)$_2$
	$D^{3}_{3}(15)$	*(N9-H9A···Cl1)$_2$ & N8-H8B···O10<br>*(N8-H8A···Cl1)$_2$ & C6-H6A···O10<br>*(N8-H8C···Cl1)$_2$ & C6-H6A···O10
	$D^{3}_{3}(16)$	*N9-H9B···O2 & (C7-H7A···Cl1)$_2$<br>*(N8-H8C···Cl1 & N9-H9C···O2 & C7-H7A···Cl1
	$D^{3}_{3}(18)$	*(N8-H8A…Cl1)$_2$ & N9-H9B···O2<br>*(N8-H8C···Cl1)$_2$ & N9-H9B···O2<br>*(N8-H8A…Cl1)$_2$ & N9-H9C···O2
**BAPKIB**	$D(2)$	*O1-H1$_{(-COOH)}$···O8$_{(SO4)}$<br>*N1-H3$_{(-NH3+)}$ ···O6A$_{(SO4)}$<br>*N1-H4$_{(-NH3+)}$ ···O8A$_{(SO4)}$<br>*N2-H12$_{(-NH3+)}$ ···O6A$_{(SO4)}$<br>*N2-H13$_{(-NH3+)}$ ···O3$_{(-NO3-)}$<br>*N2-H14$_{(-NH3+)}$ ···O4$_{(-NO3-)}$<br>*C2-H5$_{(-CH)}$···O5$_{(SO4)}$<br>*C5-H1O$_{(-CH2)}$ ···O7$_{(SO4)}$
	$D^{1}_{2}(3)$	*(C2-H2$_{(-CH)}$···O5$_{(SO4)}$)$_2$<br>*(C5-H10$_{(-CH2)}$ ···O7$_{(SO4)}$)$_2$
	$D^{2}_{2}(5)$	*O1-H1$_{(-COOH)}$···O8$_{(SO4)}$ & O1-H1$_{(-COOH)}$···O8A$_{(SO4)}$<br>*N1-H3$_{(-NH3+)}$ ···O6A$_{(SO4)}$ & N1-H3$_{(-NH3+)}$ ···O6$_{(SO4)}$<br>*N1-H4$_{(-NH3+)}$ ···O8A$_{(SO4)}$ & N1-H4$_{(-NH3+)}$ ···O8$_{(SO4)}$<br>*N2-H12$_{(-NH3+)}$ ···O6$_{(SO4)}$ & N2-H12$_{(-NH3+)}$ ···O6A$_{(SO4)}$<br>*N2-H13$_{(-NH3+)}$ ···O3A$_{(-NO3-)}$ & N2-H13$_{(-NH3+)}$ ···O3$_{(-NO3-)}$<br>*N2-H14$_{(-NH3+)}$ ···O4$_{(-NO3-)}$ & N2-H14$_{(-NH3+)}$ ···O4A$_{(-NO3-)}$
	$R^{3}_{3}(15)$	(N1-H5$_{(-NH3+)}$ ···O2 $_{(-COOH)}$ **ornithine**)$_2$
level 2	$C^{1}_{2}(9)$	N1-H3$_{(-NH3+)}$ ···O6A$_{(SO4)}$ & N2-H12$_{(-NH3+)}$ ···O6A$_{(SO4)}$
	$C^{2}_{2}(9)$	O1-H1$_{(-COOH)}$···O8$_{(SO4)}$ & N1-H4$_{(-NH3+)}$ ···O8A$_{(SO4)}$
	$C^{2}_{2}(10)$	N2-H12$_{(-NH3+)}$ ···O6$_{(SO4)}$ & C2-H2$_{(-CH)}$···O5$_{(SO4)}$
	$C^{2}_{2}(11)$	*O1-H1$_{(-COOH)}$···O8$_{(SO4)}$ & C5-H10$_{(-CH2)}$ ···O7$_{(SO4)}$<br>*N1-H3$_{(-NH3+)}$ ···O6A$_{(SO4)}$ & N2-H12$_{(-NH3+)}$ ···O6$_{(SO4)}$
	$C^{2}_{2}(14)$	(C2-H2$_{(-CH)}$···O5$_{(SO4)}$)$_2$ & (C5-H10$_{(-CH2)}$ ···O7$_{(SO4)}$)$_2$
	$C^{2}_{3}(9)$	N1-H5$_{(-NH3+)}$ ···O2$_{(-COOH)}$ **ornithine** & (C2-H2$_{(-CH)}$···O5$_{(SO4)}$)$_2$
	$C^{2}_{3}(15)$	N1-H5$_{(-NH3+)}$ ···O2$_{(-COOH)}$ **ornithine** & (C5-H10$_{(-CH2)}$ ···O7$_{(SO4)}$)$_2$
	$C^{3}_{3}(11)$	*N1-H5$_{(-NH3+)}$ ···O2 $_{(-COOH)}$ **ornithine** & N1-H3$_{(-NH3+)}$ ···O6$_{(SO4)}$ & N1-H3$_{(-NH3+)}$ ···O6A$_{(SO4)}$<br>*N1-H4$_{(-NH3+)}$ ···O8$_{(SO4)}$ & N1-H4$_{(-NH3+)}$ ···O8A$_{(SO4)}$ & N1-H5$_{(-NH3+)}$ ···O2$_{(-COOH)}$ **ornithine**
	$C^{3}_{3}(19)$	*N1-H5$_{(-NH3+)}$ ···O2 $_{(-COOH)}$ **ornithine** & N2-H13$_{(-NH3+)}$ ···O3$_{(-NO3-)}$ & N2-H13$_{(-NH3+)}$ ···O3B$_{(-NO3-)}$<br>*N2-H14$_{(-NH3+)}$ ···O1$_{(-COOH)}$ **ornithine** & N2-H14$_{(-NH3+)}$ ···O4B$_{(-NO3-)}$ & N1-H5$_{(-NH3+)}$ ···O2$_{(-COOH)}$ **ornithine**
	$C^{3}_{4}(12)$	*N1-H4$_{(-NH3+)}$ ···O8A$_{(SO4)}$ & N1-H4$_{(-NH3+)}$ ···O8B$_{(SO4)}$ & (C2-H2$_{(-CH)}$···O5$_{(SO4)}$)$_2$<br>*N2-H12$_{(-NH3+)}$ ···O6B$_{(SO4)}$ & (C5-H10$_{(-CH2)}$ ···O7$_{(SO4)}$)$_2$<br>*N2-H14$_{(-NH3+)}$ ···O4$_{(-NO3-)}$ & (C5-H10$_{(-CH2)}$ ···O7$_{(SO4)}$)$_2$
	$C^{3}_{4}(14)$	O1-H1$_{(-COOH)}$···O8$_{(SO4)}$ & O1-H1$_{(-COOH)}$···O8A$_{(SO4)}$ & (C2-H2$_{(-CH)}$···O5$_{(SO4)}$)$_2$
	$C^{3}_{4}(18)$	*N2-H13$_{(-NH3+)}$ ···O3$_{(-NO3-)}$ & N2-H13$_{(-NH3+)}$ ···O3A$_{(-NO3-)}$ & (C2-H2$_{(-CH)}$···O5$_{(SO4)}$)$_2$<br>*N1-H3$_{(-NH3+)}$ ···O6$_{(SO4)}$ & N1-H3$_{(-NH3+)}$ ···O6A$_{(SO4)}$ & (C5-H10$_{(-CH2)}$ ···O7$_{(SO4)}$)$_2$
	$C^{3}_{4}(20)$	O1-H1$_{(-COOH)}$···O8$_{(SO4)}$ & (C5-H10$_{(-CH2)}$ ···O7$_{(SO4)}$)2
	$C^{4}_{4}(12)$	*N1-H3$_{(-NH3+)}$ ···O6$_{(SO4)}$ & N1-H3$_{(-NH3+)}$ ···O6A$_{(SO4)}$ & N1-H4$_{(-NH3+)}$ ···O8$_{(SO4)}$ & N1-H4$_{(-NH3+)}$ ···O8A$_{(SO4)}$<br>*N2-H12$_{(-NH3+)}$ ···O6$_{(SO4)}$ & N2-H12$_{(-NH3+)}$ ···O6A$_{(SO4)}$ & N2-H13$_{(-NH3+)}$ ···O3$_{(-NO3-)}$ & N2-H13$_{(-NH3+)}$ ···O3$_{(-NO3-)}$<br>*N2-H12$_{(-NH3+)}$ ···O6$_{(SO4)}$ & N2-H12$_{(-NH3+)}$ ···O6A$_{(SO4)}$ & N2-H14$_{(-NH3+)}$ ···O4$_{(-NO3-)}$ & N2-H14$_{(-NH3+)}$ ···O4B$_{(-NO3-)}$<br>*N2-H13$_{(-NH3+)}$ ···O3$_{(-NO3-)}$ & N2-H13$_{(-NH3+)}$ ···O3A$_{(-NO3-)}$ & N2-H14$_{(-NH3+)}$ ···O4$_{(-NO3-)}$ & N2-H14$_{(-NH3+)}$ ···O4A$_{(-NO3-)}$
	$C^{4}_{4}(18)$	*N1-H3$_{(-NH3+)}$ ···O6A$_{(SO4)}$ & N1-H3$_{(-NH3+)}$ ···O6B$_{(SO4)}$ & O1-H1$_{(-COOH)}$···O8$_{(SO4)}$ & O1-H1$_{(-COOH)}$···O8A$_{(SO4)}$<br>*O1-H1$_{(-COOH)}$···O8$_{(SO4)}$ & O1-H1$_{(-COOH)}$···O8A$_{(SO4)}$ & N1-H5$_{(-NH3+)}$ ···O2$_{(-COOH)}$<br>*N2-H12$_{(-NH3+)}$ ···O6B$_{(SO4)}$ & (C2-H2$_{(-CH)}$···O5$_{(SO4)}$)$_2$ & N2-H12$_{(-NH3+)}$ ···O6A$_{(SO4)}$
	$R^{2}_{2}(7)$	N1-H3$_{(-NH3+)}$ ···O6A$_{(SO4)}$ & C2-H5$_{(-CH)}$···O5$_{(SO4)}$
	$R^{2}_{2}(10)$	N1-H4$_{(-NH3+)}$ ···O8A$_{(SO4)}$ & C5-H10$_{(-CH2)}$ ···O7$_{(SO4)}$
	$R^{3}_{3}(19)$	N1-H5$_{(-NH3+)}$ ···O2$_{(-COOH)}$ **ornithine** & N2-H12$_{(-NH3+)}$ ···O6$_{(SO4)}$ & N2-H12$_{(-NH3+)}$ ···O6A$_{(SO4)}$
	$R^{3}_{4}(12)$	N2-H13$_{(-NH3+)}$ ···O3A$_{(-NO3-)}$ & (C5-H10$_{(-CH2)}$ ···O7$_{(SO4)}$)$_2$
	$R^{3}_{4}(18)$	N2-H14$_{(-NH3+)}$ ···O4$_{(-NO3-)}$ & N2-H14$_{(-NH3+)}$ ···O4A$_{(-NO3-)}$ & (C2-H5$_{(-CH)}$···O5$_{(SO4)}$)$_2$
	$D^{2}_{2}(5)$	*N1-H4$_{(-NH3+)}$ ···O8A$_{(SO4)}$ & N1-H3$_{(-NH3+)}$ ···O6A$_{(SO4)}$<br>*N2-H12$_{(-NH3+)}$ ···O6A$_{(SO4)}$ & N2-H13$_{(-NH3+)}$ ···O3$_{(-NO3-)}$<br>*N2-H12$_{(-NH3+)}$ ···O6A$_{(SO4)}$ & N2-H14$_{(-NH3+)}$ ···O4$_{(-NO3-)}$<br>*N2-H13$_{(-NH3+)}$ ···O3$_{(-NO3-)}$ & N2-H14$_{(-NH3+)}$ ···O4$_{(-NO3-)}$

	$D^{2}_{2}(6)$	*N1-H4$_{(-NH3+)}$···O8A$_{(SO4)}$ & C2-H5$_{(-CH)}$···O5$_{(SO4)}$<br>*N2-H12$_{(-NH3+)}$···O6A$_{(SO4)}$ & C5-H10$_{(-CH2)}$···O7$_{(SO4)}$<br>*N2-H13$_{(-NH3+)}$···O3$_{(-NO3-)}$ & (C5-H10$_{(-CH2)}$···O7$_{(SO4)}$)$_2$<br>*N2-H14$_{(-NH3+)}$···O4$_{(-NO3-)}$ & (C5-H10$_{(-CH2)}$···O7$_{(SO4)}$)$_2$
	$D^{2}_{2}(7)$	O1-H1$_{(-COOH)}$···O8$_{(SO4)}$ & C2-H2$_{(-CH)}$···O5$_{(SO4)}$
	$D^{2}_{2}(8)$	*O1-H1$_{(-COOH)}$···O8$_{(SO4)}$ & N1-H3$_{(-NH3+)}$···O6A$_{(SO4)}$<br>*C2-H2$_{(-CH)}$···O5$_{(SO4)}$ & C5-H10$_{(-CH2)}$···O7$_{(SO4)}$
	$D^{2}_{2}(9)$	*N2-H13$_{(-NH3+)}$···O3$_{(-NO3-)}$ & C2-H2$_{(-CH)}$···O5$_{(SO4)}$<br>*N2-H14$_{(-NH3+)}$···O4$_{(-NO3-)}$ & C2-H2$_{(-CH)}$···O5$_{(SO4)}$
	$D^{2}_{2}(10)$	*N1-H3$_{(-NH3+)}$···O6A$_{(SO4)}$ & N2-H13$_{(-NH3+)}$···O3$_{(-NO3-)}$<br>*N1-H3$_{(-NH3+)}$···O6A$_{(SO4)}$ & N2-H14$_{(-NH3+)}$···O4$_{(-NO3-)}$<br>*N1-H4$_{(-NH3+)}$···O8A$_{(SO4)}$ & N2-H12$_{(-NH3+)}$···O6A$_{(SO4)}$<br>*N1-H4$_{(-NH3+)}$···O8A$_{(SO4)}$ & N2-H13$_{(-NH3+)}$···O3$_{(-NO3-)}$<br>*N1-H4$_{(-NH3+)}$···O8A$_{(SO4)}$ & N2-H14$_{(-NH3+)}$···O4$_{(-NO3-)}$
	$D^{2}_{2}(11)$	*O1-H1$_{(-COOH)}$···O8$_{(SO4)}$ & N2-H12$_{(-NH3+)}$···O6A$_{(SO4)}$<br>*O1-H1$_{(-COOH)}$···O8$_{(SO4)}$ & N2-H13$_{(-NH3+)}$···O3$_{(-NO3-)}$<br>*O1-H1$_{(-COOH)}$···O8$_{(SO4)}$ & N2-H14$_{(-NH3+)}$···O4$_{(-NO3-)}$<br>*O1-H1$_{(-COOH)}$···O8$_{(SO4)}$ & N2-H14$_{(-NH3+)}$···O4$_{(-NO3-)}$
	$D^{2}_{3}(7)$	*N1-H4$_{(-NH3+)}$···O8B$_{(SO4)}$ & (C2-H5$_{(-CH)}$···O5$_{(SO4)}$)$_2$<br>*N2-H12$_{(-NH3+)}$···O6B$_{(SO4)}$ & (C5-H10$_{(-CH2)}$···O7$_{(SO4)}$)$_2$<br>*N2-H13$_{(-NH3+)}$···O3A$_{(-NO3-)}$ & (C5-H10$_{(-CH2)}$···O7$_{(SO4)}$)$_2$<br>*N2-H14$_{(-NH3+)}$···O4A$_{(-NO3-)}$ & (C5-H10$_{(-CH2)}$···O7$_{(SO4)}$)$_2$
	$D^{2}_{3}(8)$	O1-H1$_{(-COOH)}$···O8A$_{(SO4)}$ & (C2-H2$_{(-CH)}$···O5$_{(SO4)}$)$_2$
	$D^{2}_{3}(9)$	*(C2-H2$_{(-CH)}$···O5$_{(SO4)}$)$_2$ & C5-H10$_{(-CH2)}$···O7$_{(SO4)}$<br>*N2-H13$_{(-NH3+)}$···O3$_{(-NO3-)}$ & N2-H13$_{(-NH3+)}$···O3A$_{(-NO3-)}$ & C5-H10$_{(-CH2)}$···O7$_{(SO4)}$
	$D^{2}_{3}(10)$	*O1-H1$_{(-COOH)}$···O8$_{(SO4)}$ & O1-H1$_{(-COOH)}$···O8A$_{(SO4)}$ & C2-H2$_{(-CH)}$···O5$_{(SO4)}$<br>*N2-H13$_{(-NH3+)}$···O3A$_{(-NO3-)}$ & (C2-H2$_{(-CH)}$···O5$_{(SO4)}$)$_2$<br>*N2-H14$_{(-NH3+)}$···O4A$_{(-NO3-)}$ & (C2-H2$_{(-CH)}$···O5$_{(SO4)}$)$_2$<br>*N1-H3$_{(-NH3+)}$···O6B$_{(SO4)}$ & (C5-H10$_{(-CH2)}$···O7$_{(SO4)}$)$_2$
	$D^{3}_{3}(8)$	*N1-H3$_{(-NH3+)}$···O6A$_{(SO4)}$ & N4-H3$_{(-NO3-)}$···O6$_{(SO4)}$ & N1-H4$_{(-NH3+)}$···O8$_{(SO4)}$<br>*N1-H3$_{(-NH3+)}$···O6$_{(SO4)}$ & N1-H4$_{(-NH3+)}$···O8$_{(SO4)}$ & N1-H4$_{(-NH3+)}$···O8A$_{(SO4)}$<br>*N2-H12$_{(-NH3+)}$···O6A$_{(SO4)}$ & N2-H12$_{(-NH3+)}$···O6 & N2-H13$_{(-NH3+)}$···O3B$_{(-NO3-)}$<br>*N2-H13$_{(-NH3+)}$···O3$_{(-NO3-)}$ & N2-H13$_{(-NH3+)}$···O3A$_{(-NO3-)}$ & N2-H12$_{(-NH3+)}$···O6B$_{(SO4)}$<br>*N2-H12$_{(-NH3+)}$···O6$_{(SO4)}$ & N2-H12$_{(-NH3+)}$···O6A$_{(SO4)}$ & N2-H14$_{(-NH3+)}$···O4B$_{(-NO3-)}$<br>*N2-H14$_{(-NH3+)}$···O4$_{(-NO3-)}$ & N2-H14$_{(-NH3+)}$···O4A$_{(-NO3-)}$ & N2-H12$_{(-NH3+)}$···O6B$_{(SO4)}$<br>*N2-H13$_{(-NH3+)}$···O3$_{(-NO3-)}$ & N2-H13$_{(-NH3+)}$···O3A$_{(-NO3-)}$ & N2-H14$_{(-NH3+)}$···O4A$_{(-NO3-)}$<br>*N2-H14$_{(-NH3+)}$···O4$_{(-NO3-)}$ & N2-H14$_{(-NH3+)}$···O4A$_{(-NO3-)}$ & N2-H13$_{(-NH3+)}$···O3A$_{(-NO3-)}$
	$D^{3}_{3}(9)$	*N1-H4$_{(-NH3+)}$···O8A$_{(SO4)}$ & N1-H4$_{(-NH3+)}$···O8$_{(SO4)}$ & C2-H2$_{(-CH)}$···O5$_{(SO4)}$<br>*N2-H12$_{(-NH3+)}$···O6$_{(SO4)}$ & N2-H12$_{(-NH3+)}$···O6A$_{(SO4)}$ & (C5-H10$_{(-CH2)}$···O7$_{(SO4)}$)$_2$<br>*N2-H14$_{(-NH3+)}$···O4$_{(-NO3-)}$ & N2-H14$_{(-NH3+)}$···O4A$_{(-NO3-)}$ & C5-H10$_{(-CH2)}$···O7$_{(SO4)}$
	$D^{3}_{3}(11)$	*O1-H1$_{(-COOH)}$···O8$_{(SO4)}$ & O1-H1$_{(-COOH)}$···O8A$_{(SO4)}$ & N1-H3$_{(-NH3+)}$···O6B$_{(SO4)}$<br>*N1-H3$_{(-NH3+)}$···O6$_{(SO4)}$ & N1-H3$_{(-NH3+)}$···O6$_{(SO4)}$ & O1-H1$_{(-COOH)}$···O8B$_{(SO4)}$
	$D^{3}_{3}(12)$	*N2-H13$_{(-NH3+)}$···O3$_{(-NO3-)}$ & N2-H13A$_{(-NH3+)}$···O3A$_{(-NO3-)}$ & C2-H2$_{(-CH)}$···O5$_{(SO4)}$<br>*N2-H14$_{(-NH3+)}$···O4$_{(-NO3-)}$ & N2-H14$_{(-NH3+)}$···O4A$_{(-NO3-)}$ & (C2-H2$_{(-CH)}$···O5$_{(SO4)}$)$_2$<br>*N1-H3$_{(-NH3+)}$···O6$_{(SO4)}$ & N1-H3$_{(-NH3+)}$···O6A$_{(SO4)}$ & C5-H10$_{(-CH2)}$···O7$_{(SO4)}$
	$D^{3}_{3}(13)$	*N1-H3$_{(-NH3+)}$···O6$_{(SO4)}$ & N1-H3$_{(-NH3+)}$···O6A$_{(SO4)}$ & N2-H13$_{(-NH3+)}$···O3B$_{(-NO3-)}$<br>*N1-H3$_{(-NH3+)}$···O6B$_{(SO4)}$ & N2-H13$_{(-NH3+)}$···O3$_{(-NO3-)}$ & N2-H13$_{(-NH3+)}$···O3A$_{(-NO3-)}$<br>*N1-H3$_{(-NH3+)}$···O6$_{(SO4)}$ & N1-H3$_{(-NH3+)}$···O6A$_{(SO4)}$ & N2-H14$_{(-NH3+)}$···O4B$_{(-NO3-)}$<br>*N1-H3$_{(-NH3+)}$···O6B$_{(SO4)}$ & N2-H14$_{(-NH3+)}$···O4A$_{(-NO3-)}$ & N2-H14$_{(-NH3+)}$···O4$_{(-NO3-)}$<br>*N1-H4$_{(-NH3+)}$···O8$_{(SO4)}$ & N1-H4$_{(-NH3+)}$···O8A$_{(SO4)}$ & N2-H12$_{(-NH3+)}$···O6$_{(SO4)}$<br>*N1-H4$_{(-NH3+)}$···O8$_{(SO4)}$ & N2-H12$_{(-NH3+)}$···O6$_{(SO4)}$ & N2-H12$_{(-NH3+)}$···O6A$_{(SO4)}$<br>*N1-H4$_{(-NH3+)}$···O8B$_{(SO4)}$ & N2-H13$_{(-NH3+)}$···O3A$_{(-NO3-)}$ & N2-H13$_{(-NH3+)}$···O3$_{(-NO3-)}$<br>*N1-H4$_{(-NH3+)}$···O8A$_{(SO4)}$ & N2-H14$_{(-NH3+)}$···O4B$_{(-NO3-)}$ & N1-H4$_{(-NH3+)}$···O8$_{(SO4)}$<br>*N1-H4$_{(-NH3+)}$···O8B$_{(SO4)}$ & N2-H14$_{(-NH3+)}$···O4A$_{(-NO3-)}$ & N2-H4$_{(-NH3+)}$···O4$_{(-NO3-)}$
	$D^{3}_{3}(14)$	*O1-H1$_{(-COOH)}$···O8$_{(SO4)}$ & O1-H1$_{(-COOH)}$···O8A$_{(SO4)}$ & N2-H12$_{(-NH3+)}$···O6B$_{(SO4)}$<br>*O1-H1$_{(-COOH)}$···O8$_{(SO4)}$ & O1-H1$_{(-COOH)}$···O8A$_{(SO4)}$ & N2-H13$_{(-NH3+)}$···O3A$_{(-NO3-)}$<br>*O1-H1$_{(-COOH)}$···O8B$_{(SO4)}$ & N2-H12$_{(-NH3+)}$···O6A$_{(SO4)}$ & N2-H12$_{(-NH3+)}$···O6$_{(SO4)}$<br>*O1-H1$_{(-COOH)}$···O8$_{(SO4)}$ & O1-H1$_{(-COOH)}$···O8A$_{(SO4)}$ & N2-H13$_{(-NH3+)}$···O3A$_{(-NO3-)}$<br>*O1-H1$_{(-COOH)}$···O8A$_{(SO4)}$ & N2-H13$_{(-NH3+)}$···O3$_{(-NO3-)}$ & N2-H13$_{(-NH3+)}$···O3A$_{(-NO3-)}$<br>*O1-H1$_{(-COOH)}$···O8$_{(SO4)}$ & O1-H1$_{(-COOH)}$···O8A$_{(SO4)}$ & N2-H14$_{(-NH3+)}$···O4A$_{(-NO3-)}$<br>*O1-H1$_{(-COOH)}$···O4$_{(-NO3-)}$ & N2-H14$_{(-NH3+)}$···O4$_{(-NO3-)}$ & N2-H14$_{(-NH3+)}$···O4A$_{(-NO3-)}$
**BAPKOV**	$C(4)$	C2-H8$_{(-CH)}$···O2 $_{(-COOH)}$ **ornithine**
	$C(8)$	N2-H6$_{(-NH3+)}$···O2 $_{(-COOH)}$ **ornithine**
	$R^{2}_{2}(12)$	(C4-H12$_{(-CH2)}$···O1$_{(-COOH)}$)$_2$ **ornithine**
	$D(2)$	*O1-H1$_{(-COOH)}$···O4A $_{(-SO4)}$<br>*N1-H2$_{(-NH3+)}$···Cl1

		*N1-H3$_{(-NH3+)}$ ···O3$_{(-SO4)}$<br>*N1-H4$_{(-NH3+)}$ ···Cl2<br>*N2-H5$_{(-NH3+)}$ ···O3$_{(-SO4)}$<br>*N2-H5$_{(-NH3+)}$ ···O4A$_{(-SO4)}$<br>*N2-H6$_{(-NH3+)}$ ···Cl1<br>*N2-H7$_{(-NH3+)}$ ···Cl2<br>*C3-H10 $_{(-CH2)}$ ···O3A$_{(-SO4)}$
	$D^{1}_{2}(3)$	*(N1-H2$_{(-NH3+)}$ ···Cl1)$_2$<br>*(N1-H4$_{(-NH3+)}$ ···Cl2)$_2$<br>*(N2-H6$_{(-NH3+)}$ ···Cl1)$_2$<br>*(N2-H7$_{(-NH3+)}$ ···Cl2)$_2$
	$D^{2}_{2}(5)$	*O1-H1$_{(-COOH)}$ ···O4A$_{(-SO4)}$ & O1-H1$_{(-COOH)}$ ···O4$_{(-SO4)}$<br>*N1-H3$_{(-NH3+)}$ ···O3$_{(-SO4)}$ & N1-H3$_{(-NH3+)}$ ···O3A$_{(-SO4)}$<br>*N2-H5$_{(-NH3+)}$ ···O3$_{(-SO4)}$ & N2-H5$_{(-NH3+)}$ ···O3A$_{(-SO4)}$<br>*N2-H5$_{(-NH3+)}$ ···O4A$_{(-SO4)}$ & N2-H5$_{(-NH3+)}$ ···O4$_{(-SO4)}$<br>*C3-H10$_{(-CH2)}$ ···O3A$_{(-SO4)}$ & C3-H10$_{(-CH2)}$ ···O3$_{(-SO4)}$
level 2	$C^{1}_{2}(9)$	*N1-H2$_{(-NH3+)}$ ···Cl1 & N2-H6$_{(-NH3+)}$ ···Cl1<br>*N1-H3$_{(-NH3+)}$ ···O3$_{(-SO4)}$ & N2-H5$_{(-NH3+)}$ ···O3$_{(-SO4)}$<br>*N1-H4$_{(-NH3+)}$ ···Cl2 & N2-H7$_{(-NH3+)}$ ···Cl2
	$C^{1}_{2}(10)$	O1-H1$_{(-COOH)}$ ···O4A$_{(-SO4)}$& N2-H5$_{(-NH3+)}$ ···O4A$_{(-SO4)}$
	$C^{2}_{2}(9)$	*O1-H1$_{(-COOH)}$ ···O4A$_{(-SO4)}$ & N1-H3$_{(-NH3+)}$ ···O3$_{(-SO4)}$<br>*O1-H1$_{(-COOH)}$ ···O4$_{(-SO4)}$ & O1-H1$_{(-COOH)}$ ···O4A$_{(-SO4)}$ & C3-H10$_{(-CH2)}$ ···O3$_{(-SO4)}$
	$C^{2}_{2}(10)$	N2-H6$_{(-NH3+)}$ ···O2$_{(-COOH)}$ **ornithine** & (N2-H6$_{(-NH3+)}$ ···Cl1)$_2$
	$C^{2}_{2}(11)$	N1-H3$_{(-NH3+)}$ ···O3$_{(-SO4)}$ & N2-H5$_{(-NH3+)}$ ···O4A$_{(-SO4)}$
	$C^{2}_{2}(12)$	*O1-H1$_{(-COOH)}$ ···O4A$_{(-SO4)}$ & N2-H5$_{(-NH3+)}$ ···O3 $_{(-SO4)}$<br>*O1-H1$_{(-COOH)}$ ···O4A$_{(-SO4)}$ & N2-H5$_{(-NH3+)}$ ···O4$_{(-SO4)}$
	$C^{2}_{3}(12)$	N2-H6$_{(-NH3+)}$ ···O2 $_{(-COOH)}$ **ornithine** & (N2-H7$_{(-NH3+)}$ ···Cl2)$_2$
	$C^{2}_{3}(14)$	*N2-H6$_{(-NH3+)}$ ···O2$_{(-COOH)}$ **ornithine** & (N1-H2$_{(-NH3+)}$ ···Cl1)$_2$<br>*(N1-H4$_{(-NH3+)}$ ···Cl2)$_2$ & N2-H6$_{(-NH3+)}$ ···O2$_{(-COOH)}$ **ornithine**
	$C^{2}_{4}(18)$	*(N2-H6$_{(-NH3+)}$ ···Cl1)$_2$ & (N1-H2$_{(-NH3+)}$ ···Cl1)$_2$<br>*(N1-H2$_{(-NH3+)}$ ···Cl1)$_2$ & (N2-H7$_{(-NH3+)}$ ···Cl2)$_2$<br>*(N1-H4$_{(-NH3+)}$ ···Cl2) & (N2-H6$_{(-NH3+)}$ ···Cl1)$_2$<br>*(N1-H4$_{(-NH3+)}$ ···Cl2)$_2$ & (N2-H7$_{(-NH3+)}$ ···Cl2)$_2$
	$C^{3}_{3}(12)$	O1-H1$_{(-COOH)}$ ···O4$_{(-SO4)}$ & O1-H1$_{(-COOH)}$ ···O4A$_{(-SO4)}$ & C2-H8$_{(-CH)}$ ···O2$_{(-COOH)}$**ornithine**
	$C^{3}_{3}(14)$	*N2-H5$_{(-NH3+)}$ ···O3$_{(-SO4)}$ & N2-H5$_{(-NH3+)}$ ···O3A$_{(-SO4)}$ & N2-H6$_{(-NH3+)}$ ···O2$_{(-COOH)}$**ornithine**<br>*N2-H5$_{(-NH3+)}$ ···O4A$_{(-SO4)}$ & N2-H5$_{(-NH3+)}$ ···O4$_{(-SO4)}$ & N2-H6$_{(-NH3+)}$ ···O2$_{(-COOH)}$**ornithine**
	$C^{3}_{3}(16)$	*O1-H1$_{(-COOH)}$ ···O4$_{(-SO4)}$ & O1-H1$_{(-COOH)}$ ···O4A$_{(-SO4)}$ & N2-H6$_{(-NH3+)}$ ···O2$_{(-COOH)}$**ornithine**<br>*N1-H3$_{(-NH3+)}$ ···O3$_{(-SO4)}$ & N2-H6$_{(-NH3+)}$ ···O2$_{(-COOH)}$ **ornithine**
	$C^{3}_{4}(10)$	*(N1-H2$_{(-NH3+)}$ ···Cl1)$_2$ & N1-H3$_{(-NH3+)}$ ···O3$_{(-SO4)}$ & N1-H3$_{(-NH3+)}$ ···O3A$_{(-SO4)}$<br>*(N1-H4$_{(-NH3+)}$ ···Cl2)$_2$ & N1-H3$_{(-NH3+)}$ ···O3$_{(-SO4)}$ & N1-H3$_{(-NH3+)}$ ···O3A$_{(-SO4)}$<br>*N2-H5$_{(-NH3+)}$ ···O3$_{(-SO4)}$ & N2-H5$_{(-NH3+)}$ ···O3A$_{(-SO4)}$ & (N2-H6$_{(-NH3+)}$ ···Cl1)$_2$<br>*N2-H5$_{(-NH3+)}$ ···O3$_{(-SO4)}$ & N2-H5$_{(-NH3+)}$ ···O3A$_{(-SO4)}$ & (N2-H7$_{(-NH3+)}$ ···Cl2)$_2$<br>*N2-H5$_{(-NH3+)}$ ···O4$_{(-SO4)}$ & N2-H5$_{(-NH3+)}$ ···O4A$_{(-SO4)}$ & (N2-H6$_{(-NH3+)}$ ···Cl1)$_2$<br>*N2-H5$_{(-NH3+)}$ ···O4$_{(-SO4)}$& N2-H5$_{(-NH3+)}$ ···O4A$_{(-SO4)}$ & (N2-H7$_{(-NH3+)}$ ···Cl2)$_2$
	$C^{3}_{4}(16)$	*O1-H1$_{(-COOH)}$ ···O4$_{(-SO4)}$ & O1-H1$_{(-COOH)}$ ···O4A$_{(-SO4)}$ & (N1-H2$_{(-NH3+)}$ ···Cl1)$_2$<br>*O1-H1$_{(-COOH)}$ ···O4$_{(-SO4)}$ & O1-H1$_{(-COOH)}$ ···O4A$_{(-SO4)}$ & (N1-H4$_{(-NH3+)}$ ···Cl2)$_2$
	$C^{3}_{4}(20)$	*N1-H2$_{(-NH3+)}$ ···Cl1 & N2-H5$_{(-NH3+)}$ ···O3$_{(-SO4)}$ & N2-H5$_{(-NH3+)}$ ···O3A$_{(-SO4)}$<br>*N2-H5$_{(-NH3+)}$ ···O4$_{(-SO4)}$ & & (N1-H2$_{(-NH3+)}$ ···Cl1)$_2$ & N2-H5$_{(-NH3+)}$ ···O4A$_{(-SO4)}$<br>*N1-H3$_{(-NH3+)}$ ···O3$_{(-SO4)}$ & N1-H3$_{(-NH3+)}$ ···O3A $_{(-SO4)}$& N2-H6$_{(-NH3+)}$ ···Cl1<br>*N1-H3$_{(-NH3+)}$ ···O3$_{(-SO4)}$ & N1-H3$_{(-NH3+)}$ ···O3A $_{(-SO4)}$& (N2-H7$_{(-NH3+)}$ ···Cl2)$_2$<br>*(N1-H4$_{(-NH3+)}$ ···Cl2)$_2$ & N2-H5$_{(-NH3+)}$ ···O4$_{(-SO4)}$ & N2-H5$_{(-NH3+)}$ ···O4A$_{(-SO4)}$
	$C^{4}_{4}(18)$	O1-H1$_{(-COOH)}$ ···O4$_{(-SO4)}$ & O1-H1···O4A$_{(-SO4)}$ & (N1-H3$_{(-NH3+)}$ ···O3$_{(-SO4)}$)$_2$
	$R^{2}_{1}(4)$	N2-H5$_{(-NH3+)}$ ···O3$_{(-SO4)}$ & N2-H5$_{(-NH3+)}$ ···O4A$_{(-SO4)}$
	$R^{2}_{4}(8)$	*N1-H2$_{(-NH3+)}$ ···Cl1 & (N1-H4$_{(-NH3+)}$ ···Cl2)$_2$<br>*(N2-H6$_{(-NH3+)}$ ···Cl1)$_2$ & (N2-H7$_{(-NH3+)}$ ···Cl2)$_2$
	$R^{3}_{3}(12)$	O1-H1$_{(-COOH)}$ ···O4$_{(-SO4)}$ & O1-H1$_{(-COOH)}$ ···O4A$_{(-SO4)}$ & C4-H12$_{(-CH2)}$ ···O1$_{(-COOH)}$ **ornithine**
	$R^{4}_{4}(18)$	O1-H1$_{(-COOH)}$ ···O4$_{(-SO4)}$ & O1-H1$_{(-COOH)}$ ···O4A$_{(-SO4)}$ & C3-H10$_{(-CH2)}$ ···O3$_{(-SO4)}$& C3-H10$_{(-CH2)}$ ···O3A$_{(-SO4)}$
	$D^{2}_{2}(5)$	*N1-H2$_{(-NH3+)}$ ···Cl1 & N1-H3$_{(-NH3+)}$ ···O3$_{(-SO4)}$<br>*(N1-H2$_{(-NH3+)}$ ···Cl1)$_2$ & N1-H4$_{(-NH3+)}$ ···Cl2<br>*N1-H3$_{(-NH3+)}$ ···O3$_{(-SO4)}$ & N2-H7$_{(-NH3+)}$ ···Cl2<br>*N2-H6$_{(-NH3+)}$ ···Cl1 & N2-H5$_{(-NH3+)}$ ···O3$_{(-SO4)}$<br>*N2-H7$_{(-NH3+)}$ ···Cl2 & N2-H5$_{(-NH3+)}$ ···O3$_{(-SO4)}$<br>*N2-H6$_{(-NH3+)}$ ···Cl1 & N2-H5$_{(-NH3+)}$ ···O4A$_{(-SO4)}$<br>*N2-H7$_{(-NH3+)}$ ···Cl2 & N2-H5$_{(-NH3+)}$ ···O4A$_{(-SO4)}$<br>*N2-H6$_{(-NH3+)}$ ···Cl1 & (N2-H7$_{(-NH3+)}$ ···Cl2)$_2$

	$D^2_2(8)$	*O1-H1$_{(-COOH)}$ ···O4A$_{(-SO4)}$ & N1-H2$_{(-NH3+)}$ ···Cl1<br>*O1-H1$_{(-COOH)}$ ···O4A$_{(-SO4)}$& N1-H4$_{(-NH3+)}$ ···Cl2
	$D^2_2(10)$	*N2-H5$_{(-NH3+)}$ ···O3$_{(-SO4)}$ & N1-H2$_{(-NH3+)}$ ···Cl1<br>*N1-H2$_{(-NH3+)}$ ···Cl1 & N2-H5$_{(-NH3+)}$ ···O4A$_{(-SO4)}$<br>*N1-H2$_{(-NH3+)}$ ···Cl1 & N2-H7$_{(-NH3+)}$ ···Cl2<br>*N1-H3$_{(-NH3+)}$ ···O3$_{(-SO4)}$ & N2-H6$_{(-NH3+)}$ ···Cl1<br>*N1-H3$_{(-NH3+)}$ ···O3$_{(-SO4)}$ & N2-H7$_{(-NH3+)}$ ···Cl2<br>*N1-H4$_{(-NH3+)}$ ···Cl2 & N2-H5$_{(-NH3+)}$ ···O4A$_{(-SO4)}$<br>*N1-H4$_{(-NH3+)}$ ···Cl2 & N2-H6$_{(-NH3+)}$ ···Cl1
	$D^2_2(11)$	*O1-H1$_{(-COOH)}$ ···O4A$_{(-SO4)}$ & N2-H6$_{(-NH3+)}$ ···Cl1<br>*O1-H1$_{(-COOH)}$ ···O4A$_{(-SO4)}$ & N2-H7$_{(-NH3+)}$ ···Cl2
	$D^2_3(6)$	*(N1-H2$_{(-NH3+)}$ ···Cl1)$_2$ & N1-H3$_{(-NH3+)}$ ···O3$_{(-SO4)}$<br>*(N1-H2$_{(-NH3+)}$ ···Cl1)$_2$ & N1-H4$_{(-NH3+)}$ ···Cl2<br>*N1-H2$_{(-NH3+)}$ ···Cl1 & (N1-H4$_{(-NH3+)}$ ···Cl2)$_2$<br>*N1-H3$_{(-NH3+)}$ ···O3A$_{(-SO4)}$ & (N1-H4$_{(-NH3+)}$ ···Cl2)$_2$<br>*N2-H5$_{(-NH3+)}$ ···O3A$_{(-SO4)}$ & (N2-H6$_{(-NH3+)}$ ···Cl1)$_2$<br>*N2-H5$_{(-NH3+)}$ ···O3A$_{(-SO4)}$ & (N2-H7$_{(-NH3+)}$ ···Cl2)$_2$<br>*N2-H5$_{(-NH3+)}$ ···O4$_{(-SO4)}$ & (N2-H6$_{(-NH3+)}$ ···Cl1)$_2$<br>*N2-H5$_{(-NH3+)}$ ···O4$_{(-SO4)}$ & (N2-H7$_{(-NH3+)}$ ···Cl2)$_2$<br>*(N2-H6$_{(-NH3+)}$ ···Cl1)$_2$ & (N2-H7$_{(-NH3+)}$ ···Cl2)$_2$
	$D^2_3(8)$	*N2-H5$_{(-NH3+)}$ ···O3$_{(-SO4)}$ & N2-H5$_{(-NH3+)}$ ···O3A$_{(-SO4)}$ & (N2-H6$_{(-NH3+)}$ ···Cl1)$_2$<br>*N2-H5$_{(-NH3+)}$ ···O3$_{(-SO4)}$ & N2-H5$_{(-NH3+)}$ ···O3A$_{(-SO4)}$ & N2-H7$_{(-NH3+)}$ ···Cl2
	$D^2_3(9)$	*(N1-H2$_{(-NH3+)}$ ···Cl1)$_2$<br>*O1-H1$_{(-COOH)}$ ···O4$_{(-SO4)}$ & N1-H4$_{(-NH3+)}$ ···Cl2
	$D^2_3(11)$	*(N1-H2$_{(-NH3+)}$ ···Cl1)$_2$ & N2-H5$_{(-NH3+)}$ ···O3A$_{(-SO4)}$<br>*(N1-H2$_{(-NH3+)}$ ···Cl1)$_2$ & N2-H5$_{(-NH3+)}$ ···O4$_{(-SO4)}$<br>*N2-H7$_{(-NH3+)}$ ···Cl2 & (N1-H2$_{(-NH3+)}$ ···Cl1)$_2$<br>*N1-H3$_{(-NH3+)}$ ···O3A$_{(-SO4)}$ & (N2-H6$_{(-NH3+)}$ ···Cl1)$_2$<br>*N1-H3$_{(-NH3+)}$ ···O3A$_{(-SO4)}$ & (N2-H7$_{(-NH3+)}$ ···Cl2)$_2$<br>*(N1-H4$_{(-NH3+)}$ ···Cl2)$_2$ & N2-H5$_{(-NH3+)}$ ···O4$_{(-SO4)}$<br>*(N1-H4$_{(-NH3+)}$ ···Cl2)$_2$ & N2-H6$_{(-NH3+)}$ ···Cl1
	$D^2_3(12)$	*(N2-H6$_{(-NH3+)}$ ···Cl1)$_2$<br>*(N2-H7$_{(-NH3+)}$ ···Cl2)$_2$
	$D^3_3(8)$	*N1-H2$_{(-NH3+)}$ ···Cl1 & N1-H3$_{(-NH3+)}$ ···O3$_{(-SO4)}$ & N1-H3$_{(-NH3+)}$ ···O3A$_{(-SO4)}$<br>*N1-H3$_{(-NH3+)}$ ···O3$_{(-SO4)}$ & N1-H3$_{(-NH3+)}$ ···O3A$_{(-SO4)}$ & N1-H4$_{(-NH3+)}$ ···Cl2<br>*N2-H5$_{(-NH3+)}$ ···O4A$_{(-SO4)}$ & N2-H5$_{(-NH3+)}$ ···O4$_{(-SO4)}$ & N2-H6$_{(-NH3+)}$ ···Cl1<br>*N2-H5$_{(-NH3+)}$ ···O4$_{(-SO4)}$ & N2-H5$_{(-NH3+)}$ ···O4A$_{(-SO4)}$ & N2-H7$_{(-NH3+)}$ ···Cl2
	$D^3_3(11)$	*O1-H1$_{(-COOH)}$ ···O4A$_{(-SO4)}$ & O1-H1$_{(-COOH)}$ ···O4$_{(-SO4)}$ & N1-H2$_{(-NH3+)}$ ···Cl1<br>*O1-H1$_{(-COOH)}$ ···O4$_{(-SO4)}$ & O1-H1$_{(-COOH)}$ ···O4A$_{(-SO4)}$ & N1-H4$_{(-NH3+)}$ ···Cl2
	$D^3_3(13)$	*N1-H2$_{(-NH3+)}$ ···Cl1 & N2-H5$_{(-NH3+)}$ ···O3$_{(-SO4)}$ & N2-H5$_{(-NH3+)}$ ···O3A$_{(-SO4)}$<br>*N1-H2$_{(-NH3+)}$ ···Cl1 & N2-H5$_{(-NH3+)}$ ···O4$_{(-SO4)}$ & N2-H5$_{(-NH3+)}$ ···O4A$_{(-SO4)}$<br>*N1-H3$_{(-NH3+)}$ ···O3$_{(-SO4)}$ & N1-H3$_{(-NH3+)}$ ···O3A$_{(-SO4)}$ & N2-H6$_{(-NH3+)}$ ···Cl1<br>*N1-H3$_{(-NH3+)}$ ···O3$_{(-SO4)}$ & N1-H3$_{(-NH3+)}$ ···O3A$_{(-SO4)}$ & N2-H7$_{(-NH3+)}$ ···Cl2<br>*N2-H5$_{(-NH3+)}$ ···O4$_{(-SO4)}$ & N1-H4$_{(-NH3+)}$ ···Cl2 & N2-H5$_{(-NH3+)}$ ···O4A$_{(-SO4)}$
	$D^3_3(14)$	*O1-H1$_{(-COOH)}$ ···O4$_{(-SO4)}$ & O1-H1$_{(-COOH)}$ ···O4A$_{(-SO4)}$ & N1-H6$_{(-NH3+)}$ ···Cl1<br>*O1-H1$_{(-COOH)}$ ···O4$_{(-SO4)}$ & O1-H1$_{(-COOH)}$ ···O4A$_{(-SO4)}$ & (N2-H7$_{(-NH3+)}$ ···Cl2)$_2$
**BAPKUB**	$D(2)$	*O1-H1$_{(-COOH)}$ ···O4A$_{(-SO4)}$<br>*N1-H2$_{(-NH3+)}$ ···Br1<br>*N1-H3$_{(-NH3+)}$ ···O3$_{(-SO4)}$<br>*N1-H4$_{(-NH3+)}$ ···Br2<br>*N2-H5$_{(-NH3+)}$ ···O3$_{(-SO4)}$<br>*N2-H5$_{(-NH3+)}$ ···O4A$_{(-SO4)}$<br>*N2-H6$_{(-NH3+)}$ ···Br1<br>*N2-H7$_{(-NH3+)}$ ···Br2
	$D^1_2(3)$	*(N1-H2$_{(-NH3+)}$ ···Br1)$_2$<br>*(N1-H4$_{(-NH3+)}$ ···Br2)$_2$<br>*(N2-H6$_{(-NH3+)}$ ···Br1)$_2$<br>*(N2-H7$_{(-NH3+)}$ ···Br2)$_2$
	$D^2_2(5)$	*O1-H1$_{(-COOH)}$ ···O4A$_{(-SO4)}$ & O1-H3$_{(-COOH)}$ ···O3$_{(-SO4)}$<br>*N1-H3$_{(-NH3+)}$ ···O3$_{(-SO4)}$ & N1-H3$_{(-NH3+)}$ ···O3A$_{(-SO4)}$<br>*N2-H5$_{(-NH3+)}$ ···O3A$_{(-SO4)}$ & N2-H5$_{(-NH3+)}$ ···O3$_{(-SO4)}$<br>*N2-H5$_{(-NH3+)}$ ···O4A$_{(-SO4)}$ & N2-H5$_{(-NH3+)}$ ···O4$_{(-SO4)}$
	$R^2_2(12)$	(C4-H12···O1$_{(-COOH)}$)$_2$ **ornithine**
level 2	$D^2_2(8)$	*O1-H1$_{(-COOH)}$ ···O4A$_{(-SO4)}$ & N1-H2$_{(-NH3+)}$ ···Br1<br>*O1-H1$_{(-COOH)}$ ···O4A$_{(-SO4)}$ & N1-H4$_{(-NH3+)}$ ···Br2

	$D^2_3(9)$	*O1-H1$_{(-COOH)}$ ···O4A$_{(-SO4)}$ & N1-H2$_{(-NH3+)}$ ···Br1<br>*O1-H1$_{(-COOH)}$ ···O4$_{(-SO4)}$ & (N1-H4$_{(-NH3+)}$ ···Br)$_2$
	$D^3_3(11)$	*O1-H1$_{(-COOH)}$ ···O4A$_{(-SO4)}$ & (N1-H2$_{(-NH3+)}$ ···Br1)$_2$<br>*O1-H1$_{(-COOH)}$ ···O4$_{(-SO4)}$ & O1-H1$_{(-COOH)}$ ···O4A$_{(-SO4)}$ & N1-H4$_{(-NH3+)}$ ···Br2
	$C^3_4(16)$	*O1-H1$_{(-COOH)}$ ···O4$_{(-SO4)}$ & O1-H1$_{(-COOH)}$ ···O4A$_{(-SO4)}$ & (N1-H2$_{(-NH3+)}$ ···Br1)$_2$<br>*O1-H1$_{(-COOH)}$ ···O4$_{(-SO4)}$ & O1-H1$_{(-COOH)}$ ···O4A$_{(-SO4)}$ & (N1-H4$_{(-NH3+)}$ ···Br2)$_2$
	$C^2_2(9)$	O1-H1$_{(-COOH)}$ ···O4A$_{(-SO4)}$ & N1-H3$_{(-NH3+)}$ ···O3$_{(-SO4)}$
	$C^4_4(18)$	O1-H1$_{(-COOH)}$ ···O4$_{(-SO4)}$ & O1-H1$_{(-COOH)}$ ···O4$_{(-SO4)}$ & N1-H3$_{(-NH3+)}$ ···O3$_{(-SO4)}$ & N1-H3$_{(-NH3+)}$ ···O3A$_{(-SO4)}$
	$C^2_2(12)$	O1-H1$_{(-COOH)}$ ···O4A$_{(-SO4)}$ & N2-H5$_{(-NH3+)}$ ···O3$_{(-SO4)}$
	$C^1_2(10)$	O1-H1$_{(-COOH)}$ ···O4A$_{(-SO4)}$ & N2-H5$_{(-NH3+)}$ ···O4A$_{(-SO4)}$
	$C^2_2(12)$	O1-H1$_{(-COOH)}$ ···O4A$_{(-SO4)}$ & N2-H5$_{(-NH3+)}$ ···O4$_{(-SO4)}$
	$D^2_2(11)$	*O1-H1$_{(-COOH)}$ ···O4A$_{(-SO4)}$ & N2-H6$_{(-NH3+)}$ ···Br1<br>*O1-H1$_{(-COOH)}$ ···O4A$_{(-SO4)}$ & N2-H6$_{(-NH3+)}$ ···Br1<br>*O1-H1$_{(-COOH)}$ ···O4A$_{(-SO4)}$ & N2-H7$_{(-NH3+)}$ ···Br2
	$D^2_3(12)$	*O1-H1$_{(-COOH)}$ ···O4$_{(-SO4)}$ & (N2-H6$_{(-NH3+)}$ ···Br1)$_2$<br>*(N2-H7$_{(-NH3+)}$ ···Br2)$_2$
	$D^3_3(14)$	O1-H1$_{(-COOH)}$··O4$_{(-SO4)}$ & O1-H1$_{(-COOH)}$ ···O4A$_{(-SO4)}$ & N2-H6$_{(-NH3+)}$ ···Br1
	$D^2_2(5)$	*N1-H3$_{(-NH3+)}$ ···O3$_{(-SO4)}$ & N1-H2$_{(-NH3+)}$ ···Br2<br>*N1-H2$_{(-NH3+)}$ ···Br1 & N1-H4$_{(-NH3+)}$ ···Br2<br>*N2-H5$_{(-NH3+)}$ ···O3$_{(-SO4)}$ & N2-H6$_{(-NH3+)}$ ···Br1<br>*N2-H5$_{(-NH3+)}$ ···O3$_{(-SO4)}$ & N2-H7$_{(-NH3+)}$ ···Br2<br>*N2-H5$_{(-NH3+)}$ ···O4A$_{(-SO4)}$ & N2-H6$_{(-NH3+)}$ ···Br1<br>*N1-H3$_{(-NH3+)}$ ···O3$_{(-SO4)}$ & N1-H4$_{(-NH3+)}$ ···Br2<br>*N2-H5$_{(-NH3+)}$ ···O4A$_{(-SO4)}$ & N2-H7$_{(-NH3+)}$ ···Br2<br>*N2-H6$_{(-NH3+)}$ ···Br1 & N2-H7$_{(-NH3+)}$ ···Br2
	$C^3_4(20)$	*N1-H3$_{(-NH3+)}$ ···O3A$_{(-SO4)}$ & (N2-H7$_{(-NH3+)}$ ···Br2)$_2$<br>*N2-H5$_{(-NH3+)}$ ···O4$_{(-SO4)}$ & (N1-H4$_{(-NH3+)}$ ···Br2)$_2$
	$D^2_2(10)$	*N1-H4$_{(-NH3+)}$ ···Br2 & N2-H5$_{(-NH3+)}$ ···O3$_{(-SO4)}$<br>*N2-H5$_{(-NH3+)}$ ···O4A$_{(-SO4)}$ & (N1-H4$_{(-NH3+)}$ ···Br2)$_2$<br>*N2-H6$_{(-NH3+)}$ ···Br1 & N1-H4$_{(-NH3+)}$ ···Br2<br>*N1-H4$_{(-NH3+)}$ ···Br2 & (N1-H2$_{(-NH3+)}$ ···Br1)$_2$<br>*N2-H6$_{(-NH3+)}$ ···Br1 & N2-H7$_{(-NH3+)}$ ···Br2<br>*N1-H3$_{(-NH3+)}$ ···O3$_{(-SO4)}$ & N2-H6$_{(-NH3+)}$ ···Br1<br>*N2-H7$_{(-NH3+)}$ ···Br2 & (N2-H6$_{(-NH3+)}$ ···Br1)$_2$ & N1-H3$_{(-NH3+)}$ ···O3$_{(-SO4)}$
	$D^2_3(11)$	*(N1-H4$_{(-NH3+)}$ ···Br2)$_2$ & N2-H5$_{(-NH3+)}$ ···O3A$_{(-SO4)}$<br>*(N1-H4$_{(-NH3+)}$ ···Br2)$_2$ & N2-H6$_{(-NH3+)}$ ···Br1<br>*(N1-H2$_{(-NH3+)}$ ···Br1)$_2$ & N2-H5$_{(-NH3+)}$ ···O3A$_{(-SO4)}$<br>*(N1-H2$_{(-NH3+)}$ ···Br1)$_2$ & N2-H7$_{(-NH3+)}$ ···Br2<br>*N1-H3$_{(-NH3+)}$ ···O3$_{(-SO4)}$ & (N2-H7$_{(-NH3+)}$ ···Br2)$_2$
	$D^3_3(13)$	*N2-H5$_{(-NH3+)}$ ···O3$_{(-SO4)}$ & N2-H5$_{(-NH3+)}$ ···O3A$_{(-SO4)}$ & N1-H4$_{(-NH3+)}$ ···Br2<br>*N2-H5$_{(-NH3+)}$ ···O4$_{(-SO4)}$ & N2-H5$_{(-NH3+)}$ ···O4A$_{(-SO4)}$<br>*N1-H3$_{(-NH3+)}$ ···O3A$_{(-SO4)}$ & N1-H3$_{(-NH3+)}$ ···O3$_{(-SO4)}$ & (N2-H6$_{(-NH3+)}$ ···Br1)$_2$<br>*N1-H3$_{(-NH3+)}$ ···O3$_{(-SO4)}$ & N2-H7$_{(-NH3+)}$ ···Br2 & N1-H3$_{(-NH3+)}$ ···O3A$_{(-SO4)}$
	$C^3_4(20)$	*N2-H5$_{(-NH3+)}$ ···O3$_{(-SO4)}$ & N2-H5$_{(-NH3+)}$ ···O3A$_{(-SO4)}$ & (N1-H4$_{(-NH3+)}$ ···Br2)$_2$<br>*(N2-H5$_{(-NH3+)}$ ···O3$_{(-SO4)}$)$_2$ & (N1-H2$_{(-NH3+)}$ ···Br1)$_2$<br>*N1-H3$_{(-NH3+)}$ ···O3A$_{(-SO4)}$ & N1-H3$_{(-NH3+)}$ ···O3$_{(-SO4)}$ & (N2-H6$_{(-NH3+)}$ ···Br1)$_2$
	$D^2_3(11)$	*(N1-H4$_{(-NH3+)}$ ···Br2)$_2$ & N2-H5$_{(-NH3+)}$ ···O4$_{(-SO4)}$<br>*N1-H3$_{(-NH3+)}$ ···O3A$_{(-SO4)}$ & (N2-H6$_{(-NH3+)}$ ···Br1)$_2$
	$C^2_4(18)$	*(N1-H4$_{(-NH3+)}$ ···Br2)$_2$ & (N2-H6$_{(-NH3+)}$ ···Br1)$_2$<br>*(N1-H4$_{(-NH3+)}$ ···Br2)$_2$ & (N2-H7$_{(-NH3+)}$ ···Br2)$_2$
	$C^1_2(9)$	*N1-H4$_{(-NH3+)}$··Br2 & N2-H7$_{(-NH3+)}$ ···Br2<br>*N1-H2$_{(-NH3+)}$ ···Br1 & N2-H6$_{(-NH3+)}$ ···Br1<br>*N1-H3$_{(-NH3+)}$ ···O3$_{(-SO4)}$ & N2-H5$_{(-NH3+)}$ ···O3$_{(-SO4)}$
	$R^2_1(4)$	N2-H5$_{(-NH3+)}$ ···O3$_{(-SO4)}$ & N2-H5$_{(-NH3+)}$ ···O4A$_{(-SO4)}$
	$D^2_3(6)$	*N2-H5$_{(-NH3+)}$ ···O3A$_{(-SO4)}$ & (N2-H6$_{(-NH3+)}$ ···Br1)$_2$<br>*(N2-H7$_{(-NH3+)}$ ···Br2)$_2$ & N2-H5$_{(-NH3+)}$ ···O3A$_{(-SO4)}$<br>*N2-H5$_{(-NH3+)}$ ···O4$_{(-SO4)}$ & (N2-H6$_{(-NH3+)}$ ···Br1)$_2$<br>*N1-H3$_{(-NH3+)}$ ···O3A$_{(-SO4)}$ & (N1-H2$_{(-NH3+)}$ ···Br1)$_2$<br>*(N1-H2$_{(-NH3+)}$ ···Br1)$_2$ & N1-H4$_{(-NH3+)}$ ···Br2<br>*(N1-H2$_{(-NH3+)}$ ···Br1)$_2$ & (N1-H4$_{(-NH3+)}$ ···Br2)$_2$<br>*N1-H3$_{(-NH3+)}$ ···O3A$_{(-SO4)}$ & (N1-H4$_{(-NH3+)}$ ···Br2)$_2$<br>*N2-H5$_{(-NH3+)}$ ···O4$_{(-SO4)}$ & (N2-H7$_{(-NH3+)}$ ···Br2)$_2$<br>*N2-H7$_{(-NH3+)}$ ···Br2 & (N2-H6$_{(-NH3+)}$ ···Br1)$_2$
	$D^2_3(8)$	*N2-H5$_{(-NH3+)}$ ···O3$_{(-SO4)}$ & N2-H5$_{(-NH3+)}$ ···O3A$_{(-SO4)}$ & N2-H6$_{(-NH3+)}$ ···Br1<br>*N2-H5$_{(-NH3+)}$ ···O3$_{(-SO4)}$ & N2-H5$_{(-NH3+)}$ ···O3A$_{(-SO4)}$ & N2-H7$_{(-NH3+)}$ ···Br2

	$C^3_4(10)$	*N2-H5$_{(-NH3+)}$ ···O3$_{(-SO4)}$ & N2-H5$_{(-NH3+)}$ ···O3A$_{(-SO4)}$ & (N2-H6$_{(-NH3+)}$ ···Br1)$_2$<br>*N2-H5$_{(-NH3+)}$ ···O3$_{(-SO4)}$ & N2-H5$_{(-NH3+)}$ ···O3A$_{(-SO4)}$ & (N2-H7$_{(-NH3+)}$ ···Br2)$_2$<br>*N2-H5$_{(-NH3+)}$ ···O4A$_{(-SO4)}$ & N2-H5$_{(-NH3+)}$ ···O4$_{(-SO4)}$ & (N2-H6$_{(-NH3+)}$ ···Br1)$_2$<br>*N1-H3$_{(-NH3+)}$ ···O3$_{(-SO4)}$ & N1-H3$_{(-NH3+)}$ ···O3A$_{(-SO4)}$ & (N1-H2$_{(-NH3+)}$ ···Br1)$_2$<br>*N1-H3$_{(-NH3+)}$ ···O3$_{(-SO4)}$ & N1-H3$_{(-NH3+)}$ ···O3A$_{(-SO4)}$ & (N1-H4$_{(-NH3+)}$ ···Br2)$_2$
	$D^3_3(8)$	*N2-H5$_{(-NH3+)}$ ···O4$_{(-SO4)}$ & N2-H5$_{(-NH3+)}$ ···O4A$_{(-SO4)}$ & N2-H6$_{(-NH3+)}$ ···Br1<br>*N1-H3$_{(-NH3+)}$ ···O3$_{(-SO4)}$ & N1-H3$_{(-NH3+)}$ ···O3A$_{(-SO4)}$ & N1-H2$_{(-NH3+)}$ ···Br1<br>*N1-H3$_{(-NH3+)}$ ···O3$_{(-SO4)}$ & N1-H3$_{(-NH3+)}$ ···O3A$_{(-SO4)}$ & N1-H4$_{(-NH3+)}$ ···Br2<br>*N2-H5$_{(-NH3+)}$ ···O4A$_{(-SO4)}$ & N2-H5$_{(-NH3+)}$ ···O4$_{(-SO4)}$ & (N2-H7$_{(-NH3+)}$ ···Br2)$_2$
	$R^2_4(8)$	*(N1-H2$_{(-NH3+)}$ ···Br1)$_2$ & (N1-H4$_{(-NH3+)}$ ···Br2)$_2$<br>*(N2-H6$_{(-NH3+)}$ ···Br1)$_2$ & (N2-H7$_{(-NH3+)}$ ···Br2)$_2$
	$D^3_3(13)$	N2-H5$_{(-NH3+)}$ ···O3$_{(-SO4)}$ & N2-H5$_{(-NH3+)}$ ···O3A & N1-H2$_{(-NH3+)}$ ···Br1
	$C^2_4(18)$	*N2-H5$_{(-NH3+)}$ ···O3$_{(-SO4)}$ & (N2-H6$_{(-NH3+)}$ ···Br1)$_2$<br>*(N1-H2$_{(-NH3+)}$ ···Br1)$_2$ & (N2-H7$_{(-NH3+)}$ ···Br2)$_2$
	$C^2_2(11)$	N1-H3$_{(-NH3+)}$ ···O3$_{(-SO4)}$ & N2-H5$_{(-NH3+)}$ ···O4A$_{(-SO4)}$
	$C^3_4(10)$	N2-H5$_{(-NH3+)}$ ···O4A$_{(-SO4)}$ & N2-H5$_{(-NH3+)}$ ···O4$_{(-SO4)}$ & (N2-H7$_{(-NH3+)}$ ···Br2)$_2$
**BEZQOO**	$C(5)$	N1-H4$_{(-NH3+)}$ ···O2$_{(-COOH)}$ **ornithine**
	$C^1_2(4)$	*N2-H12$_{(-NH3+)}$ ···F2 & N2-H13$_{(-NH3+)}$ ···F2<br>*N2-H14$_{(-NH3+)}$ ···F2 & N2-H12$_{(-NH3+)}$ ···F2<br>*N2-H14$_{(-NH3+)}$ ···F3 & N2-H12$_{(-NH3+)}$ ···F3
	$C^1_2(7)$	N1-H3$_{(-NH3+)}$ ···F1 & C4-H9$_{(-CH2)}$ ···F1
	$C^1_2(9)$	N1-H3$_{(-NH3+)}$ ···F1 & N2-H14$_{(-NH3+)}$ ···F1
	$C^1_2(10)$	*O1-H1$_{(-COOH)}$ ···F6 & N2-H12$_{(-NH3+)}$ ···F6<br>*O1-H1$_{(-COOH)}$ ···F6 & N2-H12$_{(-NH3+)}$ ···F3
	$C^2_2(5)$	N1-H5$_{(-NH3+)}$ ···O3$_{(-OH2)}$ & C2-H2$_{(-CH)}$ ···O3$_{(-OH2)}$
	$C^2_2(6)$	*N1-H3$_{(-NH3+)}$ ···F1 & N1-H4$_{(-NH3+)}$ ···F5<br>*N2-H13$_{(-NH3+)}$ ···F4 & N2-H12$_{(-NH3+)}$ ···F2<br>*N2-H14$_{(-NH3+)}$ ···F3 & N2-H12$_{(-NH3+)}$ ···F2<br>*N2-H14$_{(-NH3+)}$ ···F1 & N2-H12$_{(-NH3+)}$ ···F2<br>*N2-H12$_{(-NH3+)}$ ···F3 & N2-H13$_{(-NH3+)}$ ···F2<br>*N2-H13$_{(-NH3+)}$ ···F2 & N2-H14$_{(-NH3+)}$ ···F1
	$C^2_2(8)$	N2-H12$_{(-NH3+)}$ ···F2 & C4-H9$_{(-CH2)}$ ···F1
	$C^2_2(9)$	*N1-H3$_{(-NH3+)}$ ···F1 & O1-H1$_{(-COOH)}$ ···F6<br>*N1-H5$_{(-NH3+)}$ ···F5 & O1-H1$_{(-COOH)}$ ···F6<br>*N1-H4$_{(-NH3+)}$ ···F5 & C4-H9$_{(-CH2)}$ ···F1
	$C^2_2(10)$	O1-H1$_{(-COOH)}$ ···F6 & C4-H9$_{(-CH2)}$ ···F1
	$C^2_2(11)$	*N1-H3$_{(-NH3+)}$ ···F1 & N2-H12$_{(-NH3+)}$ ···F2<br>*N1-H3$_{(-NH3+)}$ ···F1 & N2-H12$_{(-NH3+)}$ ···F3<br>*N1-H3$_{(-NH3+)}$ ···F1 & N2-H12$_{(-NH3+)}$ ···F6<br>*N1-H3$_{(-NH3+)}$ ···F1 & N2-H13$_{(-NH3+)}$ ···F2<br>*N1-H3$_{(-NH3+)}$ ···F1 & N2-H13$_{(-NH3+)}$ ···F4<br>*N1-H3$_{(-NH3+)}$ ···F1 & N2-H14$_{(-NH3+)}$ ···F2<br>*N1-H3$_{(-NH3+)}$ ···F1 & N2-H14$_{(-NH3+)}$ ···F3<br>*N1-H4$_{(-NH3+)}$ ···F5 & N2-H12$_{(-NH3+)}$ ···F2<br>*N1-H4$_{(-NH3+)}$ ···F5 & N2-H12$_{(-NH3+)}$ ···F3<br>*N1-H4$_{(-NH3+)}$ ···F5 & N2-H12$_{(-NH3+)}$ ···F6<br>*N1-H4$_{(-NH3+)}$ ···F5 & N2-H13$_{(-NH3+)}$ ···F2<br>*N1-H4$_{(-NH3+)}$ ···F5 & N2-H13$_{(-NH3+)}$ ···F4<br>*N1-H4$_{(-NH3+)}$ ···F5 & N2-H14$_{(-NH3+)}$ ···F1<br>*N1-H4$_{(-NH3+)}$ ···F5 & N2-H14$_{(-NH3+)}$ ···F2<br>*N1-H4$_{(-NH3+)}$ ···F5 & N2-H14$_{(-NH3+)}$ ···F3
	$C^2_2(12)$	*O1-H1$_{(-COOH)}$ ···F6 & N2-H12$_{(-NH3+)}$ ···F2<br>*O1-H1$_{(-COOH)}$ ···F6 & N2-H12$_{(-NH3+)}$ ···F3<br>*O1-H1$_{(-COOH)}$ ···F6 & N2-H12$_{(-NH3+)}$ ···F4<br>*O1-H1$_{(-COOH)}$ ···F6 & N2-H14$_{(-NH3+)}$ ···F1<br>*O1-H1$_{(-COOH)}$ ···F6 & N2-H14$_{(-NH3+)}$ ···F2<br>*O1-H1$_{(-COOH)}$ ···F6 & N2-H14$_{(-NH3+)}$ ···F3
	$R^1_2(6)$	N2-H14$_{(-NH3+)}$ ···F1 & C4-H9$_{(-CH2)}$ ···F1
	$R^2_1(4)$	*N2-H12$_{(-NH3+)}$ ···F3 & N2-H12$_{(-NH3+)}$ ···F2<br>*N2-H12$_{(-NH3+)}$ ···F2 & N2-H12$_{(-NH3+)}$ ···F6<br>*N2-H12$_{(-NH3+)}$ ···O3$_{(-OH2)}$ & N2-H12$_{(-NH3+)}$ ···F6<br>*N2-H13$_{(-NH3+)}$ ···F2 & N2-H13$_{(-NH3+)}$ ···F4
	$R^2_2(8)$	N2-H14$_{(-NH3+)}$ ···F2 & C4-H9$_{(-CH2)}$ ···F1
	$D(2)$	*O1-H1$_{(-COOH)}$ ···F6<br>*O3-H15$_{(-OH2)}$···F5

		*O3-H16$_{(-OH2)}$···F1<br>*C2-H2$_{(-CH)}$ ···O3$_{(-OH2)}$<br>*N1-H3$_{(-NH3+)}$ ···F1<br>*N2-H12$_{(-NH3+)}$ ···F2<br>*N2-H12$_{(-NH3+)}$ ···F3<br>*N1-H4$_{(-NH3+)}$ ···F5<br>*N2-H13$_{(-NH3+)}$ ···F4<br>*N2-H12$_{(-NH3+)}$ ···F6<br>*C4-H9$_{(-CH2)}$ ···F1<br>*N2-H14$_{(-NH3+)}$ ···F1<br>*N2-H14$_{(-NH3+)}$ ···F2<br>*N2-H14$_{(-NH3+)}$ ···F3
	$D^{1}_{2}(3)$	*O3-H16$_{(-OH2)}$···F1 & N1-H3$_{(-NH3+)}$ ···F1<br>*O3-H15$_{(-OH2)}$···F5 & N1-H4$_{(-NH3+)}$ ···F5<br>*O3-H16$_{(-OH2)}$···F1 & C4-H9$_{(-CH2)}$ ···F1<br>*O3-H16$_{(-OH2)}$···F1 & N2-H14$_{(-NH3+)}$ ···F1
	$D^{2}_{2}(4)$	*O3-H15$_{(-OH2)}$···F5 & C2-H2$_{(-CH)}$ ···O3$_{(-OH2)}$<br>*O3-H16$_{(-OH2)}$···F1 & C2-H2$_{(-CH)}$ ···O3$_{(-OH2)}$<br>*O3-H15$_{(-OH2)}$···F5 & N1-H5$_{(-NH3+)}$ ···O3$_{(-OH2)}$
	$D^{2}_{2}(5)$	*O1-H1$_{(-COOH)}$ ···F6 & O3-H15$_{(-OH2)}$···F5<br>*O1-H1$_{(-COOH)}$ ···F6 & O3-H16$_{(-OH2)}$···F1<br>*N1-H5$_{(-NH3+)}$ ···O3$_{(-OH2)}$ & N1-H3$_{(-NH3+)}$ ···F1<br>*O3-H15$_{(-OH2)}$···F5 & N1-H3$_{(-NH3+)}$ ···F1<br>*N1-H5$_{(-NH3+)}$ ···O3$_{(-OH2)}$ & N1-H4$_{(-NH3+)}$ ···F5<br>*N1-H4$_{(-NH3+)}$ ···F5 & O3-H16$_{(-OH2)}$···F1<br>*O3-H15$_{(-OH2)}$···F5 & C4-H9$_{(-CH2)}$ ···F1<br>*O3-H15$_{(-OH2)}$···F5 & N2-H12$_{(-NH3+)}$ ···F3
	$D^{2}_{2}(6)$	*N1-H3$_{(-NH3+)}$ ···F1 & C2-H2$_{(-CH)}$ ···O3$_{(-OH2)}$<br>*N1-H4$_{(-NH3+)}$ ···F5 & C2-H2$_{(-CH)}$ ···O3$_{(-OH2)}$
	$D^{2}_{2}(7)$	*C2-H2$_{(-CH)}$ ···O3$_{(-OH2)}$ & C4-H9$_{(-CH2)}$ ···F1
	$D^{2}_{2}(8)$	O1-H1$_{(-COOH)}$ ···F6 & N1-H5$_{(-NH3+)}$ ···O3$_{(-OH2)}$
	$D^{2}_{2}(9)$	*N2-H12$_{(-NH3+)}$ ···F2 & C2-H2$_{(-CH)}$ ···O3$_{(-OH2)}$<br>*N2-H12$_{(-NH3+)}$ ···F3 & C2-H2$_{(-CH)}$ ···O3$_{(-OH2)}$<br>*N2-H12$_{(-NH3+)}$ ···F6 & C2-H2$_{(-CH)}$ ···O3$_{(-OH2)}$<br>*N2-H13$_{(-NH3+)}$ ···F2 & C2-H2$_{(-CH)}$ ···O3$_{(-OH2)}$<br>*N2-H13$_{(-NH3+)}$ ···F4 & C2-H2$_{(-CH)}$ ···O3$_{(-OH2)}$<br>*N2-H14$_{(-NH3+)}$ ···F1 & C2-H2$_{(-CH)}$ ···O3$_{(-OH2)}$<br>*N2-H14$_{(-NH3+)}$ ···F2 & C2-H2$_{(-CH)}$ ···O3$_{(-OH2)}$<br>*O3-H15$_{(-OH2)}$···F5 & N2-H12$_{(-NH3+)}$ ···F2<br>*O3-H16$_{(-OH2)}$···F1 & N2-H12$_{(-NH3+)}$ ···F2
	$D^{2}_{2}(10)$	N1-H5$_{(-NH3+)}$ ···O3$_{(-OH2)}$ & N2-H12$_{(-NH3+)}$ ···F2
	$D^{3}_{2}(8)$	*N1-H4$_{(-NH3+)}$ ···O2$_{(-COOH)}$ **ornithine** & (N1-H4$_{(-NH3+)}$ ···F5)$_{2}$<br>*N1-H5$_{(-NH3+)}$ ···O3$_{(-OH2)}$ & C4-H9$_{(-CH2)}$ ···F1
	$D^{3}_{3}(10)$	*N1-H4$_{(-NH3+)}$ ···O2$_{(-COOH)}$ **ornithine** & C2-H2$_{(-CH)}$ ···O3$_{(-OH2)}$<br>*(N1-H3$_{(-NH3+)}$ ···F1)$_{2}$ & N1-H4$_{(-NH3+)}$ ···O2$_{(-COOH)}$ **ornithine**<br>*N1-H4$_{(-NH3+)}$ ···O2$_{(-COOH)}$ **ornithine** & (N1-H5$_{(-NH3+)}$ ···O3$_{(-OH2)}$)$_{2}$
	$D^{3}_{3}(12)$	O1-H1$_{(-COOH)}$ ···F6 & N1-H4$_{(-NH3+)}$ ···O2$_{(-COOH)}$ **ornithine** & O1-H1$_{(-COOH)}$ ···F6
	$D^{3}_{3}(14)$	*N1-H4$_{(-NH3+)}$ ···O2$_{(-COOH)}$ **ornithine** & (C4-H9$_{(-CH2)}$ ···F1)$_{2}$
	$D^{3}_{3}(18)$	*N1-H4$_{(-NH3+)}$ ···O2$_{(-COOH)}$ **ornithine** & (N2-H12$_{(-NH3+)}$ ···F2)$_{2}$<br>*N1-H4$_{(-NH3+)}$ ···O2$_{(-COOH)}$ **ornithine** & (N2-H12$_{(-NH3+)}$ ···F3)$_{2}$
**BIHYEX**	$D(2)$	*O5-H1$_{(-COOH)}$ ···O1$_{(-SO4)}$<br>*O7-H15$_{(-OH2)}$···O4$_{(-SO4)}$<br>*O7-H16$_{(-OH2)}$···O4$_{(-SO4)}$<br>*O7-H16$_{(-OH2)}$···O6$_{(-COOH)}$<br>*N1-H3$_{(-NH3+)}$···O4$_{(-SO4)}$<br>*N1-H4$_{(-NH3+)}$···O7$_{(-OH2)}$<br>*N1-H5$_{(-NH3+)}$···O3$_{(-SO4)}$<br>*N2-H12$_{(-NH3+)}$···O3$_{(-SO4)}$<br>*N2-H13$_{(-NH3+)}$···O2$_{(-SO4)}$<br>*N2-H14$_{(-NH3+)}$···O1$_{(-SO4)}$<br>*N2-H14$_{(-NH3+)}$···O2$_{(-SO4)}$<br>*C2-H2$_{(-CH)}$···O7$_{(-OH2)}$
	$C(5)$	N1-H3$_{(-NH3+)}$···O6$_{(-COOH)}$ **ornithine**
level 2	$C^{1}_{2}(4)$	*O7-H15$_{(-OH2)}$···O4$_{(-SO4)}$ & O7-H16$_{(-OH2)}$···O4$_{(-SO4)}$<br>*N2-H13$_{(-NH3+)}$···O2$_{(-SO4)}$ & N2-H14$_{(-NH3+)}$···O2$_{(-SO4)}$
	$C^{1}_{2}(5)$	N1-H4$_{(-NH3+)}$···O7$_{(-OH2)}$ & C2-H2$_{(-CH)}$···O7$_{(-OH2)}$

	$C^1_2(9)$	N1-H5$_{(-NH3+)}$···O3$_{(-SO4)}$ & N2-H12$_{(-NH3+)}$···O3$_{(-SO4)}$
	$C^1_2(10)$	*O5-H1$_{(-COOH)}$···O1$_{(-SO4)}$ & N2-H14$_{(-NH3+)}$···O1$_{(-SO4)}$<br>*O5-H1$_{(-COOH)}$···O1$_{(-SO4)}$ & N2-H13$_{(-NH3+)}$···O2$_{(-SO4)}$
	$C^2_2(6)$	*O7-H16$_{(-OH2)}$···O6$_{(-COOH)}$ & C2-H$_{2(-CH)}$···O7$_{(-OH2)}$<br>*N1-H3$_{(-NH3+)}$···O4$_{(-SO4)}$ & N1-H5$_{(-NH3+)}$···O3$_{(-SO4)}$<br>*N2-H12$_{(-NH3+)}$···O3$_{(-SO4)}$ & N2-H13$_{(-NH3+)}$···O2$_{(-SO4)}$<br>*N2-H12$_{(-NH3+)}$···O3$_{(-SO4)}$ & N2-H14$_{(-NH3+)}$···O1$_{(-SO4)}$<br>*N2-H12$_{(-NH3+)}$···O3$_{(-SO4)}$ & N2-H14$_{(-NH3+)}$···O2$_{(-SO4)}$<br>*N2-H13$_{(-NH3+)}$···O2$_{(-SO4)}$ & N2-H14$_{(-NH3+)}$···O1$_{(-SO4)}$
	$C^2_2(7)$	O7-H16$_{(-OH2)}$···O6$_{(-COOH)}$ & N1-H4$_{(-NH3+)}$···O7$_{(-OH2)}$
	$C^2_2(9)$	*O5-H1$_{(-COOH)}$···O1$_{(-SO4)}$ & N1-H3$_{(-NH3+)}$···O4$_{(-SO4)}$<br>*O5-H1$_{(-COOH)}$···O1$_{(-SO4)}$ & N1-H5$_{(-NH3+)}$···O3$_{(-SO4)}$
	$C^2_2(11)$	*N1-H3$_{(-NH3+)}$···O4$_{(-SO4)}$ & N2-H12$_{(-NH3+)}$···O3$_{(-SO4)}$<br>*N1-H3$_{(-NH3+)}$···O4$_{(-SO4)}$ & N2-H13$_{(-NH3+)}$···O2$_{(-SO4)}$<br>*N1-H3$_{(-NH3+)}$···O3$_{(-SO4)}$ & N2-H14$_{(-NH3+)}$···O1$_{(-SO4)}$<br>*N1-H3$_{(-NH3+)}$···O4$_{(-SO4)}$ & N2-H14$_{(-NH3+)}$···O2$_{(-SO4)}$<br>*N1-H5$_{(-NH3+)}$···O3$_{(-SO4)}$ & N2-H13$_{(-NH3+)}$···O2$_{(-SO4)}$<br>*N1-H5$_{(-NH3+)}$···O3$_{(-SO4)}$ & N2-H14$_{(-NH3+)}$···O2$_{(-SO4)}$
	$C^2_2(12)$	*O5-H1$_{(-COOH)}$···O1$_{(-SO4)}$ & N2-H12$_{(-NH3+)}$···O3$_{(-SO4)}$<br>*O5-H1$_{(-COOH)}$···O1$_{(-SO4)}$ & N2-H13$_{(-NH3+)}$···O2$_{(-SO4)}$<br>*O5-H1$_{(-COOH)}$···O1$_{(-SO4)}$ & N2-H14$_{(-NH3+)}$···O2$_{(-SO4)}$
	$R^2_1(4)$	N2-H14$_{(-NH3+)}$···O1$_{(-SO4)}$ & N2-H14$_{(-NH3+)}$···O2$_{(-SO4)}$
	$D^1_2(3)$	*O7-H15$_{(-OH2)}$···O4$_{(-SO4)}$ & N1-H3$_{(-NH3+)}$···O4$_{(-SO4)}$<br>*O7-H16$_{(-OH2)}$···O4$_{(-SO4)}$ & N1-H3$_{(-NH3+)}$···O4$_{(-SO4)}$
	$D^2_1(3)$	O7-H16$_{(-OH2)}$···O4$_{(-SO4)}$ & O7-H16$_{(-OH2)}$···O6$_{(-COOH)}$
	$D^2_2(4)$	*O7-H15$_{(-OH2)}$···O4$_{(-SO4)}$ & C2-H2$_{(-CH)}$···O7$_{(-OH2)}$<br>*O7-H16$_{(-OH2)}$···O4$_{(-SO4)}$ & C2-H2$_{(-CH)}$···O7$_{(-OH2)}$<br>*O7-H15$_{(-OH2)}$···O4$_{(-SO4)}$ & N1-H4$_{(-NH3+)}$···O7$_{(-OH2)}$<br>*O7-H1$_{6(-OH2)}$···O4$_{(-SO4)}$ & N1-H4$_{(-NH3+)}$···O7$_{(-OH2)}$
	$D^2_2(5)$	*O5-H1$_{(-COOH)}$···O1$_{(-SO4)}$ & O7-H15···O4$_{(-SO4)}$<br>*O5-H1$_{(-COOH)}$···O1$_{(-SO4)}$ & O7-H16···O4$_{(-SO4)}$<br>*O7-H15$_{(-OH2)}$···O4$_{(-SO4)}$ & O7-H16$_{(-OH2)}$···O6$_{(-COOH)}$<br>*N1-H5$_{(-NH3+)}$···O3$_{(-SO4)}$ & N1-H4$_{(-NH3+)}$···O7$_{(-OH2)}$<br>*O7-H15$_{(-OH2)}$···O4$_{(-SO4)}$ & N1-H5$_{(-NH3+)}$···O3$_{(-SO4)}$<br>*O7-H15$_{(-OH2)}$···O4$_{(-SO4)}$ & N2-H13$_{(-NH3+)}$···O2$_{(-SO4)}$<br>*O7-H15$_{(-OH2)}$···O4$_{(-SO4)}$ & N2-H14$_{(-NH3+)}$···O1$_{(-SO4)}$<br>*O7-H15$_{(-OH2)}$···O4$_{(-SO4)}$ & N2-H14$_{(-NH3+)}$···O2$_{(-SO4)}$<br>*O7-H16$_{(-OH2)}$···O4$_{(-SO4)}$ & N1-H5$_{(-NH3+)}$···O3$_{(-SO4)}$<br>*O7-H16$_{(-OH2)}$···O4$_{(-SO4)}$ & N2-H12$_{(-NH3+)}$···O3$_{(-SO4)}$<br>*O7-H16$_{(-OH2)}$···O4$_{(-SO4)}$ & N2-H13$_{(-NH3+)}$···O2$_{(-SO4)}$<br>*O7-H16$_{(-OH2)}$···O4$_{(-SO4)}$ & N2-H14$_{(-NH3+)}$···O1$_{(-SO4)}$<br>*O7-H16$_{(-OH2)}$···O4$_{(-SO4)}$ & N2-H14$_{(-NH3+)}$···O2$_{(-SO4)}$
	$D^2_2(6)$	*O5-H1$_{(-COOH)}$···O1$_{(-SO4)}$ & O7-H16$_{(-OH2)}$···O6$_{(-COOH)}$<br>*N1-H3$_{(-NH3+)}$···O4$_{(-SO4)}$ & C2-H2$_{(-CH)}$···O7$_{(-OH2)}$<br>*N1-H5$_{(-NH3+)}$···O3$_{(-SO4)}$ & C2-H2$_{(-CH)}$···O7$_{(-OH2)}$
	$D^2_2(7)$	*O5-H1$_{(-COOH)}$···O1$_{(-SO4)}$ & C2-H2$_{(-CH)}$···O7$_{(-OH2)}$<br>*O7-H16$_{(-OH2)}$···O6$_{(-COOH)}$ & N1-H5$_{(-NH3+)}$···O3$_{(-SO4)}$<br>*O7-H16$_{(-OH2)}$···O6$_{(-COOH)}$ & N1-H3$_{(-NH3+)}$···O4$_{(-SO4)}$
	$D^2_2(8)$	O5-H1$_{(-COOH)}$···O1$_{(-SO4)}$ & N1-H4$_{(-NH3+)}$···O7$_{(-OH2)}$
	$D^2_2(9)$	*N2-H12$_{(-NH3+)}$···O3$_{(-SO4)}$ & C2-H2$_{(-CH)}$···O7$_{(-OH2)}$<br>*N2-H13$_{(-NH3+)}$···O2$_{(-SO4)}$ & C2-H2$_{(-CH)}$···O7$_{(-OH2)}$<br>*N2-H14$_{(-NH3+)}$···O1$_{(-SO4)}$ & C2-H2$_{(-CH)}$···O7$_{(-OH2)}$<br>*N2-H14$_{(-NH3+)}$···O2$_{(-SO4)}$ & C2-H2$_{(-CH)}$···O7$_{(-OH2)}$
	$D^2_2(10)$	*O7-H16$_{(-OH2)}$···O6$_{(-COOH)}$ & N2-H12$_{(-NH3+)}$···O3$_{(-SO4)}$<br>*O7-H16$_{(-OH2)}$···O6$_{(-COOH)}$ & N2-H13$_{(-NH3+)}$···O2$_{(-SO4)}$<br>*O7-H16$_{(-OH2)}$···O6$_{(-COOH)}$ & N2-H14$_{(-NH3+)}$···O1$_{(-SO4)}$<br>*O7-H16$_{(-OH2)}$···O6$_{(-COOH)}$ & N2-H14$_{(-NH3+)}$···O2$_{(-SO4)}$<br>*N1-H4$_{(-NH3+)}$···O7$_{(-OH2)}$ & N2-H12$_{(-NH3+)}$···O3$_{(-SO4)}$<br>*N1-H4$_{(-NH3+)}$···O7$_{(-OH2)}$ & N2-H13$_{(-NH3+)}$···O2$_{(-SO4)}$<br>*N1-H4$_{(-NH3+)}$···O7$_{(-OH2)}$ & N2-H14$_{(-NH3+)}$···O1$_{(-SO4)}$<br>*N1-H4$_{(-NH3+)}$···O7$_{(-OH2)}$ & N2-H14$_{(-NH3+)}$···O2$_{(-SO4)}$
	$D^2_3(8)$	(O7-H16$_{(-OH2)}$···O6$_{(-COOH)}$)$_2$ & N1-H3$_{(-NH3+)}$···O6$_{(-COOH)}$ **ornithine**
	$D^3_2(8)$	N1-H3$_{(-NH3+)}$···O4$_{(-SO4)}$ & N1-H3$_{(-NH3+)}$···O6$_{(-COOH)}$ **ornithine**
	$D^3_3(10)$	*N1-H3$_{(-NH3+)}$···O6$_{(-COOH)}$ **ornithine** & C2-H2$_{(-CH)}$···O7$_{(-OH2)}$
	$D^3_3(12)$	O5-H1$_{(-COOH)}$···O1$_{(-SO4)}$ & N1-H3$_{(-NH3+)}$···O6$_{(-COOH)}$ **ornithine**

	$D^{3}_{3}(18)$	*N1-H3$_{(-NH3+)}$···O6$_{(-COOH)}$ **ornithine** & (N2-H12$_{(-NH3+)}$···O3$_{(-SO4)}$)$_2$<br>*N1-H3$_{(-NH3+)}$···O6$_{(-COOH)}$ **ornithine** & (N2-H13$_{(-NH3+)}$···O2$_{(-SO4)}$)$_2$<br>*N1-H3$_{(-NH3+)}$···O6$_{(-COOH)}$ **ornithine** & (N2-H14$_{(-NH3+)}$···O1$_{(-SO4)}$)$_2$<br>*N1-H3$_{(-NH3+)}$···O6$_{(-COOH)}$ **ornithine** & (N2-H14$_{(-NH3+)}$···O2$_{(-SO4)}$)$_2$
**CAPRAM**	$C(4)$	*C2-H4$_{(-CH)}$ ···O1$_{(-COO-)}$ **ornithine**<br>*C7-H17$_{(-CH)}$ ···O3$_{(-COO-)}$
	$C(6)$	N3-H14$_{(-NH3+)}$ ···O5$_{(-COO-)}$ & N3-H15$_{(-NH3+)}$···O5$_{(-COO-)}$
	$C(8)$	*N2-H11$_{(-NH3+)}$ ···O1$_{(-COO-)}$ **ornithine**<br>*N2-H11$_{(-NH3+)}$ ···O2$_{(-COO-)}$ **ornithine**<br>*N2-H13$_{(-NH3+)}$ ···O2$_{(-COO-)}$ **ornithine**
	$D(2)$	*O7-H20B$_{(-OH2)}$ ···O6$_{(-COO-)}$<br>*N1-H1$_{(-NH3+)}$ ···O4$_{(-COO-)}$<br>*N1-H2$_{(-NH3+)}$ ···O3$_{(-COO-)}$<br>*N1-H2$_{(-NH3+)}$ ···O4$_{(-COO-)}$<br>*N1-H3$_{(-NH3+)}$ ···O3$_{(-COO-)}$<br>*N2-H12$_{(-NH3+)}$ ···O6$_{(-COO-)}$<br>*N3-H16$_{(-NH3+)}$···O1$_{(-COO-)}$
level 2	$C^{1}_{2}(4)$	*N1-H1$_{(-NH3+)}$···O4$_{(-COO-)}$ & N1-H2$_{(-NH3+)}$···O4$_{(-COO-)}$<br>*N2-H11$_{(-NH3+)}$···O2$_{(-COO-)}$ **ornithine** & N2-H13$_{(-NH3+)}$···O2$_{(-COO-)}$ **ornithine**<br>*N2-H11$_{(-NH3+)}$···O2$_{(-COO-)}$ **ornithine** & (N3-H16$_{(-NH3+)}$···O1$_{(-COO-)}$)$_2$<br>*N3-H14$_{(-NH3+)}$···O5$_{(-COO-)}$ & N3-H15$_{(-NH3+)}$ ···O5$_{(-COO-)}$
	$C^{1}_{2}(8)$	N2-H11$_{(-NH3+)}$···O1$_{(-COO-)}$ **ornithine** & C2-H4$_{(-CH)}$···O1$_{(-COO-)}$ **ornithine**
	$C^{2}_{2}(6)$	*N1-H1$_{(-NH3+)}$···O4$_{(-COO-)}$ & N1-H2$_{(-NH3+)}$···O3$_{(-COO-)}$<br>*N1-H1$_{(-NH3+)}$···O4$_{(-COO-)}$ & N1-H3$_{(-NH3+)}$···O3$_{(-COO-)}$<br>*N1-H11$_{(-NH3+)}$···O1$_{(-COO-)}$ **ornithine** & N2-H13$_{(-NH3+)}$···O2$_{(-COO-)}$**ornithine**
	$C^{2}_{2}(10)$	*N1-H2$_{(-NH3+)}$···O3$_{(-COO-)}$ & N3-H16$_{(-NH3+)}$···O1$_{(-COO-)}$<br>*N1-H2$_{(-NH3+)}$···O4$_{(-COO-)}$ & N3-H16$_{(-NH3+)}$···O1$_{(-COO-)}$<br>*N1-H3$_{(-NH3+)}$···O3$_{(-COO-)}$ & N3-H16$_{(-NH3+)}$···O1$_{(-COO-)}$<br>*N2-H11$_{(-NH3+)}$···O1$_{(-COO-)}$ **ornithine** & C2-H4$_{(-CH)}$···O1$_{(-COO-)}$ **ornithine**<br>*N2-H13$_{(-NH3+)}$···O2$_{(-COO-)}$ **ornithine** & C2-H4$_{(-CH)}$···O1$_{(-COO-)}$ **ornithine**<br>*N3-H14$_{(-NH3+)}$···O5$_{(-COO-)}$ & C7-H17$_{(-CH)}$···O3$_{(-COO-)}$<br>*N3-H14$_{(-NH3+)}$···O5$_{(-COO-)}$ & C7-H17$_{(-CH)}$···O3$_{(-COO-)}$
	$C^{2}_{2}(11)$	*N3-H15$_{(-NH3+)}$···O5$_{(-COO-)}$ & C7-H17$_{(-CH)}$···O3$_{(-COO-)}$<br>*N3-H15$_{(-NH3+)}$···O5$_{(-COO-)}$ & C7-H17$_{(-CH)}$···O3$_{(-COO-)}$
	$C^{2}_{2}(12)$	*N2-H11$_{(-NH3+)}$···O1$_{(-COO-)}$ **ornithine** & C2-H4$_{(-CH)}$···O1$_{(-COO-)}$ **ornithine**<br>*N2-H13$_{(-NH3+)}$···O2$_{(-COO-)}$ **ornithine** & C2-H4$_{(-CH)}$···O1$_{(-COO-)}$ **ornithine**
	$C^{2}_{2}(14)$	*N1-H2$_{(-NH3+)}$···O3$_{(-COO-)}$ & N2-H12$_{(-NH3+)}$···O6$_{(-COO-)}$<br>*N1-H2$_{(-NH3+)}$···O4$_{(-COO-)}$ & N2-H12$_{(-NH3+)}$···O6$_{(-COO-)}$<br>*N1-H3$_{(-NH3+)}$···O3$_{(-COO-)}$ & N2-H12$_{(-NH3+)}$···O6$_{(-COO-)}$<br>*N2-H12$_{(-NH3+)}$···O6$_{(-COO-)}$ & N3-H16$_{(-NH3+)}$···O1$_{(-COO-)}$
	$C^{2}_{2}(16)$	N2-H11$_{(-NH3+)}$···O1$_{(-COO-)}$ **ornithine** & N2-H11$_{(-NH3+)}$···O2$_{(-COO-)}$ **ornithine**
	$C^{3}_{3}(12)$	*(O7-H20B$_{(-OH2)}$···O6$_{(-COO-)}$)$_2$ & N3-H14$_{(-NH3+)}$···O5$_{(-COO-)}$<br>*(O7-H20B$_{(-OH2)}$···O6$_{(-COO-)}$)$_2$ & N3-H15$_{(-NH3+)}$···O5$_{(-COO-)}$
	$C^{3}_{3}(14)$	(O7-H2OB$_{(-OH2)}$ ···O6$_{(-COO-)}$)$_2$ & C7-H17$_{(-CH)}$···O3$_{(-COO-)}$
	$R^{2}_{1}(4)$	*N1-H2$_{(-NH3+)}$···O3$_{(-COO-)}$ & N1-H2$_{(-NH3+)}$···O4$_{(-COO-)}$<br>*N2-H11$_{(-NH3+)}$···O1$_{(-COO-)}$ **ornithine** & N2-H11$_{(-NH3+)}$···O2$_{(-COO-)}$ **ornithine**
	$R^{2}_{2}(12)$	N3-H14$_{(-NH3+)}$···O5$_{(-COO-)}$ & N3-H15$_{(-NH3+)}$···O5$_{(-COO-)}$
	$R^{2}_{2}(16)$	*N2-H11$_{(-NH3+)}$···O1$_{(-COO-)}$**ornithine** & N2-H13$_{(-NH3+)}$···O2$_{(-COO-)}$ **ornithine**<br>*N2-H11$_{(-NH3+)}$···O1$_{(-COO-)}$ **ornithine** & N2-H13$_{(-NH3+)}$···O2$_{(-COO-)}$ **ornithine**
	$R^{2}_{3}(16)$	(N2-H11$_{(-NH3+)}$···O1$_{(-COO-)}$ **ornithine**)$_2$ & C2-H4$_{(-CH)}$···O1$_{(-COO-)}$ **ornithine**
	$R^{3}_{3}(10)$	(N3-H15$_{(-NH3+)}$···O5$_{(-COO-)}$)$_2$ & C7-H17$_{(-CH)}$···O3$_{(-COO-)}$
	$R^{3}_{3}(16)$	(N3-H14$_{(-NH3+)}$···O5$_{(-COO-)}$)$_2$ & C7-H17$_{(-CH)}$···O3$_{(-COO-)}$
	$R^{3}_{3}(18)$	(N2-H11$_{(-NH3+)}$···O2$_{(-COO-)}$ **ornithine**)$_2$ & C2-H4$_{(-CH)}$···O1$_{(-COO-)}$ **ornithine**
	$R^{3}_{3}(20)$	(N2-H13$_{(-NH3+)}$···O2 $_{(-COO-)}$ **ornithine**)$_2$ & C2-H4$_{(-CH)}$···O1$_{(-COO-)}$ **ornithine**
	$R^{3}_{4}(20)$	(N2-H11$_{(-NH3+)}$···O1$_{(-COO-)}$ **ornithine**)$_2$ & (C2-H4$_{(-CH)}$···O1$_{(-COO-)}$ **ornithine**)$_2$
	$R^{4}_{4}(12)$	(N1-H2$_{(-NH3+)}$ ···O4$_{(-COO-)}$)$_2$ & (N1-H3$_{(-NH3+)}$···O3$_{(-COO-)}$)$_2$
	$R^{4}_{4}(20)$	*(N3-H14$_{(-NH3+)}$ ···O5$_{(-COO-)}$)$_2$ & (C7-H17$_{(-CH)}$···O3$_{(-COO-)}$)$_2$<br>*(N3-H15$_{(-NH3+)}$ ···O5$_{(-COO-)}$)$_2$ & (C7-H17$_{(-CH)}$···O3$_{(-COO-)}$)$_2$
	$R^{2}_{4}(8)$	(N1-H2$_{(-NH3+)}$···O3$_{(-COO-)}$)$_2$ & (N1-H3$_{(-NH3+)}$···O3$_{(-COO-)}$)$_2$
	$R^{4}_{4}(20)$	(N1-H1$_{(-NH3+)}$···O4$_{(-COO-)}$)$_2$ & (N3-H16$_{(-NH3+)}$···O1$_{(-COO-)}$)$_2$
	$D^{1}_{2}(3)$	O7-H20B$_{(-OH2)}$···O6$_{(-COO-)}$ & N2-H12$_{(-NH3+)}$···O6$_{(-COO-)}$
	$D^{2}_{2}(8)$	*O7-H20B$_{(-OH2)}$···O6$_{(-COO-)}$ & (N1-H2$_{(-NH3+)}$···O3$_{(-COO-)}$)$_2$<br>*O7-H20B$_{(-OH2)}$···O6$_{(-COO-)}$ & N1-H2$_{(-NH3+)}$···O4$_{(-COO-)}$<br>*O7-H20B$_{(-OH2)}$···O6$_{(-COO-)}$ & N1-H3$_{(-NH3+)}$···O3$_{(-COO-)}$<br>*O7-H20B$_{(-OH2)}$···O6$_{(-COO-)}$ & N3-H16$_{(-NH3+)}$···O1$_{(-COO-)}$

	$D^{2}_{3}(6)$	(O7-H20B$_{(-OH2)}$···O6$_{(-COO-)}$)$_{2}$ & N2-H12$_{(-NH3+)}$···O6$_{(-COO-)}$
	$D^{2}_{3}(7)$	*(N1-H2$_{(-NH3+)}$···O3$_{(-COO-)}$)$_{2}$ & C7-H17$_{(-CH)}$···O3$_{(-COO-)}$<br>*N1-H3$_{(-NH3+)}$···O3$_{(-COO-)}$ & C7-H17$_{(-CH)}$···O3$_{(-COO-)}$<br>*(N3-H16$_{(-NH3+)}$···O1$_{(-COO-)}$)$_{2}$ & C2-H4$_{(-CH)}$···O1$_{(-COO-)}$ **ornithine**
	$D^{3}_{3}(9)$	*(N1-H1$_{(-NH3+)}$···O4$_{(-COO-)}$)$_{2}$ & C7-H17$_{(-CH)}$···O3$_{(-COO-)}$<br>*(N1-H2$_{(-NH3+)}$···O4$_{(-COO-)}$)$_{2}$ & C7-H17$_{(-CH)}$···O3$_{(-COO-)}$
	$D^{3}_{3}(11)$	*(O7-H20B$_{(-OH2)}$ ···O6$_{(-COO-)}$)$_{2}$ & N1-H1$_{(-NH3+)}$···O4$_{(-COO-)}$<br>*(O7-H20B$_{(-OH2)}$···O6$_{(-COO-)}$)$_{2}$ & N1-H2$_{(-NH3+)}$···O4$_{(-COO-)}$<br>*(O7-H20B$_{(-OH2)}$···O6$_{(-COO-)}$)$_{2}$ & N1-H2$_{(-NH3+)}$···O3$_{(-COO-)}$<br>*(O7-H20B$_{(-OH2)}$···O6$_{(-COO-)}$)$_{2}$ & N1-H3$_{(-NH3+)}$···O3$_{(-COO-)}$<br>*(N1-H1$_{(-NH3+)}$···O4$_{(-COO-)}$)$_{2}$ & C2-H4$_{(-CH)}$···O1$_{(-COO-)}$ **ornithine**<br>*(N1-H$_{2(-NH3+)}$···O3$_{(-COO-)}$)$_{2}$ & C2-H4$_{(-CH)}$···O1$_{(-COO-)}$ **ornithine**<br>*(N1-H2$_{(-NH3+)}$···O4$_{(-COO-)}$)$_{2}$ & C2-H4$_{(-CH)}$···O1$_{(-COO-)}$ **ornithine**<br>*(N1-H3$_{(-NH3+)}$···O3$_{(-COO-)}$)$_{2}$ & C2-H4$_{(-CH)}$···O1$_{(-COO-)}$ **ornithine**<br>*N2-H11$_{(-NH3+)}$···O1$_{(-COO-)}$ **ornithine** & (N3-H16$_{(-NH3+)}$···O1$_{(-COO-)}$)$_{2}$<br>*(N2-H12$_{(-NH3+)}$···O6$_{(-COO-)}$)$_{2}$ & N3-H14$_{(-NH3+)}$···O5$_{(-COO-)}$<br>*(N2-H12$_{(-NH3+)}$···O6$_{(-COO-)}$)$_{2}$ & N3-H15$_{(-NH3+)}$···O5$_{(-COO-)}$<br>*N3-H14$_{(-NH3+)}$···O5$_{(-COO-)}$ & (N3-H16$_{(-NH3+)}$···O1$_{(-COO-)}$)$_{2}$<br>*(N3-H16$_{(-NH3+)}$···O2$_{(-COO-)}$)$_{2}$ & N3-H15$_{(-NH3+)}$···O5$_{(-COO-)}$<br>*(N3-H16$_{(-NH3+)}$···O1$_{(-COO-)}$)$_{2}$ & C7-H17$_{(-CH)}$···O3$_{(-COO-)}$<br>*(O7-H20B$_{(-OH2)}$···O6$_{(-COO-)}$)$_{2}$ & N3-H16$_{(-NH3+)}$···O1$_{(-COO-)}$
	$D^{3}_{3}(13)$	*(N1-H2$_{(-NH3+)}$···O3$_{(-COO-)}$)$_{2}$ & N3-H14$_{(-NH3+)}$···O5$_{(-COO-)}$<br>*(N1-H2$_{(-NH3+)}$···O3$_{(-COO-)}$)$_{2}$ & N3-H15$_{(-NH3+)}$···O5$_{(-COO-)}$<br>*(N1-H1$_{(-NH3+)}$···O4$_{(-COO-)}$)$_{2}$ & N3-H14$_{(-NH3+)}$···O5$_{(-COO-)}$<br>*(N1-H1$_{(-NH3+)}$···O4$_{(-COO-)}$)$_{2}$ & N3-H15$_{(-NH3+)}$···O5$_{(-COO-)}$<br>*(N1-H2$_{(-NH3+)}$···O4$_{(-COO-)}$)$_{2}$ & N3-H14$_{(-NH3+)}$···O5$_{(-COO-)}$<br>*(N1-H$_{2(-NH3+)}$···O4$_{(-COO-)}$)$_{2}$ & N3-H15$_{(-NH3+)}$···O5$_{(-COO-)}$<br>*(N1-H3$_{(-NH3+)}$···O3$_{(-COO-)}$)$_{2}$ & N3-H14$_{(-NH3+)}$···O5$_{(-COO-)}$<br>*(N1-H3$_{(-NH3+)}$···O3$_{(-COO-)}$)$_{2}$ & N3-H15$_{(-NH3+)}$···O5$_{(-COO-)}$<br>*N2-H11$_{(-NH3+)}$···O1$_{(-COO-)}$ **ornithine** & (N2-H12$_{(-NH3+)}$···O6$_{(-COO-)}$)$_{2}$<br>*N2-H11$_{(-NH3+)}$···O2$_{(-COO-)}$ **ornithine** & (N2-H12$_{(-NH3+)}$···O6$_{(-COO-)}$)$_{2}$<br>*N2-H11$_{(-NH3+)}$···O2$_{(-COO-)}$ **ornithine** & (N3-H16$_{(-NH3+)}$···O1$_{(-COO-)}$)$_{2}$<br>*N2-H13$_{(-NH3+)}$···O2$_{(-COO-)}$ **ornithine** & (N2-H16$_{(-NH3+)}$···O6$_{(-COO-)}$)$_{2}$<br>*(N2-H12$_{(-NH3+)}$···O6$_{(-COO-)}$)$_{2}$ & C7-H17$_{(-CH)}$···O3$_{(-COO-)}$<br>*N2-H13$_{(-NH3+)}$···O2$_{(-COO-)}$ **ornithine** & (N3-H16$_{(-NH3+)}$···O1$_{(-COO-)}$)$_{2}$
	$D^{3}_{3}(15)$	*(N1-H1$_{(-NH3+}$··O4$_{(-COO-)}$)$_{2}$ & N2-H11$_{(-NH3+)}$···O2$_{(-COO-)}$ **ornithine**<br>*(N1-H1$_{(-NH3+}$··O4$_{(-COO-)}$)$_{2}$ & N2-H13$_{(-NH3+)}$···O2$_{(-COO-)}$ **ornithine**<br>*(N1-H2$_{(-NH3+)}$···O3$_{(-COO-)}$)$_{2}$ & N2-H11$_{(-NH3+)}$···O1$_{(-COO-)}$ **ornithine**<br>*N2-H11$_{(-NH3+)}$···O2$_{(-COO-)}$ **ornithine** & (N1-H2$_{(-NH3+)}$···O3$_{(-COO-)}$)$_{2}$<br>*(N1-H2$_{(-NH3+)}$···O3$_{(-COO-)}$)$_{2}$ & N2-H13$_{(-NH3+)}$···O2$_{(-COO-)}$ **ornithine**<br>*(N1-H2$_{(-NH3+)}$···O4$_{(-COO-)}$)$_{2}$ & N2-H11$_{(-NH3+)}$···O1$_{(-COO-)}$ **ornithine**<br>*(N1-H2$_{(-NH3+)}$···O4$_{(-COO-)}$)$_{2}$ & N2-H13$_{(-NH3+)}$···O2$_{(-COO-)}$ **ornithine**<br>*(N1-H3$_{(-NH3+)}$···O3$_{(-COO-)}$)$_{2}$ & N2-H11$_{(-NH3+)}$···O1$_{(-COO-)}$ **ornithine**<br>*(N1-H3$_{(-NH3+)}$···O3$_{(-COO-)}$)$_{2}$ & N2-H11$_{(-NH3+)}$···O2$_{(-COO-)}$ **ornithine**<br>*(N1-H3$_{(-NH3+)}$···O3$_{(-COO-)}$)$_{2}$ & N2-H13$_{(-NH3+)}$···O2$_{(-COO-)}$ **ornithine**
	$D^{3}_{3}(17)$	(N2-H12$_{(-NH3+)}$ ···O6$_{(-COO-)}$)$_{2}$ & C2-H4···O1$_{(-COO-)}$ **ornithine**
**EVIJAU**	$C(5)$	N1-H5$_{(-NH3+)}$···O1$_{(-COOH)}$ **ornithine**
	$C(8)$	N2-H12$_{(-NH3+)}$···O1$_{(-COOH)}$ **ornithine**
	$R^{2}_{2}(12)$	(C4-H9$_{(-CH2)}$ ···O2$_{(-COOH)}$ **ornithine**)$_{2}$
	$D(2)$	*O2-H1$_{(-COOH)}$···O6$_{(-SO4)}$<br>*N1-H3$_{(-NH3+)}$···Cl1<br>*N1-H4$_{(-NH3+)}$···O5A$_{(-SO4)}$<br>*N1-H5$_{(-NH3+)}$···O3$_{(-NO3-)}$<br>*C3-H6$_{(-CH2)}$ ···O4$_{(-NO3-)}$<br>*N2-H12$_{(-NH3+)}$···Cl1<br>*N2-H13$_{(-NH3+)}$···O4A$_{(-NO3-)}$<br>*N2-H14$_{(-NH3+)}$···O6$_{(-SO4)}$<br>*N2-H14$_{(-NH3+)}$···O5A$_{(-SO4)}$
	$D^{1}_{2}(3)$	*(N1-H3$_{(-NH3+)}$···Cl1)$_{2}$<br>*(N1-H3$_{(-NH3+)}$···O3$_{(-NO3-)}$)$_{2}$<br>*(N2-H12$_{(-NH3+)}$···Cl1)$_{2}$
	$D^{2}_{2}(5)$	*O2-H1$_{(-COOH)}$···O6$_{(-SO4)}$ & O2-H1$_{(-COOH)}$···O6A$_{(-SO4)}$<br>*N1-H4$_{(-NH3+)}$···O5A$_{(-SO4)}$ & N1-H4$_{(-NH3+)}$···O5$_{(-SO4)}$<br>*C3-H6$_{(-CH2)}$···O4A$_{(-NO3-)}$ & C3-H6$_{(-CH2)}$···O4$_{(-NO3-)}$<br>*N2-H14$_{(-NH3+)}$···O6$_{(-SO4)}$ & N2-H14$_{(-NH3+)}$···O6A$_{(-SO4)}$<br>*N2-H13$_{(-NH3+)}$···O4A$_{(-NO3-)}$ & N2-H13$_{(-NH3+)}$···O4$_{(-NO3-)}$

		*N2-H14$_{(-NH3+)}$···O5A$_{(-SO4)}$ & N2-H14$_{(-NH3+)}$···O5$_{(-SO4)}$
level 2	$C^{1}_{2}(7)$	C3-H6$_{(-CH2)}$···O4$_{(-NO3-)}$
	$C^{1}_{2}(9)$	*N1-H3$_{(-NH3+)}$···Cl1 & N2-H12$_{(-NH3+)}$···Cl1<br>*N1-H4$_{(-NH3+)}$···O5A$_{(-SO4)}$ & N2-H14$_{(-NH3+)}$···O5A$_{(-SO4)}$<br>*N1-H5$_{(-NH3+)}$···O1$_{(-COOH)}$ **ornithine** & N2-H12$_{(-NH3+)}$···O1$_{(-COOH)}$ **ornithine**
	$C^{1}_{2}(10)$	O2-H1$_{(-COOH)}$···O6$_{(-SO4)}$ & N2-H14$_{(-NH3+)}$···O6$_{(-SO4)}$
	$C^{2}_{4}(18)$	N1-H3$_{(-NH3+)}$···Cl1 & (N2-H12$_{(-NH3+)}$···Cl1)$_2$
	$C^{2}_{2}(7)$	N1-H5$_{(-NH3+)}$···O3$_{(-NO3-)}$ & N1-H5$_{(-NH3+)}$···O1$_{(-COOH)}$ **ornithine**
	$C^{2}_{2}(8)$	C4-H9$_{(-CH2)}$···O2$_{(-COOH)}$ **ornithine** & N2-H12$_{(-NH3+)}$···O1$_{(-COOH)}$ **ornithine**
	$C^{2}_{2}(9)$	*O2-H1$_{(-COOH)}$···O6$_{(-SO4)}$ & N1-H1$_{(-NH3+)}$···O5$_{(-SO4)}$<br>*N1-H5$_{(-NH3+)}$···O1$_{(-COOH)}$ **ornithine** & (N1-H3$_{(-NH3+)}$···Cl1)$_2$<br>*N1-H5$_{(-NH3+)}$···O1$_{(-COOH)}$ **ornithine** & C4-H9$_{(-CH2)}$···O2$_{(-COOH)}$ **ornithine**<br>*C3-H6$_{(-CH2)}$···O4$_{(-NO3-)}$ & N2-H13$_{(-NH3+)}$···O4A$_{(-NO3-)}$
	$C^{2}_{2}(10)$	N2-H12$_{(-NH3+)}$···Cl1 & N2-H12$_{(-NH3+)}$···O1$_{(-COOH)}$ **ornithine**
	$C^{2}_{2}(11)$	*N1-H4$_{(-NH3+)}$···O5A$_{(-SO4)}$ & N2-H14$_{(-NH3+)}$···O6$_{(-SO4)}$<br>*N1-H5$_{(-NH3+)}$···O1$_{(-COOH)}$ **ornithine** & C4-H9$_{(-CH2)}$···O2$_{(-COOH)}$ **ornithine**<br>*N1-H5$_{(-NH3+)}$···O3$_{(-NO3-)}$ & N2-H13$_{(-NH3+)}$···O4$_{(-NO3-)}$
	$C^{2}_{2}(12)$	*O2-H1$_{(-COOH)}$···O6$_{(-SO4)}$ & N2-H14$_{(-NH3+)}$···O6A$_{(-SO4)}$<br>*O2-H1$_{(-COOH)}$···O6$_{(-SO4)}$ & N2-H14$_{(-NH3+)}$···O5$_{(-SO4)}$
	$C^{2}_{2}(13)$	N1-H5$_{(-NH3+)}$···O1$_{(-COOH)}$ **ornithine** & N2-H12$_{(-NH3+)}$···O1$_{(-COOH)}$ **ornithine**
	$C^{2}_{2}(14)$	C4-H9$_{(-CH2)}$···O2$_{(-COOH)}$ **ornithine** & N2-H12$_{(-NH3+)}$···O1$_{(-COOH)}$ **ornithine**
	$C^{2}_{3}(12)$	(N1-H5$_{(-NH3+)}$···O3$_{(-NO3-)}$)$_2$ & C4-H9$_{(-CH2)}$···O2$_{(-COOH)}$ **ornithine**
	$C^{2}_{3}(14)$	*(N1-H3$_{(-NH3+)}$···Cl1)$_2$ & N2-H12$_{(-NH3+)}$···O1$_{(-COOH)}$ **ornithine**<br>*(N1-H5$_{(-NH3+)}$···O3$_{(-NO3-)}$)$_2$ & N2-H12$_{(-NH3+)}$···O1$_{(-COOH)}$ **ornithine**<br>*C4-H9$_{(-CH2)}$···O2$_{(-COOH)}$ **ornithine** & (N2-H12$_{(-NH3+)}$···Cl1)$_2$
	$C^{2}_{3}(17)$	N1-H5$_{(-NH3+)}$···O1$_{(-COOH)}$ **ornithine** & (N2-H12$_{(-NH3+)}$···Cl1)$_2$
	$C^{2}_{4}(18)$	*N1-H3$_{(-NH3+)}$···Cl1 & (N2-H12$_{(-NH3+)}$···Cl1)$_2$<br>*(N1-H5$_{(-NH3+)}$···O3$_{(-NO3-)}$)$_2$ & (N2-H12$_{(-NH3+)}$···Cl1)$_2$
	$C^{3}_{3}(11)$	N1-H4$_{(-NH3+)}$···O5$_{(-SO4)}$ & N1-H4$_{(-NH3+)}$···O5$_{(-SO4)}$ & N1-H5$_{(-NH3+)}$···O1$_{(-COOH)}$ **ornithine**
	$C^{3}_{3}(12)$	*(N1-H3$_{(-NH3+)}$···Cl1)$_2$ & C4-H9$_{(-CH2)}$···O2$_{(-COOH)}$**ornithine**<br>*C3-H6$_{(-CH2)}$···O4$_{(-NO3-)}$ & C3-H6$_{(-CH2)}$···O4A$_{(-NO3-)}$ & C4-H9$_{(-CH2)}$···O2$_{(-COOH)}$ **ornithine**
	$C^{3}_{3}(13)$	*O2-H1$_{(-COOH)}$···O6$_{(-SO4)}$ & O2-H1$_{(-COOH)}$···O6A$_{(-SO4)}$ & N1-H5$_{(-NH3+)}$···O1$_{(-COOH)}$ **ornithine**<br>*N1-H5$_{(-NH3+)}$···O1$_{(-COOH)}$ **ornithine** & C3-H6$_{(-CH2)}$···O4A$_{(-NO3-)}$ & C3-H6$_{(-CH2)}$···O4$_{(-NO3-)}$
	$C^{3}_{3}(14)$	*N1-H4$_{(-NH3+)}$···O5$_{(-SO4)}$ & N1-H4$_{(-NH3+)}$···O5A$_{(-SO4)}$ & C4-H9$_{(-CH2)}$···O2$_{(-COOH)}$ **ornithine**<br>*N2-H12$_{(-NH3+)}$···O1$_{(-COOH)}$ **ornithine** & C3-H6$_{(-CH2)}$···O4$_{(-NO3-)}$ & C3-H6$_{(-CH2)}$···O4A$_{(-NO3-)}$<br>*N2-H13$_{(-NH3+)}$···O4$_{(-NO3-)}$ **ornithine** & N2-H13$_{(-NH3+)}$···O4A$_{(-NO3-)}$ & N2-H12$_{(-NH3+)}$···O1$_{(-COOH)}$ **ornithine**<br>*N2-H14$_{(-NH3+)}$···O6$_{(-SO4)}$ & N2-H14$_{(-NH3+)}$···O6A$_{(-SO4)}$ & N2-H12$_{(-NH3+)}$···O1$_{(-COOH)}$ **ornithine**
	$C^{3}_{3}(16)$	*O2-H1$_{(-COOH)}$···O6$_{(-SO4)}$ & O2-H1$_{(-COOH)}$···O6A$_{(-SO4)}$ & N2-H12$_{(-NH3+)}$···O1$_{(-COOH)}$ **ornithine**<br>*N1-H4$_{(-NH3+)}$···O5$_{(-SO4)}$ & N1-H4$_{(-NH3+)}$···O5A$_{(-SO4)}$ & N2-H12$_{(-NH3+)}$···O1$_{(-COOH)}$ **ornithine**<br>*N2-H13$_{(-NH3+)}$···O4$_{(-NO3-)}$ & N2-H13$_{(-NH3+)}$···O4A$_{(-NO3-)}$ & C4-H9$_{(-CH2)}$···O2$_{(-COOH)}$ **ornithine**<br>*N2-H14$_{(-NH3+)}$···O6$_{(-SO4)}$ & N2-H14$_{(-NH3+)}$···O6A$_{(-SO4)}$ & C4-H9$_{(-CH2)}$···O2$_{(-COOH)}$ **ornithine**<br>*N2-H14$_{(-NH3+)}$···O5$_{(-SO4)}$ & N2-H14$_{(-NH3+)}$···O5A$_{(-SO4)}$ & C4-H9$_{(-CH2)}$···O2$_{(-COOH)}$ **ornithine**
	$C^{3}_{3}(19)$	*N1-H5$_{(-NH3+)}$···O1$_{(-COOH)}$ **ornithine** & N2-H13$_{(-NH3+)}$···O4$_{(-NO3-)}$ & N2-H13$_{(-NH3+)}$···O4A$_{(-NO3-)}$<br>*N1-H5$_{(-NH3+)}$···O1$_{(-COOH)}$ **ornithine** & N2-H14$_{(-NH3+)}$···O6$_{(-SO4)}$ & N2-H14$_{(-NH3+)}$···O6A$_{(-SO4)}$<br>*N2-H14$_{(-NH3+)}$···O5$_{(-SO4)}$ & N1-H5$_{(-NH3+)}$···O1$_{(-COOH)}$ **ornithine** & N2-H14$_{(-NH3+)}$···O5A$_{(-SO4)}$
	$C^{3}_{4}(10)$	*(N1-H3$_{(-NH3+)}$···Cl1)$_2$ & N1-H4$_{(-NH3+)}$···O5$_{(-SO4)}$ & N1-H4$_{(-NH3+)}$···O5A$_{(-SO4)}$<br>*(N1-H5$_{(-NH3+)}$···O3$_{(-NO3-)}$)$_2$ & N1-H4$_{(-NH3+)}$···O5$_{(-SO4)}$ & N1-H4$_{(-NH3+)}$···O5A$_{(-SO4)}$<br>*(N2-H12$_{(-NH3+)}$···Cl1)$_2$ & N2-H14$_{(-NH3+)}$···O6$_{(-SO4)}$ & N2-H14$_{(-NH3+)}$···O6A$_{(-SO4)}$<br>*(N2-H12$_{(-NH3+)}$···Cl1)$_2$ & N2-H14$_{(-NH3+)}$···O5$_{(-SO4)}$
	$C^{3}_{4}(16)$	*O2-H1$_{(-COOH)}$···O6A$_{(-SO4)}$ & (O2-H1$_{(-COOH)}$···O6$_{(-SO4)}$)$_2$ & (N1-H3$_{(-NH3+)}$···Cl1)$_2$<br>*O2-H1$_{(-COOH)}$···O6$_{(-SO4)}$ & O2-H1$_{(-COOH)}$···O6A$_{(-SO4)}$ & (N1-H5$_{(-NH3+)}$···O3$_{(-NO3-)}$)$_2$<br>*C3-H6$_{(-CH2)}$···O4$_{(-NO3-)}$ & C3-H6$_{(-CH2)}$···O4A$_{(-NO3-)}$ & (N2-H12$_{(-NH3+)}$···Cl1)$_2$
	$C^{3}_{4}(20)$	*(N1-H3$_{(-NH3+)}$···Cl1)$_2$ & N2-H13$_{(-NH3+)}$···O4$_{(-NO3-)}$<br>*N2-H14$_{(-NH3+)}$···O6$_{(-SO4)}$ & N2-H14$_{(-NH3+)}$···O6A$_{(-SO4)}$ & (N1-H3$_{(-NH3+)}$···Cl1)$_2$<br>*N1-H4$_{(-NH3+)}$···O5$_{(-SO4)}$ & N1-H4$_{(-NH3+)}$···O5A$_{(-SO4)}$ & (N2-H12$_{(-NH3+)}$···Cl1)$_2$<br>*(N1-H5$_{(-NH3+)}$···O3$_{(-NO3-)}$)$_2$ & N2-H13$_{(-NH3+)}$···O4$_{(-NO3-)}$ & N2-H13$_{(-NH3+)}$···O4A$_{(-NO3-)}$<br>*N2-H14$_{(-NH3+)}$···O5$_{(-SO4)}$ & N2-H14$_{(-NH3+)}$···O5A$_{(-SO4)}$ & (N1-H5$_{(-NH3+)}$···O3$_{(-NO3-)}$)$_2$
	$C^{4}_{4}(12)$	N2-H13$_{(-NH3+)}$···O4$_{(-NO3-)}$ & N2-H13$_{(-NH3+)}$···O4A$_{(-NO3-)}$ & N2-H14$_{(-NH3+)}$···O6$_{(-SO4)}$ & N2-H14$_{(-NH3+)}$···O6A$_{(-SO4)}$
	$C^{4}_{4}(16)$	*N1-H4$_{(-NH3+)}$···O5A$_{(-SO4)}$ & N1-H4$_{(-NH3+)}$···O5$_{(-SO4)}$ & C3-H6$_{(-CH2)}$···O4$_{(-NO3-)}$ & C3-H6$_{(-CH2)}$···O4A$_{(-NO3-)}$<br>*N1-H5$_{(-NH3+)}$···O1$_{(-COOH)}$ **ornithine**)$_2$ & C4-H9$_{(-CH2)}$···O2$_{(-COOH)}$ **ornithine**
	$C^{4}_{4}(18)$	*N1-H4$_{(-NH3+)}$···O5$_{(-SO4)}$ & O2-H1$_{(-COOH)}$···O6$_{(-SO4)}$ & O2-H1$_{(-COOH)}$···O6A$_{(-SO4)}$ & N1-H4$_{(-NH3+)}$···O5A$_{(-SO4)}$<br>*O2-H1$_{(-COOH)}$···O6$_{(-SO4)}$ & O2-H1$_{(-COOH)}$···O6A$_{(-SO4)}$ & C3-H6$_{(-CH2)}$···O4A$_{(-NO3-)}$ & C3-H6$_{(-CH2)}$···O4$_{(-NO3-)}$<br>*C3-H6$_{(-CH2)}$···O4$_{(-NO3-)}$ & C3-H6$_{(-CH2)}$···O4A$_{(-NO3-)}$ & N2-H13$_{(-NH3+)}$···O4$_{(-NO3-)}$ & N2-H13$_{(-NH3+)}$···O4A$_{(-NO3-)}$<br>*C3-H6$_{(-CH2)}$···O4$_{(-NO3-)}$ & N2-H14$_{(-NH3+)}$···O6A$_{(-SO4)}$ & C3-H6$_{(-CH2)}$···O4A$_{(-NO3-)}$ & N2-H14$_{(-NH3+)}$···O6$_{(-SO4)}$<br>*C3-H6$_{(-CH2)}$···O4$_{(-NO3-)}$ & C3-H6$_{(-CH2)}$···O4A$_{(-NO3-)}$ & N2-H14$_{(-NH3+)}$···O5$_{(-SO4)}$ & N2-H14$_{(-NH3+)}$···O5A$_{(-SO4)}$

	$C^{4}_{4}(20)$	(N1-H5$_{\text{(-NH3+)}}$···O1$_{\text{(-COOH)}}$ **ornithine**)$_2$ & C4-H9$_{\text{(-CH2)}}$···O2$_{\text{(-COOH)}}$ **ornithine**
	$R^{2}_{1}(4)$	N2-H14$_{\text{(-NH3+)}}$···O6$_{\text{(-SO4)}}$ & N2-H14$_{\text{(-NH3+)}}$···O5A$_{\text{(-SO4)}}$
	$R^{2}_{2}(8)$	N1-H5$_{\text{(-NH3+)}}$···O3$_{\text{(-NO3-)}}$ & C3-H6$_{\text{(-CH2)}}$···O4$_{\text{(-NO3-)}}$
	$R^{2}_{4}(8)$	(N1-H5$_{\text{(-NH3+)}}$···O3$_{\text{(-NO3-)}}$)$_2$ & (N1-H3$_{\text{(-NH3+)}}$···Cl1)$_2$
	$R^{3}_{3}(12)$	O2-H1$_{\text{(-COOH)}}$···O6$_{\text{(-SO4)}}$ & O2-H1$_{\text{(-COOH)}}$···O6A$_{\text{(-SO4)}}$ & C4-H9$_{\text{(-CH2)}}$···O2$_{\text{(-COOH)}}$ **ornithine**
	$R^{3}_{3}(14)$	(N1-H3$_{\text{(-NH3+)}}$···Cl1)$_2$ & C3-H6$_{\text{(-CH2)}}$···O4$_{\text{(-NO3-)}}$ & C3-H6$_{\text{(-CH2)}}$···O4A$_{\text{(-NO3-)}}$
	$R^{3}_{4}(10)$	(N2-H12$_{\text{(-NH3+)}}$···Cl1)$_2$ & N2-H13$_{\text{(-NH3+)}}$···O4$_{\text{(-NO3-)}}$ & N2-H13$_{\text{(-NH3+)}}$···O4A$_{\text{(-NO3-)}}$
	$D^{1}_{2}(10)$	N1-H4$_{\text{(-NH3+)}}$···O5A$_{\text{(-SO4)}}$ & N2-H13$_{\text{(-NH3+)}}$···O4A$_{\text{(-NO3-)}}$
	$D^{2}_{2}(5)$	*N1-H3$_{\text{(-NH3+)}}$···Cl1 & N1-H4$_{\text{(-NH3+)}}$···O5A$_{\text{(-SO4)}}$<br>*N1-H5$_{\text{(-NH3+)}}$···O3$_{\text{(-NO3-)}}$ & N1-H3$_{\text{(-NH3+)}}$···Cl1<br>*(N2-H12$_{\text{(-NH3+)}}$···Cl1 & N2-H14$_{\text{(-NH3+)}}$···O6$_{\text{(-SO4)}}$<br>*(N2-H12$_{\text{(-NH3+)}}$···Cl1)$_2$ & N2-H14$_{\text{(-NH3+)}}$···O5A$_{\text{(-SO4)}}$<br>*N2-H13$_{\text{(-NH3+)}}$···O4A$_{\text{(-NO3-)}}$ & N2-H14$_{\text{(-NH3+)}}$···O6$_{\text{(-SO4)}}$
	$D^{2}_{2}(7)$	*N1-H3$_{\text{(-NH3+)}}$···Cl1 & C3-H6$_{\text{(-CH2)}}$···O4$_{\text{(-NO3-)}}$<br>*N1-H4$_{\text{(-NH3+)}}$···O5A$_{\text{(-SO4)}}$ & C3-H6$_{\text{(-CH2)}}$···O4$_{\text{(-NO3-)}}$
	$D^{2}_{2}(5)$	N2-H12$_{\text{(-NH3+)}}$···Cl1 & N2-H13$_{\text{(-NH3+)}}$···O4A$_{\text{(-NO3-)}}$
	$D^{2}_{2}(8)$	*O2-H1$_{\text{(-COOH)}}$···O6$_{\text{(-SO4)}}$ & N1-H3$_{\text{(-NH3+)}}$···Cl1<br>*O2-H1$_{\text{(-COOH)}}$···O6$_{\text{(-SO4)}}$ & N1-H5$_{\text{(-NH3+)}}$···O3$_{\text{(-NO3-)}}$<br>*O2-H1$_{\text{(-COOH)}}$···O6$_{\text{(-SO4)}}$ & C3-H6$_{\text{(-CH2)}}$···O4$_{\text{(-NO3-)}}$<br>*N2-H12$_{\text{(-NH3+)}}$···Cl1 & C3-H6$_{\text{(-CH2)}}$···O4$_{\text{(-NO3-)}}$<br>*C3-H6$_{\text{(-CH2)}}$···O4$_{\text{(-NO3-)}}$ & N2-H14$_{\text{(-NH3+)}}$···O6$_{\text{(-SO4)}}$<br>*N2-H14$_{\text{(-NH3+)}}$···O5A$_{\text{(-SO4)}}$ & C3-H6$_{\text{(-CH2)}}$···O4$_{\text{(-NO3-)}}$
	$D^{2}_{2}(10)$	*N1-H3$_{\text{(-NH3+)}}$···Cl1 & N1-H13$_{\text{(-NH3+)}}$···O4A$_{\text{(-NO3-)}}$<br>*N1-H3$_{\text{(-NH3+)}}$···Cl1 & N2-H14$_{\text{(-NH3+)}}$···O6$_{\text{(-SO4)}}$<br>*N2-H14$_{\text{(-NH3+)}}$···O5A$_{\text{(-SO4)}}$ & N1-H3$_{\text{(-NH3+)}}$···Cl1<br>*N1-H4$_{\text{(-NH3+)}}$···O5A$_{\text{(-SO4)}}$ & N2-H12$_{\text{(-NH3+)}}$···Cl1<br>*N1-H5$_{\text{(-NH3+)}}$···O3$_{\text{(-NO3-)}}$ & N2-H12$_{\text{(-NH3+)}}$···Cl1<br>*N1-H5$_{\text{(-NH3+)}}$···O3$_{\text{(-NO3-)}}$ & N2-H14$_{\text{(-NH3+)}}$···O6$_{\text{(-SO4)}}$<br>*(N1-H5$_{\text{(-NH3+)}}$···O3$_{\text{(-NO3-)}}$)$_2$ & N2-H14$_{\text{(-NH3+)}}$···O5A$_{\text{(-SO4)}}$
	$D^{2}_{2}(11)$	*O2-H1$_{\text{(-COOH)}}$···O6$_{\text{(-SO4)}}$ & N2-H12$_{\text{(-NH3+)}}$···Cl1<br>*O2-H1$_{\text{(-COOH)}}$···O6$_{\text{(-SO4)}}$ & N2-H13$_{\text{(-NH3+)}}$···O4A$_{\text{(-NO3-)}}$<br>*C3-H6$_{\text{(-CH2)}}$···O4$_{\text{(-NO3-)}}$ & C3-H6$_{\text{(-CH2)}}$···O4A$_{\text{(-NO3-)}}$ & N2-H14$_{\text{(-NH3+)}}$···O5$_{\text{(-SO4)}}$<br>*N2-H14$_{\text{(-NH3+)}}$···O5A$_{\text{(-SO4)}}$ & C3-H6$_{\text{(-CH2)}}$···O4A$_{\text{(-NO3-)}}$ & N2-H14$_{\text{(-NH3+)}}$···O5$_{\text{(-SO4)}}$
	$D^{2}_{3}(6)$	*N1-H4$_{\text{(-NH3+)}}$···O5$_{\text{(-SO4)}}$ & (N1-H3$_{\text{(-NH3+)}}$···Cl1)$_2$<br>*N1-H5$_{\text{(-NH3+)}}$···O3$_{\text{(-NO3-)}}$ & (N1-H3$_{\text{(-NH3+)}}$···Cl1)$_2$<br>*N1-H5$_{\text{(-NH3+)}}$···O3$_{\text{(-NO3-)}}$ & (N1-H3$_{\text{(-NH3+)}}$···Cl1)$_2$<br>*N1-H4$_{\text{(-NH3+)}}$···O5$_{\text{(-SO4)}}$ & N1-H5$_{\text{(-NH3+)}}$···O1$_{\text{(-COOH)}}$ **ornithine** & (N1-H5$_{\text{(-NH3+)}}$···O3$_{\text{(-NO3-)}}$)$_2$<br>*(N2-H12$_{\text{(-NH3+)}}$···Cl1)$_2$ & N2-H14$_{\text{(-NH3+)}}$···O6A$_{\text{(-SO4)}}$<br>*(N2-H12$_{\text{(-NH3+)}}$···Cl1)$_2$ & N2-H14$_{\text{(-NH3+)}}$···O5$_{\text{(-SO4)}}$
	$D^{2}_{3}(8)$	*(N1-H3$_{\text{(-NH3+)}}$···Cl1)$_2$ & C3-H6$_{\text{(-CH2)}}$···O4A$_{\text{(-NO3-)}}$<br>*N2-H12$_{\text{(-NH3+)}}$···Cl1 & N2-H13$_{\text{(-NH3+)}}$···O4$_{\text{(-NO3-)}}$ & N2-H13$_{\text{(-NH3+)}}$···O4A$_{\text{(-NO3-)}}$
	$D^{2}_{3}(9)$	*O2-H1$_{\text{(-COOH)}}$···O6A$_{\text{(-SO4)}}$ & (N1-H3$_{\text{(-NH3+)}}$···Cl1)$_2$<br>*(N2-H12$_{\text{(-NH3+)}}$···Cl1)$_2$ & C3-H6$_{\text{(-CH2)}}$···O4A$_{\text{(-NO3-)}}$
	$D^{2}_{3}(11)$	*N2-H14$_{\text{(-NH3+}}$··O6A$_{\text{(-SO4)}}$ & (N1-H3$_{\text{(-NH3+)}}$···Cl1)$_2$<br>*N2-H14$_{\text{(-NH3+)}}$···O5$_{\text{(-SO4)}}$ & (N1-H3$_{\text{(-NH3+)}}$···Cl1)$_2$<br>*N1-H4$_{\text{(-NH3+)}}$···O5$_{\text{(-SO4)}}$ & (N2-H12$_{\text{(-NH3+)}}$···Cl1)$_2$<br>*(N1-H5$_{\text{(-NH3+)}}$···O3$_{\text{(-NO3-)}}$)$_2$ & N2-H12$_{\text{(-NH3+)}}$···Cl1<br>*(N1-H5$_{\text{(-NH3+)}}$···O3$_{\text{(-NO3-)}}$)$_2$ & N2-H14$_{\text{(-NH3+)}}$···O6A$_{\text{(-SO4)}}$<br>*(N1-H5$_{\text{(-NH3+)}}$···O3$_{\text{(-NO3-)}}$)$_2$ & N2-H14$_{\text{(-NH3+)}}$···O5$_{\text{(-SO4)}}$
	$D^{2}_{3}(12)$	O2-H1$_{\text{(-COOH)}}$···O6$_{\text{(-SO4)}}$ & (N2-H12$_{\text{(-NH3+)}}$···Cl1)$_2$
	$D^{2}_{3}(14)$	O2-H1$_{\text{(-COOH)}}$···O6$_{\text{(-SO4)}}$ & O2-H1$_{\text{(-COOH)}}$···O6A$_{\text{(-SO4)}}$ & (N2-H12$_{\text{(-NH3+)}}$···Cl1)$_2$
	$D^{3}_{3}(8)$	*N1-H4$_{\text{(-NH3+)}}$···O5$_{\text{(-SO4)}}$ & N1-H4$_{\text{(-NH3+)}}$···O5A$_{\text{(-SO4)}}$ & N1-H3$_{\text{(-NH3+)}}$···Cl1<br>*(N1-H5$_{\text{(-NH3+)}}$···O3$_{\text{(-NO3-)}}$)$_2$ & N1-H4$_{\text{(-NH3+)}}$···O5$_{\text{(-SO4)}}$ & (N1-H4$_{\text{(-NH3+)}}$···O5$_{\text{(-SO4)}}$)$_2$<br>*(N2-H12$_{\text{(-NH3+)}}$···Cl1)$_2$ & N2-H14$_{\text{(-NH3+)}}$···O6A$_{\text{(-SO4)}}$<br>*N2-H14$_{\text{(-NH3+)}}$···O5$_{\text{(-SO4)}}$ & N2-H14$_{\text{(-NH3+)}}$···O5A$_{\text{(-SO4)}}$ & N2-H12$_{\text{(-NH3+)}}$···Cl1<br>*N2-H13$_{\text{(-NH3+)}}$···O4$_{\text{(-NO3-)}}$ & N2-H13$_{\text{(-NH3+)}}$···O4A$_{\text{(-NO3-)}}$ & N2-H14$_{\text{(-NH3+)}}$···O6A$_{\text{(-SO4)}}$
	$D^{3}_{3}(10)$	*C3-H6$_{\text{(-CH2)}}$···O4$_{\text{(-NO3-)}}$ & C3-H6$_{\text{(-CH2)}}$···O4A$_{\text{(-NO3-)}}$ & N1-H3$_{\text{(-NH3+)}}$···Cl1<br>*N1-H4$_{\text{(-NH3+)}}$···O5$_{\text{(-SO4)}}$ & C3-H6$_{\text{(-CH2)}}$···O4A$_{\text{(-NO3-)}}$ & N1-H4$_{\text{(-NH3+)}}$···O5A$_{\text{(-SO4)}}$<br>*N1-H4$_{\text{(-NH3+)}}$···O5$_{\text{(-SO4)}}$ & C3-H6$_{\text{(-CH2)}}$···O4$_{\text{(-NO3-)}}$ & C3-H6$_{\text{(-CH2)}}$···O4A$_{\text{(-NO3-)}}$
	$D^{3}_{3}(11)$	*(O2-H1$_{\text{(-COOH)}}$···O6$_{\text{(-SO4)}}$)$_2$ & N1-H3$_{\text{(-NH3+)}}$···Cl1<br>*O2-H1$_{\text{(-COOH)}}$···O6$_{\text{(-SO4)}}$ & O2-H1$_{\text{(-COOH)}}$···O6A$_{\text{(-SO4)}}$ & N1-H5$_{\text{(-NH3+)}}$···O3$_{\text{(-NO3-)}}$<br>*O2-H1$_{\text{(-COOH)}}$···O6$_{\text{(-SO4)}}$ & O2-H1$_{\text{(-COOH)}}$···O6A$_{\text{(-SO4)}}$ & C3-H6$_{\text{(-CH2)}}$···O4A$_{\text{(-NO3-)}}$<br>*O2-H1$_{\text{(-COOH)}}$···O6A$_{\text{(-SO4)}}$ & C3-H6$_{\text{(-CH2)}}$···O4$_{\text{(-NO3-)}}$ & C3-H6$_{\text{(-CH2)}}$···O4A$_{\text{(-NO3-)}}$<br>*(N1-H3$_{\text{(-NH3+)}}$···Cl1)$_2$ & N2-H13$_{\text{(-NH3+)}}$···O4$_{\text{(-NO3-)}}$<br>*C3-H6$_{\text{(-CH2)}}$···O4$_{\text{(-NO3-)}}$ & C3-H6$_{\text{(-CH2)}}$···O4A$_{\text{(-NO3-)}}$ & N2-H12$_{\text{(-NH3+)}}$···Cl1

		*C3-H16$_{(-CH2)}$···O4$_{(-NO3-)}$ & C3-H6$_{(-CH2)}$···O4A$_{(-NO3-)}$& N2-H14$_{(-NH3+)}$···O6A$_{(-SO4)}$<br>*N2-H14$_{(-NH3+)}$···O6$_{(-SO4)}$ & N2-H14$_{(-NH3+)}$···O6A$_{(-SO4)}$ & C3-H6$_{(-CH2)}$···O4A$_{(-NO3-)}$
	$D^3_3(13)$	*N1-H3$_{(-NH3+)}$···Cl1 & N2-H13$_{(-NH3+)}$···O4$_{(-NO3-)}$ & N2-H13$_{(-NH3+)}$···O4A$_{(-NO3-)}$<br>*N2-H14$_{(-NH3+)}$···O6$_{(-SO4)}$ & N2-H14$_{(-NH3+)}$···O6A$_{(-SO4)}$ & N1-H3$_{(-NH3+)}$···Cl1<br>*N1-H4$_{(-NH3+)}$···O5$_{(-SO4)}$ & N1-H4$_{(-NH3+)}$···O5A$_{(-SO4)}$ & N2-H12$_{(-NH3+)}$···Cl1<br>*N1-H4$_{(-NH3+)}$···O5$_{(-SO4)}$ & N1-H4$_{(-NH3+)}$···O5A$_{(-SO4)}$ & N2-H13$_{(-NH3+)}$···O4$_{(-NO3-)}$<br>*N1-H4$_{(-NH3+)}$···O5$_{(-SO4)}$ & N2-H13$_{(-NH3+)}$···O4$_{(-NO3-)}$ & N2-H13$_{(-NH3+)}$···O4$_{(-NO3-)}$<br>*N1-H5$_{(-NH3+)}$···O3$_{(-NO3-)}$ & N2-H14$_{(-NH3+)}$···O6$_{(-SO4)}$ & N2-H14$_{(-NH3+)}$···O6A$_{(-SO4)}$<br>*N1-H5$_{(-NH3+)}$···O3$_{(-NO3-)}$ & N2-H14$_{(-NH3+)}$···O5$_{(-SO4)}$ & N2-H14$_{(-NH3+)}$···O5A$_{(-SO4)}$
	$D^3_3(14)$	*O2-H1$_{(-COOH)}$···O6$_{(-SO4)}$ & O2-H1$_{(-COOH)}$···O6A$_{(-SO4)}$ & N2-H13$_{(-NH3+)}$···O4$_{(-NO3-)}$<br>*O2-H1$_{(-COOH)}$···O6A$_{(-SO4)}$ & N2-H13$_{(-NH3+)}$···O4$_{(-NO3-)}$ & N2-H13$_{(-NH3+)}$···O4A$_{(-NO3-)}$
**GOTFAY**	$C(7)$	C12-H3$_{(-CHarom.)}$ ···O11$_{(-NO2)}$
	$C^1_2(7)$	*O16-H17$_{(-COOH)}$ ···O10$_{(-NO2)}$ & C15-H5$_{(-CH2)}$ ···O10$_{(-NO2)}$<br>*N8-H10$_{(-NH3+)}$ ···O8$_{(-C=O)}$ & C15-H5$_{(-CH2)}$ ···O8$_{(-C=O)}$
	$C^1_2(9)$	*N7-H8$_{(-NH3+)}$ ···O3$_{(-NO2)}$ & N8-H18$_{(-NH3+)}$ ···O3$_{(-NO2)}$<br>*N7-H16$_{(-NH3+)}$ ···O8$_{(-C=O)}$ & N8-H10$_{(-NH3+)}$ ···O8$_{(-C=O)}$
	$C^2_2(7)$	C13-H7$_{(-CH)}$ ···O12$_{(-NO2)}$ & C15-H6$_{(-CH2)}$ ···O14$_{(-NO2)}$
	$C^2_2(8)$	*N7-H8$_{(-NH3+)}$ ···O1$_{(-C=O)}$ & N7-H15$_{(-NH3+)}$ ···O4$_{(-NO2)}$<br>*N8-H13$_{(-NH3+)}$ ···O1$_{(-C=O)}$ & N8-H18$_{(-NH3+)}$ ···O3$_{(-NO2)}$
	$C^2_2(10)$	N7-H8$_{(-NH3+)}$ ···O3$_{(-NO2)}$ & N7-H15$_{(-NH3+)}$ ···O4$_{(-NO2)}$
	$C^2_2(11)$	*O16-H17$_{(-COOH)}$···O10$_{(-NO2)}$ & N7-H16$_{(-NH3+)}$ ···O8$_{(-C=O)}$<br>* O16-H17$_{(-COOH)}$···O10$_{(-NO2)}$ & C15-H5$_{(-CH2)}$ ···O8$_{(-C=O)}$<br>*N7-H16$_{(-NH3+)}$ ···O8$_{(-C=O)}$ & C13-H7$_{(-CH)}$···O12$_{(-NO2)}$<br>*N8-H10$_{(-NH3+)}$ ···O8$_{(-C=O)}$ & C15-H5$_{(-CH2)}$ ···O10$_{(-NO2)}$
	$C^2_2(12)$	*O16-H17$_{(-COOH)}$···O10$_{(-NO2)}$ & C13-H7$_{(-CH)}$···O12$_{(-NO2)}$<br>*N7-H16$_{(-NH3+)}$ ···O8$_{(-C=O)}$ & C15-H6$_{(-CH2)}$ ···O14$_{(-NO2)}$
	$C^2_2(13)$	*O16-H17$_{(-COOH)}$···O10$_{(-NO2)}$ & N7-H16$_{(-NH3+)}$ ···O11$_{(-NO2)}$<br>*O16-H17$_{(-COOH)}$···O10$_{(-NO2)}$ & C15-H6$_{(-CH2)}$ ···O14$_{(-NO2)}$<br>*N7-H8$_{(-NH3+)}$ ···O1$_{(-C=O)}$ & N8-H18$_{(-NH3+)}$ ···O3$_{(-NO2)}$<br>*(N7-H8$_{(-NH3+)}$ ···O3$_{(-NO2)}$)$_2$ & N8-H13$_{(-NH3+)}$ ···O1$_{(-C=O)}$<br>*N7-H16$_{(-NH3+)}$ ···O11$_{(-NO2)}$ & N8-H10$_{(-NH3+)}$ ···O8$_{(-C=O)}$<br>*N8-H10$_{(-NH3+)}$ ···O8$_{(-C=O)}$ & C15-H6$_{(-CH2)}$ ···O14$_{(-NO2)}$
	$C^2_2(14)$	*O16-H17$_{(-COOH)}$···O10$_{(-NO2)}$ & N8-H10$_{(-NH3+)}$ ···O8$_{(-C=O)}$<br>*N7-H8$_{(-NH3+)}$ ···O1$_{(-C=O)}$ & C14-H12$_{(-CH2)}$···O7$_{(-NO2)}$<br>*N7-H8$_{(-NH3+)}$ ···O3$_{(-NO2)}$ & C14-H12$_{(-CH2)}$···O7$_{(-NO2)}$
	$R^2_1(6)$	*N7-H8$_{(-NH3+)}$ ···O1$_{(-C=O)}$ & N7-H8$_{(-NH3+)}$ ···O3$_{(-NO2)}$<br>*N7-H16$_{(-NH3+)}$ ···O8$_{(-C=O)}$ & N7-H16$_{(-NH3+)}$ ···O11$_{(-NO2)}$
	$R^2_2(7)$	C14-H12$_{(-CH2)}$···O7$_{(-NO2)}$ & C16-H9$_{(-CH2)}$ ···O6$_{(-NO2)}$
	$R^2_2(10)$	N7-H16$_{(-NH3+)}$ ···08$_{(-C=O)}$ & C15-H5$_{(-CH2)}$ ···O10$_{(-NO2)}$
	$R^2_2(12)$	N7-H16$_{(-NH3+)}$ ···011$_{(-NO2)}$ & C15-H5$_{(-CH2)}$ ···O10$_{(-NO2)}$
	$D(2)$	*C6-H2$_{(-CHarom.)}$ ···O9$_{(-NO2)}$<br>*O16-H17$_{(-COOH)}$···O10$_{(-NO2)}$<br>*N7-H8$_{(-NH3+)}$ ···O1$_{(-C=O)}$<br>*N7-H8$_{(-NH3+)}$ ···O3$_{(-NO2)}$<br>*N7-H15$_{(-NH3+)}$ ···O4$_{(-NO2)}$<br>*N7-H16$_{(-NH3+)}$ ···O8$_{(-C=O)}$<br>*N7-H16$_{(-NH3+)}$ ···O11$_{(-NO2)}$<br>*N8-H10$_{(-NH3+)}$ ···O8$_{(-C=O)}$<br>*N8-H13$_{(-NH3+)}$ ···O4$_{(-NO2)}$<br>*N8-H18$_{(-NH3+)}$ ···O3$_{(-NO2)}$<br>*C13-H7$_{(-CH)}$···O12$_{(-NO2)}$<br>*C16-H9$_{(-CH2)}$ ···O6$_{(-NO2)}$<br>*C14-H12$_{(-CH2)}$···O7$_{(-NO2)}$<br>*C15-H15$_{(-CH2)}$ ···O8$_{(-C=O)}$<br>*C15-H5$_{(-CH2)}$ ···O10$_{(-NO2)}$<br>*C15-H6$_{(-CH2)}$ ···O14$_{(-NO2)}$
	$D^2_2(5)$	*N7-H16$_{(-NH3+)}$···O11$_{(-NO2)}$ & C6-H2$_{(-CHarom.)}$ ···O9$_{(-NO2)}$<br>*N7-H8$_{(-NH3+)}$ ···O1$_{(-C=O)}$ & N7-H16$_{(-NH3+)}$ ···O8$_{(-C=O)}$<br>*N7-H8$_{(-NH3+)}$ ···O3$_{(-NO2)}$ & N7-H16$_{(-NH3+)}$ ···O8$_{(-C=O)}$
	$D^2_2(6)$	*N7-H8$_{(-NH3+)}$ ···O1$_{(-C=O)}$ & C13-H7$_{(-CH)}$···O12$_{(-NO2)}$<br>*N7-H8$_{(-NH3+)}$ ···O3$_{(-NO2)}$ & C13-H7$_{(-CH)}$···O12$_{(-NO2)}$
	$D^2_2(7)$	*N7-H8$_{(-NH3+)}$ ···O1$_{(-C=O)}$ & C6-H2$_{(-CHarom.)}$ ···O9$_{(-NO2)}$<br>*N7-H15$_{(-NH3+)}$ ···O4$_{(-NO2)}$ & C6-H2$_{(-CHarom.)}$ ···O9$_{(-NO2)}$<br>*N7-H8$_{(-NH3+)}$ ···O1$_{(-C=O)}$ & C15-H5$_{(-CH2)}$ ···O8$_{(-C=O)}$<br>*N7-H8$_{(-NH3+)}$ ···O3$_{(-NO2)}$ & C15-H5$_{(-CH2)}$ ···O8$_{(-C=O)}$

	$D^2_2(8)$	*O16-H17$_{(-COOH)}$···O10$_{(-NO2)}$ & N7-H8$_{(-NH3+)}$ ···O1$_{(-C=O)}$<br>*N7-H16$_{(-NH3+)}$ ···O8$_{(-C=O)}$ & C16-H9$_{(-CH2)}$ ···O6$_{(-NO2)}$
	$D^2_2(9)$	*C6-H2$_{(-CHarom.)}$···O11$_{(-NO2)}$ & O16-H17$_{(-COOH)}$···O10$_{(-NO2)}$<br>*N7-H8$_{(-NH3+)}$ ···O3$_{(-NO2)}$ & C6-H2$_{(-CHarom.)}$···O9$_{(-NO2)}$<br>*O16-H17$_{(-COOH)}$···O10$_{(-NO2)}$ & C16-H9$_{(-CH2)}$ ···O6$_{(-NO2)}$<br>*N7-H16$_{(-NH3+)}$ ···O8$_{(-C=O)}$ & C14-H12$_{(-CH2)}$···O7$_{(-NO2)}$
	$D^2_2(10)$	*O16-H17$_{(-COOH)}$···O10$_{(-NO2)}$ & N8-H13$_{(-NH3+)}$ ···O1$_{(-C=O)}$<br>*O16-H17$_{(-COOH)}$···O10$_{(-NO2)}$ & C14-H12$_{(-CH2)}$···O7$_{(-NO2)}$<br>*N7-H8$_{(-NH3+)}$ ···O1$_{(-C=O)}$ & N8-H10$_{(-NH3+)}$ ···O8$_{(-C=O)}$<br>*N7-H8$_{(-NH3+)}$ ···O3$_{(-NO2)}$ & N8-H10$_{(-NH3+)}$ ···O8$_{(-C=O)}$<br>*N7-H16$_{(-NH3+)}$ ···O11$_{(-NO2)}$ & N8-H13$_{(-NH3+)}$ ···O1$_{(-C=O)}$
	$D^2_2(11)$	O16-H17$_{(-COOH)}$···O10$_{(-NO2)}$ & N8-H18$_{(-NH3+)}$ ···O3$_{(-NO2)}$
	$D^2_3(10)$	(N7-H16$_{(-NH3+)}$···O11$_{(-NO2)}$)$_2$ & C12-H3$_{(-CHarom.)}$···O11$_{(-NO2)}$
	$D^3_3(12)$	*C6-H2$_{(-CHarom.)}$···O9$_{(-NO2)}$ & C12-H3$_{(-CHarom.)}$···O11$_{(-NO2)}$ & C6-H2$_{(-CHarom.)}$···O9$_{(-NO2)}$<br>*(N7-H16$_{(-NH3+)}$···O8$_{(-C=O)}$)$_2$ & C12-H3$_{(-CHarom.)}$···O11$_{(-NO2)}$<br>*(N8-H10$_{(-NH3+)}$···O8$_{(-C=O)}$)$_2$ & C12-H3$_{(-CHarom.)}$···O11$_{(-NO2)}$<br>*C12-H3$_{(-CHarom.)}$···O11$_{(-NO2)}$ & (C15-H5$_{(-CH2)}$ ···O8$_{(-C=O)}$)$_2$
	$D^3_3(14)$	*(O6-H17$_{(-NO2)}$)···O10$_{(-NO2)}$)$_2$ & C12-H3$_{(-CHarom.)}$···O11$_{(-NO2)}$<br>*C12-H3$_{(-CHarom.)}$···O11$_{(-NO2)}$ & (C13-H7$_{(-CH)}$···O12$_{(-NO2)}$)$_2$
**EXOFAY**	$D(2)$	*O1-H2$_{(-COOH)}$···Cl1<br>*N1-H9$_{(-NH3+)}$···Cl1<br>*N1-H11$_{(-NH3+)}$···Cl1
	$C(4)$	N2-H12$_{(-NH)}$ **ornithine** ···O4$_{(-O=C)}$**FMOC**
	$C(5)$	C3-H4$_{(-CH2)}$···O1$_{(-COOH)}$ **ornithine**
	$C(13)$	C3-H3$_{(-CH2)}$ **ornithine** ···C13 $_{(-Carom.)}$**FMOC**
level 2	$C^1_2(4)$	N1-H9$_{(-NH3+)}$···Cl1 & N1-H11$_{(-NH3+)}$···Cl1
	$C^1_2(10)$	*(O1-H2$_{(-COOH)}$···Cl1)$_2$ & N1-H9$_{(-NH3+)}$···Cl1<br>*(O1-H2$_{(-COOH)}$···Cl1)$_2$ & N1-H11$_{(-NH3+)}$···Cl1
	$C^2_2(11)$	N2-H12$_{(-NH)}$ **ornithine** ···O4$_{(-O=C)}$**FMOC** & C3-H4$_{(-CH2)}$···O1$_{(-COOH)}$ **ornithine**
	$R^2_2(11)$	N2-H12$_{(-NH)}$ **ornithine** ···O4$_{(-O=C)}$**FMOC** & C3-H4$_{(-CH2)}$···O1$_{(-COOH)}$ **ornithine**
	$D^2_2(5)$	*N1-H9$_{(-NH3+)}$···Cl1 & N1-H10$_{(-NH3+)}$···O5$_{(-C-O-C)}$<br>*N1-H10$_{(-NH3+)}$···O5$_{(-C-O-C)}$ & N1-H11$_{(-NH3+)}$···Cl1
	$D^2_2(11)$	O1-H2$_{(-COOH)}$···Cl1 & N1-H10$_{(-NH3+)}$···O5$_{(-C-O-C)}$
	$D^3_3(10)$	(O1-H2$_{(-COOH)}$···Cl1)$_2$ & C3-H4$_{(-CH2)}$···O1$_{(-COOH)}$ **ornithine**
	$D^3_3(15)$	(O1-H2$_{(-COOH)}$···Cl1)$_2$ & N2-H12$_{(-NH)}$ **ornithine** ···O4$_{(-O=C)}$**FMOC**
	$D^3_3(16)$	*(N1-H9$_{(-NH3+)}$···Cl1)$_2$ & C3-H4$_{(-CH2)}$···O1$_{(-COOH)}$ **ornithine**<br>*(N1-H10$_{(-NH3+)}$···O5$_{(-C-O-C)}$))$_2$ & C3-H4$_{(-CH2)}$···O1$_{(-COOH)}$ **ornithine**<br>*(N1-H11$_{(-NH3+)}$···Cl1)$_2$ & C3-H4$_{(-CH2)}$···O1$_{(-COOH)}$ **ornithine**
	$D^3_3(19)$	*(N1-H9$_{(-NH3+)}$···Cl1)$_2$ & N2-H12$_{(-NH)}$ **ornithine** ···O4$_{(-O=C)}$**FMOC**<br>*(N1-H10$_{(-NH3+)}$···O5$_{(-C-O-C)}$)$_2$ & N2-H12$_{(-NH)}$ **ornithine** ···O4$_{(-O=C)}$**FMOC**<br>*(N1-H11$_{(-NH3+)}$···Cl1)$_2$ & N2-H12$_{(-NH)}$ **ornithine** ···O4$_{(-O=C)}$**FMOC**
**IHEPES**	C(9)	*N2-H3···Cl2<br>*N2-H4···Cl2
	$\mathrm{C}^2_1(4)$	*N2-H3···Cl2 & N2-H3···Cl2A<br>*N2-H4···Cl2A & N2-H4···Cl2
	C(7)	C5-H11···F1
	D(2)	*O3-H13···Cl1<br>*O3-H14···O1<br>*N2-H5···O3<br>*C4-H8···O3<br>*C6-H12···O3
Level 2	$\mathrm{C}^1_2(4)$	N2-H3···Cl2 & N2-H4···Cl2
	$\mathrm{C}^1_2(6)$	N2-H5···O3 & C4-H8···O3
	$\mathrm{C}^1_2(7)$	C4-H8···O3 & C6-H12···O3
	$\mathrm{C}^1_2(9)$	(N2-H5···O3)$_2$ & C6-H12···O3
	$\mathrm{C}^2_2(8)$	*O3-H13···Cl1 & O3-H14···O1<br>*O3-H14···O1 & C4-H8···O3<br>*O3-H13···Cl1 & C6-H12···O3
	$\mathrm{C}^2_2(9)$	O3-H13···Cl1 & C4-H8···O3
	$\mathrm{C}^2_2(10)$	*O3-H14···O1 & N2-H5···O3<br>*N2-H3···Cl2 & C5-H11···F1<br>*N2-H4···Cl2A & C5-H11···F1
	$\mathrm{C}^2_2(11)$	*N2-H3···Cl2 & N2-H4···Cl2<br>*N2-H3···Cl2 & N2-H4···Cl2A
	$\mathrm{C}^2_2(16)$	*N2-H3···Cl2 & C5-H11···F1

		*N2-H4···Cl2A & C5-H11···F1
	$C^{2}_{2}(18)$	N2-H3···Cl2 & N2-H4···Cl2A
	$C^{3}_{2}(15)$	*N2-H3···Cl2 & N2-H3···Cl2A & C5-H11···F1<br>(N1-H4···Cl2A)$_2$ & C5-H11···F1
	$C^{3}_{3}(15)$	*N2-H3···Cl2 & N2-H3···Cl2A & N2-H4···Cl2A<br>*N2-H3···Cl2 & (N2-H4···Cl2A)$_2$<br>*N2-H3···Cl2 & N2-H3···Cl2A & C5-H11···F1<br>*N2-H4···Cl2 & N2-H4···Cl2A & C5-H11···F1
	$R^{2}_{2}(4)$	N2-H4···Cl2A & N2-H3···Cl2A
	$R^{2}_{2}(6)$	N2-H3···Cl2 & N2-H4···Cl2A
	$R^{2}_{2}(7)$	O3-H14···O1 & C6-H12···O3
	$R^{2}_{2}(11)$	O3-H13···Cl1 & N2-H5···O3
	$R^{3}_{3}(8)$	*N2-H3···Cl2 & N2-H3···Cl2A & N2-H4···Cl2A<br>*(N2-H4···Cl2A)$_2$ & N2-H3···Cl2
	$R^{4}_{4}(12)$	*N2-H3···Cl2 & N2-H3···Cl2A & (N2-H4···Cl2A)$_2$
	$R^{5}_{3}(19)$	*(N2-H3···Cl2A)$_2$ & C5-H11···F1<br>*(N2-H4···Cl2A)$_2$ & C5-H11···F1
	$R^{5}_{4}(19)$	*(N2-H3···Cl2A)$_2$ & N2-H3···Cl2 & C5-H11···F1
	$D^{3}_{3}(12)$	(C4-H8···O3)$_2$ & C5-H11···F1<br>* C5-H11···F1 & (C6-H12···O3)$_2$
	$D^{3}_{3}(14)$	*(O3-H13···Cl1)$_2$ & N2-H3···Cl2<br>*(O3-H13···Cl1)$_2$ & N2-H4···Cl2A<br>*(O3-H14···O1)$_2$ & C5-H11···F1<br>*(N2-H5···O3)$_2$ & N2-H3···Cl2<br>*(N2-H5···O3)$_2$ & C5-H11···F1<br>*N2-H3···Cl2 & (C4-H8···O3)$_2$<br>*N2-H4···Cl2 & (C4-H8···O3)$_2$<br>*N2-H4···Cl2A & (N2-H5···O3)$_2$
	$D^{3}_{3}(15)$	*(O3-H14···O1)$_2$ & N2-H3···Cl2<br>*(O3-H14···O1)$_2$ & N2-H4···Cl2A
	$D^{3}_{3}(16)$	*(O3-H13···Cl1)$_2$ & C5-H11···F1<br>*N2-H3···Cl2 & (C6-H12···O3)$_2$<br>*N2-H4···Cl2A & (C6-H12···O3)$_2$
**JADGED**	*C*(2)	C8-H22$_{(-CH2)}$ ···O12$_{(-C=O)}$
	C(4)	C7-H20$_{(-CH)}$ ···O3$_{(-COO-)}$<br>C19-H35$_{(-CH2)}$ ···O13$_{(-COOH)}$
	C(5)	N1-H2$_{(-NH3+)}$ ···O1$_{(-COO-)}$
	C(6)	C4-H10$_{(-CH2)}$ ···O1$_{(-COO-)}$
	D(2)	*N1-H1$_{(-NH3+)}$ ···O5$_{(-COO-)}$<br>*N1-H3$_{(-NH3+)}$ ···O5$_{(-COO-)}$<br>*N1-H3$_{(-NH3+)}$ ···O7$_{(-C=O)}$<br>*N2-H4$_{(-NH3+)}$ ···O3$_{(-COO-)}$<br>*N2-H5$_{(-NH3+)}$ ···O8$_{(-COOH)}$<br>*N2-H6$_{(-NH3+)}$ ···O11$_{(-COOH)}$<br>*C2-H7$_{(-CH)}$ ···O5$_{(-COO-)}$<br>*C3-H9$_{(-CH2)}$···O7$_{(-C=O)}$<br>*C5-H12$_{(-CH2)}$···O7$_{(-C=O)}$<br>*C5-H12$_{(-CH2)}$···O9$_{(-COOH)}$<br>*C5-H13$_{(-CH2)}$···O11$_{(-COOH)}$<br>*N3-H14$_{(-NH3+)}$ ···O10$_{(-COOH)}$<br>*N3-H15$_{(-NH3+)}$ ···O10$_{(-COOH)}$<br>*N3-H15$_{(-NH3+)}$ ···O12$_{(-C=O)}$<br>*N3-H16$_{(-NH3+)}$ ···O10$_{(-COOH)}$<br>*N4-H17$_{(-NH3+)}$ ···O1$_{(-COO-)}$<br>*N4-H18$_{(-NH3+)}$ ···O6$_{(-COO-)}$<br>*N4-H19$_{(-NH3+)}$ ···O14$_{(-COOH)}$<br>*C10-H26$_{(-CH2)}$···O14$_{(-COOH)}$<br>*O9-H27$_{(-COOH)}$···O4$_{(-COO-)}$<br>*O13-H32$_{(-COOH)}$···O2$_{(-COO-)}$
level 2	$C^{1}_{2}(4)$	*N1-H3$_{(-NH3+)}$ ···O5$_{(-COO-)}$ & N1-H1$_{(-NH3+)}$ ···O5$_{(-COO-)}$<br>*N3-H14$_{(-NH3+)}$ ···O10$_{(-COOH)}$ & N3-H15$_{(-NH3+)}$ ···O10$_{(-COOH)}$<br>*N3-H16$_{(-NH3+)}$ ···O10$_{(-COOH)}$ & N3-H14$_{(-NH3+)}$ ···O10$_{(-COOH)}$<br>*N3-H16$_{(-NH3+)}$ ···O10$_{(-COOH)}$ & N3-H15$_{(-NH3+)}$ ···O10$_{(-COOH)}$
	$C^{1}_{2}(5)$	*N1-H1$_{(-NH3+)}$ ···O5$_{(-COO-)}$ & C2-H7$_{(-CH)}$···O5$_{(-COO-)}$<br>*N1-H3$_{(-NH3+)}$ ···O5$_{(-COO-)}$ & C2-H7$_{(-CH)}$···O5$_{(-COO-)}$

		*N2-H6$_{(-NH3+)}$ ···O11$_{(-COOH)}$ & C5-H13$_{(-CH2)}$···O11$_{(-COOH)}$<br>*N4-H19$_{(-NH3+)}$ ···O14$_{(-COOH)}$ & C10-H26$_{(-CH2)}$···O14$_{(-COOH)}$
	$C^2_2(7)$	*N1-H3$_{(-NH3+)}$ ···O7$_{(-C=O)}$ & N1-H1$_{(-NH3+)}$···O5$_{(-COO-)}$<br>*N2-H5$_{(-NH3+)}$ ···O8$_{(-COOH)}$ & C5-H12$_{(-CH2)}$···O9$_{(-COOH)}$<br>*N3-H14$_{(-NH3+)}$ ···O10$_{(-COOH)}$ & N3-H15$_{(-NH3+)}$···O12$_{(-C=O)}$<br>*N3-H15$_{(-NH3+)}$···O13$_{(-COOH)}$ & N3-H16$_{(-NH3+)}$···O10$_{(-COOH)}$
	$C^2_2(8)$	*N1-H3$_{(-NH3+)}$ ···O7$_{(-C=O)}$ & C2-H7$_{(-CH)}$···O5$_{(-COO-)}$<br>*C2-H7$_{(-CH)}$···O5$_{(-COO-)}$ & C3-H9$_{(-CH2)}$ ···O7$_{(-C=O)}$
	$C^2_2(9)$	*N1-H1$_{(-NH3+)}$ ···O5$_{(-COO-)}$ & C3-H9$_{(-CH2)}$ ···O7$_{(-C=O)}$<br>*N3-H14$_{(-NH3+)}$···O10$_{(-COOH)}$ & C8-H22$_{(-CH2)}$···O12$_{(-C=O)}$
	$C^2_2(10)$	*N2-H5$_{(-NH3+)}$···O8$_{(-COOH)}$ & C5-H12$_{(-CH2)}$···O7$_{(-C=O)}$<br>*C2-H7$_{(-CH)}$···O5$_{(-COO-)}$ & C5-H12$_{(-CH2)}$···O7$_{(-C=O)}$
	$C^2_2(11)$	*(N1-H1$_{(-NH3+)}$···O5$_{(-COO-)}$)$_2$ & C5-H12$_{(-CH2)}$···O7$_{(-C=O)}$<br>*N1-H4$_{(-NH3+)}$···O1$_{(-COO-)}$ & C4-H10$_{(-CH2)}$···O1$_{(-COO-)}$
	$C^2_2(12)$	*N2-H5$_{(-NH3+)}$···O8$_{(-COOH)}$ & C3-H9$_{(-CH2)}$ ···O7$_{(-C=O)}$<br>*N14-H19$_{(-NH3+)}$···O14$_{(-COOH)}$ & C8-H22$_{(-CH2)}$···O12$_{(-C=O)}$
	$C^2_2(13)$	C2-H7$_{(-CH)}$···O5$_{(-COO-)}$ & C5-H12$_{(-CH2)}$···O9$_{(-COOH)}$
	$C^2_2(14)$	*N1-H1$_{(-NH3+)}$···O5$_{(-COO-)}$ & C5-H12$_{(-CH2)}$···O9$_{(-COOH)}$<br>*N1-H3$_{(-NH3+)}$···O7$_{(-C=O)}$ & N2-H5$_{(-NH3+)}$···O8$_{(-COOH)}$<br>*N3-H14$_{(-NH3+)}$···O10$_{(-COOH)}$ & C10-H26$_{(-CH2)}$···O14$_{(-COOH)}$<br>*N3-H15$_{(-NH3+)}$···O12$_{(-C=O)}$ & N4-H19$_{(-NH3+)}$···O14$_{(-COOH)}$<br>*C10-H26$_{(-CH2)}$···O14$_{(-COOH)}$ & N3-H16$_{(-NH3+)}$···O10$_{(-COOH)}$
	$C^2_2(15)$	*N1-H1$_{(-NH3+)}$···O5$_{(-COO-)}$ & N2-H5$_{(-NH3+)}$···O8$_{(-COOH)}$<br>*N1-H3$_{(-NH3+)}$···O5$_{(-COO-)}$ & N2-H5$_{(-NH3+)}$···O8$_{(-COOH)}$<br>*O13-H32$_{(-COOH)}$···O2$_{(-COO-)}$ & C5-H13$_{(-CH2)}$···O11$_{(-COOH)}$<br>*N3-H14$_{(-NH3+)}$···O10$_{(-COOH)}$ & N4-H19$_{(-NH3+)}$···O14$_{(-COOH)}$<br>*N3-H15$_{(-NH3+)}$···O10$_{(-COOH)}$ & N4-H19$_{(-NH3+)}$···O14$_{(-COOH)}$
	$C^2_2(16)$	O9$_{(COOH)}$-H27···O4$_{(-COO-)}$ & N4-H18$_{(-NH3+)}$···O6$_{(-COO-)}$
	$R^1_2(6)$	*N1-H3$_{(-NH3+)}$···O7$_{(-C=O)}$ & C3-H9$_{(-CH2)}$ ···O7$_{(-C=O)}$<br>*C3-H9$_{(-CH2)}$ ···O7$_{(-C=O)}$ & C5-H12$_{(-CH2)}$···O7$_{(-C=O)}$<br>*N3-H15$_{(-NH3+)}$···O12$_{(-C=O)}$ & C8-H22$_{(-CH2)}$···O12$_{(-C=O)}$
	$R^1_2(7)$	*N1-H2$_{(-NH3+)}$···O1$_{(-COO-)}$ & C4-H1O$_{(-CH2)}$ ···O1$_{(-COO-)}$<br>*C5-H12$_{(-CH2)}$···O9$_{(-COOH)}$ & C5-H12$_{(-CH2)}$···O7$_{(-C=O)}$
	$R^1_2(8)$	N1-H3$_{(-NH3+)}$···O7$_{(-C=O)}$ & C5-H12$_{(-CH2)}$···O7$_{(-C=O)}$
	$R^2_1(5)$	*N1-H3$_{(-NH3+)}$···O5$_{(-COO-)}$ & N1-H3$_{(-NH3+)}$···O7$_{(-C=O)}$<br>*N3-H15$_{(-NH3+)}$···O12$_{(-C=O)}$ & N3-H15$_{(-NH3+)}$···O10$_{(-COOH)}$
	$R^2_2(7)$	C3-H9$_{(-CH2)}$ ···O7$_{(-C=O)}$ & C5-H13$_{(-CH2)}$···O11$_{(-COOH)}$
	$R^2_2(9)$	*N1-H3$_{(-NH3+)}$···O5$_{(-COO-)}$ & C3-H9$_{(-CH2)}$ ···O7$_{(-C=O)}$<br>*N3-H15$_{(-NH3+)}$···O10$_{(-COOH)}$ & C8-H22$_{(-CH2)}$···O12$_{(-C=O)}$
	$R^2_2(11)$	*N1-H3$_{(-NH3+)}$···O5$_{(-COO-)}$ & C5-H12$_{(-CH2)}$···O7$_{(-C=O)}$<br>*C3-H9$_{(-CH2)}$ ···O7$_{(-C=O)}$ & C5-H12$_{(-CH2)}$···O9$_{(-COOH)}$<br>*C8-H22$_{(-CH2)}$···O12$_{(-C=O)}$ & C10-H26$_{(-CH2)}$···O14$_{(-COOH)}$
	$R^2_2(13)$	*N1-H3$_{(-NH3+)}$···O7$_{(-C=O)}$ & C5-H12$_{(-CH2)}$···O9$_{(-COOH)}$<br>*N3-H15$_{(-NH3+)}$···O12$_{(-C=O)}$ & C10-H26$_{(-CH2)}$···O14$_{(-COOH)}$
	$R^2_2(14)$	*N1-H3$_{(-NH3+)}$···O5$_{(-COO-)}$ & C5-H12$_{(-CH2)}$···O9$_{(-COOH)}$<br>*N2-H5$_{(-NH3+)}$···O8$_{(-COOH)}$ & C2-H7$_{(-CH)}$···O5$_{(-COO-)}$<br>*N3-H15$_{(-NH3+)}$···O10$_{(-COOH)}$ & C10-H26$_{(-CH2)}$···O14$_{(-COOH)}$
	$R^2_2(15)$	N3-H16$_{(-NH3+)}$···O10$_{(-COOH)}$ & N4-H19$_{(-NH3+)}$···O14$_{(-COOH)}$
	$D^2_2(5)$	*N1-H1$_{(-NH3+)}$···O5$_{(-COO-)}$ & N4-H18$_{(-NH3+)}$···O6$_{(-COO-)}$<br>*N1-H3$_{(-NH3+)}$···O5$_{(-COO-)}$ & N4-H18$_{(-NH3+)}$···O6$_{(-COO-)}$<br>*O9-H27$_{(COOH)}$ ···O4$_{(-COO-)}$ & N2-H4$_{(-NH3+)}$···O3$_{(-COO-)}$<br>*N2-H6$_{(-NH3+)}$···O11$_{(-COOH)}$ & N3-H14$_{(-NH3+)}$···O10$_{(-COOH)}$<br>*N4-H18$_{(-NH3+)}$···O6$_{(-COO-)}$ & C2-H7$_{(-CH)}$···O5$_{(-COO-)}$<br>*C5-H12$_{(-CH2)}$···O7$_{(-C=O)}$ & C5-H13$_{(-CH2)}$···O1$_{(-COO-)}$<br>*N4-H17$_{(-NH3+)}$···O1$_{(-COO-)}$ & N4-H18$_{(-NH3+)}$···O6$_{(-COO-)}$<br>*N4-H17$_{(-NH3+)}$···O1$_{(-COO-)}$ & N4-H19$_{(-NH3+)}$···O14$_{(-COOH)}$<br>*O13-H32$_{(COOH)}$ ···O2$_{(-COO-)}$ & N4-H17$_{(-NH3+)}$···O1$_{(-COO-)}$<br>*N4-H19$_{(-NH3+)}$···O14$_{(-COOH)}$ & N4-H18$_{(-NH3+)}$···O6$_{(-COO-)}$
	$D^2_2(6)$	*N1-H3$_{(-NH3+)}$···O7$_{(-C=O)}$ & N4-H18$_{(-NH3+)}$···O6$_{(-COO-)}$<br>*N2-H5$_{(-NH3+)}$···O8$_{(-COOH)}$ & C5-H13$_{(-CH2)}$···O11$_{(-COOH)}$<br>*O13-H32$_{(COOH)}$···O2$_{(-COO-)}$ & C2-H7$_{(-CH)}$···O5$_{(-COO-)}$<br>*N4-H17$_{(-NH3+)}$···O1$_{(-COO-)}$ & C10-H26$_{(-CH2)}$···O14$_{(-COOH)}$<br>*N4-H18$_{(-NH3+)}$···O6$_{(-COO-)}$ & C10-H26$_{(-CH2)}$···O14$_{(-COOH)}$<br>*O13-H32$_{(COOH)}$···O2$_{(-COO-)}$ & N4-H19$_{(-NH3+)}$···O14$_{(-COOH)}$<br>*O13-H32$_{(COOH)}$···O2$_{(-COO-)}$ & C10-H26$_{(-CH2)}$···O14$_{(-COOH)}$

	$D^{2}_{2}(7)$	*N1-H1$_{(-NH3+)}$···O5$_{(-COO-)}$ & N4-H17$_{(-NH3+)}$···O1$_{(-COO-)}$<br>*N1-H1$_{(-NH3+)}$···O5$_{(-COO-)}$ & O13-H32$_{(COOH)}$···O2$_{(-COO-)}$<br>*N4-H17$_{(-NH3+)}$···O1$_{(-COO-)}$ & N1-H3$_{(-NH3+)}$···O5$_{(-COO-)}$<br>*N1-H3$_{(-NH3+)}$···O5$_{(-COO-)}$ & O13-H32$_{(COOH)}$···O2$_{(-COO-)}$<br>*O9-H27$_{(-COOH)}$···O4$_{(-COO-)}$ & N3-H14$_{(-NH3+)}$···O10$_{(-COOH)}$<br>*O9-H27$_{(-COOH)}$···O4$_{(-COO-)}$ & N3-H15$_{(-NH3+)}$···O10$_{(-COOH)}$<br>*O9-H27$_{(-COOH)}$···O4$_{(-COO-)}$ & N3-H15$_{(-NH3+)}$···O12$_{(-C=O)}$<br>*O9-H27$_{(-COOH)}$···O4$_{(-COO-)}$ & N3-H16$_{(-NH3+)}$···O10$_{(-COOH)}$<br>*O9-H27$_{(-COOH)}$···O4$_{(-COO-)}$ & C8-H22$_{(-CH2)}$···O12$_{(-C=O)}$
	$D^{2}_{2}(8)$	*N4-H17$_{(-NH3+)}$···O1$_{(-COO-)}$ & C8-H22$_{(-CH2)}$···O12$_{(-C=O)}$<br>*N4-H18$_{(-NH3+)}$···O6$_{(-COO-)}$ & C8-H22$_{(-CH2)}$···O12$_{(-C=O)}$
	$D^{2}_{2}(9)$	*N1-H1$_{(-NH3+)}$···O5$_{(-COO-)}$ & C5-H13$_{(-CH2)}$···O11$_{(-COOH)}$<br>*N1-H3$_{(-NH3+)}$···O5$_{(-COO-)}$ & C5-H13$_{(-CH2)}$···O11$_{(-COOH)}$<br>*O9-H27$_{(-COOH)}$···O4$_{(-COO-)}$ & N1-H3$_{(-NH3+)}$···O7$_{(-C=O)}$<br>*N2-H4$_{(-NH3+)}$···O3$_{(-COO-)}$ & C10-H26$_{(-CH2)}$···O14$_{(-COOH)}$<br>*N2-H6$_{(-NH3+)}$···O11$_{(-COOH)}$ & N4-H19$_{(-NH3+)}$···O14$_{(-COOH)}$<br>*O9-H27$_{(-COOH)}$···O4$_{(-COO-)}$ & C3-H9$_{(-CH2)}$···O7$_{(-C=O)}$<br>*N4-H17$_{(-NH3+)}$···O1$_{(-COO-)}$ & C5-H12$_{(-CH2)}$···O7$_{(-C=O)}$<br>*O13-H32$_{(-COOH)}$···O2$_{(-COO-)}$ & N3-H15$_{(-NH3+)}$···O12$_{(-C=O)}$<br>*O13-H32$_{(-COOH)}$···O2$_{(-COO-)}$ & C8-H22$_{(-CH2)}$···O12$_{(-C=O)}$<br>*O9-H27$_{(-COOH)}$···O4$_{(-COO-)}$ & C10-H26$_{(CH2)}$···O14$_{(-COOH)}$
	$D^{2}_{2}(10)$	*N1-H1$_{(-NH3+)}$···O5$_{(-COO-)}$ & N2-H4$_{(-NH3+)}$···O3$_{(-COO-)}$<br>*O9-H27$_{(-COOH)}$···O4$_{(-COO-)}$ & N1-H1$_{(-NH3+)}$···O5$_{(-COO-)}$<br>*N1-H3$_{(-NH3+)}$···O5$_{(-COO-)}$ & N2-H4$_{(-NH3+)}$···O3$_{(-COO-)}$<br>*O9-H27$_{(-COOH)}$···O4$_{(-COO-)}$ & N1-H3$_{(-NH3+)}$···O5$_{(-COO-)}$<br>*O13-H32$_{(-COOH)}$···O2$_{(COO-)}$ & N2-H4$_{(-NH3+)}$···O3$_{(-COO-)}$<br>*N2-H5$_{(-NH3+)}$···O8$_{(-COOH)}$ & N4-H17$_{(-NH3+)}$···O1$_{(-COO-)}$<br>*N3-H14$_{(-NH3+)}$···O10$_{(-COOH)}$ & N4-H17$_{(-NH3+)}$···O1$_{(-COO-)}$<br>*O13-H32$_{(-COOH)}$···O2$_{(-COO-)}$ & N3-H14$_{(-NH3+)}$···O10$_{(-COOH)}$<br>*N3-H15$_{(-NH3+)}$···O10$_{(-COOH)}$ & N4-H18$_{(-NH3+)}$···O6$_{(-COO-)}$<br>*O13-H32$_{(-COOH)}$···O2$_{(-COO-)}$ & N3-H15$_{(-NH3+)}$···O10$_{(-COOH)}$<br>*N3-H15$_{(-NH3+)}$···O12$_{(-C=O)}$ & N4-H17$_{(-NH3+)}$···O1$_{(-COO-)}$<br>*N3-H16$_{(-NH3+)}$···O10$_{(-COOH)}$ & N4-H17$_{(-NH3+)}$···O1$_{(-COO-)}$<br>*N3-H16$_{(-NH3+)}$···O10$_{(-COOH)}$ & N4-H18$_{(-NH3+)}$···O6$_{(-COO-)}$<br>*O13-H32$_{(-COOH)}$···O2$_{(-COO-)}$ & N3-H16$_{(-NH3+)}$···O10$_{(-COOH)}$<br>*O9-H27$_{(-COOH)}$···O4$_{(-COO-)}$ & N4-H17$_{(-NH3+)}$···O1$_{(-COO-)}$<br>*O9-H27$_{(-COOH)}$···O4$_{(-COO-)}$ & N4-H19$_{(-NH3+)}$···O14$_{(-COOH)}$
	$D^{2}_{3}(8)$	N1-H2$_{(-NH3+)}$···O1$_{(-COO-)}$ & (N4-H17$_{(-NH3+)}$···O1$_{(-COO-)}$)$_{2}$
	$D^{2}_{3}(9)$	*(N4-H17$_{(-NH3+)}$···O1$_{(-COO-)}$)$_{2}$ & C4-H10$_{(-CH2)}$···O1$_{(-COO-)}$<br>*(C10-H26$_{(CH2)}$···O14$_{(-COOH)}$)$_{2}$ & C19-H35$_{(-CH2)}$···O13$_{(-COOH)}$<br>*(O13-H32$_{(-COOH)}$···O2$_{(-COO-)}$)$_{2}$ & C19-H35$_{(-CH2)}$···O13$_{(-COOH)}$
	$D^{3}_{3}(9)$	*(N4-H18$_{(-NH3+)}$···O14$_{(-COOH)}$)$_{2}$ & C19-H35$_{(-CH2)}$···O13$_{(-COOH)}$<br>*(O9-H27$_{(-COOH)}$···O4$_{(-COO-)}$)$_{2}$ & C7-H20$_{(-CH)}$···O3$_{(-COO-)}$
	$D^{3}_{3}(10)$	*(N1-H1$_{(-NH3+)}$···O5$_{(-COO-)}$)$_{2}$ & N1-H2$_{(-NH3+)}$···O1$_{(-COO-)}$<br>*N1-H2$_{(-NH3+)}$···O1$_{(-COO-)}$ & (C2-H7$_{(-CH)}$···O5$_{(-COO-)}$)$_{2}$<br>*N1-H2$_{(-NH3+)}$···O1$_{(-COO-)}$ & (O13-H32$_{(-COOH)}$···O2$_{(-COO-)}$)$_{2}$
	$D^{3}_{3}(11)$	*C2-H7$_{(-CH)}$···O5$_{(-COO-)}$ & C4-H10$_{(-CH2)}$···O1$_{(-COO-)}$ & C2-H8$_{(-CH)}$···O5$_{(-COO-)}$<br>*(O13-H32$_{(-COOH)}$···O2$_{(-COO-)}$)$_{2}$ & C4-H10$_{(-CH2)}$···O1$_{(-COO-)}$<br>*(N3-H15$_{(-NH3+)}$···O10$_{(-COOH)}$)$_{2}$ & C7-H20$_{(-CH)}$···O3$_{(-COO-)}$<br>*(N3-H15$_{(-NH3+)}$···O12$_{(C=O)}$)$_{2}$ & C7-H20$_{(-CH)}$···O3$_{(-COO-)}$<br>*(N3-H16$_{(-NH3+)}$···O10$_{(-COOH)}$)$_{2}$ & C7-H20$_{(-CH)}$···O3$_{(-COO-)}$<br>*C7-H20$_{(-CH)}$···O3$_{(-COO-)}$ & C8-H22$_{(-CH2)}$···O12$_{(C=O)}$ & C8-H22$_{(-CH2)}$···O12$_{(-C=O)}$
	$D^{3}_{3}(12)$	N1-H2$_{(-NH3+)}$···O1$_{(-COO-)}$ & (C3-H9$_{(-CH2)}$···O7$_{(-C=O)}$)$_{2}$
	$D^{3}_{3}(13)$	*(N1-H1$_{(-NH3+)}$···O5$_{(-COO-)}$)$_{2}$ & C4-H10$_{(-CH2)}$···O1$_{(-COO-)}$<br>*(N1-H3$_{(-NH3+)}$···O5$_{(-COO-)}$)$_{2}$ & C4-H10$_{(-CH2)}$···O1$_{(-COO-)}$<br>*C4-H10$_{(-CH2)}$···O1$_{(-COO-)}$ & (C5-H12$_{(-CH2)}$···O7$_{(-C=O)}$)$_{2}$<br>*(N3-H15$_{(-NH3+)}$···O12$_{(-C=O)}$)$_{2}$ & C19-H35$_{(-CH2)}$···O13$_{(-COOH)}$<br>*(C8-H22$_{(-CH2)}$···O12$_{(-C=O)}$)$_{2}$ & C19-H35$_{(-CH2)}$···O13$_{(-COOH)}$
	$D^{3}_{3}(15)$	*(N2-H5$_{(-NH3+)}$···O8$_{(-COOH)}$)$_{2}$ & C4-H10$_{(-CH2)}$···O1$_{(-COO-)}$<br>*C5-H13$_{(-CH2)}$···O11$_{(-COOH)}$ & C19-H35$_{(-CH2)}$···O13$_{(-COOH)}$<br>*N3-H14$_{(-NH3+)}$···O10$_{(-COOH)}$ & N3-H14$_{(-NH3+)}$···O10$_{(-COOH)}$<br>*(N3-H15$_{(-NH3+)}$···O10$_{(-COOH)}$)$_{2}$ & C19-H35$_{(-CH2)}$···O13$_{(-COOH)}$<br>*(N3-H16$_{(-NH3+)}$···O10$_{(-COOH)}$)$_{2}$ & C19-H35$_{(-CH2)}$···O13$_{(-COOH)}$<br>*C7-H20$_{(-CH)}$···O3$_{(-COO-)}$ & (C10-H26$_{(-CH2)}$···O14$_{(-COOH)}$)$_{2}$
	$D^{3}_{3}(16)$	*N1-H2$_{(-NH3+)}$···O1$_{(-COO-)}$ & (C5-H12$_{(-CH2)}$···O7$_{(-C=O)}$)$_{2}$<br>*N1-H2$_{(-NH3+)}$···O1$_{(-COO-)}$ & (C5-H12$_{(-CH2)}$···O9$_{(-COOH)}$)$_{2}$

	$D^3_3(17)$	*(N4-H17$_{(-NH3+)}$···O1$_{(-COO-)}$)$_2$ & C7-H20$_{(-CH)}$···O3$_{(-COO-)}$<br>*(N4-H13$_{(-NH3+)}$···O6$_{(-COO-)}$)$_2$ & C7-H20$_{(-CH)}$···O3$_{(-COO-)}$<br>*(N4-H19$_{(-NH3+)}$···O14)$_2$ & C7-H20$_{(-CH)}$···O3$_{(-COO-)}$
	$D^3_3(18)$	N1-H2$_{(-NH3+)}$···O1$_{(-COO-)}$ & (N2-H4$_{(-NH3+)}$···O3$_{(-COO-)}$)$_2$
**PUYVUA**	$S(6)$	C4-H10$_{(CH2)}$···O2$_{(COO-)}$**ornithine**
	$C(5)$	N1-H2$_{(NH3+)}$···O2$_{(COO-)}$ **ornithine**
	$C(6)$	C4-H10$_{(CH2)}$···O1$_{(COO-)}$ **ornithine**
	$C(8)$	N2-H9$_{(NH3+)}$···O1$_{(COO-)}$ **ornithine**
	$D(2)$	*N1-H3$_{(NH3+)}$···O3$_{(NO3-)}$<br>*N2-H7$_{(NH3+)}$···O4$_{(NO3-)}$<br>*C5-H12$_{(CH2)}$···O5$_{(NO3-)}$
level 2	$C^1_2(4)$	N1-H4$_{(NH3+)}$···O2$_{(COO-)}$ **ornithine** & N1-H2$_{(NH3+)}$···O2$_{(COO-)}$ **ornithine**
	$C^1_2(5)$	N2-H8$_{(NH3+)}$···O5$_{(NO3-)}$ & C5-H12$_{(CH2)}$···O5$_{(NO3-)}$
	$C^2_2(6)$	N2-H7$_{(NH3+)}$···O4$_{(NO3-)}$ & N2-H8$_{(NH3+)}$···O5$_{(NO3-)}$
	$C^2_2(7)$	N2-H7$_{(NH3+)}$···O4$_{(NO3-)}$ & C5-H12$_{(CH2)}$···O5$_{(NO3-)}$
	$C^2_2(9)$	N1-H2$_{(NH3+)}$···O2$_{(COO-)}$ **ornithine** & C4-H10$_{(CH2)}$···O1$_{(COO-)}$ **ornithine**
	$C^2_2(10)$	*N1-H4$_{(NH3+)}$···O2$_{(COO-)}$ **ornithine** & N1-H2$_{(NH3+)}$···O2$_{(COO-)}$ **ornithine**<br>*N1-H3$_{(NH3+)}$···O3$_{(NO3-)}$ & C5-H12$_{(CH2)}$···O5$_{(NO3-)}$
	$C^2_2(11)$	*N1-H2$_{(NH3+)}$···O2$_{(COO-)}$ **ornithine** & N2-H9$_{(NH3+)}$···O1$_{(COO-)}$ **ornithine**<br>*N1-H3$_{(NH3+)}$···O3$_{(NO3-)}$ & N2-H7$_{(NH3+)}$···O4$_{(NO3-)}$<br>*N1-H4$_{(NH3+)}$···O2$_{(COO-)}$ **ornithine** & C4-H10$_{(CH2)}$···O1$_{(COO-)}$ **ornithine**
	$C^2_2(13)$	N1-H4$_{(NH3+)}$···O2$_{(COO-)}$ **ornithine** & N2-H9$_{(NH3+)}$···O1$_{(COO-)}$ **ornithine**
	$C^2_2(14)$	N2-H9$_{(NH3+)}$···O1$_{(COO-)}$ **ornithine** & C4-H10$_{(CH2)}$···O1$_{(COO-)}$ **ornithine**
	$C^3_4(14)$	(N1-H4$_{(NH3+)}$···O2$_{(COO-)}$)$_2$ **ornithine** & (N1-H2$_{(NH3+)}$···O2$_{(COO-)}$)$_2$ **ornithine**
	$C^4_4(20)$	(N1-H4$_{(NH3+)}$···O2$_{(COO-)}$)$_2$ **ornithine** & (C4-H10$_{(CH2)}$···O1$_{(COO-)}$)$_2$ **ornithine**
	$R^1_2(6)$	N2-H9$_{(NH3+)}$···O1$_{(COO-)}$ **ornithine** & C4-H10$_{(CH2)}$···O1$_{(COO-)}$ **ornithine**
	$R^2_2(11)$	N1-H2$_{(NH3+)}$···O2$_{(COO-)}$ **ornithine** & C4-H10$_{(CH2)}$···O1$_{(COO-)}$ **ornithine**
	$R^2_2(13)$	N1-H2$_{(NH3+)}$···O2$_{(COO-)}$ **ornithine** & N2-H9$_{(NH3+)}$···O1$_{(COO-)}$ **ornithine**
	$R^3_4(14)$	(N1-H4$_{(NH3+)}$···O2$_{(COO-)}$)$_2$ **ornithine** & (N1-H2$_{(NH3+)}$···O2$_{(COO-)}$)$_2$ **ornithine**
	$R^4_4(20)$	(N1-H4$_{(NH3+)}$···O2$_{(COO-)}$)$_2$ **ornithine** & (C4-H10$_{(CH2)}$···O1$_{(COO-)}$)$_2$ **ornithine**
	$D^3_3(10)$	(N1-H3$_{(NH3+)}$···O3$_{(NO3-)}$)$_2$ & N1-H2···O2$_{(COO-)}$ **ornithine**
	$D^3_3(13)$	*(N2-H8$_{(NH3+)}$···O5$_{(NO3-)}$)$_2$ & N2-H9$_{(NH3+)}$···O1$_{(COO-)}$ **ornithine**<br>*(N1-H3$_{(NH3+)}$···O3$_{(NO3-)}$)$_2$ & C4-H10$_{(CH2)}$···O1$_{(COO-)}$ **ornithine**<br>*C4-H10$_{(CH2)}$···O1$_{(COO-)}$ **ornithine** & (C5-H12$_{(CH2)}$···O5$_{(NO3-)}$)$_2$ **ornithine**
	$D^3_3(15)$	*(N1-H3$_{(NH3+)}$···O3$_{(NO3-)}$)$_2$ & N2-H9$_{(NH3+)}$···O1$_{(COO-)}$ **ornithine**<br>*(N2-H7$_{(NH3+)}$···O4$_{(NO3-)}$)$_2$ & C4-H10$_{(CH2)}$···O1$_{(COO-)}$ **ornithine**
	$D^3_3(16)$	*(N2-H7$_{(NH3+)}$···O4$_{(NO3-)}$)$_2$ & N2-H9$_{(NH3+)}$···O1$_{(COO-)}$ **ornithine**<br>*N1-H2$_{(NH3+)}$···O2$_{(COO-)}$ **ornithine** & (C5-H12$_{(CH2)}$···O5$_{(NO3-)}$)$_2$
	$D^3_3(18)$	N1-H2$_{(NH3+)}$···O2$_{(COO-)}$ **ornithine** & (N2-H7$_{(NH3+)}$···O4$_{(NO3-)}$)$_2$
	$D^3_3(16)$	N1-H2$_{(NH3+)}$ ···O2$_{(COO-)}$ **ornithine** & (C5-H12$_{(CH2)}$···O5$_{(NO3-)}$)$_2$
**TEHMIA**	$D(2)$	*O16$_{(COOH)}$-H3···O13$_{(NO2)}$<br>*C14-H6$_{(CH)}$···O13$_{(NO2)}$<br>*C14-H18$_{(CH)}$···O8$_{(C=Oarom.)}$<br>*N7-H7$_{(NH3+)}$···O1$_{(C=Oarom.)}$<br>*N7-H8$_{(NH3+)}$···O14$_{(NO2)}$<br>*C15-H9$_{(CH2)}$···O11$_{(NO2)}$<br>*C15-H10$_{(CH2)}$···O4$_{(NO2)}$<br>*C16-H11$_{(CH2)}$···O4$_{(NO2)}$<br>*C14-H12$_{(CH)}$···O11$_{(NO2)}$<br>*C17-H13$_{(CH2)}$···O12$_{(NO2)}$<br>*C17-H14$_{(CH2)}$···O4$_{(NO2)}$<br>*N8-H15$_{(NH3+)}$···O1$_{(C=Oarom.)}$<br>*N8-H16$_{(NH3+)}$···O9$_{(NO2)}$<br>*N8-H16$_{(NH3+)}$···O10$_{(NO2)}$<br>*N8-H17$_{(NH3+)}$···O8$_{(C=Oarom.)}$<br>*N8-H17$_{(NH3+)}$···O14$_{(NO2)}$
level 2	$C^1_2(8)$	N7-H18$_{(NH3+)}$···O8$_{(C=Oarom.)}$ & N8-H17$_{(NH3+)}$···O8$_{(C=Oarom.)}$
	$C^1_2(9)$	*N7-H7$_{(NH3+)}$···O1$_{(C=Oarom.)}$ & N8-H15$_{(NH3+)}$···O1$_{(C=Oarom.)}$<br>*N7-H8$_{(NH3+)}$···O14$_{(NO2)}$ & N8-H17$_{(NH3+)}$···O14$_{(NO2)}$
	$C^2_2(7)$	O16-H5$_{(COOH)}$···O13$_{(NO2)}$ & N7-H7$_{(NH3+)}$···O14$_{(NO2)}$
	$C^2_2(8)$	*O16-H5$_{(COOH)}$···O13$_{(NO2)}$ & N8-H17$_{(NH3+)}$···O8$_{(C=Oarom.)}$<br>*N7-H18$_{(NH3+)}$···O8$_{(C=Oarom.)}$ & N7-H8$_{(NH3+)}$···O14$_{(NO2)}$<br>*N8-H16$_{(NH3+)}$···O9$_{(NO2)}$ & N8-H17$_{(NH3+)}$···O8$_{(C=Oarom.)}$<br>*N8-H16$_{(NH3+)}$···O10$_{(NO2)}$ & N8-H17$_{(NH3+)}$···O8$_{(C=Oarom.)}$
	$C^2_2(9)$	O16-H5$_{(COOH)}$···O13$_{(NO2)}$ & N7-H7$_{(NH3+)}$···O14$_{(NO2)}$

	$C^2_2(10)$	*O16-H5$_{(COOH)}$···O13$_{(NO2)}$ & N7-H18$_{(NH3+)}$···O8$_{(C=Oarom.)}$<br>*N8-H17$_{(NH3+)}$···O14$_{(NO2)}$ & O16-H5$_{(COOH)}$···O13$_{(NO2)}$<br>*N8-H16$_{(NH3+)}$···O9$_{(NO2)}$ & N8-H17$_{(NH3+)}$···O14$_{(NO2)}$<br>*N8-H16$_{(NH3+)}$···O10$_{(NO2)}$ & N8-H17$_{(NH3+)}$···O14$_{(NO2)}$
	$C^2_2(11)$	*O16-H5$_{(COOH)}$···O13$_{(NO2)}$ & C15-H9$_{(CH2)}$···C11$_{(CHarom.)}$<br>*N7-H18$_{(NH3+)}$···O8$_{(C=Oarom.)}$ & C15-H9$_{(CH2)}$···O11$_{(NO2)}$<br>*N8-H16$_{(NH3+)}$···O9$_{(NO2)}$ & C17-H13$_{(CH2)}$···O12$_{(NO2)}$<br>*N8-H16$_{(NH3+)}$···O10$_{(NO2)}$ & C17-H13$_{(CH2)}$···O12$_{(NO2)}$<br>*N8-H17$_{(NH3+)}$···O8$_{(C=Oarom.)}$ & C17-H13$_{(CH2)}$···O12$_{(NO2)}$<br>*N8-H17$_{(NH3+)}$···O14$_{(NO2)}$ & C17-H13$_{(CH2)}$···O12$_{(NO2)}$<br>*N8-H15$_{(NH3+)}$···O1$_{(C=Oarom.)}$ & C17-H14$_{(CH2)}$···O4$_{(NO2)}$
	$C^2_2(12)$	*O16-H5$_{(COOH)}$···O13$_{(NO2)}$ & N8-H17$_{(NH3+)}$···O8$_{(C=Oarom.)}$<br>*O16-H5$_{(COOH)}$···O13$_{(NO2)}$ & C16-H12$_{(CH2)}$···O11$_{(NO2)}$<br>*N8-H17$_{(NH3+)}$···O8$_{(C=Oarom.)}$ & C14-H6$_{(CH)}$···O13$_{(NO2)}$<br>*N7-H18$_{(NH3+)}$···O8$_{(C=Oarom.)}$ & C16-H12$_{(CH2)}$···O11$_{(NO2)}$<br>*N7-H18$_{(NH3+)}$···O8$_{(C=Oarom.)}$ & N8-H16$_{(NH3+)}$···O9$_{(NO2)}$<br>*N7-H18$_{(NH3+)}$···O8$_{(C=Oarom.)}$ & N8-H16$_{(NH3+)}$···O10$_{(NO2)}$<br>*N8-H17$_{(NH3+)}$···O8$_{(C=Oarom.)}$ & N7-H18$_{(NH3+)}$···O8$_{(C=Oarom.)}$<br>*N7-H7$_{(NH3+)}$···O1$_{(C=Oarom.)}$ & C15-H10$_{(CH2)}$···O4$_{(NO2)}$<br>*N7-H7$_{(NH3+)}$···O2$_{(NO2)}$ & C15-H10$_{(CH2)}$···O4$_{(NO2)}$<br>*N7-H8$_{(NH3+)}$···O14$_{(NO2)}$ & C15-H9$_{(CH2)}$···O11$_{(NO2)}$<br>*N8-H15$_{(NH3+)}$···O1$_{(C=Oarom.)}$) & C16-H11$_{(CH2)}$···O4$_{(NO2)}$<br>*N8-H16$_{(NH3+)}$···O9$_{(NO2)}$ & C16-H12$_{(CH2)}$···O11$_{(NO2)}$<br>*N8-H16$_{(NH3+)}$···O10$_{(NO2)}$ & C16-H12$_{(CH2)}$···O11$_{(NO2)}$<br>*N8-H17$_{(NH3+)}$···O8$_{(C=Oarom.)}$ & C16-H12$_{(CH2)}$···O11$_{(NO2)}$<br>*N8-H17$_{(NH3+)}$···O14$_{(NO2)}$ & C16-H12$_{(CH2)}$···O11$_{(NO2)}$
	$C^2_2(13)$	*O16-H5$_{(COOH)}$···O13$_{(NO2)}$ & C15-H9$_{(CH2)}$···O11$_{(NO2)}$<br>*O16-H5$_{(COOH)}$···O13$_{(NO2)}$ & C17-H13$_{(CH2)}$···O12$_{(NO2)}$<br>*N7-H18$_{(NH3+)}$···O8$_{(C=Oarom.)}$ & C17-H13$_{(CH2)}$···O12$_{(NO2)}$<br>*N7-H7$_{(NH3+)}$···O1$_{(C=Oarom.)}$ & C16-H11$_{(CH2)}$···O4$_{(NO2)}$<br>*N7-H7$_{(NH3+)}$···O2$_{(NO2)}$ & C16-H11$_{(CH2)}$···O4$_{(NO2)}$<br>*N7-H7$_{(NH3+)}$···O2$_{(NO2)}$ & N8-H15$_{(NH3+)}$···O1$_{(C=Oarom.)}$<br>*N7-H8$_{(NH3+)}$···O14$_{(NO2)}$ & C16-H12$_{(CH2)}$···O11$_{(NO2)}$<br>*N7-H8$_{(NH3+)}$···O14$_{(NO2)}$ & N8-H17$_{(NH3+)}$···O8$_{(C=Oarom.)}$<br>*N8-H16$_{(NH3+)}$···O9$_{(NO2)}$ & C15-H9$_{(CH2)}$···O11$_{(NO2)}$<br>*N8-H16$_{(NH3+)}$···O10$_{(NO2)}$ & C15-H9$_{(CH2)}$···O11$_{(NO2)}$<br>*N8-H17$_{(NH3+)}$···O8$_{(C=Oarom.)}$ & C15-H9$_{(CH2)}$···O11$_{(NO2)}$<br>*N8-H17$_{(NH3+)}$···O14$_{(NO2)}$ & C15-H9$_{(CH2)}$···O11$_{(NO2)}$<br>*N8-H15$_{(NH3+)}$···O1$_{(C=Oarom.)}$ & C15-H10$_{(CH2)}$···O4$_{(NO2)}$
	$C^2_2(14)$	*O16-H5$_{(COOH)}$···O13$_{(NO2)}$ & C16-H12$_{(CH2)}$···O11$_{(NO2)}$<br>*O16-H5$_{(COOH)}$···O13$_{(NO2)}$ & N8-H17$_{(NH3+)}$···O8$_{(C=Oarom.)}$<br>*O16-H5$_{(COOH)}$···O13$_{(NO2)}$ & N8-H16$_{(NH3+)}$···O9$_{(NO2)}$<br>*O16-H5$_{(COOH)}$···O13$_{(NO2)}$ & N8-H16$_{(NH3+)}$···O10$_{(NO2)}$<br>*N7-H7$_{(NH3+)}$···O1$_{(C=Oarom.)}$ & C17-H14$_{(CH2)}$···O4$_{(NO2)}$<br>*N7-H7$_{(NH3+)}$···O2$_{(NO2)}$ & C17-H14$_{(CH2)}$···O4$_{(NO2)}$<br>*N7-H8$_{(NH3+)}$···O14$_{(NO2)}$ & C17-H13$_{(CH2)}$···O12$_{(NO2)}$
	$C^2_2(15)$	*O16-H5$_{(COOH)}$···O13$_{(NO2)}$ & C17-H13$_{(CH2)}$···O12$_{(NO2)}$<br>*N7-H8$_{(NH3+)}$···O14$_{(NO2)}$ & N8-H16$_{(NH3+)}$···O9$_{(NO2)}$<br>*N7-H8$_{(NH3+)}$···O14$_{(NO2)}$ & N8-H16$_{(NH3+)}$···O10$_{(NO2)}$
	$C^2_2(16)$	*O16-H5$_{(COOH)}$···O13$_{(NO2)}$ & N8-H16$_{(NH3+)}$···O9$_{(NO2)}$<br>*O16-H5$_{(COOH)}$···O13$_{(NO2)}$ & N8-H16$_{(NH3+)}$···O10$_{(NO2)}$
	$R^1_2(5)$	*C15-H9$_{(CH2)}$···O11$_{(NO2)}$ & C16-H12$_{(CH2)}$···O11$_{(NO2)}$<br>*C15-H10$_{(CH2)}$···O4$_{(NO2)}$ & C16-H11$_{(CH2)}$···O4$_{(NO2)}$<br>*C16-H11$_{(CH2)}$···O4$_{(NO2)}$ & C17-H14$_{(CH2)}$···O4$_{(NO2)}$
	$R^1_2(6)$	*O16-H5$_{(COOH)}$···O13$_{(NO2)}$ & C14-H6$_{(CH)}$···O13$_{(NO2)}$<br>*C15-H10$_{(CH2)}$···O4$_{(NO2)}$ & C17-H14$_{(CH2)}$···O4$_{(NO2)}$
	$R^2_1(4)$	N8-H16$_{(NH3+)}$···O10$_{(NO2)}$ & N8-H16$_{(NH3+)}$···O9$_{(NO2)}$
	$R^2_1(6)$	*N7-H7$_{(NH3+)}$···O1$_{(C=Oarom.)}$) & N7-H7$_{(NH3+)}$···O2$_{(NO2)}$<br>*N8-H17$_{(NH3+)}$···O8$_{(C=Oarom.)}$ & N8-H17$_{(NH3+)}$···O14$_{(NO2)}$
	$R^2_2(7)$	C16-H12$_{(CH2)}$···O11$_{(NO2)}$ & C17-H13$_{(CH2)}$···O12$_{(NO2)}$
	$R^2_2(8)$	C15-H9$_{(CH2)}$···O11$_{(NO2)}$ & C17-H13$_{(CH2)}$···O12$_{(NO2)}$
	$D^2_2(5)$	*N7-H7$_{(NH3+)}$···O2$_{(NO2)}$ & C11-H4$_{(CHarom.)}$···O3$_{(NO2)}$<br>*N7-H7$_{(NH3+)}$···O1$_{(C=Oarom.)}$ & N7-H18$_{(NH3+)}$···O8$_{(C=Oarom.)}$<br>*N7-H7$_{(NH3+)}$···O2$_{(NO2)}$ & N7-H18$_{(NH3+)}$···O8$_{(C=Oarom.)}$<br>*N7-H7$_{(NH3+)}$···O1$_{(C=Oarom.)}$ & N7-H8$_{(NH3+)}$···O14$_{(NO2)}$<br>*N7-H7$_{(NH3+)}$···O2$_{(NO2)}$ & N8-H17$_{(NH3+)}$···O14$_{(NO2)}$

		*C15-H10$_{(CH2)}$···O4$_{(NO2)}$ & C15-H9$_{(CH2)}$···O11$_{(NO2)}$<br>*C16-H11$_{(CH2)}$···O4$_{(NO2)}$ & C16-H12$_{(CH2)}$···O11$_{(NO2)}$<br>*C17-H13$_{(CH2)}$···O12$_{(NO2)}$ & C17-H14$_{(CH2)}$···O4$_{(NO2)}$<br>*N8-H15$_{(NH3+)}$···O1$_{(C=Oarom.)}$ & N8-H16$_{(NH3+)}$···O9$_{(NO2)}$<br>*N8-H15$_{(NH3+)}$···O1$_{(C=Oarom.)}$ & N8-H16$_{(NH3+)}$···O10$_{(NO2)}$<br>*N8-H15$_{(NH3+)}$···O1$_{(C=Oarom.)}$ & N8-H17$_{(NH3+)}$···O8$_{(C=Oarom.)}$<br>*N8-H15$_{(NH3+)}$···O1$_{(C=Oarom.)}$ & N8-H17$_{(NH3+)}$···O14$_{(NO2)}$
	$D^2_2(6)$	*O16-H5$_{(COOH)}$···O13$_{(NO2)}$ & N7-H7$_{(NH3+)}$···O1$_{(C=Oarom.)}$<br>*O16-H5$_{(COOH)}$···O13$_{(NO2)}$ & N7-H7$_{(NH3+)}$···O2$_{(NO2)}$<br>*O16-H5$_{(COOH)}$···O13$_{(NO2)}$ & C15-H10$_{(CH2)}$···O4$_{(NO2)}$<br>*N7-H18$_{(NH3+)}$···O2$_{(NO2)}$ & C15-H10$_{(CH2)}$···O4$_{(NO2)}$<br>*C15-H9$_{(CH2)}$···O11$_{(NO2)}$ & C16-H11$_{(CH2)}$···O4$_{(NO2)}$<br>*C15-H10$_{(CH2)}$···O4$_{(NO2)}$ & C16-H12$_{(CH2)}$···O11$_{(NO2)}$<br>*C16-H11$_{(CH2)}$···O4$_{(NO2)}$ & C17-H13$_{(CH2)}$···O12$_{(NO2)}$<br>*C16-H12$_{(CH2)}$···O11$_{(NO2)}$ & C17-H14$_{(CH2)}$···O4$_{(NO2)}$<br>*N8-H15$_{(NH3+)}$···O1$_{(C=Oarom.)}$ & C17-H13$_{(CH2)}$···O12$_{(NO2)}$<br>*N8-H16$_{(NH3+)}$···O9$_{(NO2)}$ & C17-H14$_{(CH2)}$···O4$_{(NO2)}$<br>*N8-H16$_{(NH3+)}$···O10$_{(NO2)}$ & C17-H14$_{(CH2)}$···O4$_{(NO2)}$<br>*N8-H17$_{(NH3+)}$···O8$_{(C=Oarom.)}$ & C17-H14$_{(CH2)}$···O4$_{(NO2)}$<br>*N8-H17$_{(NH3+)}$···O14$_{(NO2)}$ & C17-H14$_{(CH2)}$···O4$_{(NO2)}$
	$D^2_2(7)$	*O16-H5$_{(COOH)}$···O13$_{(NO2)}$ & C11-H14$_{(CHarom.)}$···O3$_{(NO2)}$<br>*C11-H4$_{(CHarom.)}$···O3$_{(NO2)}$ & C14-H6$_{(CH)}$···O13$_{(NO2)}$<br>*N7-H18$_{(NH3+)}$···O8$_{(C=Oarom.)}$ & C11-H4$_{(CHarom.)}$···O3$_{(NO2)}$<br>*N7-H7$_{(NH3+)}$···O1$_{(C=Oarom.)}$) & C11-H4$_{(CHarom.)}$···O3$_{(NO2)}$<br>*N7-H8$_{(NH3+)}$···O14$_{(NO2)}$ & C11-H4$_{(CHarom.)}$···O3$_{(NO2)}$<br>*C11-H4$_{(CHarom.)}$···O3$_{(NO2)}$ & C15-H9$_{(CH2)}$···O11$_{(NO2)}$<br>*C11-H4$_{(CHarom.)}$···O3$_{(NO2)}$ & C16-H12$_{(CH2)}$···O11$_{(NO2)}$<br>*C11-H4$_{(CHarom.)}$···O3$_{(NO2)}$ & C17-H13$_{(CH2)}$···O12$_{(NO2)}$<br>*N8-H15$_{(NH3+)}$···O1$_{(C=Oarom.)}$ & C11-H14$_{(CHarom.)}$···O3$_{(NO2)}$<br>*N8-H17$_{(NH3+)}$···O8$_{(C=Oarom.)}$ & C11-H14$_{(CHarom.)}$···O3$_{(NO2)}$<br>*N8-H17$_{(NH3+)}$···O14$_{(NO2)}$ & C11-H14$_{(CHarom.)}$···O3$_{(NO2)}$<br>*O16-H5$_{(COOH)}$···O13$_{(NO2)}$ & C16-H11$_{(CH2)}$···O4$_{(NO2)}$<br>*N7-H18$_{(NH3+)}$···O8$_{(C=Oarom.)}$ & C16-H11$_{(CH2)}$···O4$_{(NO2)}$<br>*N7-H7$_{(NH3+)}$···O1$_{(C=Oarom.)}$ & C15-H9$_{(CH2)}$···O11$_{(NO2)}$<br>*N7-H7$_{(NH3+)}$···O2$_{(NO2)}$ & C15-H9$_{(CH2)}$···O11$_{(NO2)}$<br>*N7-H8$_{(NH3+)}$···O14$_{(NO2)}$ & C15-H10$_{(CH2)}$···O4$_{(NO2)}$<br>*C15-H9$_{(CH2)}$···O11$_{(NO2)}$ & C17-H14$_{(CH2)}$···O4$_{(NO2)}$<br>*C15-H10$_{(CH2)}$···O4$_{(NO2)}$ & C17-H13$_{(CH2)}$···O12$_{(NO2)}$<br>*N8-H16$_{(NH3+)}$···O9$_{(NO2)}$ & C16-H11$_{(CH2)}$···O4$_{(NO2)}$<br>*N8-H16$_{(NH3+)}$···O10$_{(NO2)}$ & C16-H11$_{(CH2)}$···O4$_{(NO2)}$<br>*N8-H17$_{(NH3+)}$···O8$_{(C=Oarom.)}$ & C16-H11$_{(CH2)}$···O4$_{(NO2)}$<br>*N8-H17$_{(NH3+)}$···O14$_{(NO2)}$ & C16-H11$_{(CH2)}$···O4$_{(NO2)}$<br>*N8-H15$_{(NH3+)}$···O1$_{(C=Oarom.)}$ & C16-H12$_{(CH2)}$···O11$_{(NO2)}$
	$D^2_2(8)$	*N7-H7$_{(NH3+)}$···O1$_{(C=Oarom.)}$ & O16-H5$_{(COOH)}$···O13$_{(NO2)}$<br>*N7-H7$_{(NH3+)}$···O2$_{(NO2)}$ & O16-H5$_{(COOH)}$···O13$_{(NO2)}$<br>*O16-H5$_{(COOH)}$···O13$_{(NO2)}$ & C15-H10$_{(CH2)}$···O4$_{(NO2)}$<br>*O16-H5$_{(COOH)}$···O13$_{(NO2)}$ & C17-H14$_{(CH2)}$···O4$_{(NO2)}$<br>*N7-H18$_{(NH3+)}$···O8$_{(C=Oarom.)}$ & C17-H14$_{(CH2)}$···O4$_{(NO2)}$<br>*N7-H7$_{(NH3+)}$···O1$_{(C=Oarom.)}$ & C16-H12$_{(CH2)}$···O11$_{(NO2)}$<br>*N7-H7$_{(NH3+)}$···O1$_{(C=Oarom.)}$ & C16-H12$_{(CH2)}$···O11$_{(NO2)}$<br>*N7-H8$_{(NH3+)}$···O14$_{(NO2)}$ & C16-H11$_{(CH2)}$···O4$_{(NO2)}$<br>*N8-H15$_{(NH3+)}$···O1$_{(C=Oarom.)}$) & C15-H9$_{(CH2)}$···O11$_{(NO2)}$<br>*N8-H16$_{(NH3+)}$···O9$_{(NO2)}$ & C15-H10$_{(CH2)}$···O4$_{(NO2)}$<br>*N8-H16$_{(NH3+)}$···O10$_{(NO2)}$ & C15-H10$_{(CH2)}$···O4$_{(NO2)}$<br>*N8-H17$_{(NH3+)}$···O8$_{(C=Oarom.)}$ & C15-H10$_{(CH2)}$···O4$_{(NO2)}$<br>*N8-H17$_{(NH3+)}$···O14$_{(NO2)}$ & C15-H10$_{(CH2)}$···O4$_{(NO2)}$
	$D^2_2(9)$	*C11-H4$_{(CHarom.)}$···O3$_{(NO2)}$ & C15-H10$_{(CH2)}$···O4$_{(NO2)}$<br>*C11-H4$_{(CHarom.)}$···O3$_{(NO2)}$ & C16-H11$_{(CH2)}$···O4$_{(NO2)}$<br>*C11-H4$_{(CHarom.)}$···O3$_{(NO2)}$ & C17-H14$_{(CH2)}$···O4$_{(NO2)}$<br>*N8-H16$_{(NH3+)}$ ···O9$_{(NO2)}$ & C11-H14$_{(CHarom.)}$···O3$_{(NO2)}$<br>*N8-H16$_{(NH3+)}$···O10$_{(NO2)}$ & C11-H14$_{(CHarom.)}$···O3$_{(NO2)}$<br>*O16-H5$_{(COOH)}$···O13$_{(NO2)}$ & C16-H11$_{(CH2)}$···O4$_{(NO2)}$<br>*O16-H5$_{(COOH)}$···O13$_{(NO2)}$ & N8-H15$_{(NH3+)}$···O1$_{(C=Oarom.)}$<br>*N7-H18$_{(NH3+)}$···O8$_{(C=Oarom.)}$ & N8-H15$_{(NH3+)}$···O1$_{(C=Oarom.)}$<br>*N7-H7$_{(NH3+)}$···O1$_{(C=Oarom.)}$ & C17-H13$_{(CH2)}$···O12$_{(NO2)}$<br>*N7-H7$_{(NH3+)}$···O2$_{(NO2)}$ & C17-H13$_{(CH2)}$···O12$_{(NO2)}$

		*N7-H7$_{(NH3+)}$···O2$_{(NO2)}$ & N8-H16$_{(NH3+)}$···O10$_{(NO2)}$<br>*N7-H7$_{(NH3+)}$···O2$_{(NO2)}$ & N8-H17$_{(NH3+)}$···O8$_{(C=Oarom.)}$<br>*N7-H7$_{(NH3+)}$···O2$_{(NO2)}$ & N8-H17$_{(NH3+)}$···O14$_{(NO2)}$<br>*N7-H8$_{(NH3+)}$···O14$_{(NO2)}$ & C17-H14$_{(CH2)}$···O4$_{(NO2)}$
	$D^{2}_{2}(10)$	*N7-H7$_{(NH3+)}$···O1$_{(C=Oarom.)}$ & N8-H16$_{(NH3+)}$···O9$_{(NO2)}$<br>*N7-H7$_{(NH3+)}$···O1$_{(C=Oarom.)}$ & N8-H16$_{(NH3+)}$···O10$_{(NO2)}$<br>*N7-H7$_{(NH3+)}$···O1$_{(C=Oarom.)}$ & N8-H17$_{(NH3+)}$···O8$_{(C=Oarom.)}$<br>*N7-H7$_{(NH3+)}$···O1$_{(C=Oarom.)}$ & N8-H17$_{(NH3+)}$···O8$_{(C=Oarom.)}$<br>*N7-H7$_{(NH3+)}$···O1$_{(C=Oarom.)}$ & N8-H17$_{(NH3+)}$···O14$_{(NO2)}$<br>*N7-H7$_{(NH3+)}$···O2$_{(NO2)}$ & N8-H16$_{(NH3+)}$···O9$_{(NO2)}$<br>*N7-H8$_{(NH3+)}$···O14$_{(NO2)}$ & N8-H15$_{(NH3+)}$···O1$_{(C=Oarom.)}$
	$D^{2}_{2}(11)$	O16-H5$_{(COOH)}$···O13$_{(NO2)}$ & N8-H15$_{(NH3+)}$···O1$_{(C=Oarom.)}$
	$D^{2}_{3}(10)$	N7-H7$_{(NH3+)}$···O2$_{(NO2)}$ & O5-H2···O2$_{(NO2)}$ & N7-H7$_{(NH3+)}$···N7 **ornithine**$_{(NH3+)}$
	$D^{3}_{3}(12)$	*N7-H7$_{(NH3+)}$···O1$_{(C=Oarom.)}$) & C5-H2$_{(CHarom.)}$···O2$_{(NO2)}$ & N7-H7$_{(NH3+)}$···O1$_{(C=Oarom.)}$<br>*(N8-H15$_{(NH3+)}$···O1$_{(C=Oarom.)}$)$_{2}$ & C5-H2$_{(CHarom.)}$···O2$_{(NO2)}$
	$D^{3}_{3}(14)$	*C15-H10$_{(CH2)}$···O4$_{(NO2)}$ & C5-H2$_{(CHarom.)}$···O2$_{(NO2)}$ & C15-H10$_{(CH2)}$···O4$_{(NO2)}$<br>*C16-H11$_{(CH2)}$···O4$_{(NO2)}$ & C5-H2$_{(CHarom.)}$···O2$_{(NO2)}$ & C16-H11$_{(CH2)}$···O4$_{(NO2)}$<br>*C17-H14$_{(CH2)}$···O4$_{(NO2)}$ & C5-H2$_{(CHarom.)}$···O2$_{(NO2)}$ & C17-H14$_{(CH2)}$···O4$_{(NO2)}$
**VUVHYII**	*C*(4)	C8-H19$_{(CH2)}$···O5$_{(COO-)}$
	C(5)	*N1-H1$_{(NH3+)}$···O2$_{(COO-)}$ **ornithine**<br>*N1-H3$_{(NH3+)}$···O2$_{(COO-)}$ **ornithine**<br>*N3-H16$_{(NH3+)}$···O4$_{(COO-)}$<br>*C7-H17$_{(CH)}$···O6$_{(COO-)}$
	C(6)	C4-H8$_{(CH2)}$···O1$_{(COO-)}$ **ornithine**
	C(8)	N2-H13$_{(NH3+)}$······O1$_{(COO-)}$ **ornithine**
	S(6)	*N3-H15$_{(NH3+)}$···O5$_{(COO-)}$<br>*C4-H8$_{(CH2)}$···O2$_{(COO-)}$ **ornithine**
	D(2)	*N1-H2$_{(NH3+)}$···O3$_{(COO-)}$<br>*C3-H6$_{(CH2)}$···O3$_{(COO-)}$<br>*N2-H11$_{(NH3+)}$···O7$_{(OH2)}$<br>*N2-H12$_{(NH3+)}$···O4$_{(COO-)}$<br>*N3-H14$_{(NH3+)}$···O7$_{(OH2)}$<br>*O7-H20$_{(OH2)}$···O6$_{(COO-)}$<br>*O7-H21$_{(OH2)}$···O5$_{(COO-)}$
	$D^{3}_{3}(10)$	N1-H1$_{(NH3+)}$···O2$_{(COO-)}$ **ornithine** & (N1-H2$_{(NH3+)}$···O3$_{(COO-)}$)$_{2}$
level 2	$C^{1}_{2}(4)$	N1-H1$_{(NH3+)}$···O2$_{(COO-)}$ **ornithine** & N1-H3$_{(NH3+)}$···O2$_{(COO-)}$ **ornithine**
	$C^{2}_{2}(6)$	(O7-H21$_{(OH2)}$···O5$_{(COO-)}$)$_{2}$ & O7-H20$_{(OH2)}$···O6$_{(COO-)}$
	$C^{2}_{2}(7)$	C7-H17$_{(CH)}$···O6$_{(COO-)}$ & C8-H19$_{(CH2)}$···O5$_{(COO-)}$
	$C^{2}_{2}(8)$	*O7-H20$_{(OH2)}$···O6$_{(COO-)}$ & N3-H14$_{(NH3+)}$···O7$_{(OH2)}$<br>*O7-H21$_{(OH2)}$···O5$_{(COO-)}$ & N3-H14$_{(NH3+)}$···O7$_{(OH2)}$
	$C^{2}_{2}(9)$	*N1-H1$_{(NH3+)}$···O2$_{(COO-)}$ **ornithine** & C4-H8$_{(CH2)}$···O1$_{(COO-)}$ **ornithine**<br>*N1-H3$_{(NH3+)}$···O2$_{(COO-)}$ **ornithine** & C3-H8$_{(CH2)}$···O1$_{(COO-)}$ **ornithine**<br>*N2-H12$_{(NH3+)}$···O4$_{(COO-)}$ & C3-H6$_{(CH2)}$···O3$_{(COO-)}$<br>*C7-H17$_{(CH)}$···O6$_{(COO-)}$ & C8-H19$_{(CH2)}$···O5$_{(COO-)}$
	$C^{2}_{2}(10)$	*N1-H1$_{(NH3+)}$···O2$_{(COO-)}$ **ornithine** & N1-H3$_{(NH3+)}$···O2$_{(COO-)}$ **ornithine**<br>*N3-H16$_{(NH3+)}$···O4$_{(COO-)}$ & C7-H17$_{(CH)}$···O6$_{(COO-)}$
	$C^{2}_{2}(11)$	*N1-H1$_{(NH3+)}$···O2$_{(COO-)}$ **ornithine** & C4-H8$_{(CH2)}$···O1$_{(COO-)}$ **ornithine**<br>*N1-H1$_{(NH3+)}$···O2$_{(COO-)}$ **ornithine** & N2-H13$_{(NH3+)}$···O1$_{(COO-)}$ **ornithine**<br>*N1-H2$_{(NH3+)}$···O4$_{(COO-)}$ & N2-H12$_{(NH3+)}$···O4$_{(COO-)}$<br>*N1-H3$_{(NH3+)}$···O2$_{(COO-)}$ **ornithine** & N2-H13···O1$_{(COO-)}$ **ornithine**<br>*C8-H19$_{(CH2)}$···O5$_{(COO-)}$ & N3-H16$_{(NH3+)}$···O4$_{(COO-)}$
	$C^{2}_{2}(13)$	N1-H1$_{(NH3+)}$···O2$_{(COO-)}$ **ornithine** & N2-H13$_{(NH3+)}$···O1$_{(COO-)}$ **ornithine**
	$C^{2}_{2}(14)$	N2-H13$_{(NH3+)}$···O1$_{(COO-)}$ **ornithine** & C4-H8$_{(CH2)}$···O1$_{(COO-)}$ **ornithine**
	$C^{3}_{4}(14)$	(N1-H1$_{(NH3+)}$···O2$_{(COO-)}$)$_{2}$ **ornithine** & (N1-H3$_{(NH3+)}$···O2$_{(COO-)}$)$_{2}$ **ornithine**
	$C^{4}_{4}(16)$	(C7-H17$_{(CH)}$···O6$_{(COO-)}$)$_{2}$ & (C8-H19$_{(CH2)}$···O5$_{(COO-)}$)$_{2}$
	$C^{4}_{4}(20)$	*(N1-H1$_{(NH3+)}$···O2$_{(COO-)}$)$_{2}$ **ornithine** & (C4-H8$_{(CH2)}$···O1$_{(COO-)}$)$_{2}$ **ornithine**<br>*N3-H16$_{(NH3+)}$···O4$_{(COO-)}$ & (C7-H17$_{(CH)}$···O6$_{(COO-)}$)$_{2}$
	$R^{1}_{2}(6)$	*(N1-H2$_{(NH3+)}$···O3$_{(COO-)}$)$_{2}$ & C3-H6$_{(CH2)}$···O3$_{(COO-)}$<br>*N2-H13$_{(NH3+)}$···O1$_{(COO-)}$ **ornithine** & C4-H8$_{(CH2)}$···O1$_{(COO-)}$ **ornithine**
	$R^{2}_{2}(11)$	N1-H3$_{(NH3+)}$···O2$_{(COO-)}$ **ornithine** & C4-H8$_{(CH2)}$···O1$_{(COO-)}$ **ornithine**
	$R^{2}_{2}(13)$	N1-H3$_{(NH3+)}$···O2$_{(COO-)}$ **ornithine** & N2-H13$_{(NH3+)}$···O1$_{(COO-)}$ **ornithine**
	$R^{3}_{4}(14)$	(N1-H1$_{(NH3+)}$···O2$_{(COO-)}$)$_{2}$ **ornithine** & (N1-H3$_{(NH3+)}$···O2$_{(COO-)}$)$_{2}$ **ornithine**
	$R^{4}_{4}(18)$	(C7-H17$_{(CH)}$···O6$_{(COO-)}$)$_{2}$ & (C8-H19$_{(CH2)}$···O5$_{(COO-)}$)$_{2}$
	$R^{4}_{4}(20)$	*(N1-H1$_{(NH3+)}$···O2$_{(COO-)}$)$_{2}$ **ornithine** & (C4-H8$_{(CH2)}$···O1$_{(COO-)}$)$_{2}$ **ornithine**<br>*(N3-H16$_{(NH3+)}$···O4$_{(COO-)}$)$_{2}$ & (C7-H17$_{(CH)}$···O6$_{(COO-)}$)$_{2}$

	D^{1}_{2}(3)	N2-H11$_{(NH3+)}$···O7$_{(OH2)}$ & N3-H14$_{(NH3+)}$···O7$_{(OH2)}$
	D^{2}_{2}(4)	*O7-H20$_{(OH2)}$···O6$_{(COO-)}$ & N2-H11$_{(NH3+)}$···O7$_{(OH2)}$<br>*O7-H21$_{(OH2)}$···O5$_{(COO-)}$ & N2-H11$_{(NH3+)}$···O7$_{(OH2)}$
	D^{2}_{2}(5)	N2-H11$_{(NH3+)}$···O7$_{(OH2)}$ & N2-H12$_{(NH3+)}$···O4$_{(COO-)}$
	D^{2}_{2}(7)	*(N1-H2$_{(NH3+)}$···O3$_{(COO-)}$)$_{2}$ & N3-H14$_{(NH3+)}$···O7$_{(OH2)}$<br>*N3-H14$_{(NH3+)}$···O7$_{(OH2)}$ & (C3-H6$_{(CH2)}$···O3$_{(COO-)}$)$_{2}$<br>*N2-H12$_{(NH3+)}$···O4$_{(COO-)}$ & N3-H14$_{(NH3+)}$···O7$_{(OH2)}$
	D^{2}_{2}(8)	*N1-H2$_{(NH3+)}$···O3$_{(COO-)}$ & O7-H20$_{(OH2)}$···O6$_{(COO-)}$<br>*N1-H2$_{(NH3+)}$···O3$_{(COO-)}$ & O7-H21$_{(OH2)}$···O5$_{(COO-)}$<br>*N2-H11$_{(NH3+)}$···O7$_{(OH2)}$ & C3-H6$_{(CH2)}$···O3$_{(COO-)}$<br>*O7-H20$_{(OH2)}$···O6$_{(COO-)}$ & (C3-H6$_{(CH2)}$···O3$_{(COO-)}$)$_{2}$<br>*O7-H21$_{(OH2)}$···O5$_{(COO-)}$ & (C3-H6$_{(CH2)}$···O3$_{(COO-)}$)$_{2}$<br>*N2-H12$_{(NH3+)}$···O4$_{(COO-)}$ & O7-H20$_{(OH2)}$···O6$_{(COO-)}$<br>*N2-H12$_{(NH3+)}$···O4$_{(COO-)}$ & O7-H21$_{(OH2)}$···O5$_{(COO-)}$
	D^{2}_{2}(10)	(N1-H2$_{(NH3+)}$···O3$_{(COO-)}$)$_{2}$ & N2-H11$_{(NH3+)}$···O7$_{(OH2)}$
	D^{2}_{3}(7)	(O7-H21$_{(OH2)}$···O5$_{(COO-)}$)$_{2}$ & C8-H19$_{(CH2)}$···O5$_{(COO-)}$
	D^{2}_{3}(8)	*(N2-H12$_{(NH3+)}$···O4$_{(COO-)}$)$_{2}$ & N3-H16$_{(NH3+)}$···O4$_{(COO-)}$<br>*(O7-H20$_{(OH2)}$···O6$_{(COO-)}$)$_{2}$ & C7-H17$_{(OH2)}$···O6$_{(COO-)}$
	D^{3}_{3}(9)	(O7-H20$_{(OH2)}$···O6$_{(COO-)}$)$_{2}$ & C8-H19$_{(CH2)}$···O5$_{(COO-)}$
	D^{3}_{3}(10)	*N1-H3$_{(NH3+)}$···O2$_{(COO-)}$ **ornithine** & (N1-H2$_{(NH3+)}$···O3$_{(COO-)}$)$_{2}$<br>*(N1-H2$_{(NH3+)}$···O3$_{(COO-)}$)2 & N3-H16$_{(NH3+)}$···O4$_{(COO-)}$<br>*N3-H16$_{(NH3+)}$···O4$_{(COO-)}$ & (C3-H6$_{(CH2)}$···O3$_{(COO-)}$)$_{2}$<br>*(N3-H14$_{(NH3+)}$···O7$_{(OH2)}$)$_{2}$ & N3-H16$_{(NH3+)}$···O4$_{(COO-)}$<br>*(O7-H21$_{(OH2)}$···O5$_{(COO-)}$)$_{2}$ & C7-H17$_{(CH)}$···O6$_{(COO-)}$
	D^{3}_{3}(11)	(C3-H6$_{(CH2)}$···O3$_{(COO-)}$)$_{2}$ & C4-H8$_{(CH2)}$···O1$_{(COO-)}$ **ornithine**
	D^{3}_{3}(12)	*(N1-H1$_{(NH3+)}$···O2$_{(COO-)}$)$_{2}$ **ornithine** & (C3-H6$_{(CH2}$···O3$_{(COO-)}$)$_{2}$<br>*(N1-H2$_{(NH3+)}$···O3$_{(COO-)}$)$_{2}$ & C7-H17$_{(CH)}$···O6$_{(COO-)}$<br>*N1-H2$_{(NH3+)}$···O3$_{(COO-)}$ & (C3-H6$_{(CH2)}$···O3$_{(COO-)}$)$_{2}$<br>*(C3-H6$_{(CH2)}$···O3$_{(COO-)}$)$_{2}$ & C7-H17$_{(CH)}$···O6$_{(COO-)}$<br>*(N2-H12$_{(NH3+)}$···O4$_{(COO-)}$)$_{2}$ & C7-H17$_{(CH)}$···O6$_{(COO-)}$<br>*(N3-H14$_{(NH3+)}$···O7$_{(OH2)}$)$_{2}$ & C7-H17$_{(CH)}$···O6$_{(COO-)}$
	D^{3}_{3}(13)	*(N1-H2$_{(NH3+)}$···O3$_{(COO-)}$)$_{2}$ & C4-H8$_{(CH2}$···O1$_{(COO-)}$ **ornithine**<br>*(C3-H6$_{(CH2}$···O3$_{(COO-)}$)$_{2}$ & C8-H19$_{(CH2)}$···O5$_{(COO-)}$<br>*(N2-H11$_{(NH3+)}$···O7$_{(OH2)}$)$_{2}$ & N2-H13$_{(NH3+)}$···O1$_{(COO-)}$ **ornithine**<br>*(N2-H12$_{(NH3+)}$···O4$_{(COO-)}$)$_{2}$ & N2-H13$_{(NH3+)}$···O1$_{(COO-)}$ **ornithine**<br>*(N2-H12$_{(NH3+)}$···O4$_{(COO-)}$)$_{2}$ & C8-H19$_{(CH2)}$···O5$_{(COO-)}$<br>*(N3-H14$_{(NH3+)}$···O7$_{(OH2)}$)$_{2}$ & C8-H19$_{(CH2)}$···O5$_{(COO-)}$
	D^{3}_{3}(14)	*(O7-H20$_{(OH2)}$···O6$_{(COO-)}$)$_{2}$ & N3-H16$_{(NH3+)}$···O4$_{(COO-)}$<br>*N3-H16$_{(NH3+)}$···O4$_{(COO-)}$ & (O7-H21$_{(OH2)}$···O5$_{(COO-)}$)$_{2}$
	D^{3}_{3}(15)	*(N1-H2$_{(NH3+)}$···O3$_{(COO-)}$)$_{2}$ & N2-H13$_{(NH3+)}$···O1$_{(COO-)}$ **ornithine**<br>*(N2-H11$_{(NH3+)}$···O7$_{(OH2)}$)$_{2}$ & C4-H8$_{(CH2}$···O1$_{(COO-)}$ **ornithine**<br>*(N2-H12$_{(NH3+)}$···O4$_{(COO-)}$)$_{2}$ & C4-H8$_{(CH2}$···O1$_{(COO-)}$ **ornithine**
	D^{3}_{3}(16)	*(N1-H2$_{(NH3+)}$···O3$_{(COO-)}$)$_{2}$ & C8-H19$_{(CH2)}$···O5$_{(COO-)}$<br>*N2-H13$_{(NH3+)}$···O1$_{(COO-)}$ **ornithine** & (C3-H6$_{(CH2}$···O3$_{(COO-)}$)$_{2}$
	D^{3}_{3}(18)	*(N1-H1$_{(NH3+)}$···O2$_{(COO-)}$)$_{2}$ **ornithine** & (N2-H11$_{(NH3+)}$···O7$_{(OH2)}$)$_{2}$<br>*N1-H1$_{(NH3+)}$···O2$_{(COO-)}$ **ornithine** & (N2-H12$_{(NH3+)}$···O4$_{(COO-)}$)$_{2}$<br>*N1-H3$_{(NH3+)}$···O2$_{(COO-)}$ **ornithine** & (N2-H11$_{(NH3+)}$···O7$_{(OH2)}$)$_{2}$<br>*N1-H3$_{(NH3+)}$···O2$_{(COO-)}$ **ornithine** & (N2-H12$_{(NH3+)}$···O4$_{(COO-)}$)$_{2}$
**YIGMAE**	*D*(2)	*N1-H2$_{(NH3+)}$···O9$_{(COO-)}$ **ornithine**<br>*N1-H3$_{(NH3+)}$···F23<br>*N1-H3$_{(NH3+)}$···F26<br>*N1-H4$_{(NH3+)}$···O11$_{(COO-)}$ **ornithine**<br>*C4-H7$_{(CH2)}$···F26<br>*N2-H11$_{(NH3+)}$···O6$_{(COO-)}$ **ornithine**<br>*N2-H12$_{(NH3+)}$···F12<br>*N2-H12$_{(NH3+)}$···F13<br>*N2-H12$_{(NH3+)}$···F16<br>*N2-H12$_{(NH3+)}$···F17<br>*N2-H13$_{(NH3+)}$···F1<br>*N2-H13$_{(NH3+)}$···F3<br>*N2-H13$_{(NH3+)}$···F10<br>*C7-H14$_{(CH)}$···F6<br>*N3-H15$_{(NH3+)}$···O5$_{(COO-)}$ **ornithine**<br>*N3-H16$_{(NH3+)}$···O12$_{(COO-)}$ **ornithine**<br>*N3-H17$_{(NH3+)}$···F4<br>*N3-H17$_{(NH3+)}$···F6

		*N3-H1$_{(NH3+)}$···F10<br>*C8-H18$_{(CH2)}$···F3<br>*C8-H18$_{(CH2)}$···F10<br>*C9-H20$_{(CH2)}$···F12<br>*N4-H24$_{(NH3+)}$···O9$_{(COO-)}$ **ornithine**<br>*N4-H25$_{(NH3+)}$···F22<br>*N4-H25$_{(NH3+)}$···F23
level 2	$D^{1}_{2}(3)$	*N2-H11$_{(NH3+)}$···O6$_{(COO-)}$ **ornithine** & N7-H41$_{(NH3+)}$···O6$_{(COO-)}$ **ornithine**<br>*N1-H3$_{(NH3+)}$···F23 & N10-H65$_{(NH3+)}$···F23<br>*N1-H3$_{(NH3+)}$···F26 & C18-H44$_{(CH2)}$···F26<br>*C18-H44$_{(CH2)}$···F26 & C4-H7$_{(CH2)}$···F26
	$D^{2}_{2}(4)$	*N2-H12$_{(NH3+)}$···F12 & N4-H26$_{(NH3+)}$···F20<br>*N6-H38$_{(NH3+)}$···F13 & N2-H12$_{(NH3+)}$···F13
	$D^{2}_{2}(5)$	*N1-H3$_{(NH3+)}$···F23 & N1-H2$_{(NH3+)}$···O9$_{(COO-)}$<br>*N1-H4$_{(NH3+)}$···O11$_{(COO-)}$**ornithine** & N1-H2$_{(NH3+)}$···O9$_{(COO-)}$ **ornithine**<br>*C18-H44$_{(CH2)}$···F22 & N1-H3$_{(NH3+)}$···F23<br>*N5-H29$_{(NH3+)}$···F21 & N1-H3$_{(NH3+)}$···F23<br>*C4-H7$_{(CH2)}$···F26 & C12-H27$_{(CH)}$···F25<br>*N2-H11$_{(NH3+)}$···O6$_{(COO-)}$ **ornithine** & C25-H61$_{(CH2)}$···O5$_{(COO-)}$ **ornithine**
	$D^{2}_{2}(6)$	*N2-H11$_{(NH3+)}$···O6$_{(COO-)}$ **ornithine** & C12-H27$_{(CH)}$···F25<br>*N2-H11$_{(NH3+)}$···O6$_{(COO-)}$ **ornithine** & N5-H28$_{(NH3+)}$···O8$_{(COO-)}$ **ornithine**
	$D^{2}_{2}(7)$	*N1-H2$_{(NH3+)}$···O9$_{(COO-)}$ **ornithine** & N6-H37$_{(NH3+)}$···O2$_{(COO-)}$ **ornithine**<br>*N1-H2$_{(NH3+)}$···O9$_{(COO-)}$ **ornithine** & N9-H55$_{(NH3+)}$···F14<br>*N2-H11$_{(NH3+)}$···O6$_{(COO-)}$ **ornithine** & C4-H7$_{(CH2)}$···F26<br>*N2-H12$_{(NH3+)}$···F12 & C4-H7$_{(CH2)}$···F26<br>*C7-H14$_{(CH)}$···F6 & C9-H20$_{(CH2)}$···F12
	$D^{2}_{2}(8)$	*N1-H2$_{(NH3+)}$···O9$_{(COO-)}$ **ornithine** & C4-H7$_{(CH2)}$···F26<br>*N1-H4$_{(NH3+)}$···O1$_{(COO-)}$ **ornithine** 1 & C4-H7$_{(CH2)}$···F26
	$D^{2}_{2}(9)$	*N1-H2$_{(NH3+)}$···O9$_{(COO-)}$ **ornithine** & C25-H61$_{(CH2)}$···O5$_{(COO-)}$ **ornithine**<br>*N1-H4$_{(NH3+)}$···O11 **ornithine** & C30-H75$_{(CH2)}$···F22<br>*N4-H25$_{(NH3+)}$···F22 & C7-H14$_{(CH)}$···F6
	$D^{2}_{2}(10)$	*N1-H2$_{(NH3+)}$···O9$_{(COO-)}$ **ornithine** & N2-H11$_{(NH3+)}$···O6$_{(COO-)}$ **ornithine**<br>*N1-H2$_{(NH3+)}$···O9$_{(COO-)}$ **ornithine** & N2-H12$_{(NH3+)}$···F12<br>*N1-H3$_{(NH3+)}$···F23 & N2-H12$_{(NH3+)}$···F12
	$D^{1}_{2}(3)$	*N1-H2$_{(NH3+)}$···O9$_{(COO-)}$ **ornithine** & N4-H24$_{(NH3+)}$···O9$_{(COO-)}$ **ornithine**<br>*N2-H12$_{(NH3+)}$···F16 & N12-H78$_{(NH3+)}$···F16
	$C^{2}_{2}(10)$	N1-H4$_{(NH3+)}$···O11$_{(COO-)}$ **ornithine** & N11-H69$_{(NH3+)}$···O1$_{(COO-)}$ **ornithine**
	$R^{1}_{2}(5)$	N3-H17$_{(NH3+)}$···F6 & C7-H14$_{(CH)}$···F6
	$R^{1}_{2}(6)$	N3-H17$_{(NH3+)}$···F4 & C8-H18$_{(CH2)}$···F10
	$R^{1}_{2}(7)$	N1-H3$_{(NH3+)}$···F26 & C4-H7$_{(CH2)}$···F26
	$R^{2}_{1}(3)$	N2-H12$_{(NH3+)}$···F13 & N2-H12$_{(NH3+)}$···F17
	$R^{2}_{1}(4)$	N1-H3$_{(NH3+)}$···F23 & N1-H3$_{(NH3+)}$···F26
	$R^{2}_{2}(7)$	*N3-H17$_{(NH3+)}$···F4 & C4-H14$_{(CH2)}$···F6<br>*C12-H27$_{(CH)}$···F25 & C13-H31$_{(CH2)}$···F24
	$R^{2}_{2}(8)$	N3-H17$_{(NH3+)}$···F4 & C8-H18$_{(CH2)}$···F3
	$R^{2}_{2}(9)$	N1-H3$_{(NH3+)}$···F23 & C4-H7$_{(CH2)}$···F26
	$R^{2}_{2}(10)$	N1-H2$_{(NH3+)}$···O9$_{(COO-)}$ **ornithine** & N9-H54···O2$_{(COO-)}$ **ornithine**
	$R^{2}_{2}(16)$	N2-H11$_{(NH3+)}$···O6$_{(COO-)}$ **ornithine** & N6-H37$_{(NH3+)}$···O2$_{(COO-)}$ **ornithine**

Table S6. Interplay of interactions (forming synthons) between various functionalities in crystals of ornithine derivatives.

**O-H**$_{(OH, OH2, COOH)}$···**O**$_{(C=O, OH, NO2, OH2, SO4, COO-, COOH)}$
**O-H**$_{(COOH, OH2)}$····**O**$_{(SO4, COOH, COO-, NO2)}$ & **N-H**$_{(NH3+)}$···O$_{(SO4, COOH, NO3-, OH2, COO-, NO2, C=O)}$
**O-H**$_{(OH, OH2, COOH)}$···**O**$_{(C=O, OH, NO2, SO4, COO-, COOH)]2}$ & **C-H**$_{(Carom, CH2, CH, COOH)}$····**O**$_{(COOH, SO4, OH2, COO-, NO3-, NO2, C=O)}$
**N-H**$_{(NH, NH3+,NO3-)}$···O$_{(C=O, COO-, NO3-, OH2, COOH, NO2)}$
**N-H**$_{(NH3+)}$···O$_{(C=O, SO4, COOH, NO3-, COO-, NO2)}$ & **C-H**$_{(CH2, CH2)}$··**O**$_{(C=O, SO4, OH2, COO-, COOH, NO3-, NO2)}$

**C-H**$_{\text{(Carom., CH, CH2)}}$···**O**$_{\text{(COO-, COOH, OH2, SO4, CO, NO3-, NO2}}$
**N-H**$_{\text{(NH3+)}}$···**Br**
**N-H**$_{\text{(NH3+)}}$···**Br** & **O-H**$_{\text{(COOH)}}$···**O**$_{\text{(SO4)}}$
**N-H**$_{\text{(NH3+)}}$···**Br** & **N-H**$_{\text{(NH3+)}}$···**O**$_{\text{(SO4)}}$
**O-H**$_{\text{(COOH, OH2)}}$···**F**
**O-H**$_{\text{(COOH, OH2)}}$···**F** & **C-H**$_{\text{(CH2)}}$···**F**
**O-H**$_{\text{(COOH, OH2)}}$···**F** & **N**$_{\text{(NH3+)}}$**-H**···**O**$_{\text{(OH2)}}$
**O-H**$_{\text{(OH2)}}$···**F** & **C**$_{\text{(CH)}}$**-H**···**O**$_{\text{(OH2)}}$
**N-H**$_{\text{(NH3+)}}$···**F**
**N-H**$_{\text{(NH3+)}}$···**F** & **N**$_{\text{(NH3+)}}$**-H**···**O**$_{\text{(OH2, COOH)}}$
**N-H**$_{\text{(NH3+)}}$···**F** & **C**$_{\text{(CH)}}$**-H**···**O**$_{\text{(OH2)}}$
**N-H**$_{\text{(NH3+)}}$···**F** & **C**$_{\text{(CH2)}}$**-H**···**F**
**C-H**$_{\text{(CH2)}}$···**F**
**C-H**$_{\text{(CH2)}}$···**F** & **N**$_{\text{(NH3+)}}$**-H**···**O**$_{\text{(OH2, COOH)}}$
**C-H**$_{\text{(CH2)}}$···**F** & **C**$_{\text{(CH)}}$**-H**···**O**$_{\text{(OH2)}}$
**O-H**$_{\text{(COOH)}}$···**Cl**
**O-H**$_{\text{(COOH)}}$···**Cl** & **N**$_{\text{(NH3+)}}$**-H**···**O**$_{\text{(C-O-C)}}$
**O-H**$_{\text{(COOH)}}$···**Cl** & **C**$_{\text{(CH2)}}$**-H**···**O**$_{\text{(COOH)}}$
**N-H**$_{\text{(NH3+)}}$···**Cl**
**C-H**$_{\text{(CH2)}}$···**Cl**
**C-H**$_{\text{(CH2)}}$···**Cl** & **N**$_{\text{(NH3+, NH)}}$**-H**···**O**$_{\text{(COO-)}}$
**C-H**$_{\text{(CH2)}}$···**Cl** & **C**$_{\text{(CH2)}}$**-H**···**O**$_{\text{(COO-)}}$
**N-H**$_{\text{(NH3+)}}$···**Cl** & **O**$_{\text{(COOH)}}$**-H**···**O**$_{\text{(SO4)}}$
**N-H**$_{\text{(NH3+)}}$···**Cl** & **N**$_{\text{(NH3+, NH)}}$**-H**···**O**$_{\text{(COO-, NO3-, SO4, C=O, COOH,)}}$
**N-H**$_{\text{(NH3+)}}$···**Cl** & **C**$_{\text{(CH2)}}$**-H**···**O**$_{\text{(COOH, COO-, NO3-)}}$
**N-**H$_{\text{(NH3+)}}$···**Cl** & **O**$_{\text{(COOH)}}$**-H**···**Cl**
**N-H**$_{\text{(NH3+)}}$···**Cl** & **C**$_{\text{(CH2)}}$**-H**···**Cl**

Table S7. Percentage contributions of interatomic contacts to the Hirshfeld surface for (**1**), (**2**) and other ornithine derivatives.

	H···H	O···H	C···H	C···O	C···C	N···H /H···N	O···O	N···O /N···O	Cl···H /H···Cl	Br···H /H···Br	F···O /O···F	F···H /H···F	Cu···Cl /Cl···Cu	Cl···F /F···Cl	Cl···O /O···Cl
**EXPFAU**	53.6	20.1	18.9		1.5										
**(1)**	53.4	23.1	17.9	3.2	1										
**GOTFAY**	12.9	77.2	1.4				6.6	1.6							
**TEFMIA**	10.9	75.2	1.2			1	9.7	1.4							

**ORNBDL10**	45.4	37.1	2				15.5					
**BAPKUB**	39.4	41.4			1.4		16.6					
**BAPKOV**	39	42.1			1.1	16.4						
**EVIJAU**	33.2	54.6			1.6	8.6						
**(2)**	30	65.2	4.3									
**BEZQOO**	26.8	25.3						1.2	45.6			
**IHEPES**	16.5	19.4				33.5		1.5	19.8	2.6	3.3	1.7
**YIGMAE**	27.7	37.6	1.6						31.9			
**VUYHII**	41	56	2.9									
**CAPRAM**	39.9	57.4	1.6									
**BIHYEX**	34.5	64.3										
**BAPKIP**	29.9	66.9										
**JADGED**	30	65.2	4.3									
**PUYVUA**	29.7	67.2	1.8	1.2								

Table S8. Properties of ornithine moiety in analyzed structures, as derived from HS calculations.

	Volume [Å^3]	*Area* [Å^2]	*Globularity*	*Asphericity*
**EXOFAY**	437.70	402.46	0.693	0.334
**(1)**	490.02	414.82	0.725	0.080
**GOTFAY**	170.58	182.82	0.814	0.111
**TEFMIA**	168.25	179.65	0.820	0.118
**ORNBDL10**	159.68	171.08	0.832	0.130
**BAPKUP**	168.92	177.59	0.832	0.088
**BAPKOV**	168	176.45	0.835	0.089
**EVIJAU**	168.20	177.50	0.830	0.089
**(2)**	161.12	172.85	0.828	0.138
**BEZQOO**	176.26	181.92	0.836	0.136
**IHEPES**	257.96	254.61	0.770	0.028
**YIGMAE**	165.89	177.66	0.822	0.119
**VUYHII**	160.49	170.86	0.836	0.132
**CAPRAM**	162.17	173.91	0.827	0.131

**BIHYEX**	180.48	184.95	0.835	0.144
**BAPKIP**	178	183.88	0.832	0.126
**JADGED**	162.94	172.7	0.835	0.102
**PUYVUA**	163.96	172.54	0.840	0.123

Table S9. Water solubility predictions of ornithine-based compounds.

Compound	LogP (concensus LogP)	LogS (ESOL)	LogS (Ali)	LogS (SILICOS-IT)
DFMO	-0.87	highly soluble	highly soluble	soluble
(**1**)	1.81	soluble	soluble	moderately soluble
EXOFAY	0.06	soluble	soluble	soluble
GOTFAY	-2.75	highly soluble	highly soluble	soluble
TEFMIA	-1.24	highly soluble	soluble	soluble
(**2**)	-3.83	highly soluble	highly soluble	soluble
ORNBDL10	-3.76	highly soluble	highly soluble	soluble
BAPKUB	-4.56	highly soluble	highly soluble	soluble
BAPKOV	-4.63	highly soluble	highly soluble	soluble
EVIJAU	-4.49	highly soluble	highly soluble	soluble
BEZQOO	-1.84	highly soluble	highly soluble	soluble
YIGMAE	-1.83	highly soluble	highly soluble	soluble
IHEPES	-0.73	soluble	soluble	soluble
VUYHII	-6.60	highly soluble	highly soluble	soluble
CAPRAM	-6.60	highly soluble	highly soluble	soluble
BIHYEX	-4.72	highly soluble	highly soluble	soluble
BAPKIP	-4.39	highly soluble	highly soluble	soluble
JADGED	-4.49	highly soluble	highly soluble	soluble
PUYVUA	-3.23	highly soluble	highly soluble	soluble

Table S10. Pharmacokinetic toxicity parameters obtained *via* pkCSM web server.

AMES toxicity	no
Max. tolerated dose (human) [log mg/kg/day]	0.199
hERG I inhibitor	no

hERG II inhibitor	no
Oral rat acute toxicity (LD50) [mol/kg]	2.456
Oral rat chronic toxicity (LOAEL) [log mg/kg_bw/day]	2.914
hepatoxicity	yes
Skin sensitisation	no
T. Pyriformis toxicity [log ug/L]	0.296
Minnow toxicity [log mM]	2.265

Table S11. Toxicity parameters for (**1**).

Oral toxicity prediction results for input compound

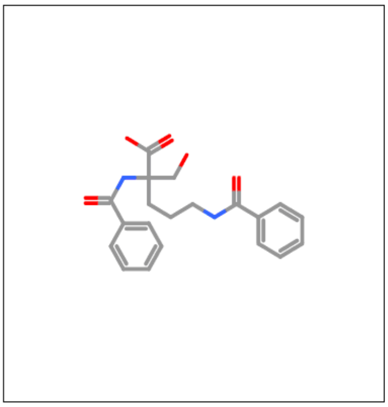

Predicted LD50: 5000mg/kg

Predicted Toxicity Class: 5

1 2 3 4 5 6

Average similarity: 87.65%

Prediction accuracy: 70.97%

20% 40% 60% 80%

Name	OCC(C(=O)O)(NC(=O)c1ccccc1)CCCN
Molweight	370.4
Number of hydrogen bond acceptors	7
Number of hydrogen bond donors	0
Number of atoms	27
Number of bonds	28
Number of rings	2
Number of rotable bonds	11
Total charge	0
Molecular Polar Surface Area	115.73

Table S12. Toxicity model report for (**1**).

Toxicity Model Report

Classification	Target	Shorthand	Prediction	Probability
Organ toxicity	Hepatotoxicity	dili	Inactive	0.86
Toxicity end points	Carcinogenicity	carcino	Inactive	0.67
Toxicity end points	Immunotoxicity	immuno	Inactive	0.98
Toxicity end points	Mutagenicity	mutagen	Inactive	0.72
Toxicity end points	Cytotoxicity	cyto	Inactive	0.78
Tox21-Nuclear receptor signalling pathways	Aryl hydrocarbon Receptor (AhR)	nr_ahr	Inactive	0.92
Tox21-Nuclear receptor signalling pathways	Androgen Receptor (AR)	nr_ar	Inactive	0.95
Tox21-Nuclear receptor signalling pathways	Androgen Receptor Ligand Binding Domain (AR-LBD)	nr_ar_lbd	Inactive	0.98
Tox21-Nuclear receptor signalling pathways	Aromatase	nr_aromatase	Inactive	0.92
Tox21-Nuclear receptor signalling pathways	Estrogen Receptor Alpha (ER)	nr_er	Inactive	0.90
Tox21-Nuclear receptor signalling pathways	Estrogen Receptor Ligand Binding Domain (ER-LBD)	nr_er_lbd	Inactive	0.99
Tox21-Nuclear receptor signalling pathways	Peroxisome Proliferator Activated Receptor Gamma (PPAR-Gamma)	nr_ppar_gamma	Inactive	0.96
Tox21-Stress response pathways	Nuclear factor (erythroid-derived 2)-like 2/antioxidant responsive element (nrf2/ARE)	sr_are	Inactive	0.96
Tox21-Stress response pathways	Heat shock factor response element (HSE)	sr_hse	Inactive	0.96
Tox21-Stress response pathways	Mitochondrial Membrane Potential (MMP)	sr_mmp	Inactive	0.87
Tox21-Stress response pathways	Phosphoprotein (Tumor Supressor) p53	sr_p53	Inactive	0.94
Tox21-Stress response pathways	ATPase family AAA domain-containing protein 5 (ATAD5)	sr_atad5	Inactive	0.97
